# Bruceine A protects nuclear receptor 4A1 from ubiquitin-degradation to alleviate mesangial proliferative glomerulonephritis

**DOI:** 10.1038/s41392-025-02495-2

**Published:** 2025-12-05

**Authors:** Huating Hu, Runze Li, Kancheng He, Lingling Wu, Rongrong Li, Jiayan Lu, Ruimin Tian, Chuanghai Zhang, Jiayan He, Yulian Chen, Ruogu Lai, Jiaqi Zhang, Jiaqi Wu, Ying Zheng, Jinlian He, Liang Liu, Xiangmei Chen, Hudan Pan

**Affiliations:** 1 State Key Laboratory of Traditional Chinese Medicine Syndrome, The Second Affiliated Hospital of Guangzhou University of Chinese Medicine, Guangzhou, China; 2 Department of Integrated Traditional Chinese and Western Medicine, The First People’s Hospital of Foshan, Foshan, China; 3 Department of Urology, The First People’s Hospital of Foshan, Foshan, China; 4 Department of Nephrology, First Medical Center of Chinese PLA General Hospital, National Key Laboratory of Kidney Diseases, National Clinical Research Center for Kidney Diseases, Beijing Key Laboratory of Kidney Diseases Research, Beijing, China

**Keywords:** Kidney diseases, Target identification

## Abstract

The nuclear receptor 4A1(NR4A1) plays a crucial role in maintaining cellular homeostasis and is involved in various disease processes; however, its functional role and pharmacological potential in mesangial proliferative glomerulonephritis (MsPGN) remain unexplored. In this study, we found that downregulation of NR4A1 promotes the pathogenesis of MsPGN by regulating inflammatory and proliferative responses in mesangial cells (MCs), whereas overexpression of NR4A1 reverses these processes. Bruceine A (BA) binds to NR4A1 at residues D481/Q568 and exhibits NR4A1-dependent anti-inflammatory and anti-proliferative effects both in vitro and in vivo. Notably, adeno-associated virus serotype 9 (AAV9)-mediated overexpression of NR4A1 alleviates glomerular injury and inflammatory cascades, while knockout of NR4A1 impairs the renoprotective effects of BA. BA binds to the ligand-binding domain (LBD) of NR4A1 and further sterically blocks K48-linked polyubiquitination at K558, thereby stabilizing NR4A1 protein levels. This stabilization enables NR4A1 to auto-activate its own promoter, amplifying the transcriptional repression of nuclear factor kappa-B (NF-κB) signaling phosphorylation, which ultimately attenuates inflammatory cascades and mesangial proliferation to confer renal protection. This study provides a promising therapeutic avenue for the development of next-generation therapies against MsPGN.

## Introduction

Mesangial proliferative glomerulonephritis (MsPGN), a major pathological subtype of primary glomerulonephritis, presents a critical global clinical challenge. As its most common form, IgAN is characterized by regional immune injury that drives disease progression in the absence of targeted therapies.^1^ Epidemiological studies indicate that MsPGN accounts for 29.7%–59.5% of primary glomerulonephritis cases and serves as a leading cause of end-stage renal disease (ESRD) progression.^1^ In China, 20%–40% of MsPGN patients advance to ESRD, highlighting its substantial disease burden.^2^

Pathologically, MsPGN is defined by two core features: pathological proliferation of mesangial cells (MCs) and aberrant deposition of extracellular matrix (ECM).^3^ These processes trigger glomerulosclerosis and interstitial fibrosis, ultimately culminating in irreversible renal failure. Mechanistically, immune complex-mediated inflammatory cascades play a pivotal role. The anti-Thy1 nephritis model effectively mimics the dual pathological changes of human MsPGN, including renal inflammatory infiltration and abnormal MCs proliferation,^4,5^ making it an ideal animal model for MsPGN research. The anti-Thy1 antibody binding triggers complement activation, induces MCs injury, and promotes in situ immune complex deposition, which synergistically activates cytokine-mediated signaling pathways to provoke MCs hyperproliferation and ECM expansion.^6^ Despite these advances in elucidating the molecular mechanisms, current therapies lack specificity for targeting MCs inflammatory and proliferation—the key drivers of MsPGN progression.^7^ This gap underscores the urgent need to identify novel molecular targets and develop new therapeutic strategies.

Nuclear receptor 4A1(NR4A1, also called Nur77, TR3), is an essential nuclear receptor superfamily member. Emerging evidence implicates NR4A1 as a dual regulator of inflammation and proliferation, with significant implications for renal pathophysiology.^8,9^ However, its role in MsPGN-associated inflammation and MCs proliferation remain unclear. This regulatory function makes NR4A1 a potential “master switch” at the crossroads of MCs proliferation and immune dysregulation—a hypothesis requiring systematic exploration. The NR4A1 protein consists of several functional domains, including an N-terminal domain, a DNA-binding domain, and a ligand-binding domain (LBD). Unlike other nuclear receptors, the LBD region of NR4A1 does not have a “pocket-like” crystal structure that facilitates ligand binding; instead, it maintains a constitutively active state independent of ligands.^10^ To date, no clear endogenous ligand has been found for NR4A1, leading to its designation as an orphan receptor. Nevertheless, some small-molecule compounds—such as Cytosporone B (CsnB)—have been shown to bind the LBD of NR4A1 and regulate its activity.^11^ Thus, identifying natural small-molecule ligands that can bind NR4A1 is of great importance.

Bruceine A (BA), a natural compound derived from Brucea javanica, exhibits biological activities that align with the pathogenic drivers of MsPGN. Beyond its established anti-inflammatory effects in diabetic nephropathy,^12^ BA also has unique antiviral properties^13^ and dual anti-tumor actions.^14,15^ However, its capacity to modulate MCs inflammation and proliferation remains unexplored.

Herein, this study intends to identify BA as a potential natural ligand for the orphan receptor NR4A1, while providing a targeted therapeutic strategy that addresses the core pathology of MsPGN. By linking this natural compound to the specific modulation of NR4A1 in MsPGN, our work is expected to offer new insights and a potential breakthrough for this disease that currently lacks specific treatments.

## Results

### NR4A1 downregulation promotes IgAN progression *via* modulating inflammatory and proliferative responses in MCs

**Fig. 1:**
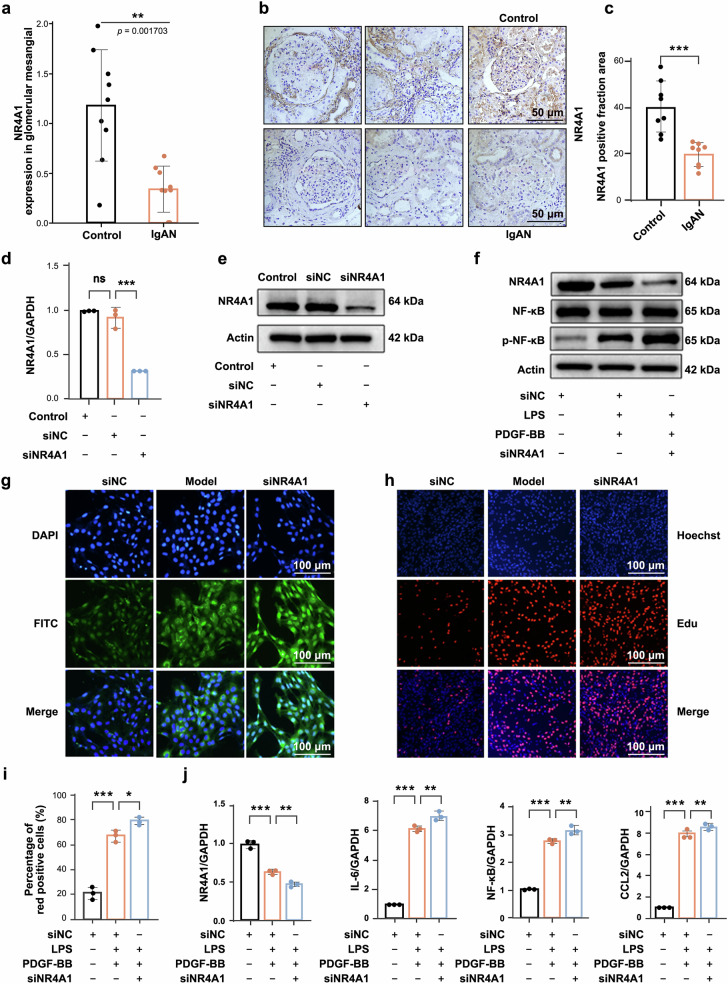
NR4A1 downregulation promotes IgAN progression *via* modulating inflammatory and proliferative responses in MCs. **a** Single-cell RNA sequencing revealed significant downregulation of NR4A1 in MCs from IgAN patients compared to controls. **b**, **c** Representative immunohistochemistry images showing NR4A1 expression in renal tissues from patients with IgAN and healthy controls. **d**, **e** siRNA knocked down the gene and protein expression of NR4A1. **f** Western blot was performed to quantify NR4A1 and NF-κB/p-NF-κB protein levels. **g** Effect of NR4A1 knockdown on CCL2 protein expression. **h**, **i** Edu cell proliferation experiment. **j** Effect of NR4A1 knockdown on inflammation and proliferation-related genes. Data were expressed as mean ± SD (*n* = 3), *p < 0.05, **p < 0.01, ***p < 0.001 vs model group

To identify key pathogenic targets in IgAN, we collected clinical data from the GSE93798 and GSE37460 datasets and analyzed them using R software. A total of 47 IgAN patients and 31 healthy controls were included, with volcano plots of differentially expressed genes (DEGs) presented in supplementary Fig. 1a. Using transcriptome data from the anti-Thy1 nephritis rat model, we finally identified 14 DEGs, among which NR4A1 was significantly downregulated in renal tissues of IgAN (supplementary Fig. 1b). Functional enrichment analysis linked these DEGs to inflammatory and fibrotic pathways (supplementary Fig. 2a, b), while module analysis identified NR4A1, not NR4A2 or NR4A3, as the most critical hub gene (supplementary Fig. 3a). The Nephroseq V5 database further confirmed reduced NR4A1 expression in IgAN patients and its positive correlation with eGFR (supplementary Fig. 3b, c). Next, we analyzed in-house single-cell transcriptome sequencing data from IgAN cases and matched controls, which showed significantly downregulated NR4A1 expression in MCs of IgAN kidney tissues (Fig. 1a). Further verification *via* paraffin sections confirmed a marked reduction in NR4A1 protein in IgAN kidneys (Fig. 1b, c, supplementary tables 3, 4 and 5, 6).

To determine whether NR4A1 contributes to IgAN development, we silenced NR4A1 in MCs *via* siRNA and established an inflammatory/proliferative model using lipopolysaccharide (LPS) and platelet-derived growth factor-BB (PDGF-BB). NR4A1 knockdown significantly reduced its expression (Fig. 1d, e), while increasing phosphorylated NF-κB and CCL2 protein levels (Fig. 1f, g). Moreover, NR4A1 depletion enhanced MCs proliferation, as demonstrated by Edu and CCK-8 assays (Fig. 1h, i and supplementary Fig. 4a); consistent results were observed in colony formation experiments (supplementary Fig. 4b, c). To determine whether NR4A1 regulates MCs proliferation by affecting the cell cycle, we observed that MCs in the model group accumulated in the S phase, an effect further exacerbated by NR4A1 knockdown (supplementary Fig. 4d–f). Additionally, NR4A1 knockdown increased the mRNA levels of IL-6, CCL2, NF-κB, Cyclin E and α-SMA (Fig. 1j and supplementary Fig. 4g). Collectively, these data provide evidence that NR4A1 acts as a critical regulator in IgAN progression, contributing to pathogenesis by coordinately modulating inflammatory signaling and proliferative responses.

### NR4A1 overexpression reverses the inflammatory response and proliferation of MCs

**Fig. 2:**
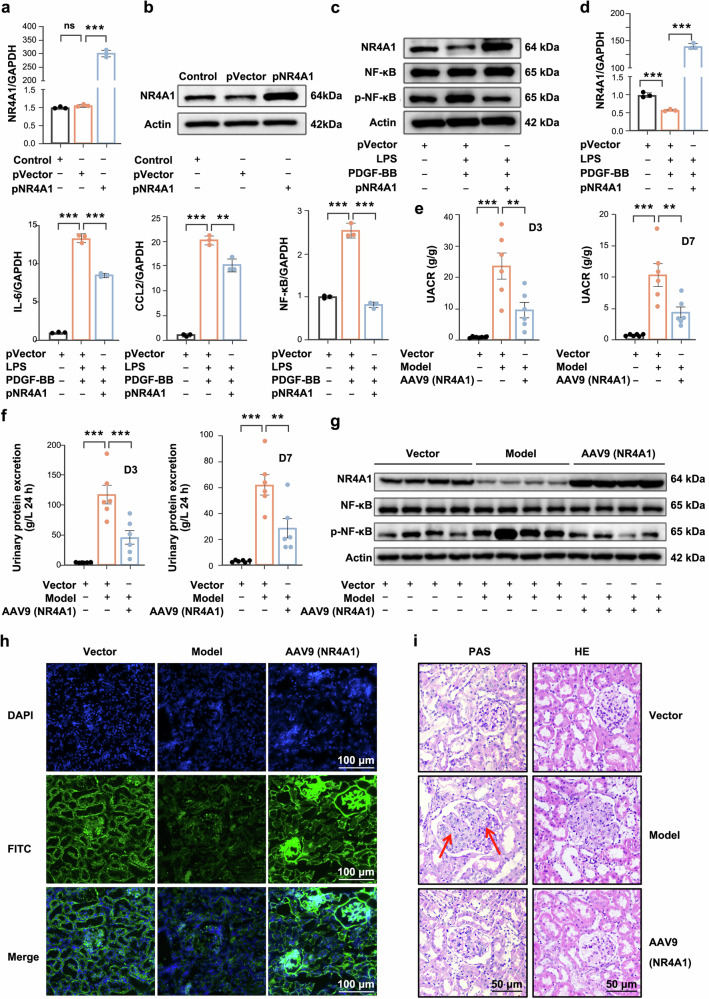
NR4A1 overexpression reverses the inflammatory response and proliferation of MCs. **a**, **b** NR4A1 overexpression plasmid increased the expression of NR4A1 gene and protein in MCs. **c** Western blot analysis of NR4A1 and NF-κB/p-NF-κB protein expression. **d** Effect of NR4A1 on inflammation-related genes. Data were expressed as mean ± SD (*n* = 3), *p < 0.05, **p < 0.01, ***p < 0.001 vs model group. **e**, **f** Effect of NR4A1 overexpression on UACR and 24 h urine protein quantification. **g** Western blot analysis of the changes in NR4A1 and NF-κB/p-NF-κB proteins. **h** Renal immunofluorescence showed changes in NR4A1 protein. Bars =100 µm. Magnification: ×200. **i** Representative images of glomeruli from AAV9-NR4A1-treated rats stained with PAS and HE staining, Bars = 50 µm. Magnification: ×400. Data were shown as mean ± SEM (*n* = 6). Statistical significance was determined as follows: *p < 0.05, **p < 0.01, ***p < 0.001 vs model group

To confirm whether NR4A1 overexpression protects against MCs proliferation and inflammation, we transfected MCs with an NR4A1 overexpression plasmid, which significantly increased NR4A1 mRNA and protein levels compared to the vector control (Fig. 2a, b). In LPS and PDGF-BB-stimulated MCs, NR4A1 overexpression inhibited the expression levels of phosphorylated NF-κB and CCL2 protein (Fig. 2c and supplementary Fig. 5a, b), as well as the mRNA levels of inflammation and proliferation-related genes (Fig. 2d and supplementary Fig. 5c). In addition, CCK8 and colony formation assays revealed that NR4A1 overexpression inhibits MCs proliferation (supplementary Fig. 5d–f). Consistently, Edu cell proliferation assays and flow cytometry-based cell cycle analyses showed that NR4A1 overexpression also suppresses S-phase replication and induces G0/G1 phase arrest in MCs (supplementary Fig. 5g–k).

Notably, we further confirmed that overexpression of NR4A1 inhibited the inflammatory response and the proliferation of MCs in vivo experiments by an AAV9 adeno-associated virus vector containing the NR4A1 plasmid, which was transfected into rats by renal vein injection. Results showed that overexpression of NR4A1 reduced the urinary albumin-to-creatinine ratio (UACR) and 24-hour urinary protein quantification (Fig. 2e, f), inducing inhibition of MCs proliferation and mesangial matrix deposition, thereby improving kidney lesions (Fig. 2i and supplementary Fig. 6a–c). Furthermore, alongside upregulated NR4A1 protein expression, phosphorylated NF-κB protein was inhibited (Fig. 2g). High NR4A1 protein expression was also observed in glomeruli (Fig. 2h), accompanied by downregulated genes associated with inflammation, proliferation, and fibrosis (supplementary Fig. 6d). Consistent with this, the protein levels of PCNA, α-SMA, and FN showed a similar trend (supplementary Fig. 6e–h). These findings indicate that NR4A1 overexpression mitigates disease progression by suppressing mesangial inflammation and proliferation.

### BA binds to NR4A1 with high affinity and forms close interactions

**Fig. 3:**
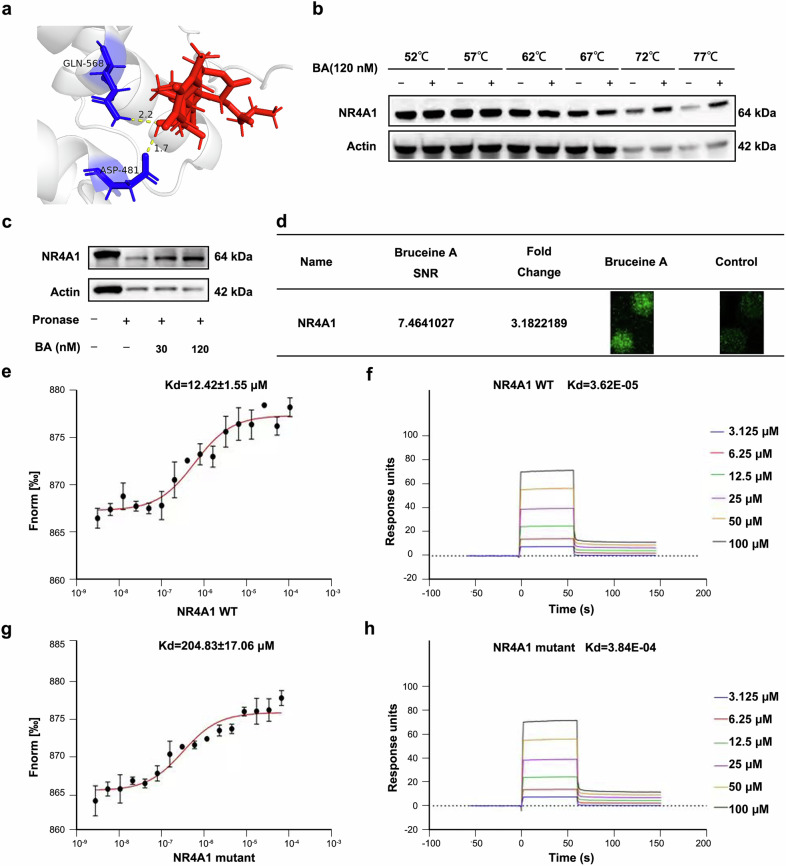
BA binds to NR4A1 with high affinity and forms close interactions. **a** Molecular docking diagram of the NR4A1 agonist and BA. **b** BA and NR4A1 protein interaction using CETSA assay. **c** BA and NR4A1 protein interaction using DARTS assay. **d** Binding proteins identification of BA using a human proteome microarray. **e** Affinity between BA and the NR4A1/LBD WT protein measured by MST. **f** Affinity between BA and the NR4A1/LBD WT protein measured by SPR. **g** MST assay detected the affinity between BA and NR4A1/LBD mutant protein. **h** SPR assay detected the affinity between BA and NR4A1/LBD mutant protein

To identify candidate compounds that bind to NR4A1, more than 20 nephroprotective compounds were selected from Traditional Chinese Medicine (TCM) databases and relevant literature for molecular docking analysis, with the docking scores presented in supplementary Fig. 7a. The docking region was designated as the ligand-binding pocket of NR4A1, a site known to interact with CsnB, a well-recognized NR4A1 activator. Molecular docking results revealed that BA theoretically exhibits strong binding affinity for NR4A1 (affinity = −8.00 kcal/mol) and, similar to CsnB, binds to the same pocket within NR4A1 (supplementary Fig. 7b). Specifically, BA forms up to four hydrogen bonds with glutamine 568 (Q568) and aspartic acid 481 (D481) in the LBD of NR4A1, suggesting enhanced binding stability (Fig. 3a).

To further confirm whether BA binds to NR4A1, we conducted CETSA and DARTS experiments in vitro. Results demonstrated that BA protects NR4A1 from proteolytic and high-temperature degradation in a dose-dependent manner (Fig. 3b, c). Meanwhile, human proteome microarray screening independently validated NR4A1 as a BA-binding target (Fig. 3d). Notably, we also verified the binding affinity between BA and NR4A1 using SPR, MST, and BLI assays. MST results showed a Kd value of 12.42 ± 1.55 μM (Fig. 3e), while SPR and BLI analyses yielded Kd values of 3.62 × 10⁻⁵ M and 9.69 × 10⁻⁵ M, respectively (Fig. 3f and supplementary Fig. 7c). To pinpoint BA-NR4A1 binding specificity, we generated NR4A1 mutants (D481A and Q568A) based on docking predictions and performed MST and SPR assays. Strikingly, D481A/Q568A mutagenesis significantly reduced binding affinity, with MST Kd increasing to 204.83 ± 17.06 μM (Fig. 3g) and SPR Kd rising to 3.84 × 10⁻⁴ M (Fig. 3h), confirming these residues as critical interaction sites. Collectively, these results demonstrate that BA binds to D481 and Q568 within the LBD of NR4A1.

### BA attenuates proteinuria and pathological alterations in anti-Thy1 rat nephritis model *via* NR4A1

**Fig. 4:**
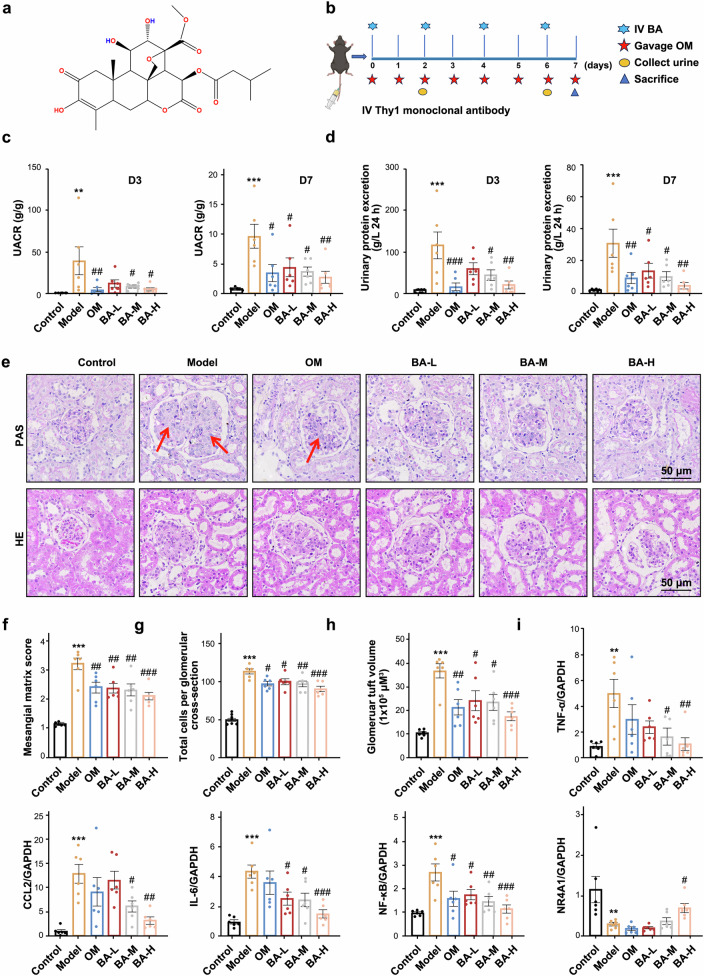
Effects of BA on proteinuria and pathological changes. **a** The chemical structure of BA. **b** Experimental flow diagram. **c**, **d** Effects of BA on 24 h urinary protein excretion and UACR on days 3 and 7 in anti-Thy1 nephritis rats. **e**–**h** Representative images of glomeruli from BA or OM treated animals with anti-Thy1 nephritis stained with PAS and HE staining, Bars = 50 µm. Magnification: ×400. **i** Effects of BA on IL-6, CCL2, NF-κB, TNF-α and NR4A1. Data were presented as the mean ± SEM (*n* = 6). Statistical significance was determined as follows: *p < 0.05, **p < 0.01, ***p < 0.001 vs the control group; #*p* < 0.05, ##*p* < 0.01, ###*p* < 0.001 vs the model group

As shown in Fig. 4a, b, BA was administered to anti-Thy1 nephritis rats’ model according to the outlined experimental timeline. BA treatment induced a dose-dependent reduction in 24-hour urinary protein excretion and UACR (Fig. 4c, d), verifying its therapeutic efficacy in ameliorating glomerular dysfunction. The anti-Thy1 nephritis rat model exhibited severe glomerular hypertrophy, MCs hyperplasia, and mesangial matrix deposition, accompanied by focal capillary loop compression and nodular lesions. BA treatment markedly attenuated these structural abnormalities, suggesting a protective role in glomerular remodeling (Fig. 4e–h). Meanwhile, protein levels of PCNA, α-SMA and FN were elevated in the model group but reduced by BA in a dose-dependent manner, whereas NR4A1 showed the opposite trend (supplementary Fig. 8a–e). Additionally, BA reduced the mRNA levels of genes related to inflammation, proliferation, and fibrosis (Fig. 4i and supplementary Fig. 8f). To elucidate the mechanism underlying BA’s effects, we performed transcriptome sequencing to identify DEGs, and functionally characterized their biological significance using GO/KEGG pathway analysis and GSEA (supplementary Fig. 9a, b, 10a, b,11a, b, 12a, b). GSEA further revealed that BA intervention downregulated inflammation, cell cycle, and fibrosis-related pathways, particularly the NF-κB pathway (supplementary Fig. 13a, 14a). Notably, BA significantly upregulated renal NR4A1 protein levels while suppressing CCL2 protein expression (supplementary Fig. 15a–d).

### BA attenuates MCs proliferation and inflammation through NR4A1/NF-κB signaling

**Fig. 5:**
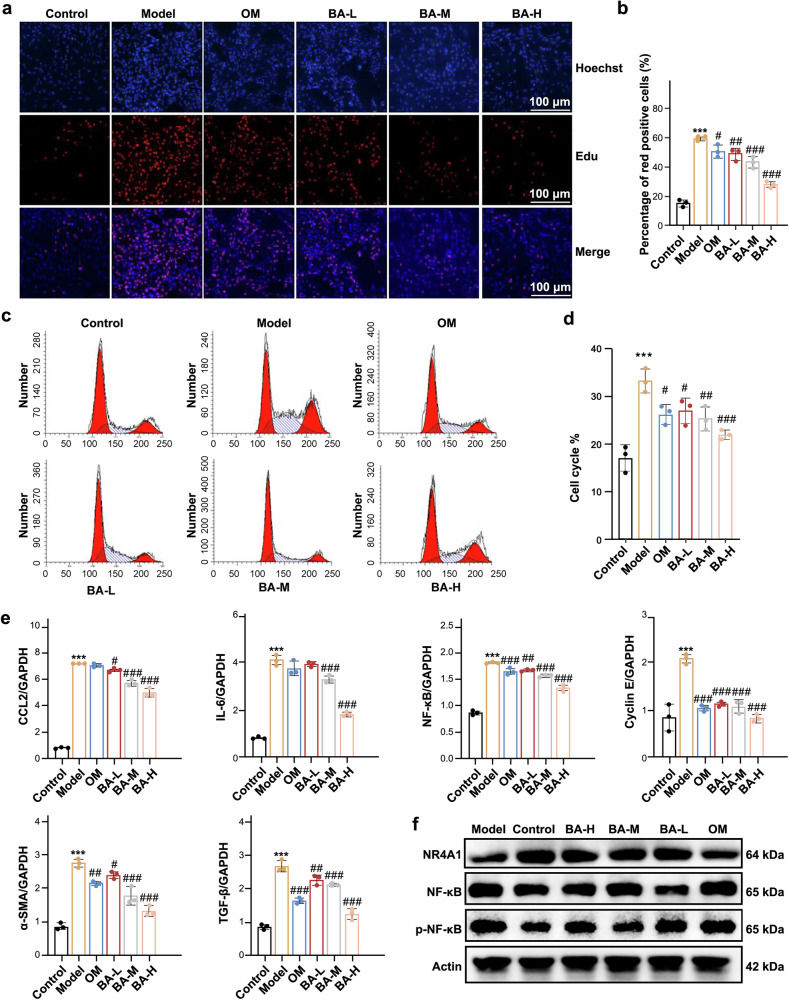
BA inhibits MCs proliferation and inflammation in vitro. **a**, **b** Effect of BA on inhibiting the proliferation of MCs by Edu incorporation assay. **c**, **d** Flow cytometry analysis of the effects of BA on the cell cycle distribution of MCs. **e** Effects of BA on IL-6, CCL2, NF-κB, α-SMA, TGF-β1 and Cyclin E. **f** Western blot analysis of NR4A1 and NF-κB/p-NF-κB protein expression. Data were presented as the mean ± SEM (*n* = 3). Statistical significance was determined as follows: *p < 0.05, **p < 0.01, ***p < 0.001 vs the control group; #*p* < 0.05, ##*p* < 0.01, ###*p* < 0.001 vs the model group

To evaluate BA’s pharmacological activity in vitro, rat MCs were stimulated with 100 ng/mL LPS and 50 ng/mL PDGF-BB for 24 h, followed by 24 h treatment with BA (30, 60, 120 nM) or 50 μM Olmesartan (OM) as a positive control. Results showed that BA dose-dependently inhibited MCs proliferation. To detect whether BA inhibited MCs proliferations by affecting the cell cycle, we observed an increased ratio of Edu-positive signals in the model group, which was reduced by BA (Fig. 5a, b and supplementary Fig. 16a–e). In the model group, MCs were stimulated to enter the S phase, with a decreased G0/G1 phase ratio, thereby enhancing proliferation; BA inhibited this proliferation by regulating the S-phase to G0/G1 phase transition and reducing S-phase levels (Fig. 5c, d, and supplementary Fig. 16f). Furthermore, BA downregulated the mRNA levels of IL-6, CCL2, NF-κB, Cyclin E, TGF-β, and α-SMA (Fig. 5e) and reduced CCL2 protein expression (supplementary Fig. 16g, h). It was also confirmed that BA increased NR4A1 protein expression while diminishing phosphorylated NF-κB signaling in vitro, suggesting that BA might inhibit inflammation and MCs proliferation through the NR4A1/NF-κB pathway (Fig. 5f).

### BA binds to NR4A1 in a competitive manner with CsnB in vitro *and* in vivo

**Fig. 6:**
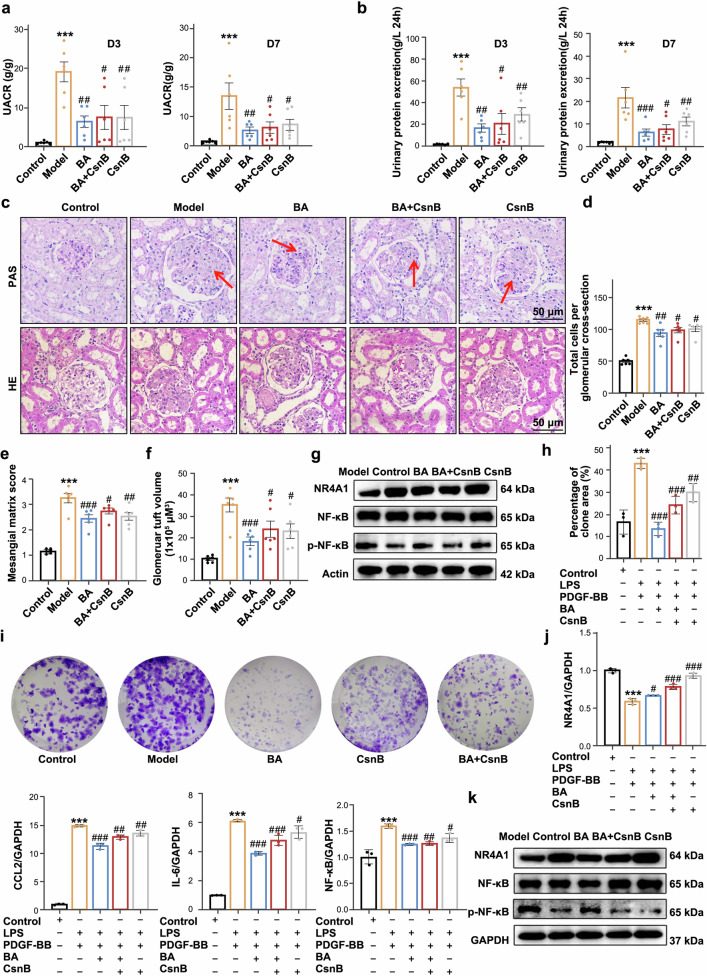
BA binds to NR4A1 in a competitive manner with CsnB in vitro and in vivo (**a**, **b**) Effects of BA on 24 h urinary protein excretion and UACR. **c** Representative images of glomeruli from BA or OM treated animals with anti-Thy1 nephritis stained with PAS and HE staining, Bars = 50 µm. Magnification: ×400. **d**–**f** Semi-quantitative analysis of renal pathological changes. **g** Western blot analysis of NR4A1 and NF-κB/p-NF-κB protein expression in each group. Data were presented as the mean ± SEM (*n* = 6). Statistical significance was determined as follows: *p < 0.05, **p < 0.01, ***p < 0.001 vs the control group; #*p* < 0.05, ##*p* < 0.01, ###*p* < 0.001 vs the model group. **h**, **i** Colony formation assay. **j** BA and CsnB on the genes NR4A1, IL-6, CCL2, NF-κB, α-SMA and Cyclin E in vitro. **k** Western blot analysis of NR4A1 and NF-κB/p-NF-κB protein expression in each group. Data were presented as the mean ± SD (*n* = 3). Statistical significance was determined as follows: *p < 0.05, **p < 0.01, ***p < 0.001 vs the control group; #*p* < 0.05, ##*p* < 0.01, ###*p* < 0.001 vs the model group

To demonstrate the therapeutic effect of BA in alleviating proteinuria and renal pathology by targeting NR4A1, the NR4A1 agonist CsnB was used in an anti-Thy1 nephritis model. The experimental design is outlined in supplementary Fig. 17a. BA was more effective than CsnB or the combination of BA and CsnB in reducing 24-hour urinary protein and UACR (Fig. 6a, b). Semi-quantitative analysis revealed that BA significantly decreased MCs proliferation and mesangial matrix deposition, with more pronounced pathological improvements compared to the CsnB group and the BA+CsnB combination group (Fig. 6c–f). Additionally, after BA and CsnB treatment, FN, PCNAand α-SMA scores were reduced in the model group, while BA had the lowest scores (supplementary Fig. 17b–e). BA also reduced the renal organ index, indicating improved glomerular hypertrophy (supplementary Fig. 17f). Consistent with the trends in urinary protein levels, BA and CsnB modulated the gene expression of NR4A1, IL-6, CCL2, NF-κB, Cyclin E and Cyclin B (supplementary Fig. 18a), while upregulating NR4A1 protein and downregulating phosphorylated NF-κB (Fig. 6g).

To determine whether BA inhibits the inflammatory response and MCs proliferation by binding to NR4A1 in vitro, we used CsnB in an LPS and PDGF-BB-induced MCs proliferation and inflammation model. We observed downregulated NR4A1 expression in the model group. Notably, CsnB increased NR4A1 gene expression in MCs after 48 h of stimulation (supplementary Fig. 19a). BA also significantly reduced MCs proliferation, but this effect was partially reversed when combined with CsnB (Fig. 6h, i, supplementary Fig. 19b-d). Moreover, BA, CsnB, and their combination significantly downregulated the mRNA levels of NR4A1, IL-6, CCL2, NF-κB, Cyclin E and α-SMA (Fig. 6j and supplementary Fig. 19e). Both BA and CsnB significantly increased NR4A1 protein expression, with the combination group showing intermediate levels—indicating that BA and CsnB bind to NR4A1 in a competitive manner (Fig. 6k).

### BA alleviates anti-Thy1 nephritis dependent on NR4A1

To determine whether BA exerts its therapeutic effects through NR4A1, we constructed an NR4A1 knockout (NR4A1^−/−^) model (Wistar) using CRISPR/Cas-mediated genome engineering. The experimental design is outlined in supplementary Fig. 20a.

**Fig. 7:**
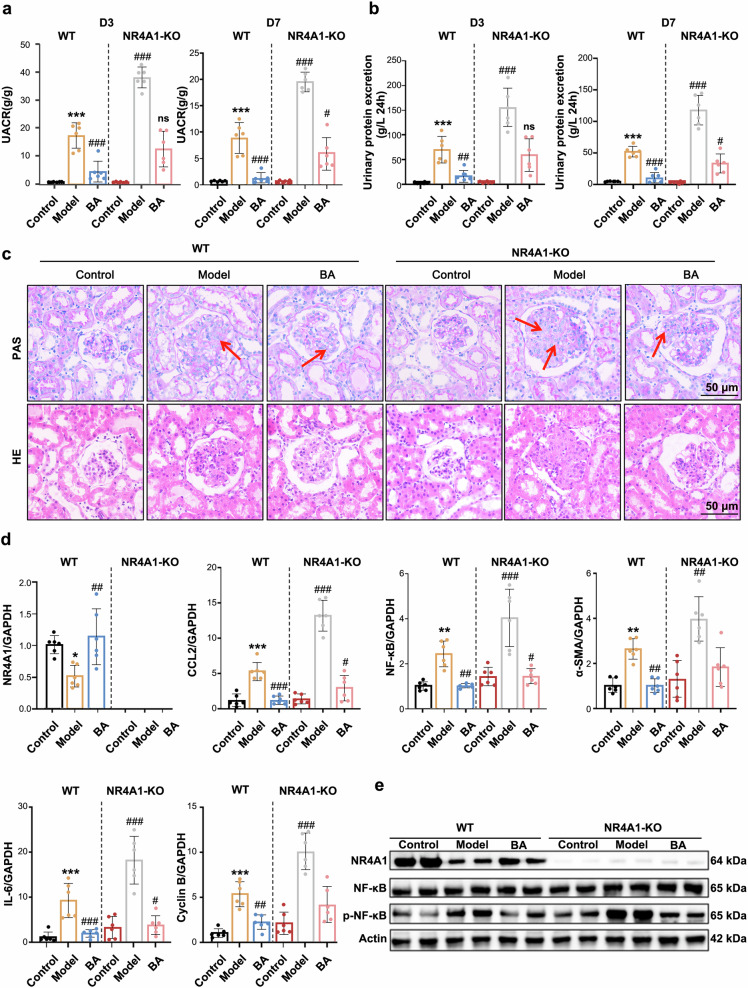
BA alleviates anti-Thy1 nephritis dependent on NR4A1. **a**, **b** Effects of NR4A1 deficiency on 24-hour urinary protein excretion and UACR. **c** Representative glomerular images from BA-treated or NR4A1-deficient rats with anti-Thy1 nephritis, stained with PAS and HE (scale bar = 50 µm; magnification ×400). **d** Impact of NR4A1 deficiency on the expression of NR4A1, IL-6, CCL2, NF-κB, α-SMA and Cyclin B. **e** Western blot analysis of NR4A1, NF-κB/p-NF-κB protein levels in each group. Data are expressed as mean ± SEM (*n* = 6). Statistical significance was determined as follows: *p < 0.05, **p < 0.01, ***p < 0.001 vs the control group; #*p* < 0.05, ##*p* < 0.01, ###*p* < 0.001 vs the model group

We observed that UACR and 24-hour urine protein levels were significantly elevated in NR4A1^−/−^ group compared to the model group. BA effectively ameliorated proteinuria in WT rats; however, its renoprotective effects were substantially diminished when NR4A1 was knockout (Fig. 7a, b). In WT rats, BA suppressed MCs proliferation and matrix deposition, thereby improving renal lesions. Strikingly, NR4A1 knockout exacerbated renal pathology, and the beneficial effects of BA were weakened in NR4A1^−/−^ group (Fig. 7c and supplementary Fig. 20b-d).

Notably, BA’s anti-inflammation, anti-fibrosis, and anti-proliferation effects were substantially diminished in NR4A1^−/−^ group, as evidenced by significantly attenuated suppression of both related genes and proteins (Fig. 7d and supplementary Fig. 20e–h). Furthermore, phosphorylated NF-κB protein expression was more significantly elevated in NR4A1^−/−^ rats (Fig. 7e), indicating that BA exerted renoprotective effects in a NR4A1-dependent manner.

### BA stabilizes NR4A1 by inhibiting K48-linked polyubiquitination at K558 to confer renal protection

**Fig. 8:**
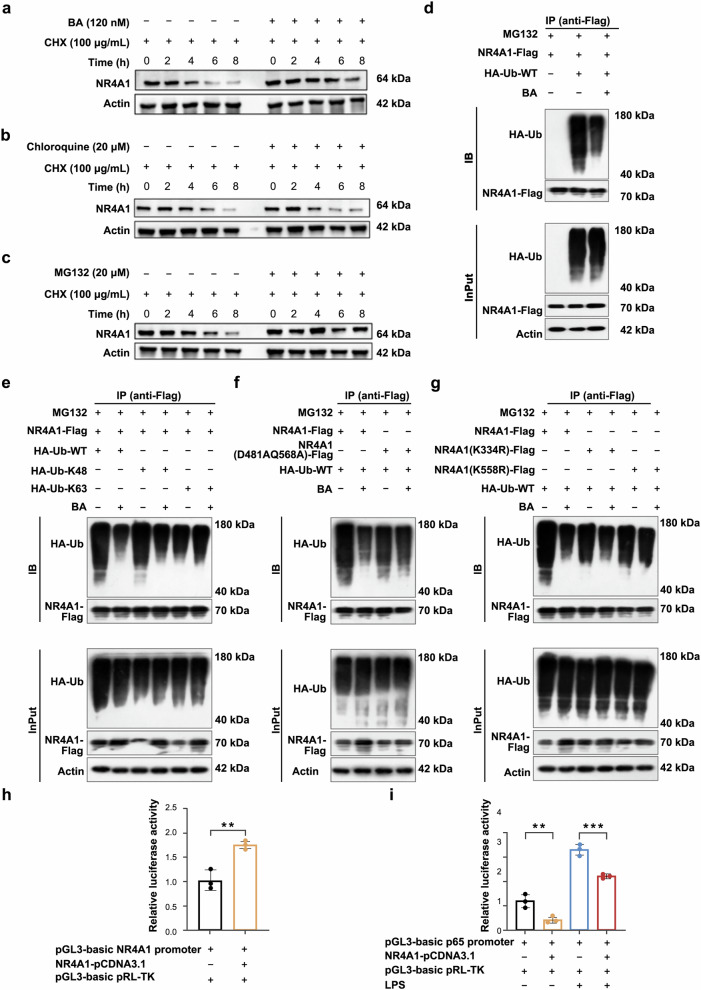
BA stabilizes NR4A1 by inhibiting K48-linked polyubiquitination at K558 to confer renal protection. **a** MCs were treated with CHX (100 μg/mL) for different times, western blot examined the protein expression of NR4A1. **b** MCs were treated with Chloroquine (20 μM) for different times, western blot examined the protein expression of NR4A1. **c** MCs were treated with MG132 (20 μM) for different times, western blot examined the protein expression of NR4A1. **d** Inhibition of NR4A1 ubiquitination expression by BA. **e** BA inhibits K48 ubiquitin chain modification. **f** BA fails to modulate NR4A1 ubiquitination upon mutation of residues D481 and Q568. **g** K558 serves as a critical ubiquitination site in NR4A1. **h** Dual-luciferase assays demonstrated NR4A1 binding to its autoregulatory promoter. **i** Dual-luciferase assays demonstrated NR4A1 inhibited the transcriptional activity of NF-κB

To decipher the precise molecular mechanism by which BA regulates NR4A1, we first performed cycloheximide (CHX) chase assays, which revealed that BA treatment significantly prolonged the half-life of NR4A1 (Fig. 8a), suggesting post-translational stabilization. Next, the lysosome inhibitor chloroquine had no impact (Fig. 8b), whereas the proteasome inhibitor MG132 mimicked the effect of BA (Fig. 8c), indicating that BA specifically suppresses ubiquitin-proteasome-dependent degradation of NR4A1.

To investigate the effect of BA on NR4A1 ubiquitination, we co-transfected flag-tagged NR4A1 and ubiquitin plasmids into HEK293T cells. Results showed that NR4A1 undergoes significant ubiquitination, and BA markedly suppressed NR4A1 polyubiquitination (Fig. 8d). Analysis of specific ubiquitin linkages demonstrated that BA selectively inhibited K48-linked polyubiquitination but not K63-linked polyubiquitination (Fig. 8e). Based on molecular docking results, we then transfected cells with D481A and Q568A mutant plasmids. Results revealed that BA’s anti-ubiquitination activity requires binding to the D481 and Q568 residues in the NR4A1 LBD, as mutations at these sites attenuated BA’s effects (Fig. 8f).

To predict NR4A1 ubiquitination sites, we used the GPS-Uber and PTMeXchange databases^16,17^ and bioinformatic analysis identified that K334 and K558 acted as the highest-scoring ubiquitination sites (supplementary Fig. 21a). Notably, K558 co-localizes with Q568 on the same α-helical structure, suggesting potential spatial coordination in BA-mediated regulation (supplementary Fig. 21b). To functionally validate these sites, we generated K334R and K558R point mutants. Results showed that while the K334R mutant remained responsive to BA treatment, the K558R mutation completely abolished BA’s inhibitory effect on NR4A1 ubiquitination, establishing K558 as the critical residue for BA-mediated stabilization (Fig. 8g). Complementary dual-luciferase assays revealed that accumulated NR4A1 autoregulates its own promoter activity while suppressing NF-κB transcriptional activity (Fig. 8h, i).

Thus, BA binds to the LBD of NR4A1 (*via* D481/Q568), thereby sterically blocking K48-linked polyubiquitination at K558 and stabilizing NR4A1 protein levels. This stabilization enables NR4A1 to auto-activate its promoter, amplifying the transcriptional repression of NF-κB, and ultimately attenuating inflammatory cascades and mesangial proliferation to confer renal protection.

## Discussion

NR4A1 is ubiquitously expressed across multiple organ systems, encompassing the nervous, endocrine, immune, and metabolic tissues.^18^ As a pleiotropic nuclear receptor, it orchestrates a wide array of cellular processes, including proliferation, apoptosis, differentiation, and inflammatory responses, through transcriptional regulation. Functioning as an early-response sensor, NR4A1 undergoes rapid conformational changes upon stimulation to modulate the expression of target genes.^19,20^ Emerging evidence indicates that dysregulation of NR4A1 is implicated in various pathologies, such as neurodegenerative disorders, malignancies, and inflammatory diseases.^21,22^ Specifically, NR4A1 can inhibit NF-κB activation by impairing the binding of p65 to DNA and reducing the expression of IκB, thereby restraining the inflammatory response of MCs.^9,23^ Additionally, inflammatory stimulation can induce the expression of NR4A1, while long-term stimulation may lead to its inhibition.^24^ In the context of renal pathophysiology, NR4A1 has been found to promote renal interstitial fibrosis by regulating the phosphorylation of p38 MAPK.^25,26^ Furthermore, the loss of NR4A1 in immune cells appears to result in increased kidney injury and reduced renal function in a hypertensive nephropathy rat model. These studies have highlighted the critical involvement of NR4A1 in renal pathophysiology, positioning it as a potential therapeutic target for kidney diseases.^27^

Consistent with these reports, our research also found that the expression of NR4A1 is low in patients with MsPGN and in the anti-Thy1 nephritis rat model. NR4A1 knockdown exacerbated MCs inflammation and proliferation, whereas its overexpression attenuated these pathological features. These results suggest that restoring NR4A1 protein levels could represent a viable therapeutic strategy for MsPGN.

BA, a triterpenoid with established efficacy in oncology and viral infections.^12,13^ Although BA has been reported to exert an anti-inflammatory effect in diabetic nephropathy, our findings confirmed that BA emerges as a groundbreaking therapeutic candidate for MsPGN through NR4A1. Pharmacological studies revealed that BA treatment significantly improved proteinuria and renal histopathology in anti-Thy1 nephritis rats. Notably, the therapeutic efficacy of BA was substantially diminished in NR4A1^−/−^ rats, establishing NR4A1 as the essential molecular target for BA’s renoprotective effects. Meanwhile, BA can directly bind to NR4A1 and enhance the NR4A1 protein expression level, these outcomes support that BA can be considered as the natural NR4A1 ligand by binding to NR4A1. To date, CsnB is the ligand that identified as the agonists for NR4A1.^10^ Unlike CsnB that activates NR4A1 transiently, BA’s stabilization strategy ensures sustained target engagement, avoiding receptor desensitization, a common limitation of nuclear receptor agonists. Thus, this stabilization of BA on NR4A1 protein degradation represents a more effective strategy for MsPGN treatment.

To further investigate how BA exerts its therapeutic effect by stabilizing NR4A1, we report for the first time that BA inhibits MsPGN progression through its targeted effect on suppressing NR4A1 protein degradation. Protein post-translational modifications (PTMs) are essential for maintaining protein homeostasis in cellular biological processes and disease development.^28^ These modifications include phosphorylation, ubiquitination, glycosylation, S-nitrosylation, and methylation, among others. Among them, ubiquitination has garnered increasing attention in numerous studies. Ubiquitin, an evolutionarily conserved 8.5 kDa protein (76 amino acids), mediates critical post-translational modifications through its seven lysine residues (K6, K11, K27, K29, K33, K48, K63) and N-terminal methionine (M1). These residues act as linkage sites for forming structurally distinct polyubiquitin chains, which determine functional outcomes. Ubiquitin modifications exhibit remarkable diversity: monoubiquitination regulates endocytosis, DNA repair, and inflammatory signaling; multiubiquitination directs membrane protein trafficking and signal transduction; and polyubiquitination (via specific lysine linkages) dictates substrate fate, with K48-linked chains predominantly targeting proteins for proteasomal degradation.^29,30^ Notably, within the emerging paradigm of targeted protein degradation (TPD), an innovative therapeutic strategy for precise molecular intervention, two major protein degradation systems: the ubiquitin-proteasome system (UPS) and the autophagy-lysosome pathway (ALP), coordinately regulate the degradation of disease-associated proteins.^31^ However, our results indicate that BA-mediated inhibition of NR4A1 degradation is associated with the ubiquitin-proteasome pathway. Thus, this study verifies the importance of BA in regulating NR4A1’s ubiquitin-mediated degradation during MsPGN progression and explores its underlying mechanism.

**Fig. 9:**
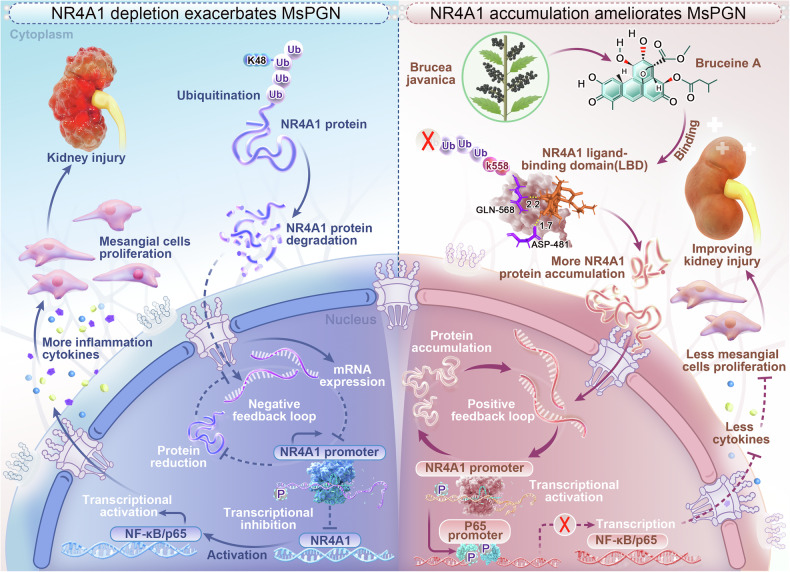
BA stabilizes NR4A1 by inhibiting K48-linked polyubiquitination at K558 to ameliorate MsPGN. (Left) Under pathological conditions, K48-linked polyubiquitination mediates NR4A1 degradation, thereby impairing its autoregulatory transcriptional activity. Subsequent NF-κB activation promotes inflammatory cytokine release and MCs proliferation, exacerbating kidney injury. (Right) BA inhibits K48-linked polyubiquitination at K558, stabilizing NR4A1. This restores NR4A1-mediated transcriptional auto-amplification and NF-κB suppression, attenuating inflammation and mesangial hyperplasia to confer renal protection

To dissect the molecular mechanism by which BA stabilizes NR4A1, we first confirmed through protein degradation assays that NR4A1 undergoes ubiquitin-dependent degradation. Ubiquitin linkage-specific analyses further revealed that BA selectively inhibits K48-linked polyubiquitination of NR4A1 (but not K63-linked), pinpointing its involvement in proteasomal degradation. To identify the structural basis of BA’s action, molecular docking and site-directed mutagenesis experiments demonstrated that BA binds to the LBD of NR4A1 at residues D481 and Q568. Mutating these sites (D481A/Q568A) attenuated BA’s ability to inhibit ubiquitination, confirming the dependence of BA’s effects on this specific binding interaction. We then pinpointed the critical ubiquitination site on NR4A1 through bioinformatic prediction and functional validation: the K558R mutation abolished BA-mediated suppression of ubiquitination, whereas the K334R mutation did not impair BA responsiveness, highlighting K558 as the key residue targeted by BA. These findings not only elucidate the precise molecular basis by which BA regulates NR4A1 stability but also reveal a novel mechanism for targeting ubiquitin-mediated degradation in MsPGN and related disorders (Fig. 9).

Although this study establishes the BA-regulated mechanism of NR4A1, several questions remain. First, while the K48 ubiquitination degradation pathway has been identified, the specific E3 ubiquitin ligases involved in this process remain unclear. Second, the subcellular dynamics of NR4A1, particularly its nucleocytoplasmic shuttling under BA treatment, warrant investigation via spatial proteomics.^32^ These limitations, however, do not diminish the present findings but rather map a roadmap for future studies.

In summary, we demonstrate that the natural compound BA confers renal protection by specifically inhibiting K48-linked ubiquitination at K558, thereby stabilizing NR4A1 and suppressing NF-κB-driven inflammation. Importantly, we establish a “protein stabilization” therapeutic paradigm that transcends conventional receptor activation strategies, addressing the limitations of classical NR4A1 agonists. These findings not only advance our understanding of nuclear receptor regulation in kidney disease but also provide a framework for targeting protein homeostasis in other pathological conditions, offering new insights for the development of novel drugs for kidney diseases.

## Materials and methods

### Human kidney specimens’ preparation

Renal tissues from seventeen patients with IgAN and matched controls were analyzed. Nine samples were profiled by single-cell RNA sequencing, and eight were assessed by immunohistochemistry to respectively verify the transcriptional and protein levels of key genes. All patients were recruited from the Second Affiliated Hospital of Guangzhou University of Chinese Medicine, in accordance with the ethical guidelines outlined in the Declaration of Helsinki. Prior approval was granted by the Ethics Committee of the Second Affiliated Hospital of Guangzhou University of Chinese Medicine; the ethical approval number was BE2023-047-01. Written informed consent has been obtained from all enrolled patients.

### Bioinformatic analysis of bulk RNA-seq and scRNA-seq data

We systematically integrated GEO datasets (GSE93798/GSE37460, 31 controls vs 47 patients). Inclusion criteria required matched healthy/IgAN groups ( ≥ 10 samples/group). After Limma/SVA-based normalization, we identified 14 DEGs ( | logFC | >1, *p* < 0.05) from the combined matrix. Functional enrichment and protein-protein interaction (PPI) network analyses (Cytoscape) were performed to identify hub genes. Raw scRNA-seq data from IgAN patients and controls were processed using Seurat (v4.0). After quality control and normalization, MCs were subset based on canonical markers. Differential expression of NR4A1 and related genes were analyzed using model-based analysis of single-cell transcriptomics (MAST), with adjusted *p* < 0.05 and |log2FC | >0.25 as thresholds. Cell-type specificity was validated *via* marker gene overlap. We further examined the correlation between glomerular filtration rate (GFR) and the hub genes using the Nephroseq v5 platform database.^33^

### Molecular docking

To identify candidate compounds that bind to NR4A1, we used the three-dimensional structures of NR4A1 and a scoring function that correctly ranks candidate dockings.^34^ Subsequently, molecular docking of the components with the targets was carried out using AutoDock Vina.^35^ The docking results were visualized and analyzed with PyMOL2.2.0 software. Molecular docking scores and binding energies were then calculated.

### Identification of BA-binding proteins with human proteome microarray

HuProt™ human proteome microarray was used for the identification of BA-binding proteins.^36–38^ 3% BSA solution was used to block the proteome microarray for 3 h at 37 °C. Subsequently, 10 μM Biotin-BA and Cy3-streptavidi were incubated separately for 1 h at 37 °C with free biotin serving as a control to prevent false-positive detections. Finally, LuxScan 10 K/A microarray scanner (CapitalBio, China) was used to detect signals, and GenePix Pro 6.0 software was employed for data analysis. Signal to noise ratio (SNR), which was defined as ratio of the median of foreground signal to the median of background signal, was calculated for each protein. The cutoff of BA’s positive proteins were set as fold change (FC) ≥ 3.^39^

### Surface plasma resonance (SPR) assay

SPR analysis (BIAcore 1 K, CM5 chip) quantified BA binding kinetics to wild-type/mutant NR4A1/LBD at 25 °C. Proteins were immobilized per manufacturer’s protocol, with BA (20 mM DMSO stock) serially diluted in PBS for injection. Association and dissociation constants were determined by globally fitting of the data to a 1:1 Langmuir binding model using BIAcore 1 K evaluation software (Cytiva, Marlborough, MA, USA). Then, the data were exported by Origin 7 software (v.7.0552, OriginLab) for the generation of the final figures.

### Microscale thermophoresis (MST) assay

MST was used to determine the interaction between BA and NR4A1. Proteins were labeled with RED-NHS- labeled proteins. Serial ligand dilution (16 gradients) was performed in 50 mM HEPES (pH 7.4) containing 0.05% Tween, and the mixtures were incubated at room temperature for 20 min. The resulting mixture was loaded into a Monolith NT.115 Capillary, and the thermophoretic signal was measured using the Monolith NT.115 (Nano Temper) according to the manufacturer’s standard protocol. Changes in Kd values were analyzed using the Nano Temper analysis software.

### Drug affinity responsive target stability (DARTS) assay

DARTS assay was conducted as previously described.^40^ In brief, cells were lysed using a HEPES solution containing protease and phosphatase inhibitors, and then treated with low and high concentrations of BA or DMSO as a control. After incubation at 37 °C for 30 min, pronase (1 μg/mL) was diluted to 0.05 μg/mL and then added to the protein-small molecule mixture, followed by a further 30 min incubation. The reactions were stopped by adding loading buffer and subsequently subjected to western blot analysis.

### Cellular thermal shift assay (CETSA)

CETSA was employed to assess target engagement by monitoring ligand-induced changes in protein thermal stability. MCs were treated with 120 nM BA or DMSO (control) for 6 h, followed by resuspension and centrifugation. Cell aliquots were subjected to thermal denaturation at 52, 57, 62, 67, 72, and 77 °C for 3 min, then lysed through three freeze-thaw cycles in liquid nitrogen. Soluble proteins were isolated by centrifugation (13,000 × g, 20 min) and analyzed *via* western blot.

### Overexpression plasmid construction and siRNA transfection

The overexpression plasmid of NR4A1 and negative control vectors (Yunzhou Biotechnology Co., Ltd., Guangzhou, China) were transfected into MCs for 48 h using Lipofectamine 3000 according to the instruction manual in vitro. NR4A1-specific siRNA (sense:5’-ACACUGCCAAGUUGGACUAUU-3’) and negative controls were transfected at 50 nM using RNA TransMate. NR4A1 coding sequence was subcloned into the WPRE3-containing AAV transfer plasmid under the CMV promoter, and the adeno-associated virus serotype 9 (AAV9) viral particles were subsequently packaged using the AAV9 capsid by Guangdong Keguanda Pharmaceutical Technology Co., Ltd. Renal vein delivery of AAV9-NR4A1 (200 μl, 5 × 10¹¹ vg/mL) was performed in anesthetized Wister rats using 31-gauge needles following flank incision.

### Protein stability assay

The half-life of NR4A1 was determined *via* cycloheximide (CHX, 100 μg/mL) chase assay at indicated time points (0, 2, 4, and 8 h). To assess degradation pathways, cells were pretreated with either the proteasome inhibitor MG132 (20 μM) or the lysosome inhibitor chloroquine (CQ, 20 μM) for 6 h prior to CHX exposure.

### Ubiquitination assay and Co-immunoprecipitation (Co-IP)

Plasmids with flag-tagged constructs, including wild-type (NR4A1), NR4A1(D481A, Q568A), NR4A1(K334R, K558R), along with the HA-tagged ubiquitin plasmid (HA-Ub-WT, HA-Ub-K48 and HA-Ub-K63) were transfected into HEK293T cells using transfection reagent. After 24 h, the cells were treated with 120 nM BA or DMSO for 24 h, followed by 20 μM MG132 for 6 h to stabilize ubiquitinated proteins. For Flag-Co-IP, cell lysates (1 mg total protein) were incubated overnight at 4 °C with Flag antibody-conjugated magnetic beads. The immunoprecipitants were analyzed by western blot, with input lysates serving as transfection controls.

### Dual-luciferase reporter gene assay

To investigate NR4A1’s transcriptional regulation, promoter fragments containing either the NR4A1 native promoter or NF-κB response elements were cloned into the pGL3-basic luciferase vector. MCs were co-transfected with each promoter construct (1 μg) and Renilla luciferase control vector (pRL-TK, 0.3 μg) using Lipofectamine 3000. After 24 h incubation, luciferase activity was quantified using the Dual-Luciferase Reporter Assay System (Promega) according to the manufacturer’s protocol. Firefly luciferase signals were normalized to Renilla values for data analysis.

### Animal experimental design

Thirty-six male Wistar rats (SPF, 160–180 g) were maintained under standard conditions (Approval No. 202295) with ethical approval from Guangdong Provincial Hospital of Traditional Chinese Medicine under comfortable conditions with regular feeding. Rats were randomly divided into six groups (*n* = 6 per group): (1) Control, (2) Model, (3) Model + BA (0.5 mg/kg), (4) Model + BA (1 mg/kg), (5) Model + BA (1.5 mg/kg), and Model + Olmesartan (10 mg/kg). BA was administered once every other day *via* intraperitoneal injection, and Olmesartan was administered daily by gavage for a week.

The NR4A1’s agonist (CsnB) was used as a positive control to assess BA’s effects on NR4A1, rats were randomly divided into six groups (*n* = 6 per group): (1) Control, (2) Model, (3) Model + 1.5 mg/kg BA, (4) Model + 10 mg/kg CsnB, (5) Model + BA + CsnB. Anti-Thy1 nephritis model was induced *via* tail vein injection of 2.5 mg/kg monoclonal anti-Thy1 antibody (mAb) as previously described.^41–44^ Control animals received equivalent volumes of sterile saline. Urine samples were collected on days 3 and 7 post-induction, and all the animals were sacrificed on the 7th day.

To evaluate the function of NR4A1 overexpression in vivo, renal vein injections of adeno-associated virus serotype 9 (AAV9) carrying NR4A1 (AAV9-NR4A1, 5 × 10^11^viral genomes/mL) or empty vector control were administered using a 31-gauge needle. Eighteen age-matched rats were randomly allocated into three groups (*n* = 6/group): (1) Vector control, (2) Model, (3) AAV9-NR4A1 intervention. The construction of animal models and the analytical methods employed followed established methodologies.

To evaluate the function of NR4A1 knockout in vivo*,* an NR4A1 knockout rat model (Wistar strain) was generated by Cyagen Biosciences Inc. (Guangzhou, China) using CRISPR/Cas9-mediated genome engineering technology. Briefly, 7 Exon 2 to exon 7 will be selected as target site (sequences shown on supplementary Table 1). Two pairs of gRNAs targeting vectors will be constructed and confirmed by sequencing. Ribonucleoprotein (RNP) will be co-injected into fertilized eggs for KO rat production.

Thirty-six male Wistar rats (SPF, 160-180 g, including 18 NR4A1^−/−^ and 18 WT rats) were maintained under standard conditions, rats were randomly allocated into six groups: (WT control, WT + disease model, WT + model + BA(1.5 mg/kg) treatment, NR4A1^−/−^ control, NR4A1^−/−^ + disease model, NR4A1^−/−^ + model + BA (1.5 mg/kg) treatment, *n* = 6/group). Animal modeling and drug administration were performed as described previously.

## Supplementary information

Supplementary Materials for Bruceine A protects nuclear receptor 4A1 from ubiquitin-degradation to alleviate mesangial proliferative glomerulonephritis

WB figures

Table 1 and Table 2

Table 3

Table 4

Table 5

Table 6

## Data Availability

The data reported here had been supplied to the GEO database. We shared the transcriptomic data in the GEO public database, the number is GSE263421 which provides access to our data. All data in this article are available upon reasonable request from the corresponding authors.
