# Supplementary material for: Bruceine A protects nuclear receptor 4A1 from ubiquitin-degradation to alleviate mesangial proliferative glomerulonephritis
Source: Signal Transduct Target Ther. 2025 Dec 5;10:397. doi: 10.1038/s41392-025-02495-2 (PMC12678413; doi:10.1038/s41392-025-02495-2)
Supplement: Supplementary file 1 — Supplementary Materials for Bruceine A protects nuclear receptor 4A1 from ubiquitin-degradation to alleviate mesangial proliferative glomerulonephritis [file 41392_2025_2495_MOESM1_ESM.docx]

**Supplementary Materials for**

**Bruceine A protects nuclear receptor 4A1 from ubiquitin-degradation to alleviate mesangial proliferative glomerulonephritis**

Huating Hu^1,2#^, Runze Li^1^, Kancheng He^3^, Lingling Wu^4^, Rongrong Li^1^, Jiayan Lu^1^ Ruimin Tian^1^, Chuanghai Zhang^1^, Jiayan He^1^, Yulian Chen^1^, Ruogu Lai^1^, Jiaqi Zhang^1^, Jiaqi Wu^1^, Ying Zheng^4^, Jinlian He^1^, Liang Liu^1*^, Xiangmei Chen^4*^, Hudan Pan^1*^

Correspondence to:

Hudan Pan ([hdpan@gzucm.edu.cn](mailto:hdpan@gzucm.edu.cn)); Xiangmei Chen ([xmchen301@126.com](mailto:xmchen301@126.com)); Liang Liu (lliu@gzucm.edu.cn)

**This PDF file includes:**

Materials and Methods

Supplementary Figures. S1 to S21

Supplementary Tables. 1 to 6

**Supplementary materials and methods**

**Chemicals and reagents**

All reagents and antibodies were obtained from commercial sources or collaborators as specified: Chemical compounds: BA (>98%, Nanjing Chunqiu), Cytosporone B (CsnB) (>98%, Source Leaf), Olmesartan(OM) (>99.91%, MCE), MG132 (MCE, HY-13259), Chloroquine (MCE, HY-17589A).Transfection reagents: Lipofectamine 3000 (Invitrogen, USA), RNA/siRNA transfection reagents (Shenggong, China).Antibodies: β-actin (#4970), α-SMA (#19245s), NF-κB (#8242), p-NF-κB (#3033) were obtained from CST. Abcam: MCP1 (#ab7202), fibronectin (#ab268020), NR4A1(#ab317395), Flag (#20543-1-AP), HA (#51064-2-AP), NR4A1 constructs (WT and mutants: D481A&Q568A, K334R, K558R) in pGC3.1-Flag backbone. Ubiquitin plasmids (HA-Ub-WT, HA-Ub-K48, HA-Ub-K63) and Flag-CoIP Kit (GeneCloudBio). Anti-Thy1 antibody (gift from Academician Xiangmei Chen’s team).

**Bilayer interferometry (BLI) assay**

To analyze the role of BA in the intermolecular interaction with NR4A1 protein, the Octet BLI system was employed. The NR4A1 protein was immobilized onto Super Streptavidin (NTA) biosensors. Five diluted solutions of BA, with concentrations of 6.25, 12.5, 25, 50 and 100 μM were prepared in PBS. The binding kinetics, including the Kd (equilibrium dissociation constant), Kon (association rate constant), Kdis (dissociation rate constant) and R2 values were reported. Kd was calculated by dividing the Kdis value by the kon value as previously described^1^.

**Renal histopathology and immunostaining**

Renal tissues were fixed in 4% paraformaldehyde for 48 h, sequentially dehydrated through graded ethanol, cleared in xylene, and paraffin-embedded. Coronal sections (3μm) were stained with periodic acid-Schiff (PAS) and hematoxylin-eosin (HE) for histopathological evaluation using an Olympus DP72 optical microscope. Glomerular hypercellularity was quantified *via* cellSens Standard software (Nikon ECLIPSE) as per published protocols^2^. Pathological scoring (5-point semi-quantitative scale) was independently performed by two board-certified pathologists blinded to experimental groups, with inter-rater reliability >90%^3^.

**Immunohistochemistry (IHC) and immunocytochemistry (ICC)**

Paraffin sections were dewaxed in xylene, rehydrated through an ethanol gradient, and subjected to antigen retrieval using EDTA buffer (BOSTER, AR0023). Endogenous peroxidase was quenched with 0.3% hydrogen peroxide (10 min), followed by blocking with 5% BSA (37°C, 1 h). Sections were incubated with primary antibodies :α-SMA (1:320), NR4A1 (1:100), FN (1:1000), and PCNA (1:200), and the signals were developed with DAB (MXB, D-AB2031).For immunofluorescence staining, frozen kidney sections were permeabilized with 0.5% Triton X-100(20 min), blocked (5% BSA, 1h), and incubated with an MCP1 antibody (1:200, 4°C overnight). After washing with PBS, sections were stained with fluorescent secondary antibodies and DAPI, and then imaged *via* confocal microscopy. Semi-quantitative analysis was performed as previously described^4,5^.

**Real-time PCR (qRT-PCR)**

To assess the genetic changes after administration, TRIzol reagent (Invitrogen, USA) was used to extract total RNA from renal tissues or cells. The total RNA was generated cDNA by reverse transcription kit (Trans Script, Beijing). Then, we used cDNA as the template, primers for amplification, and reverse transcriptase to form the reaction system, the cDNA was amplified by repeating the process of denaturation-annealing-extension. Finally, using the comparative 2 ^−ΔΔCT^ to calculate the relative transcriptional level of target genes, the primer sequences were provided in Supplementary table 2.

**Cell proliferation and cytotoxicity assessment assay**

MCs were seeded at 5×10^3^/well in 96-well plates and exposed to BA (0–2000 nM) for 24 h. Viability was determined *via* CCK-8 assay (Dojindo Laboratories), with absorbance measured at 450 nm using a microplate reader (BioTek Synergy H1). Half-maximal inhibitory concentration (IC_50_) was calculated by nonlinear regression analysis. LPS and PDGF-BB-stimulated cells were co-treated with BA (30–120nM) or OM (50 μM, positive control) for 24 h. Cell proliferation was quantified *via* CCK8 after 1 h substrate incubation. All experiments included triplicate technical replicates and were independently repeated three times.

**Colony formation assay**

Plate cloning assay was used to examine the restrain of BA on the colony formation ability of MCs.500 cells were added to 6-well plates and using 100ng/ml LPS and 50ng/ml PDGF-BB to induce MCs proliferation and inflammation model, after the cells adhered to the wall, different concentrations of BA (30,60,120 nM) and 50 μM OM were used. Change the medium every three days, after 10 days of treatment, cells were incubated with 0.1% crystal violet for 30 min after fixed by methyl alcohol. Then gently rinse the unstained crystal violet with running water. Images were taken using Nikon camera, and the clonal area was calculated using image J^6^. All the above steps were repeated 3 times individually and then statistically analyzed.

**Edu incorporation assay**

Cell-Light TM Edu Kit (Ribobio, Guangzhou, China) for assessing the suppression of BA on cell proliferation. In brief, 10000 cells/well were seeded on 12-well plates. Synchronize cells were synchronized in serum-free DMEM medium for 24 hours, The modeling method and BA dosage were the same as before. Then, the cells were incubated with complete DMEM containing 50 μM 5-Ethynyl-2, -deoxyuridine (Edu) for 2 h at 37℃ incubators, then, cells were fixed with methyl alcohol for 15 min after washing with PBS, and stained with Apollo for 30 min. Finally, stained with Hoechst for 30 min at normal temperature in the dark. 3 fields of view were randomly photographed using a fluorescence microscope (Nikon, ECLIPSE, Japan), and the number of cells was counted using Image J and analyzed with statistical software^7^.

**Cell cycle analysis**

Propidium iodide (PI) staining was used to assess cell cycle distributions, 1 ×10^5^ cells/well were seeded in 6-well plates, the modeling method and BA dosage were the same as before. Then, pre-cooling 75% ethanol fixed the cells at 4°C for suitable time and centrifuged the cells. Next, add 5 ml PBS to wash the cells, and centrifuge for 5 minutes. 0.5ml PI staining solution was joined with each tube, and incubated at 37ºC for 30 minutes. Flow cytometer (Novo Quanteon, USA) was measured to cell cycle distribution and results were analyzed by ModFit LT 2.0 software (USA)^8^.

**Western blotting**

RIPA mixture including protease and phosphatase inhibitors was used to extract total protein from rat kidney tissue and cells. The total protein was separated by 10% sodium dodecyl sulfate-polyacrylamide gel electrophoresis (SDS-PAGE), then, proteins were immediately transmitted to polyvinylidene difluoride (PVDF) membranes (Millipore, USA) by tank transfer method (Bio-Rad, USA). The membrane was blocked by 5% BSA for 2 h at room temperature and then incubated diluted primary antibodies against NR4A1, NF-κB, p-NF-κB, β-actin overnight at 4°C, the protein bands were incubated with dilute coupled secondary antibodies for 1 h and visualized using an ECL chemiluminescence solution (Merck Millipore, USA) with the Azure Imaging System (USA).

**Statistical analysis**

Data analysis was performed using Prism 8.2. Continuous variables were expressed as mean±SEM or mean±SD.The intergroup differences were assessed using an unpaired Student’s t-test for two-group comparisons or a one-way ANOVA with Tukey’s post hoc test for multi-group comparisons. Statistical significance was defined as *p* < 0.05.

**Supplementary Figures**

**
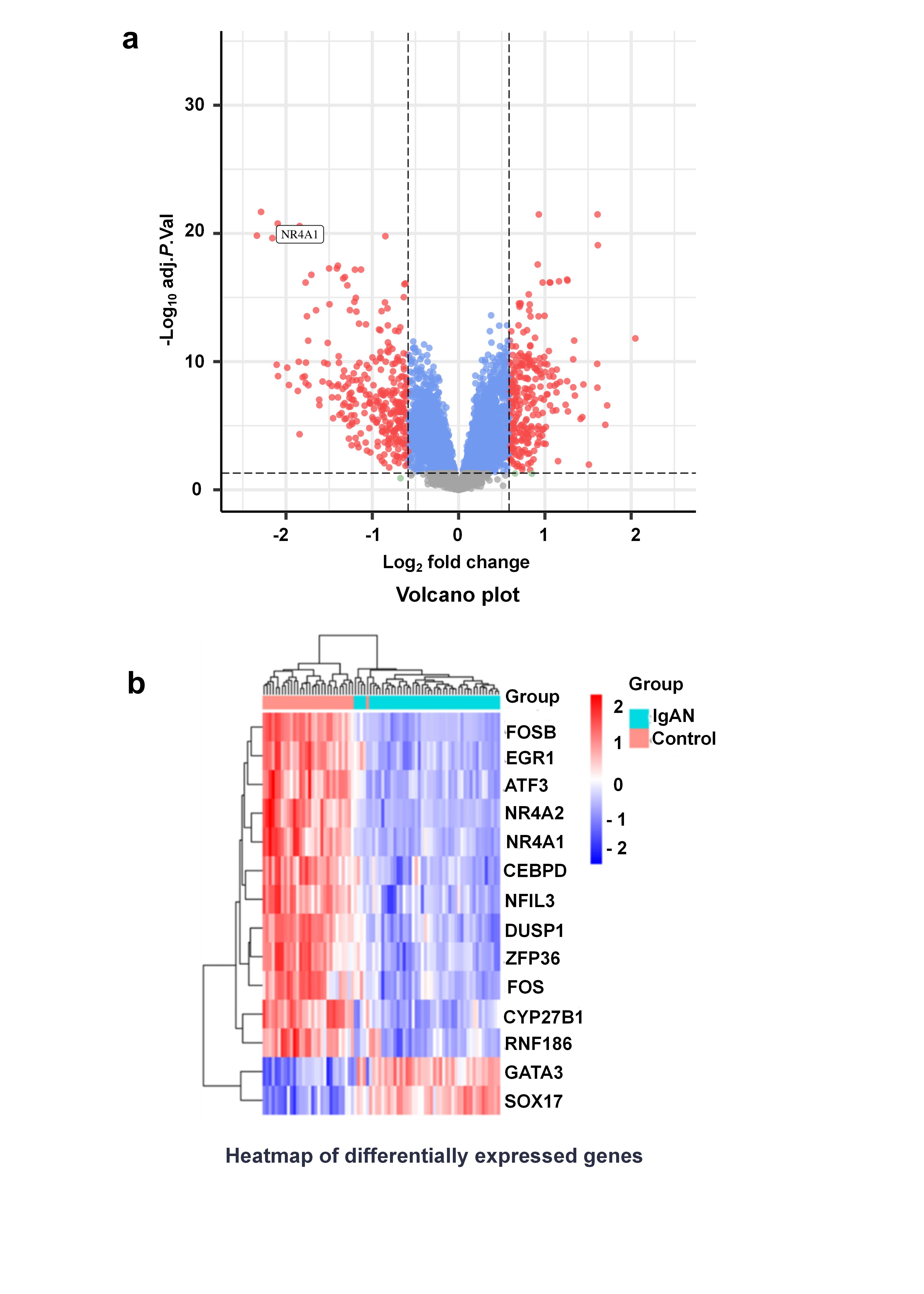
**

**Figure. S1** **The Volcano plot and heatmap of DEGs and hub genes between the IgAN patients and controls.**

**(a)** Volcano plot of DEGs between the IgAN and control groups. **(b)** Heatmap of hub genes between the IgAN and control groups.


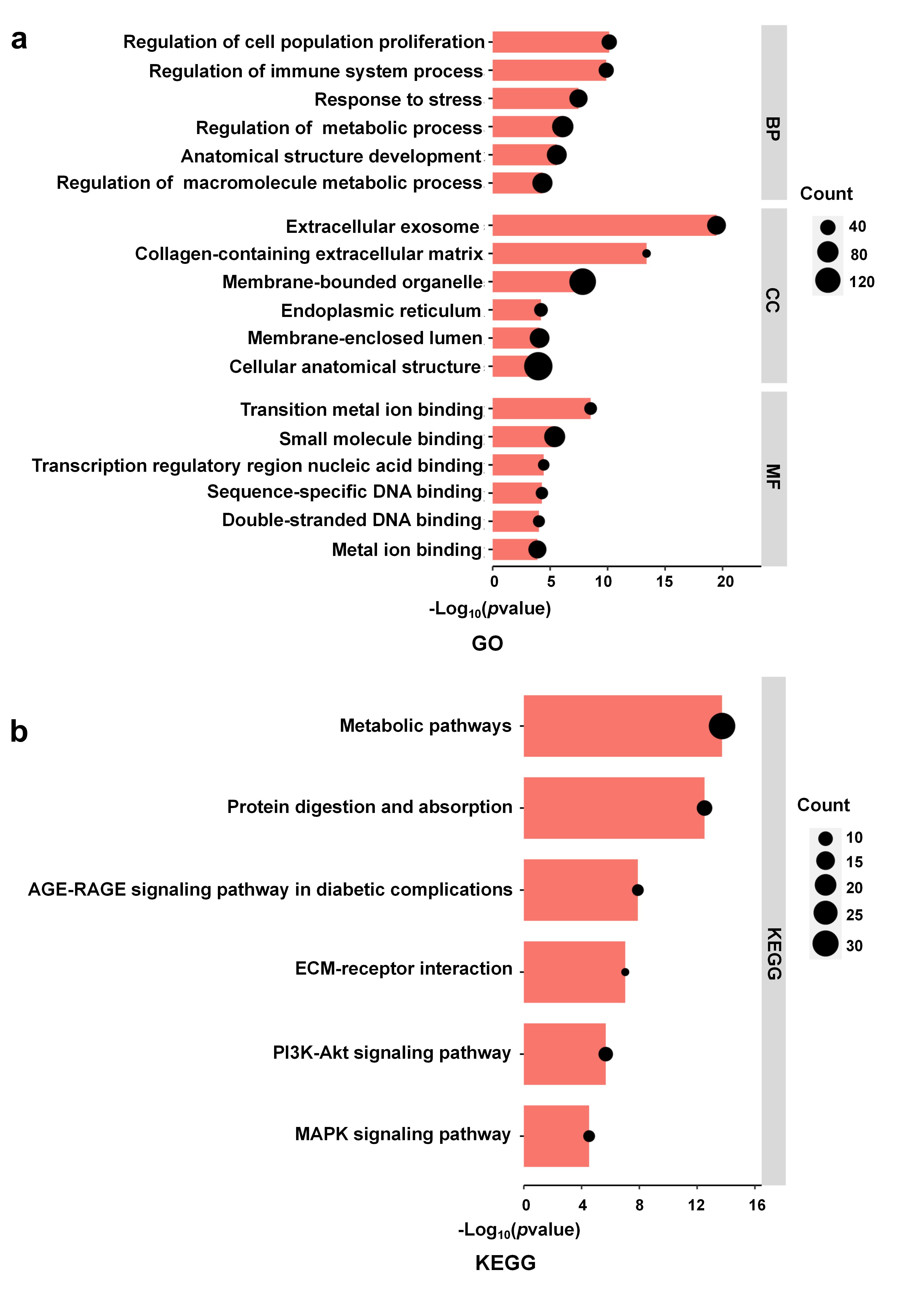
**Figure. S2** **DEGs between IgAN and controls are analyzed by GO and KEGG enrichment analysis.**

**(a-b)** Functional enrichment analysis of DEGs.


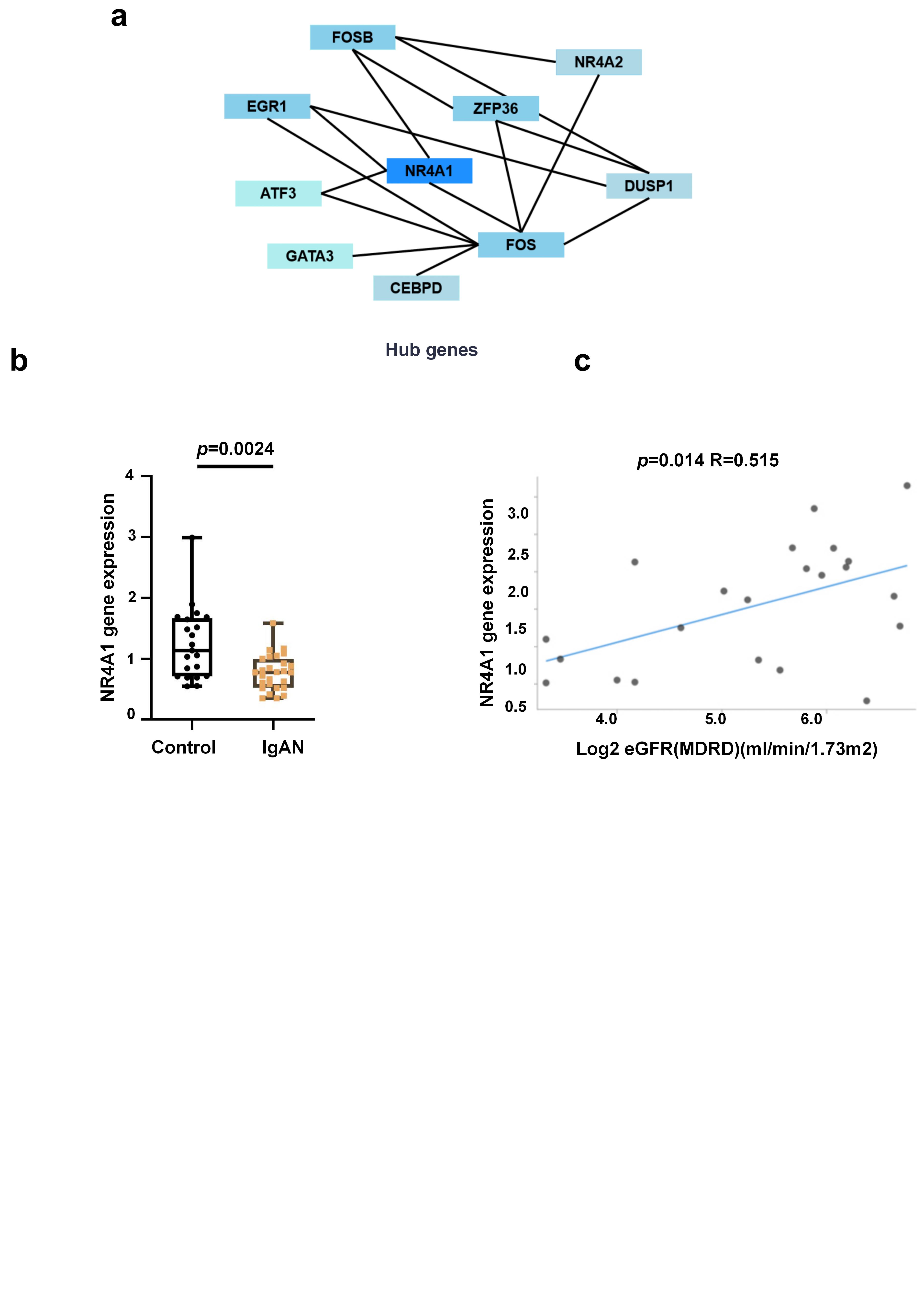


**Figure. S3 Discovery and verification of hub genes.**

**(a)** Hub genes are identified by using Cytoscape. **(b)** The Nephroseq V5 database shows NR4A1 expression between the renal tissue of IgAN patients and controls. **(c)** The Nephroseq V5 database demonstrates the correlation between the NR4A1 expression and estimates glomerular filtration rate (eGFR).


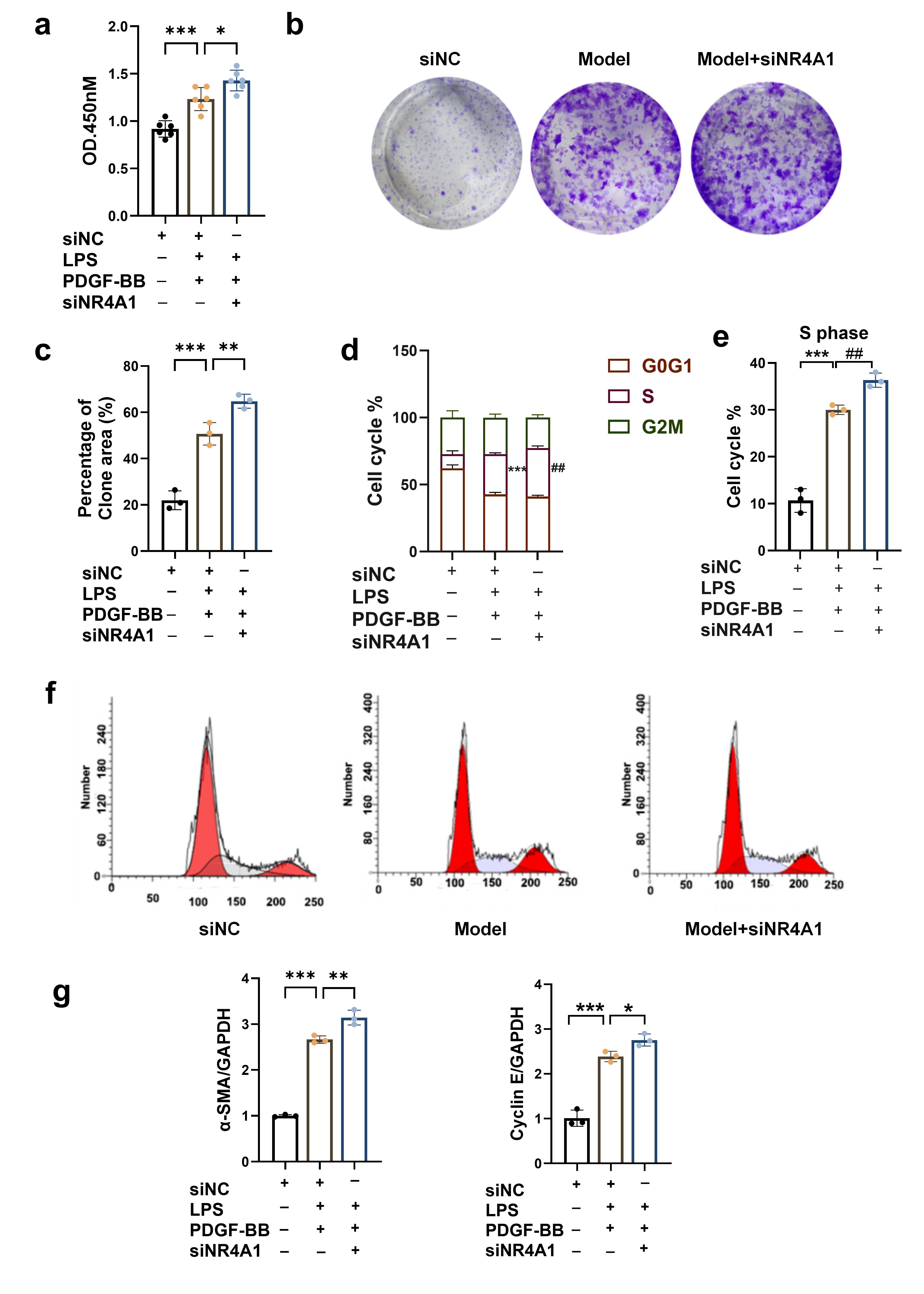
**Figure. S4 Knockdown of NR4A1 markedly enhances inflammatory responses and proliferation of MCs *in vitro*.**

1. CCK-8 assay detects the proliferation of MCs after NR4A1 knockdown. **(b-c)** Colony formation of MCs is assessed following NR4A1 knockdown. **(d-f)** Flow cytometry analysis of cell cycle distribution in MCs following NR4A1 knockdown. **(g)**  Expression of Cyclin E and α-SMA in MCs with NR4A1 knockdown.


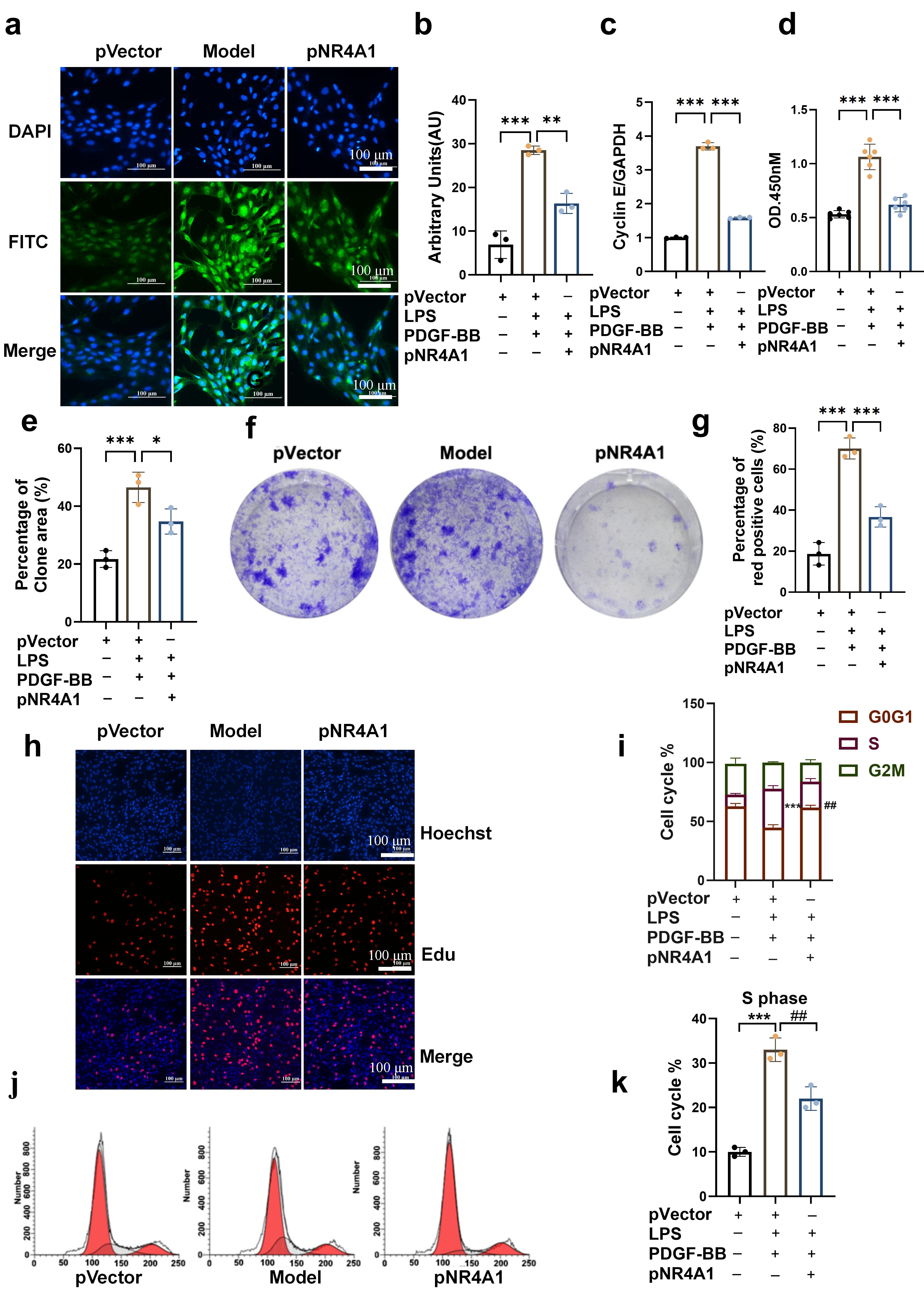


**Figure. S5 NR4A1 overexpression markedly alleviates inflammatory responses and proliferation of MCs *in vitro*.**

**(a-b)** Immunofluorescence analysis demonstrates that NR4A1 overexpression reduce CCL2 protein expression. **(c)** Effect of NR4A1 overexpression on Cyclin E expression. **(d)** Proliferation of MCs with NR4A1 overexpression detected by CCK-8 assay. **(e-f)** Colony formation of MCs with NR4A1 overexpression. **(g-h)** Effect of NR4A1 overexpression on MCs proliferation as determined by Edu incorporation assay. **(i-k)** Effects of NR4A1 overexpression on the cell cycle distribution of MCs by using Flow cytometry analysis.


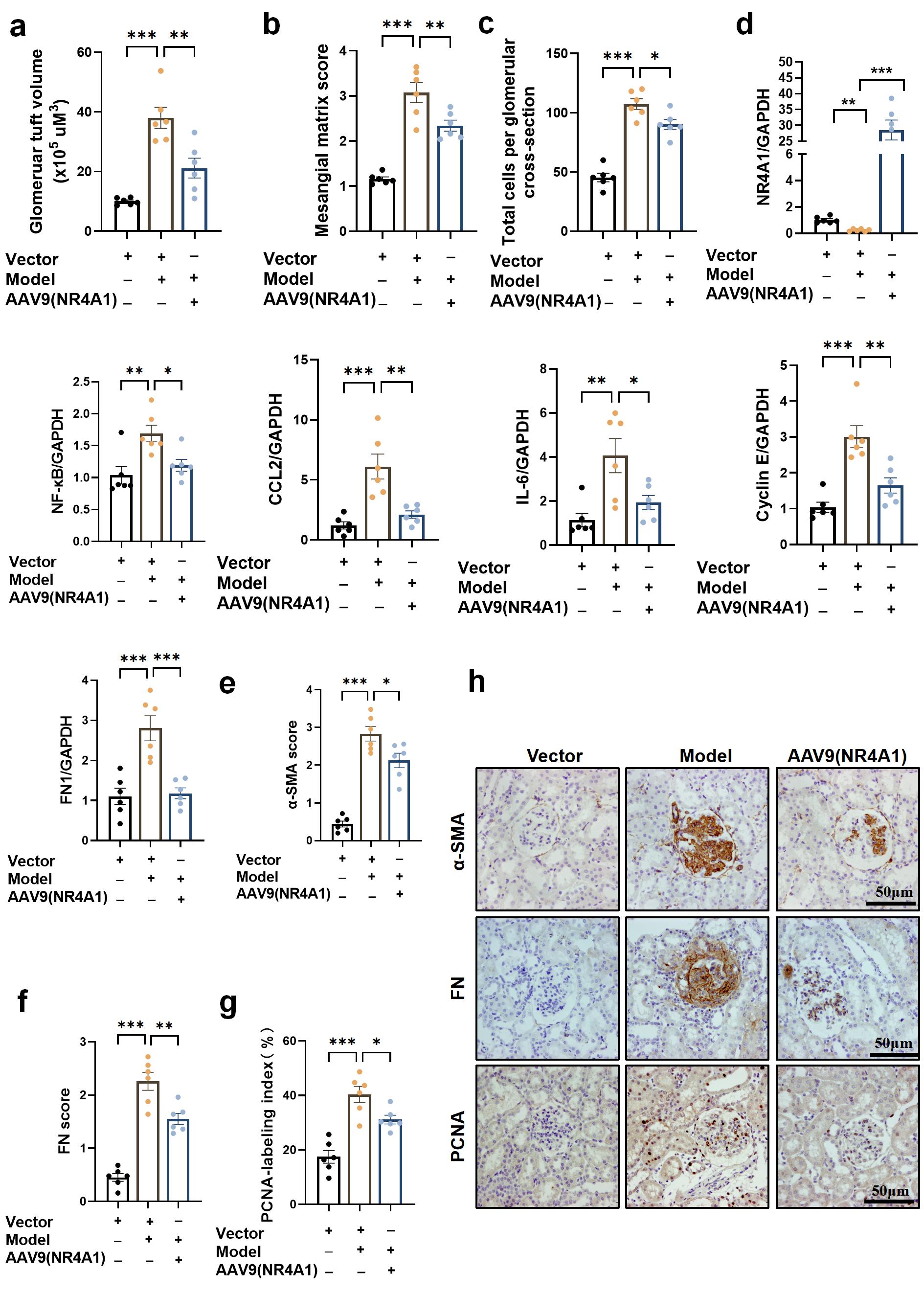
**Figure. S6 NR4A1 overexpression alleviates disease-related phenotypes *in vivo*.**

**(a-c)** Semi-quantitative analysis of renal pathological staining. **(d)** Effects of NR4A1 overexpression on the genes related to inflammation, proliferation and fibrosis. **(e-g)** Semi-quantitative scoring of immunohistochemical staining. **(h)** Effects of NR4A1 overexpression on the protein expression of PCNA, FN, and α-SMA in the kidneys.


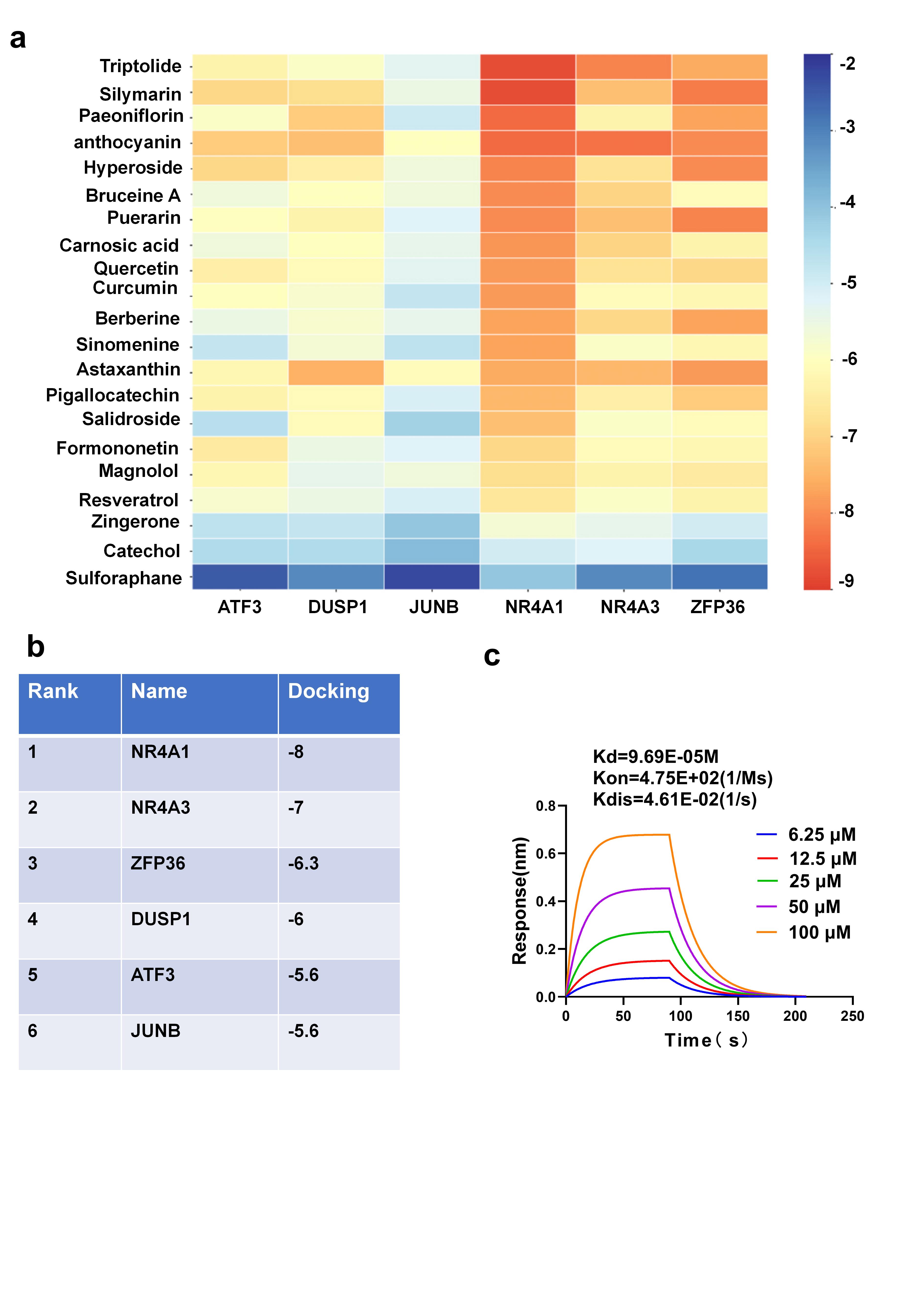


**Figure. S7 Molecular docking indicates that BA has a high affinity with NR4A1.**

**(a)** Docking scores of DEGs and compounds. **(b)** Docking score of DEGs and BA. **(c)** BLI assay is used to detect the affinity between BA and NR4A1.


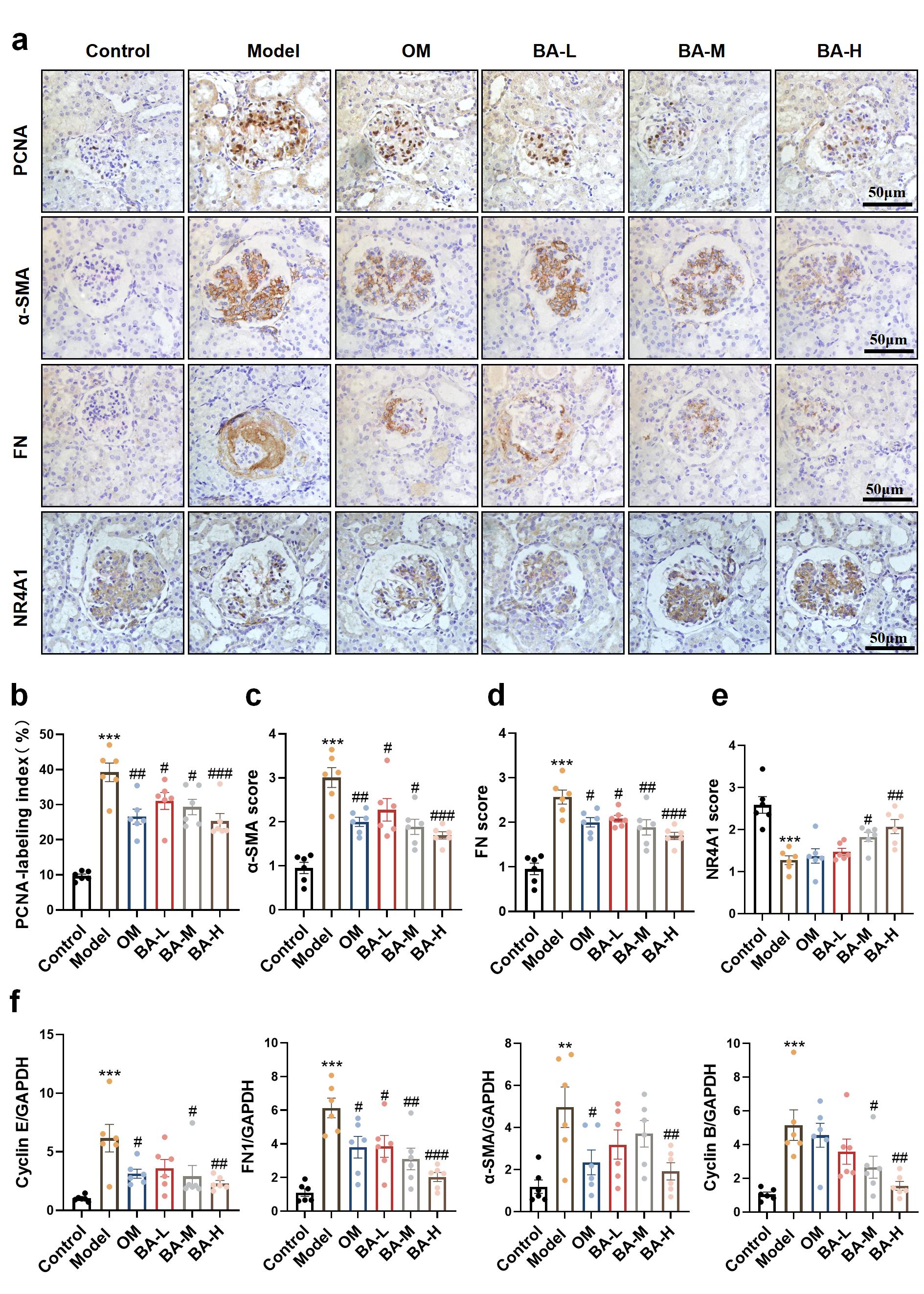
**Figure. S8 BA attenuates the proliferation and extracellular matrix deposition of MCs.**

**(a)** Protein expression of PCNA, α-SMA, FN and NR4A1 in renal tissues of the anti-Thy1 nephritis models. **(b-e)** Semi-quantitative analysis of FN, α-SMA, PCNA and NR4A1 protein expression in renal tissues of anti-Thy1 nephritis rats. **(f)** Effects of BA on Cyclin B, Cyclin E, α-SMA and FN gene expression.


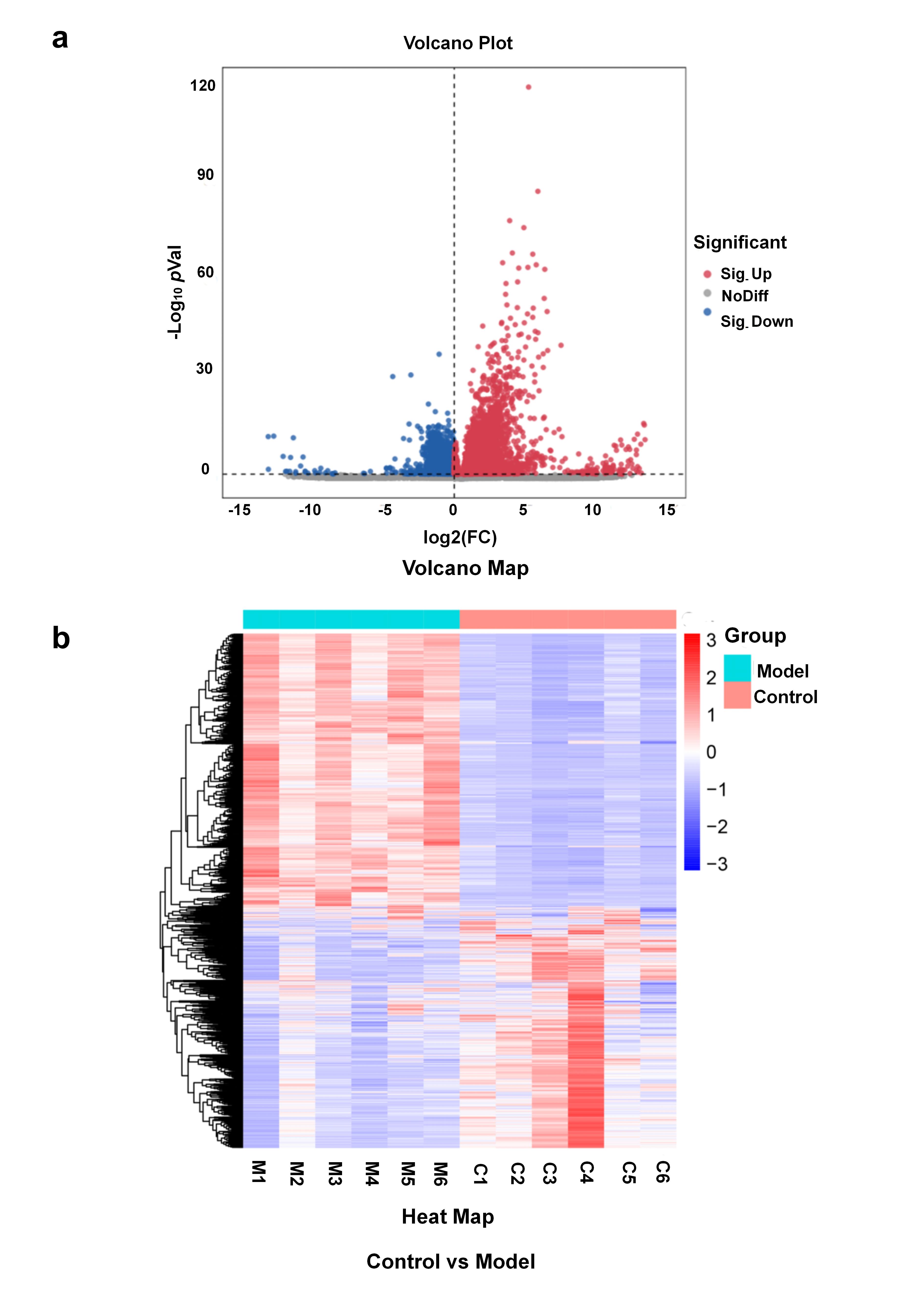
**Figure. S9 Volcano plot and heatmap analysis of DEGs between the control and model groups.**

**(a)** Volcano plot of DEGs. **(b)** Heatmap of DEGs.


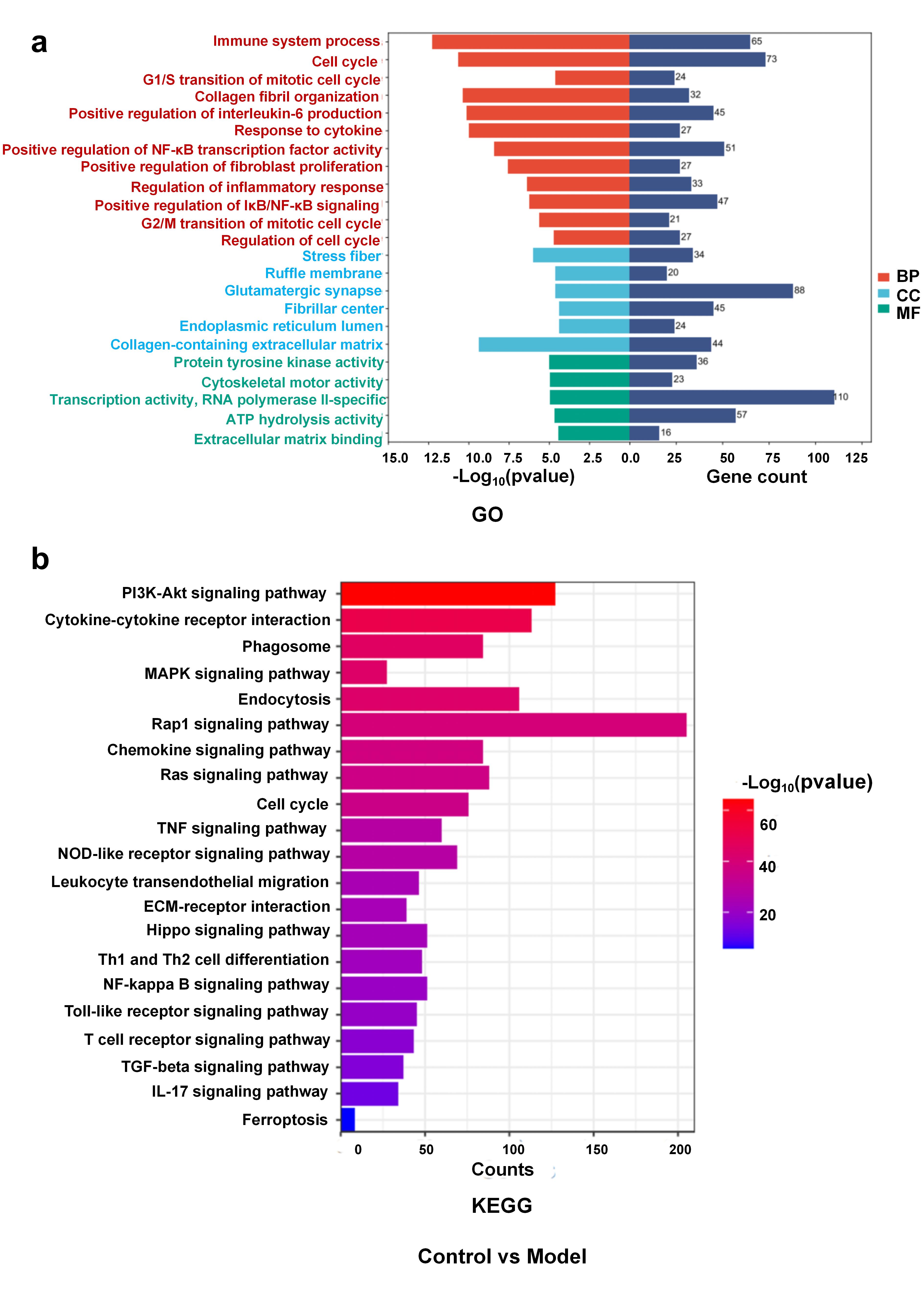


**Figure. S10 GO and KEGG enrichment analysis of DEGs between control and model groups.**

**(a–b)** DEGs between control and model groups are analyzed by using GO and KEGG.


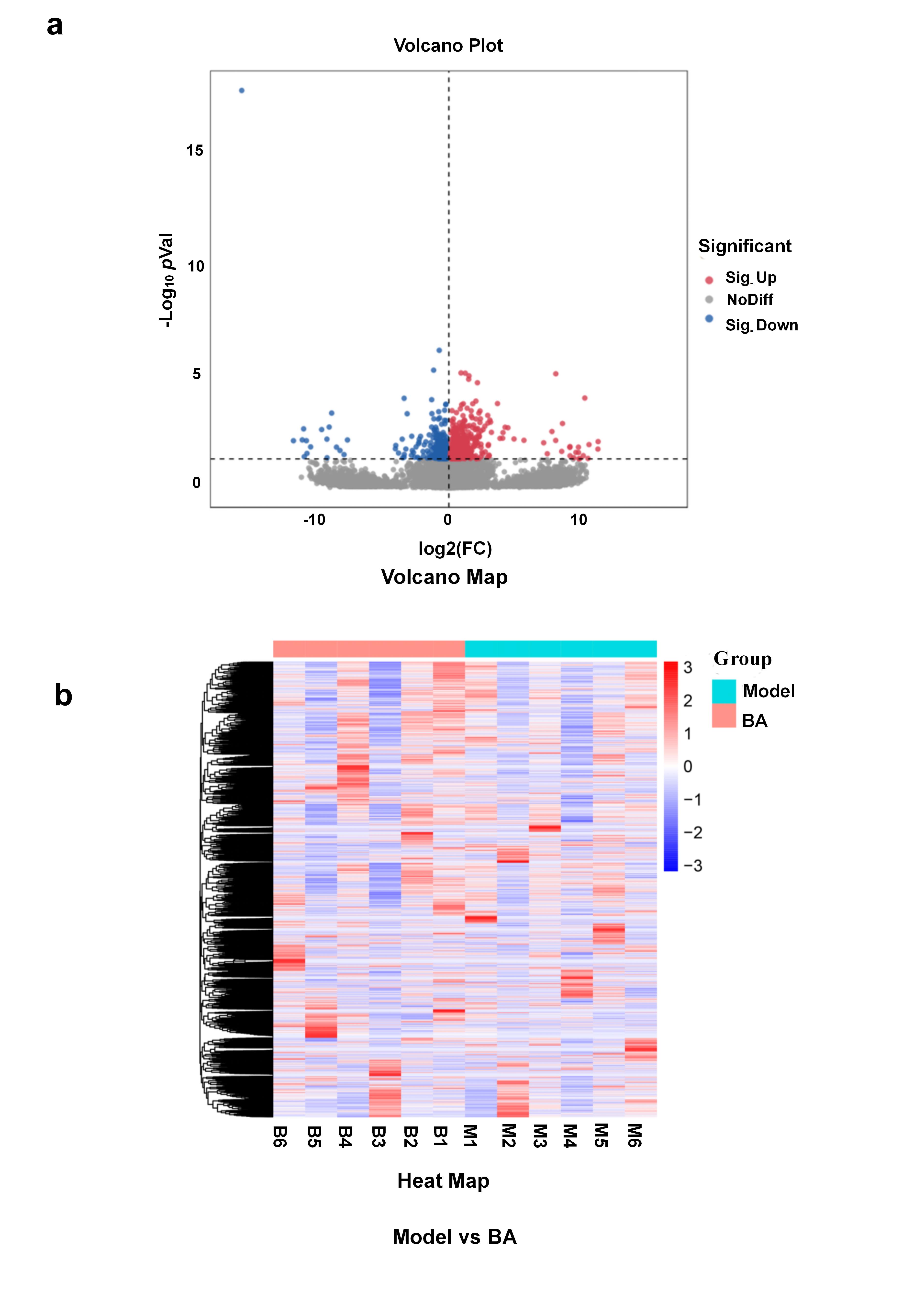


**Figure. S11** **Volcano plot and heatmap analysis of DEGs between the model and BA groups.**

**(a)** Volcano plot of DEGs. **(b)** Heatmap of DEGs.


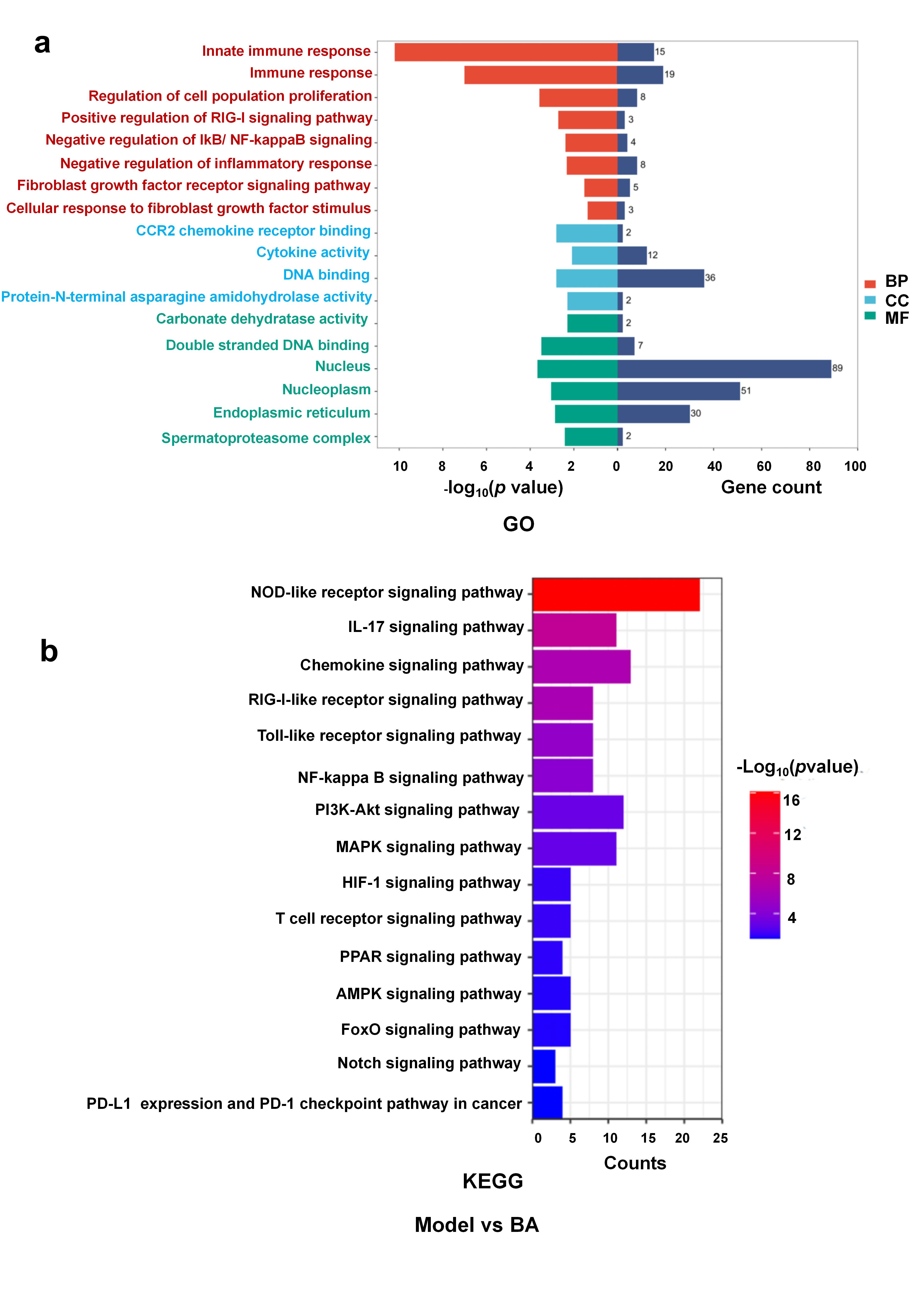
**Figure. S12 GO and KEGG enrichment analysis of DEGs between model and BA groups.**

**(a–b)** The DEGs between model and BA groups are analyzed by GO and KEGG.


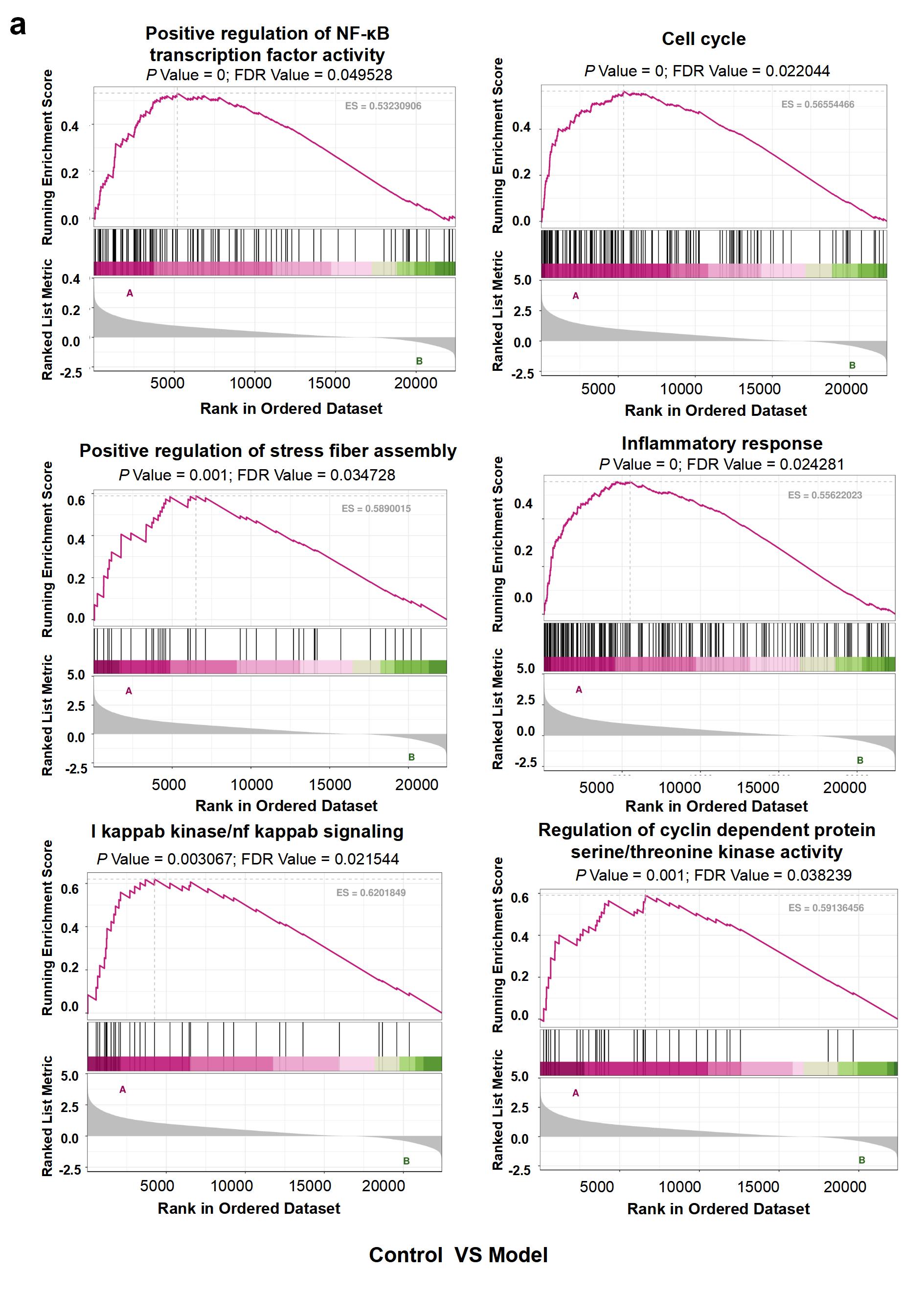


**Figure. S13** **GSEA analysis of the DEGs between the control and model groups.**

**(a)** The DEGs between the control and model groups are analyzed by using GSEA.


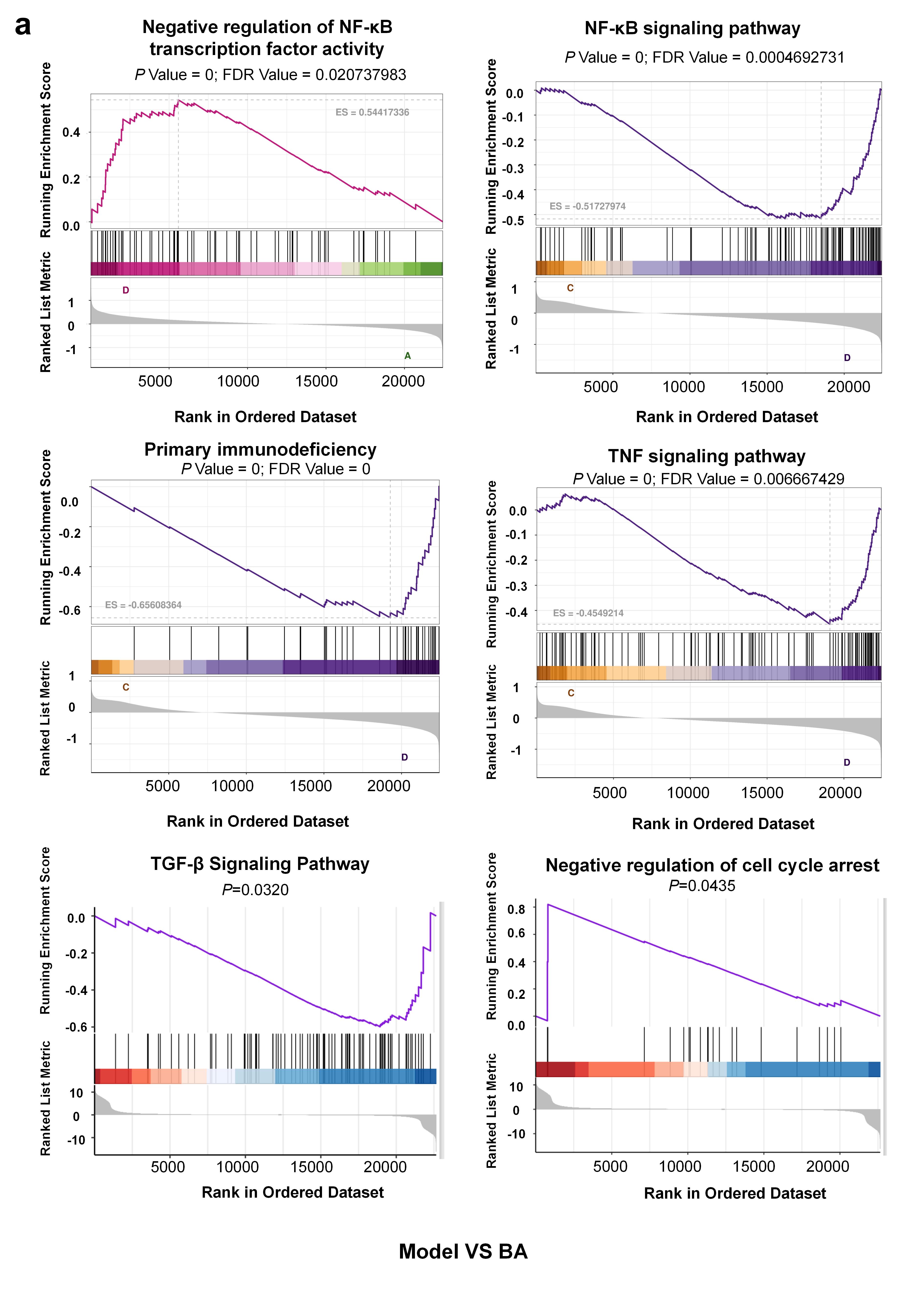


**Figure. S14** **GSEA analysis of the DEGs between the model and BA groups.**

**(a)**The DEGs between the model and BA groups are analyzed by using GSEA.
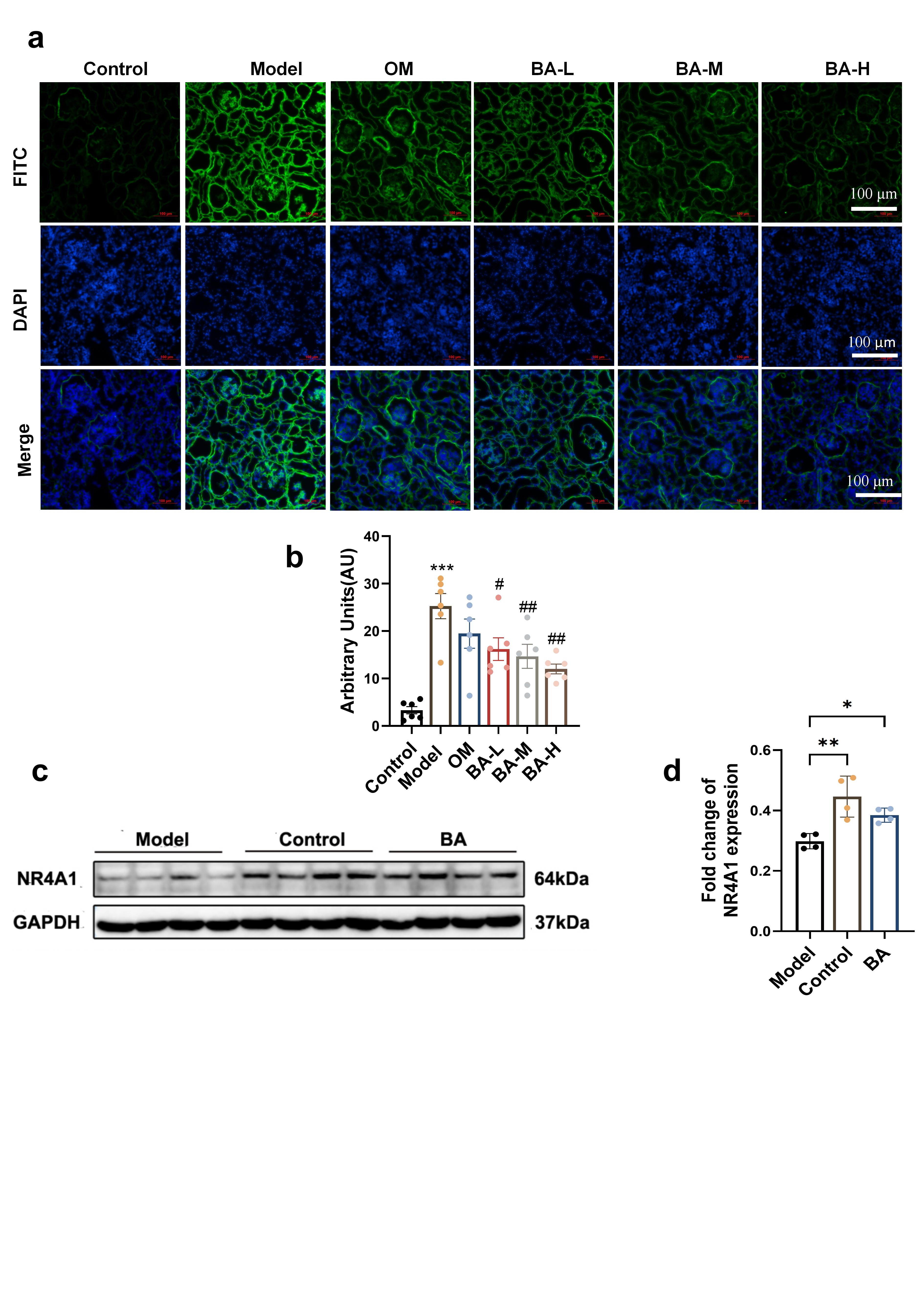


**Figure. S15 Therapeutic effect of BA on anti-Thy1 nephritis.**

**(a–b)** BA inhibits the expression of CCL2 protein in renal tissue. **(c–d)** Western blot is used to examine the effect of BA on the protein expression of NR4A1.


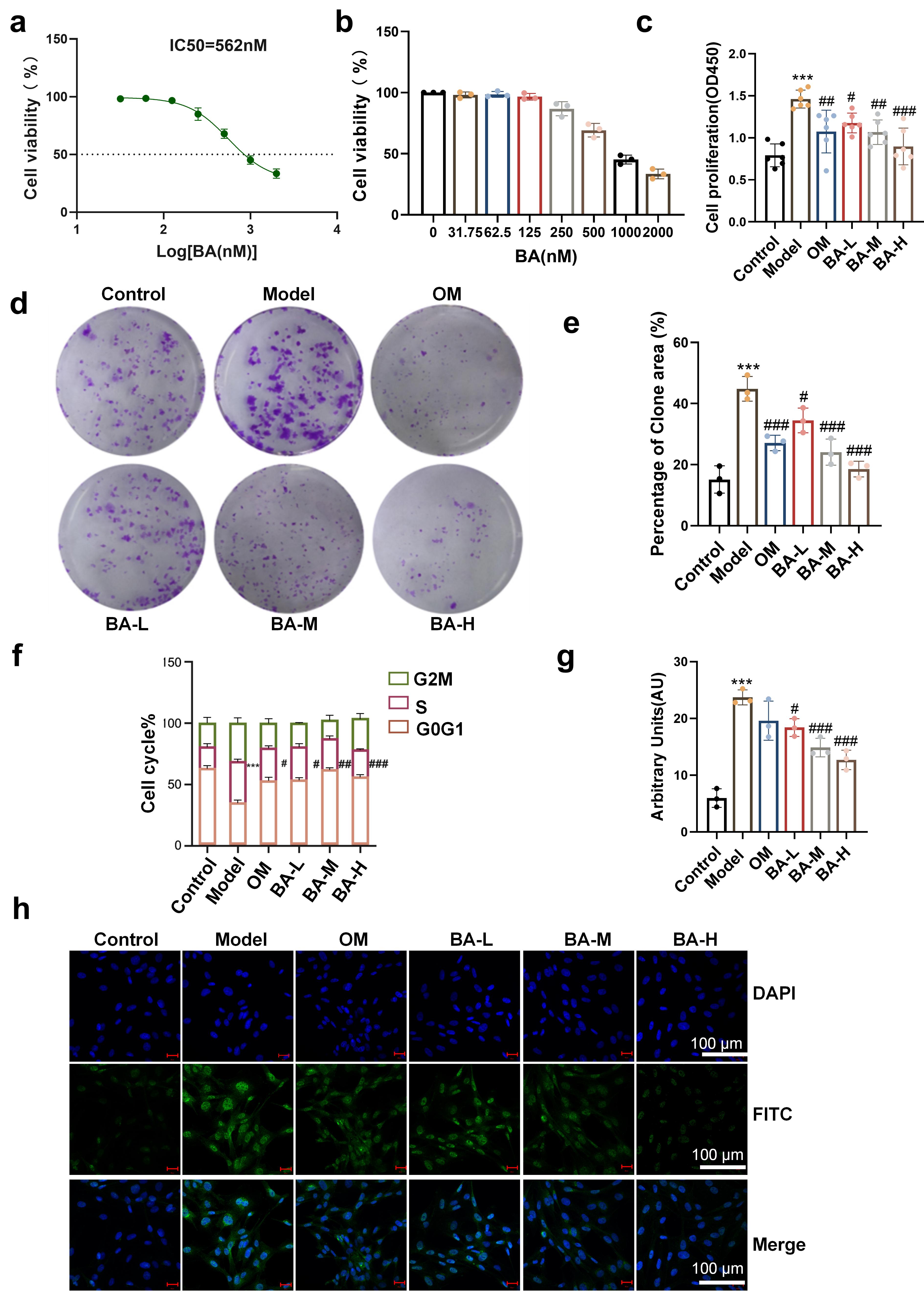


**Figure. S16 BA inhibits MCs proliferation and inflammation *in vitro*.**

**(a)**The IC50 of BA. **(b)** Detection of MCs viability by CCK-8 assay. **(c)** Proliferation of MCs treated with BA, as detected by CCK-8 assay. **(d-e)** Colony formation of MCs treated with BA. **(f)** Flow cytometry analysis of the effects of BA on the cell cycle distribution of MCs. **(g-h)** Effect of BA on CCL2 protein expression.


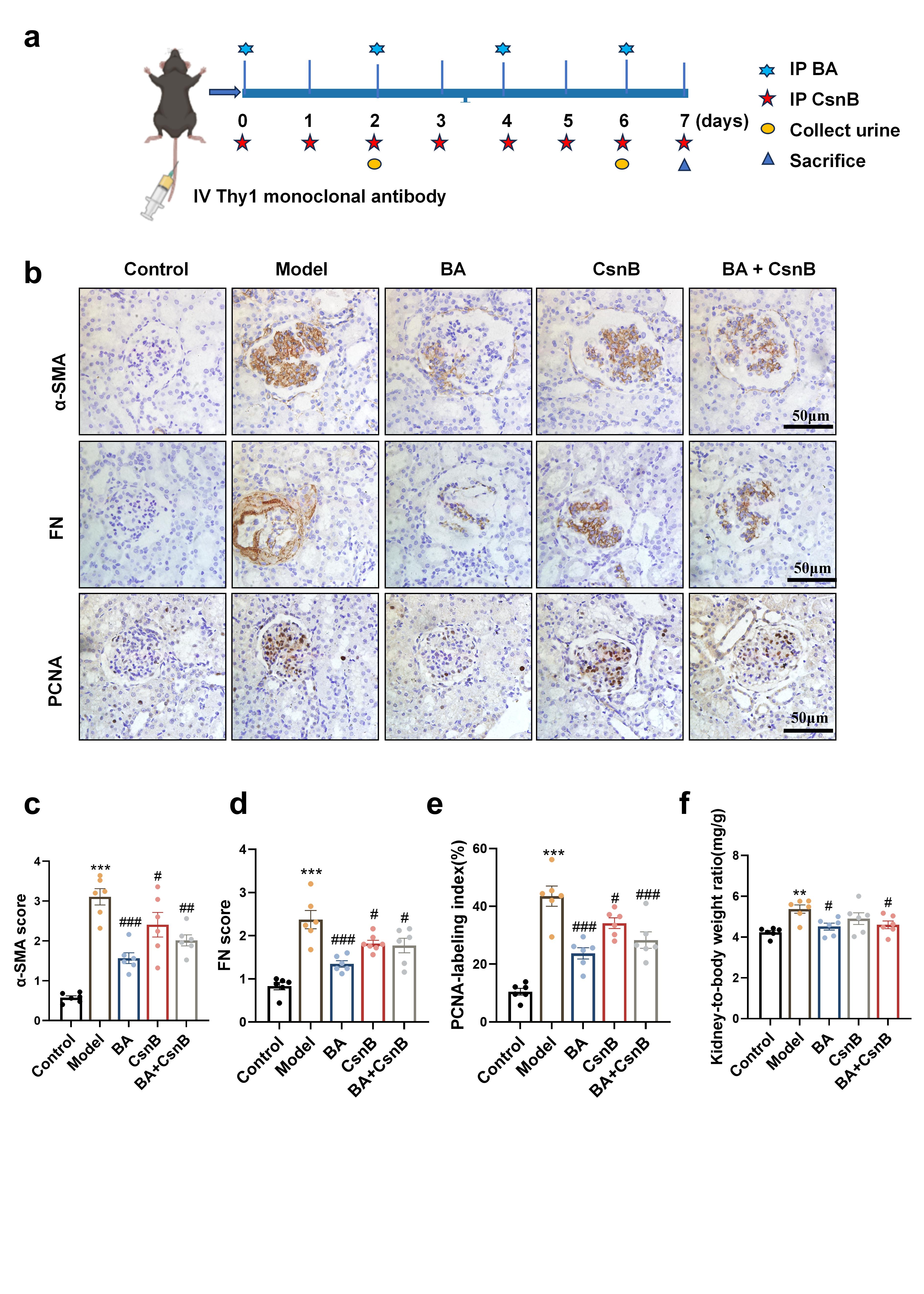
**Figure. S17 BA binds to NR4A1 in a competitive manner with CsnB *in vivo****.*

**(a)** Experimental flow diagram. **(b)** The Effects of BA and CsnB on PCNA, α-SMA and FN protein expression in the renal tissue of anti-Thy1 nephritis rats. **(c-e)** Semi-quantitative analysis of PCNA, α-SMA and FN protein expression. **(f)** The kidney indexes.


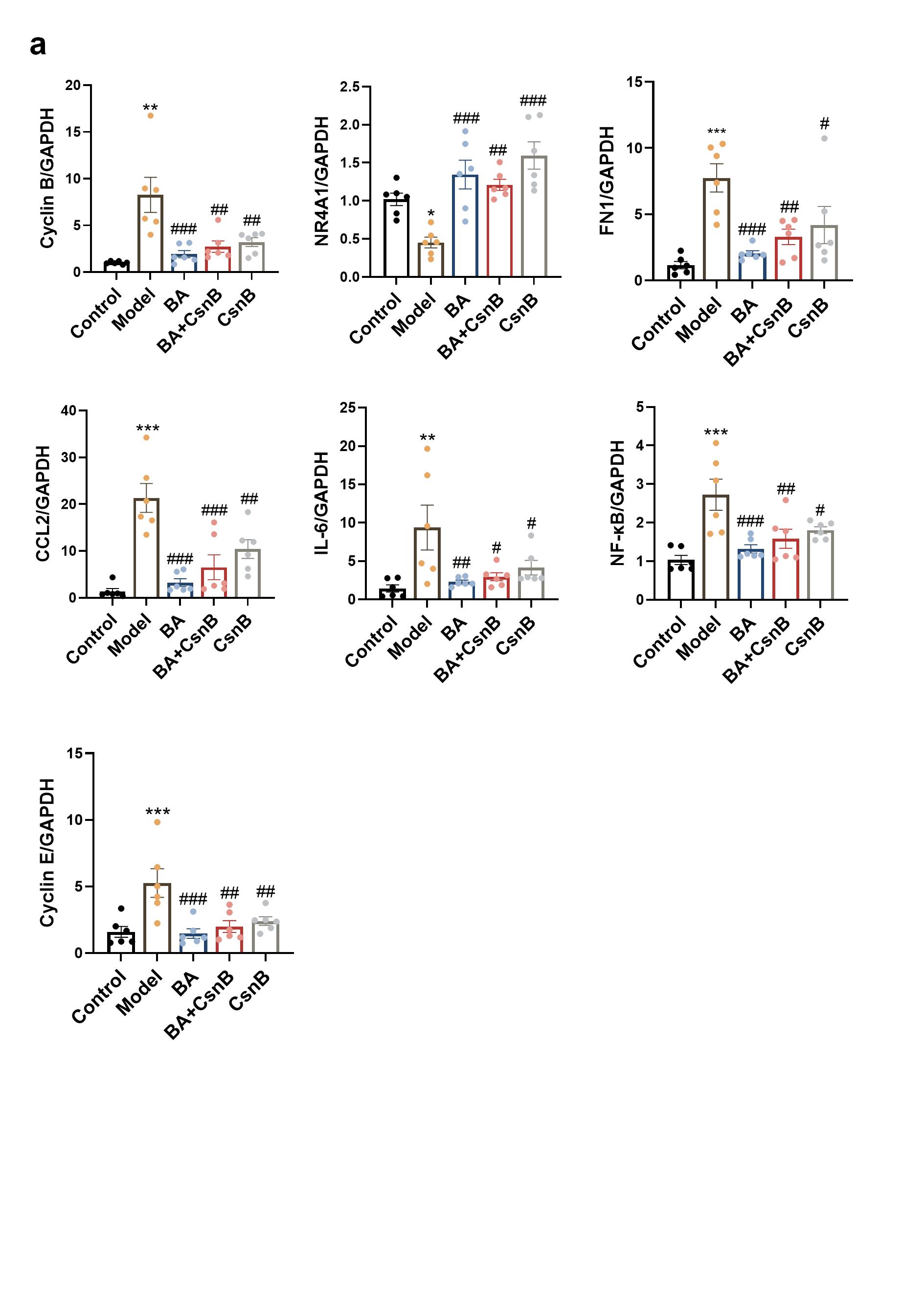


**Figure. S18** **BA and CsnB inhibit the expression of genes related to inflammation, proliferation and fibrosis *in vivo*.**

**(a)** Effects of BA or CsnB on the expression of IL-6, CCL2, NF-κB, Cyclin E, Cyclin B, NR4A1 and FN genes.


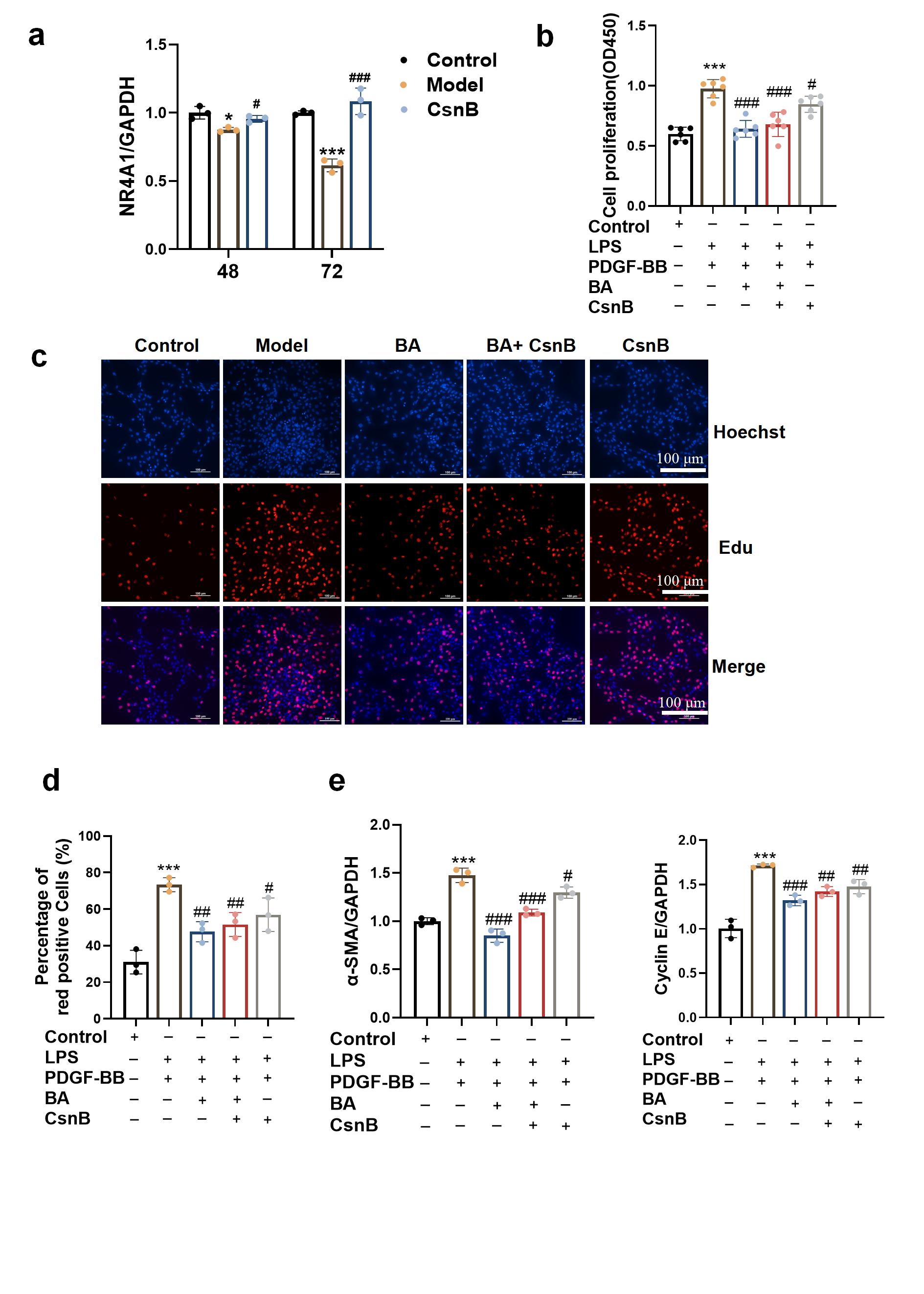
**Figure. S19 BA binds to NR4A1 in a competitive manner with CsnB *in vitro.***

**(a)** CsnB enhances the expression of the NR4A1 gene. **(b)** The effect of CsnB and BA on MCs proliferation. **(c–d)** Edu cell proliferation assay demonstrates the effect of BA and CsnB on MCs proliferation. **(e)** CsnB and BA regulate the expression of α-SMA and Cyclin E gene.


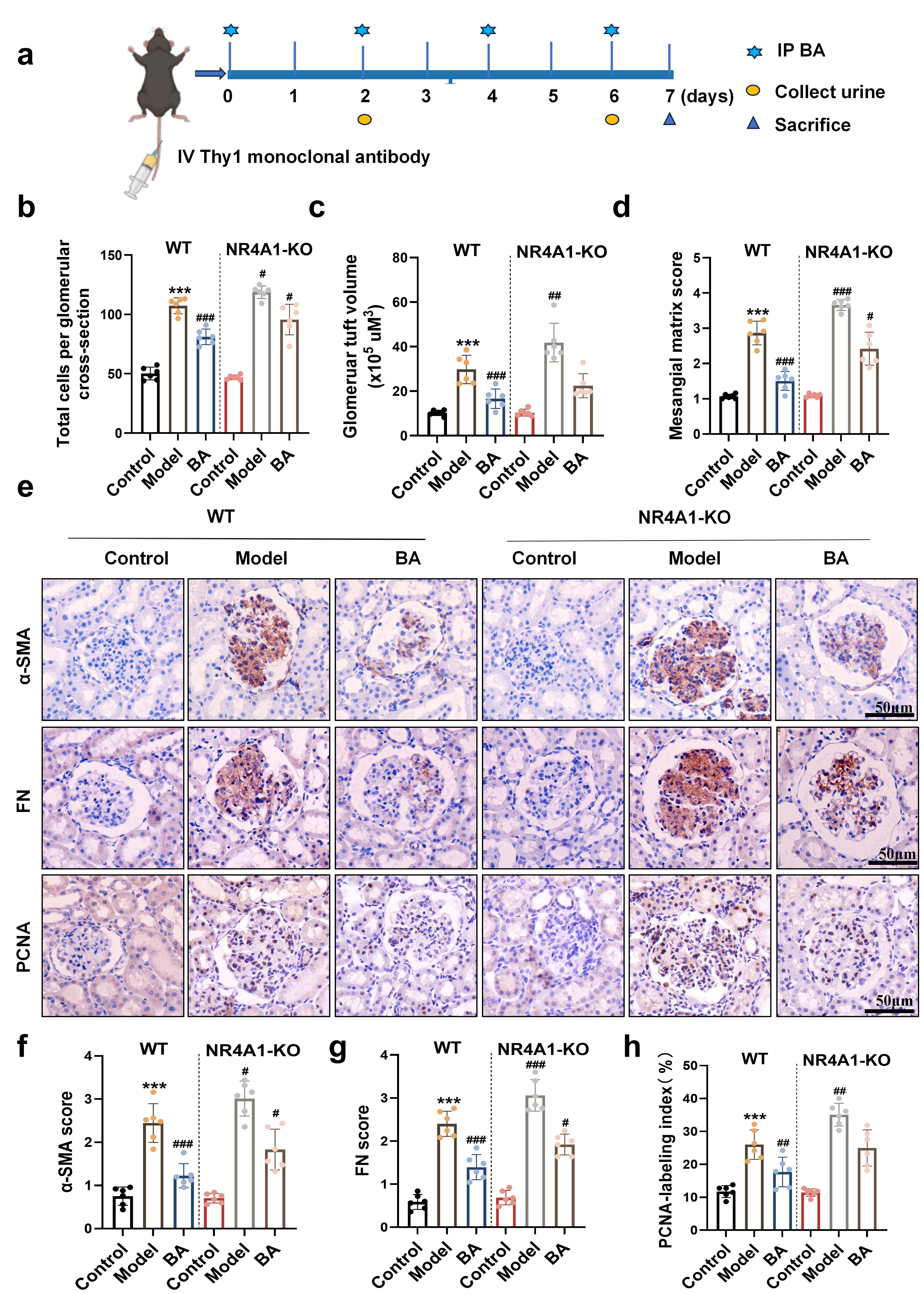
**Figure. S20 NR4A1 knockout aggravates kidney damage.**

**(a)** Experimental flow diagram. **(b-d)** Semi-quantitative analysis of renal pathology. **(e)** The effect of BA and CsnB on the protein expression of PCNA, α-SMA and FN in anti-Thy1 nephritis rat renal tissue. **(f-h)** Semi-quantitative analysis of the protein expression of PCNA, α-SMA and FN.


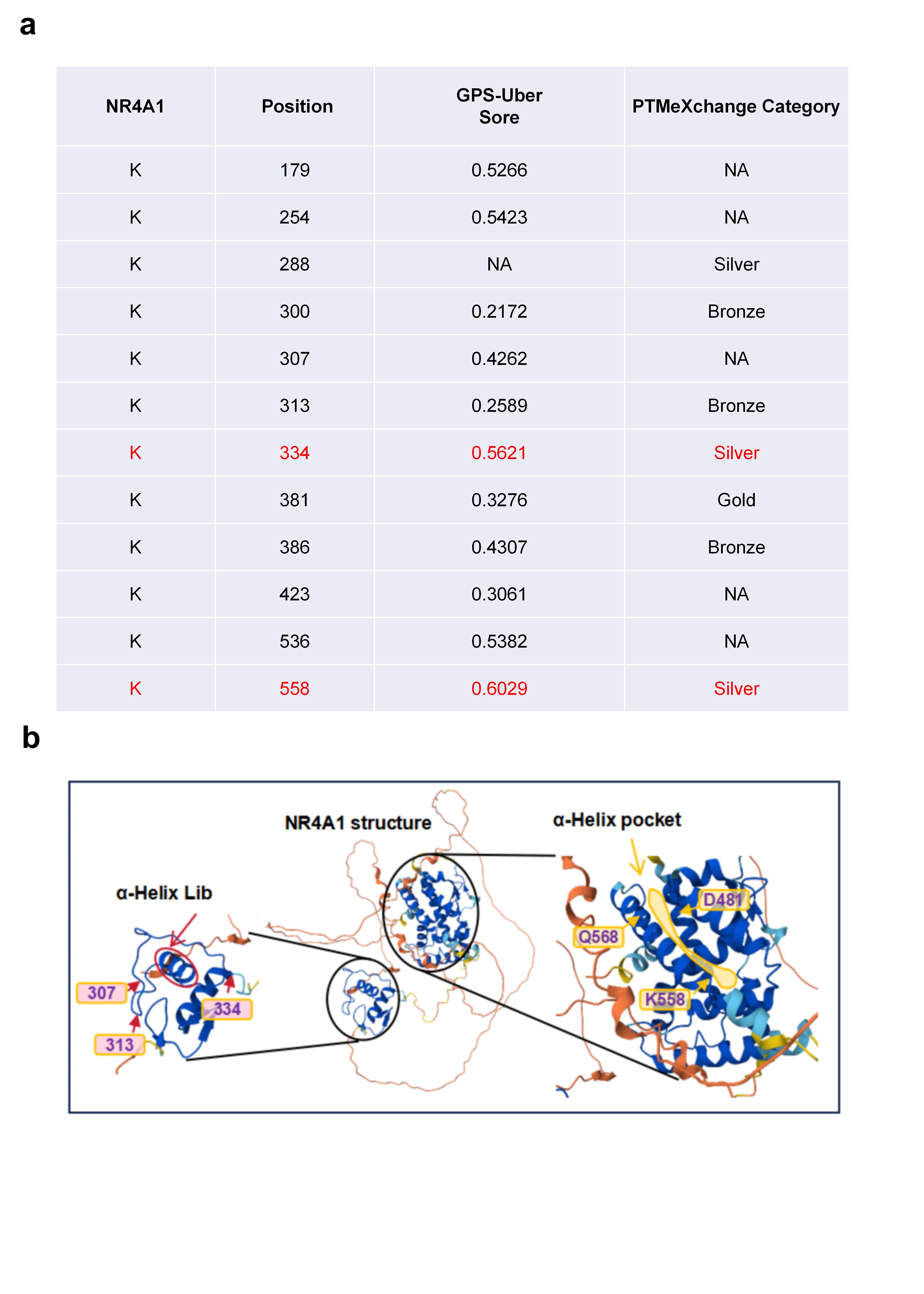


**Figure. S21 The key ubiquitination modification sites are predicted by using databases.**

**(a)**The GPS-Uber and PTMeXchange databases are used to predict the ubiquitination modification sites of NR4A1. **(b)**Structural diagram of the ubiquitination modification site and the BA binding site of NR4A1.

**Supplementary Tables**

**Table 1.** **gRNA target sequence of NR4A1**

| **Gene** | **gRNA target sequence** |
| --- | --- |
| ***NR4A1*** | gRNA-A1: AAGACCGGCCACACCCCAGT-GGG |
|  | gRNA-A2: ATCTTACAAAAACACGCGGA-AGG |
|  | gRNA-B1: TGAAAGTCAGCCGATCTGCT-TGG |
|  | gRNA-B2: GCTTGCCCAGCTGGCGGGGT-TGG |

**Table 2.** **Primer sequences of RT-PCR**

| **Gene** | **Forward primer** **(5 ′ –3 ′)** | **Reverse primer (3 ′ –5 ′)** |
| --- | --- | --- |
| ***IL-6*** | AGAGACTTCCAGCCAGTTGC | AGTCTCCTCTCCGGACTTGT |
| ***CCL2*** | TAGCATCCACGTGCTGTCTC | CAGCCGACTCATTGGGATCA |
| ***NF-κB*** | ATGGCAGACGACGATCCTTT | AGGTATGGGCCATCTGTTGAC |
| ***CyclinE*** | AGGCGAGGATGAGAGCAGTTC | AAGAAGTCCTGTGCCAAGTAGAATG |
| ***α-SMA*** | ACCATCGGGAATGAACGCTT | CTGTCAGCAATGCCTGGGTA |
| ***TGF-β*** | AGGGCTACCATGCCAACTTC | CCACGTAGTAGACGATGGGC |
| ***FN***  ***CyclinB*** | GGATCCCCTCCCAGAGAAGT  GATACTGCCTCTCCAAGCCC | GGGTGTGGAAGGGTAACCAG  TGTTCTTGACAGTCCATTCACCA |
| ***TNF-α*** | ACGTCGTAGCAAACCACCAA | AAATGGCAAATCGGCTGACG |
| ***GAPDH*** | CAGTGGCAAAGTGGAGATTGTTG | TCGCTCCTGGAAGATGGTGAT |
|  |  |  |

**Table 3. Characteristics of participants**

| Study ID | Group | Gender | Age (Years) | eGFR (mL/min/1.73 m2) | CKD stage | Specimens | Pathological Diagnosis (Lee grading, Oxford classification) |
| --- | --- | --- | --- | --- | --- | --- | --- |
| IgAN_01 | IgAN | female | 33 | 93.52 | 1 | kidney tissue | IgAN (Ⅱ, M1E0S1T0C0) |
| IgAN_02 | IgAN | female | 34 | 115.85 | 1 | kidney tissue | IgAN (Ⅲ, M1E0S1T0C0) |
| IgAN_03 | IgAN | female | 50 | 64.44 | 2 | kidney tissue | IgAN (Ⅲ, M1E0S1T0C1) |
| IgAN_04 | IgAN | female | 49 | 70.54 | 2 | kidney tissue | IgAN (Ⅲ, M1E0S1T0C0) |
| IgAN_05 | IgAN | female | 35 | 61.54 | 2 | kidney tissue | IgAN (Ⅱ, M1E0S0T0C0) |
| IgAN_06 | IgAN | male | 44 | 49.28 | 3a | kidney tissue | IgAN (Ⅳ, M1E0S1T1C1) |
| IgAN_07 | IgAN | male | 54 | 43.08 | 3b | kidney tissue | IgAN (Ⅳ, M1E1S1T1C1) |
| IgAN_08 | IgAN | female | 36 | 46.01 | 3a | kidney tissue | IgAN (Ⅴ, M1E1S1T2C0) |
| NM_01 | normal control | male | 67 | 73.41 | 2 | kidney tissue | Urothelial carcinoma |
| NM_02 | normal control | female | 56 | 93.51 | 1 | kidney tissue | Hydronephrosis with kidney stones |
| NM_03 | normal control | male | 49 | 106.84 | 1 | kidney tissue | Chromophobe renal cell carcinoma |
| NM_04 | normal control | female | 73 | 46.68 | 3a | kidney tissue | Clear cell renal cell carcinoma |
| NM_05 | normal control | female | 39 | 101.28 | 1 | kidney tissue | Renal cell carcinoma |
| NM_06 | normal control | female | 33 | 75.64 | 2 | kidney tissue | Urothelial carcinoma |
| NM_07 | normal control | female | 45 | 105.54 | 1 | kidney tissue | Renal angiomyolipoma |
| NM_08 | normal control | female | 65 | 63.04 | 2 | kidney tissue | Urothelial carcinoma |
|  |  |  |  |  |  |  |  |

eGFR, estimated glomerular filtration rate; CKD, chronic kidney disease.

| Variables | IgAN (n=8) | NM (n=8) | *P* value |
| --- | --- | --- | --- |
| Gender (n, %) |  |  |  |
| male | 2 (25.0) | 2 (25.0) |  |
| female | 6 (75.0) | 6 (75.0) |  |
| Age [years, mean ± sd] | 41.9 ± 8.4 | 53.4 ± 14.3 | 0.094 |
| Body temperature (℃) | 36.4 ± 0.2 | 36.4 ± 0.2 | 0.801 |
| MAP (mmHg) | 89.4 ± 8.7 | 100.4 ± 9.8 | 0.794 |
| Laboratory values(mean ± sd) |  |  |  |
| Serum creatinine (μmol/L) | 104.8 ± 35.4 | 79.7 ± 20.9 | 0.096 |
| Urea (mmol/L) | 5.5 ± 1.7 | 5.3 ± 1.6 | 0.464 |
| Uric Acid (μmol/L) | 377.3 ± 85.2 | 372.0 ± 93.8 | 0.951 |
| eGFR [ml/(min•1.73m2)] | 68.0 ± 25.2 | 83.2 ± 22.0 | 0.95 |
| Serum albumin (g/L) | 41.8 ± 2.8 | 42.2 ± 5.0 | 0.246 |
| ALT (U/L) | 17.6 ± 9.0 | 17.6 ± 12.1 | 0.687 |
| AST (U/L) | 19.1 ±3.9 | 20.3 ± 14.6 | 0.18 |
| Hemoglobin (g/L) | 119.1 ± 21.5 | 119.1 ± 23.3 | 0.898 |
| WBC (10^9/L) | 7.4 ± 3.4 | 8.1 ± 1.9 | 0.512 |
| RBC (10^12/L) | 7.8 ± 10.3 | 8.7 ± 11.4 | 0.863 |
| PLT (10^9/L) | 260.0 ± 68.0 | 218.4 ± 70.1 | 0.644 |
| Serum Calcium (mmol/L) | 2.3 ± 0.2 | 1.1 ± 0.9 | 0.14 |
| Phosphorus (mmol/L) | 1.2 ± 0.2 | 1.1 ± 0.1 | 0.241 |
| FPG (mmol/L) | 5.8 ± 0.8 | 7.8 ± 3.7 | 0.117 |
| HbA1c (%) | 5.6 ± 0.4 | 5.9 ± 0.8 | 0.088 |
| Triglyceride (mmol/L) | 1.5 ± 0.6 | 2.3 ± 1.6 | 0.146 |
| Total Cholesterol (mmol/L) | 4.9 ± 1.0 | 4.4 ± 1.1 | 0.887 |
| HDL-C (mmol/L) | 1.2 ± 0.2 | 1.0 ± 0.4 | 0.058 |
| LDL-C (μmol/L) | 3.1 ± 0.9 | 2.4 ± 1.0 | 0.646 |
| IgA (g/L) | 2.9 ± 1.1 | — | — |
| IgG (g/L) | 9.0 ± 3.8 | — | — |
| IgM (g/L) | 1.1 ± 0.5 | — | — |
| C3 (g/L) | 1.0 ± 0.2 | — | — |
| C4 (g/L) | 0.3 ± 0.1 | — | — |
| UPCR (mg/g) | 964.1 ± 678.1 | — | — |
| 24h Proteinuria (mg) | 1070.8 ± 773.6 | — | — |

**Table 4. Demographic and clinical characteristics of the participants at baseline**

IgA nephropathy, IgAN; NM, normal; MAP, mean arterial pressure; eGFR, estimated glomerular filtration rate; ALT, alanine aminotransferase; AST, aspartate aminotransferase; WBC, white blood cell; RBC, red blood cell; PLT, blood platelet; FPG, fasting plasma glucose; HDL-C, high density lipoprotein cholesterol; LDL-C, low-density lipoprotein cholesterol; UPCR, urinary protein-to-creatinine ratio.

**Table 5. Basic Information and Pathological Diagnosis of Single-Cell Transcriptome Clinical Samples**

| Study ID | Group |  | Gender | Age (Years) | eGFR (mL/min/1.73 m^2^) | CKD stage | Specimens | Pathological Diagnosis (Oxford classification) |
| --- | --- | --- | --- | --- | --- | --- | --- | --- |
| IgAN_01 | IgAN |  | female | 34 | 115.27 | 1 | kidney tissue | IgAN (M1E0S0T0C0) |
| IgAN_02 | IgAN |  | female | 38 | 70.27 | 3a | kidney tissue | IgAN (M1E0S0T0C0) |
| IgAN_03 | IgAN |  | female | 34 | 105.15 | 1 | kidney tissue | IgAN (M1E0S0T0C0) |
| IgAN_04 | IgAN |  | female | 60 | 23.21 | 4 | kidney tissue | IgAN (M1E1S0T0C2) |
| IgAN_05 | IgAN |  | male | 34 | 92.56 | 1 | kidney tissue | IgAN (M1E0S1T1C1) |
| IgAN_06 | IgAN |  | female | 61 | 43.53 | 3b | kidney tissue | IgAN (M1E0S1T1C1) |
| IgAN_07 | IgAN |  | female | 28 | 113.83 | 1 | kidney tissue | IgAN (M1E0S0T0C0) |
| IgAN_08 | IgAN |  | female | 39 | 95.98 | 1 | kidney tissue | IgAN (M1E0S1T0C0) |
| IgAN_09 | IgAN |  | female | 54 | 44.25 | 3b | kidney tissue | IgAN (M1E0S1T2C0) |
| NC_KID_01 | normal control |  | female | 39 | 101.28 | 3a | kidney tissue | Renal cell carcinoma |
| NC_KID_02 | normal control |  | female | 36 | 109.43 | 3a | kidney tissue | Renal angiomyolipoma |
| NC_KID_03 | normal control |  | female | 58 | 97.87 | 3a | kidney tissue | kidney stones |
| NC_KID_04 | normal control |  | female | 33 | 75.64 | None | kidney tissue | High-grade invasive papillary urothelial carcinoma of the renal pelvis |
| NC_KID_05 | normal control |  | male | 59 | 75.18 | None | kidney tissue | Clear cell renal cell carcinoma |
| NC_KID_06 | normal control |  | female | 35 | 112.35 | None | kidney tissue | Clear cell renal cell carcinoma |
| NC_KID_07 | normal control |  | female | 37 | 108.66 | None | kidney tissue | Clear cell renal cell carcinoma |
| NC_KID_08 | normal control |  | female | 64 | 79.14 | None | kidney tissue | Chromophobe Renal Cell Carcinoma |
| NC_KID_09 | normal contrl |  | male | 60 | 94.26 | None | kidney tissue | Papillary Renal Cell Carcinoma |

Abbreviations: IgAN, IgA Nephropathy; NC, Normal Control; KID, Kidney Tissue; eGFR, estimated Glomerular Filtration Rate; CKD, Chronic Kidney Disease.

**Table 6. Demographic and Clinical Characteristics of Single-Cell Transcriptome Renal Tissue Samples**

| Variables | IgAN (n=9） | NC (n=9） | *P* value |
| --- | --- | --- | --- |
| Gender (n,%) |  |  | 0.527 |
| male | 1 (11.1) | 2 (22.2) |  |
| female | 8 (88.9) | 7 (77.8) |  |
| Age [years,M（P25-P75）] | 38.0 (34.0-57.0) | 39.0 (35.5-59.5) | 0.452 |
| Laboratory values |  |  |  |
| Serum creatinine (μmol/L) | 90.0 (65.0-118.5) | 66.0 (62.5-73.5) | 0.133 |
| Urea (mmol/L) | 5.4 ± 2.1 | 4.9 ± 1.7 | 0.643 |
| Uric Acid (μmol/L) | 362.0 (301.0-419.0) | 302.5 (295.0-337.0) | 0.194 |
| eGFR [ml/(min•1.73m2)] | 78.2 ± 34.2 | 94.9 ± 14.8 | 0.207 |
| UPCR (mg/g) | 965.2 ± 898.2 | — | — |
| ALT (U/L) | 23.8 ± 19.7 | 19.7 ± 12.6 | 0.605 |
| AST (U/L) | 18.0(16.5-23.5) | 18.0 (15.5-22.5) | 0.929 |
| Hemoglobin (g/L) | 130.2 ± 17.8 | 125.6 ± 19.7 | 0.605 |
| WBC (10^9/L) | 7.5 ± 1.7 | 7.2 ± 0.9 | 0.610 |
| RBC (10^12/L) | 273.0 (252.0-351.5) | 229.0 (204.5-299.5) | 0.171 |
| PLT (10^9/L) | 291.6 ± 57.0 | 252.2 ± 55.4 | 0.157 |
| Serum Calcium (mmol/L) | 2.3 ± 0.1 | 2.4 ± 0.1 | 0.378 |
| Phosphorus (mmol/L) | 1.1 ± 0.2 | 1.1 ± 0.1 | 0.608 |
| Potassium (mmol/L) | 3.9 ± 0.4 | 3.9 ± 0.5 | 0.960 |
| FPG (mmol/L) | 4.9 (4.8-6.4) | 5.8(5.4-6.6) | 0.102 |
| HbA1c (%) | 5.5 ± 0.6 | 5.8 ± 0.4 | 0.421 |
| Triglyceride (mmol/L) | 1.8 ± 0.9 | 1.5 ± 0.5 | 0.534 |
| Total Cholesterol (mmol/L) | 4.79 ± 1.08 | 4.67 ± 0.77 | 0.809 |
| HDL-C (mmol/L) | 1.3 ± 0.4 | 1.3 ± 0.3 | 0.862 |
| LDL-C (mmol/L) | 3.0 ± 1.0 | 2.8 ± 0.5 | 0.553 |

Abbreviations: IgAN, IgA Nephropathy; NC, normal Control; eGFR, estimated Glomerular Filtration Rate; UPCR, urinary protein-to-creatinine ratio; ALT, alanine aminotransferase; AST, aspartate aminotransferase; WBC, white blood cell; RBC, red blood cell; PLT, blood platelet; FPG, fasting plasma glucose; HbA1c, glycated Hemoglobin; HDL-C, high density lipoprotein cholesterol; LDL-C, low-density lipoprotein cholesterol.

**References**

1. Zhang, D. et al. Identification of natural compounds as SARS-CoV-2 entry inhibitors by molecular docking-based virtual screening with bio-layer interferometry. *Pharmacol Res.* **172**, 105820 (2021).

2. Guo, L. et al. Targeted delivery of celastrol to mesangial cells is effective against mesangioproliferative glomerulonephritis. *Nat Commun.* **8**, 878 (2017).

3. Floege, J., Eng, E., Young, B.A., Couser, W.G. & Johnson, R.J. Heparin suppresses mesangial cell proliferation and matrix expansion in experimental mesangioproliferative glomerulonephritis. *Kidney Int.* **43**, 369-80 (1993).

4. Han, D. et al. Microenvironmental network of clonal CXCL13+CD4+ T cells and Tregs in pemphigus chronic blisters. *J Clin Invest.* **133**(2023).

5. Jorch, S.K. et al. Complex regulation of alarmins S100A8/A9 and secretion via gasdermin D pores exacerbates autoinflammation in familial Mediterranean fever. *J Allergy Clin Immunol.* **152**, 230-243 (2023).

6. Borot, F. et al. Multiplex base editing to protect from CD33 directed drugs for immune and gene therapy. *Nat Commun.* **16**, 4899 (2025).

7. Liu, X. et al. The Ubiquitin-like Protein FAT10 Stabilizes eEF1A1 Expression to Promote Tumor Proliferation in a Complex Manner. *Cancer Res.* **76**, 4897-907 (2016).

8. Han, L. et al. circSATB1 Modulates Cell Senescence in Age-Related Acute Myeloid Leukemia: A Mechanistic Proposal. *Cells.* **14**(2025).
