# Supplementary material for: Bruceine A protects nuclear receptor 4A1 from ubiquitin-degradation to alleviate mesangial proliferative glomerulonephritis
Source: Signal Transduct Target Ther. 2025 Dec 5;10:397. doi: 10.1038/s41392-025-02495-2 (PMC12678413; doi:10.1038/s41392-025-02495-2)
Supplement: Supplementary file 2 — WB figures [file 41392_2025_2495_MOESM2_ESM.pdf]

Western blot analysis of NR4A1 protein levels following siRNA-mediated knockdown *in vitro*

Repeat 1

Control siNC siNR4A1 Control siNC siNR4A1

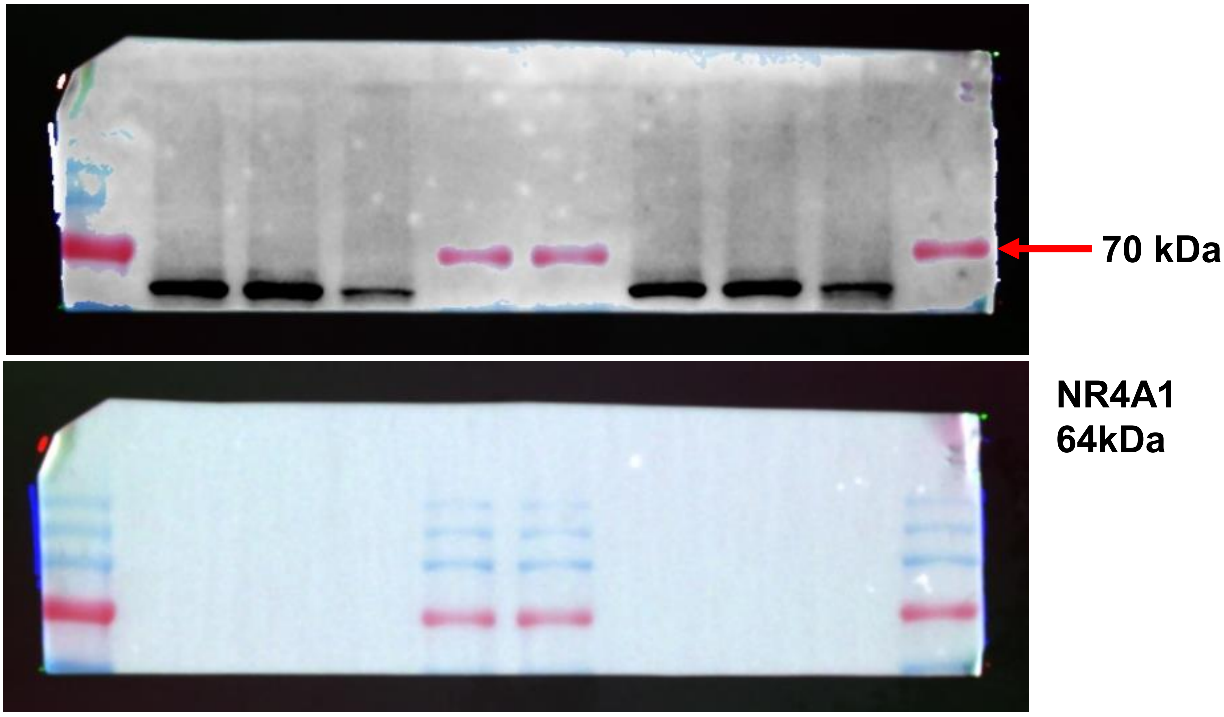

70 kDa

NR4A1 64kDa

Repeat 1

Control siNC siNR4A1 Control siNC siNR4A1

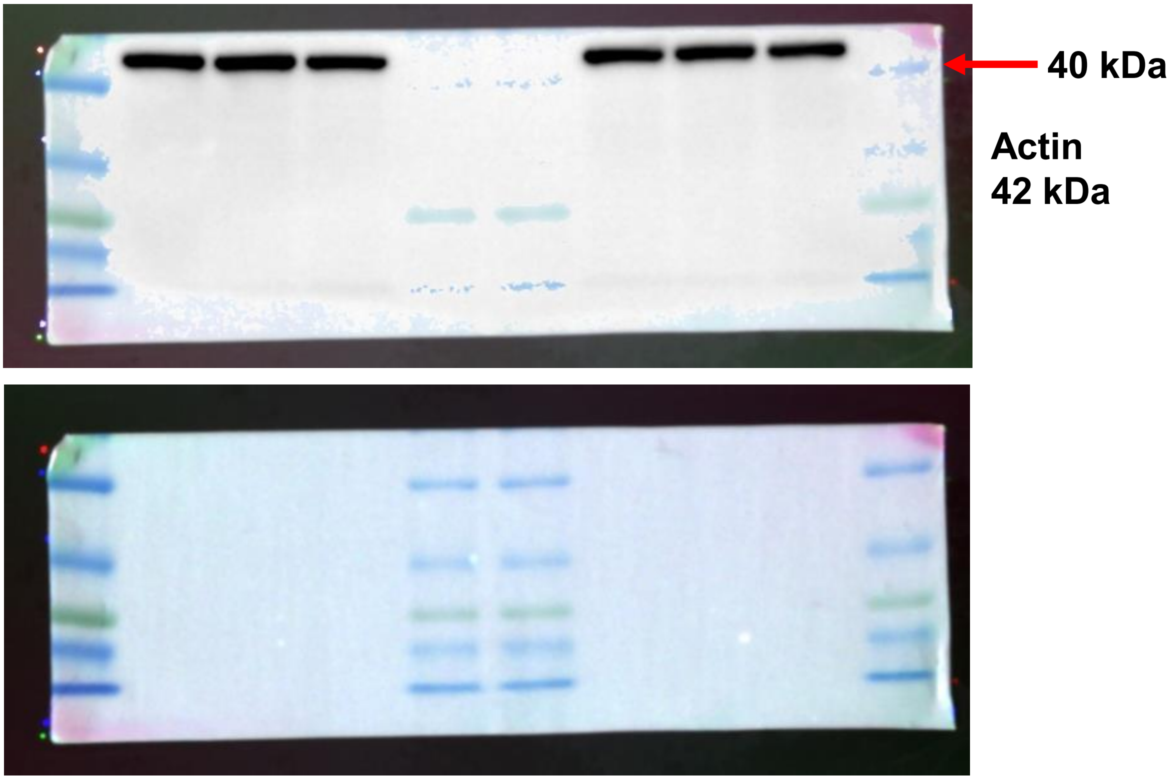

40 kDa

Actin 42 kDa

Repeat 1 The merged image

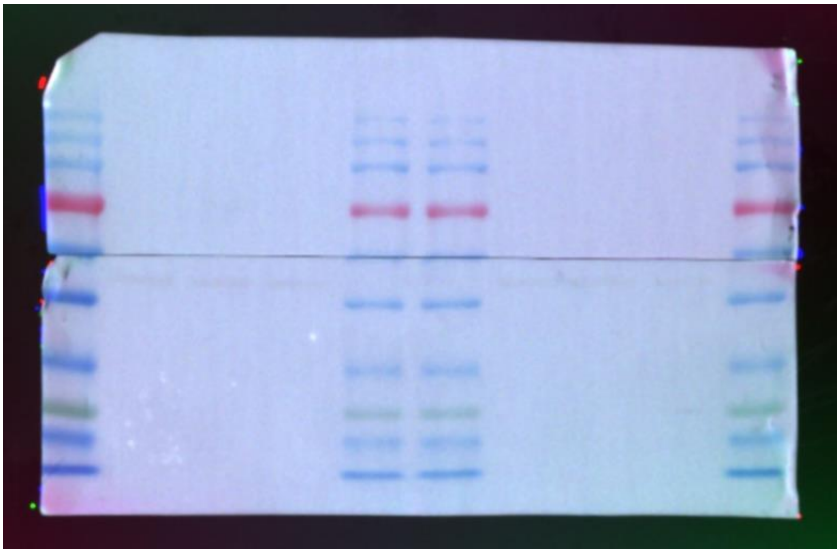

Repeat 2

Control siNC siNR4A1 Control siNC siNR4A1

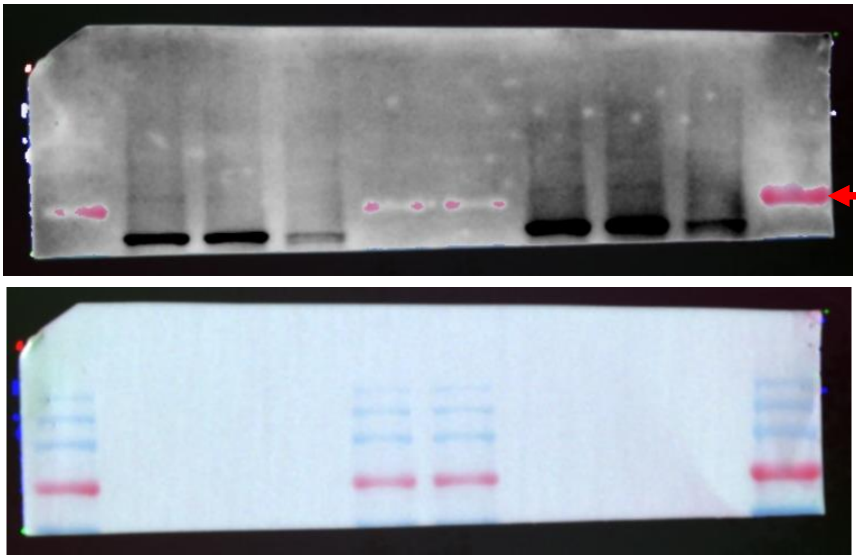

70 kDa

NR4A1
64kDa

Control siNC siNR4A1 Control siNC siNR4A1

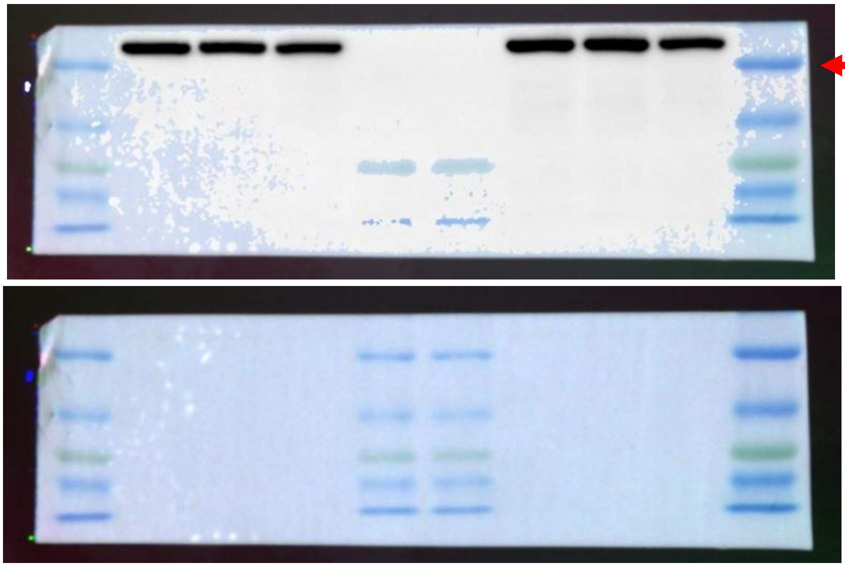

40 kDa

Actin
42 kDa

Repeat 2 The merged image

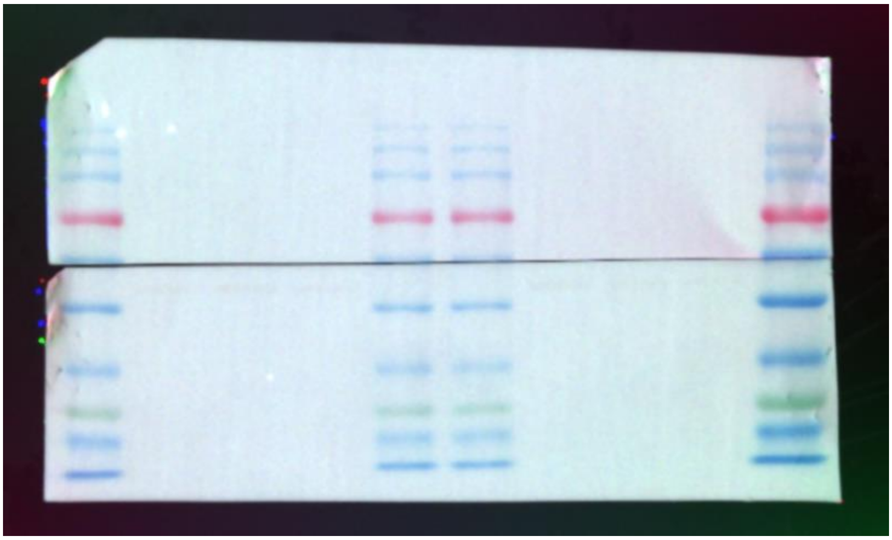

Repeat 3

Control siNC siNR4A1 Control siNC siNR4A1

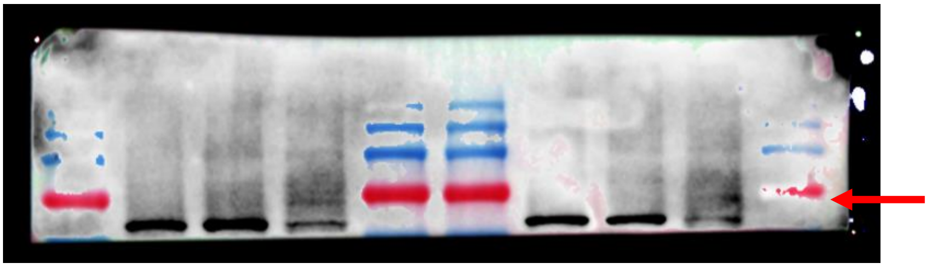

70 kDa

NR4A1
64kDa

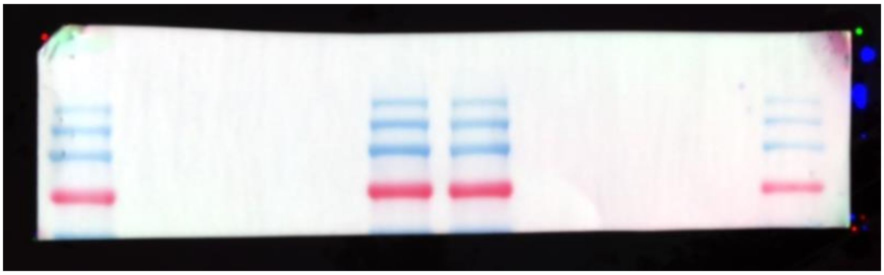

Repeat 3

Control siNC siNR4A1 Control siNC siNR4A1

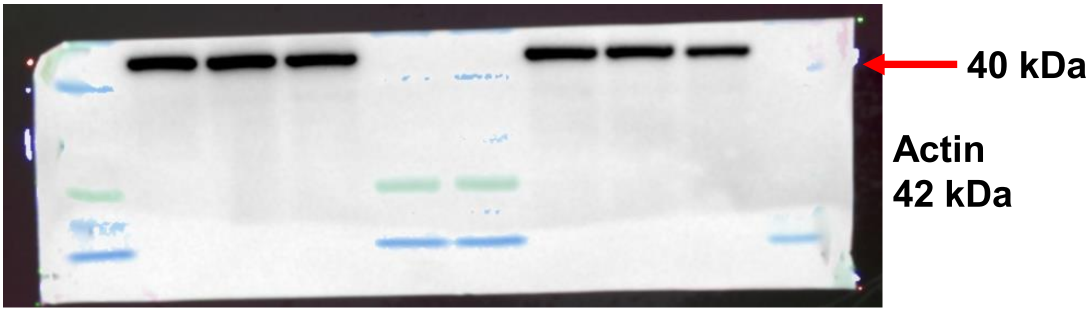

40 kDa

Actin
42 kDa

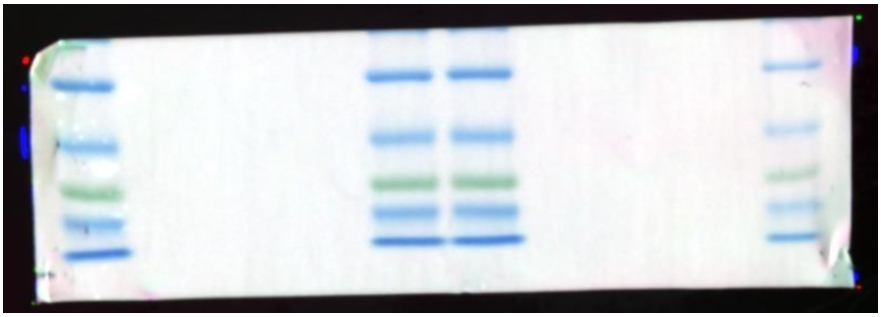

Repeat 3 The merged image

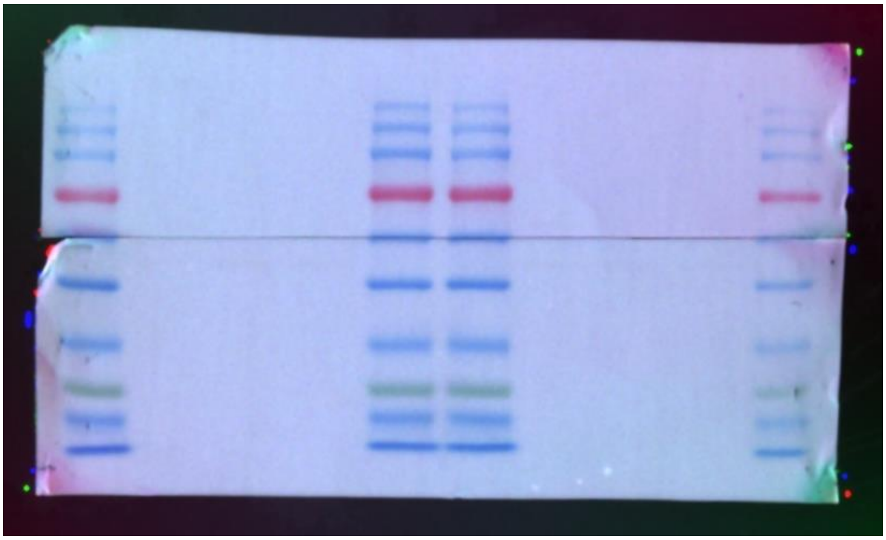

Western blot was performed to analyze the effect of siNR4A1 knockdown on the NR4A1 protein under model conditions *in vitro*

Repeat 1

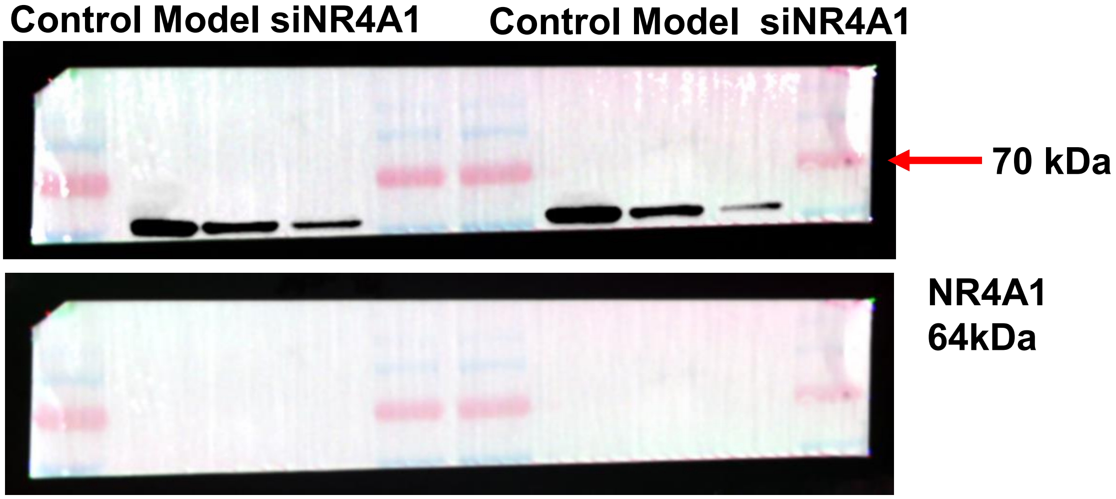

Repeat 1

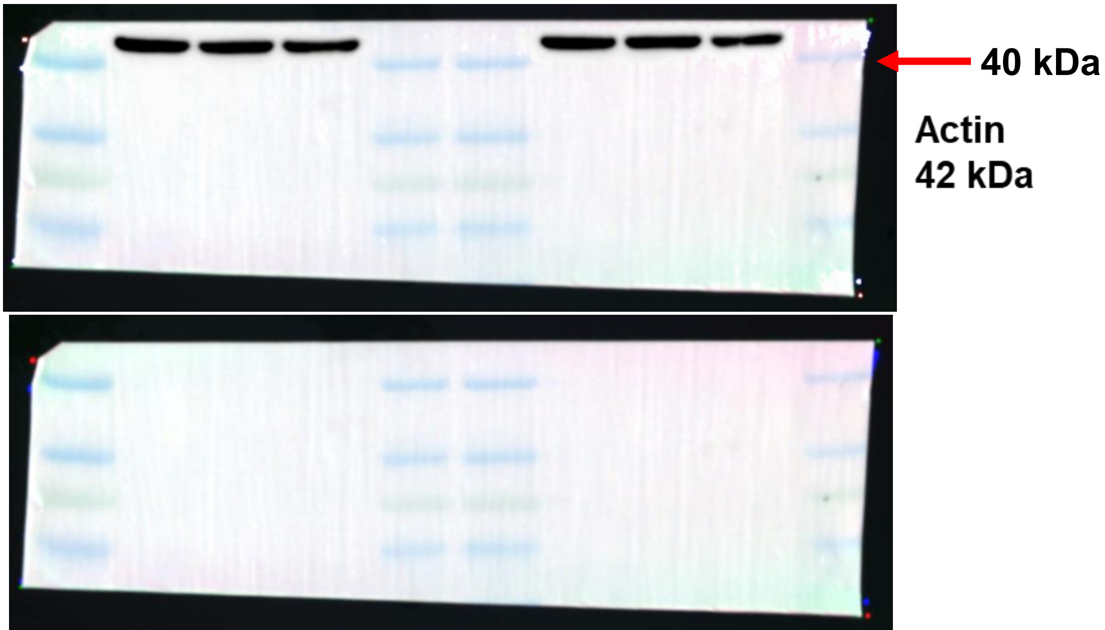

Repeat 1 The merged image

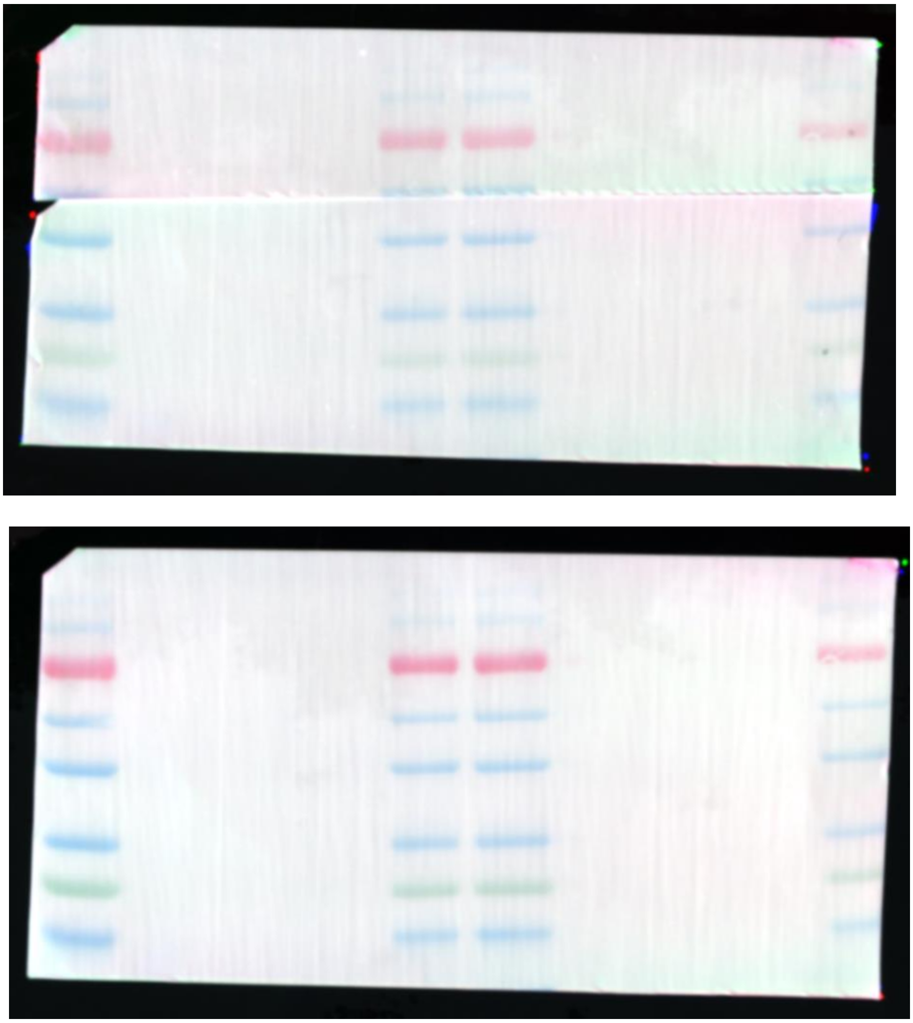

Repeat 2

Control Model siNR4A1 Control Model siNR4A1

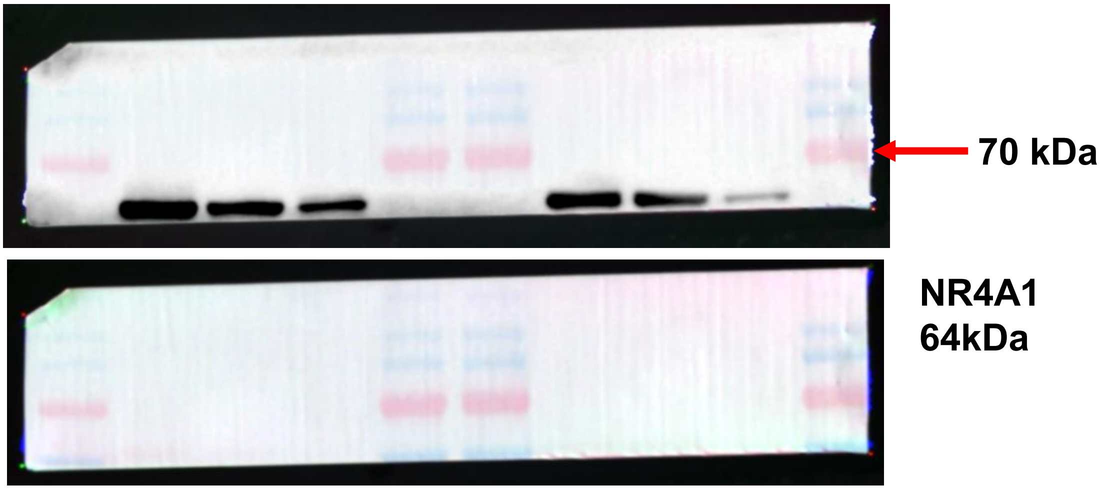

70 kDa

NR4A1
64kDa

Repeat 2

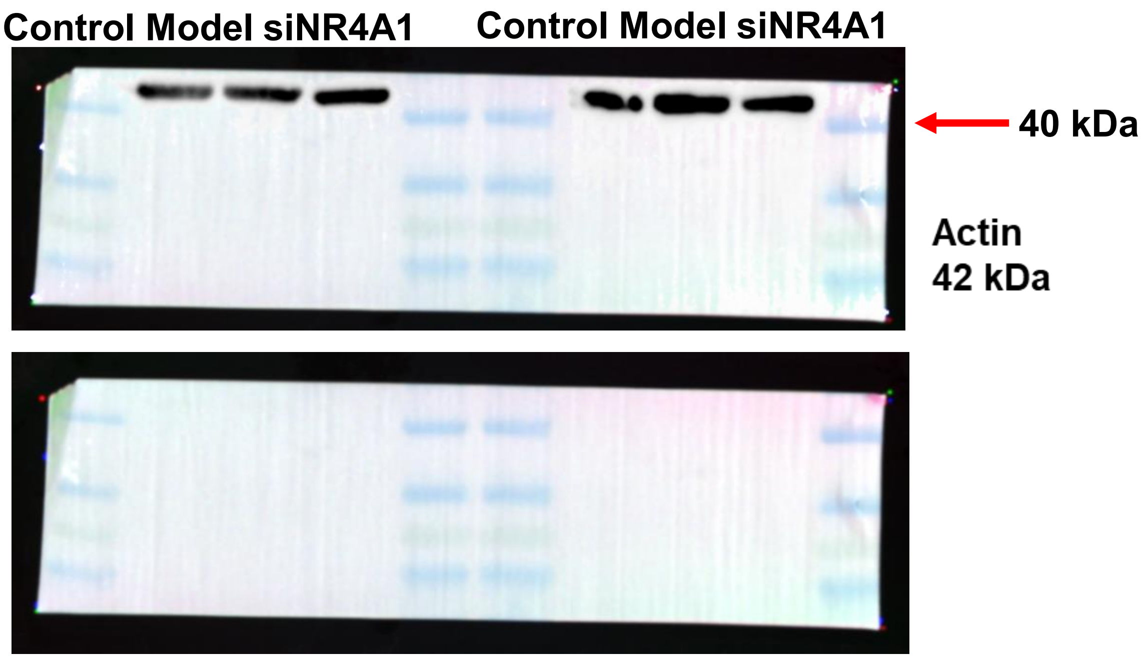

Repeat 2 The merged image

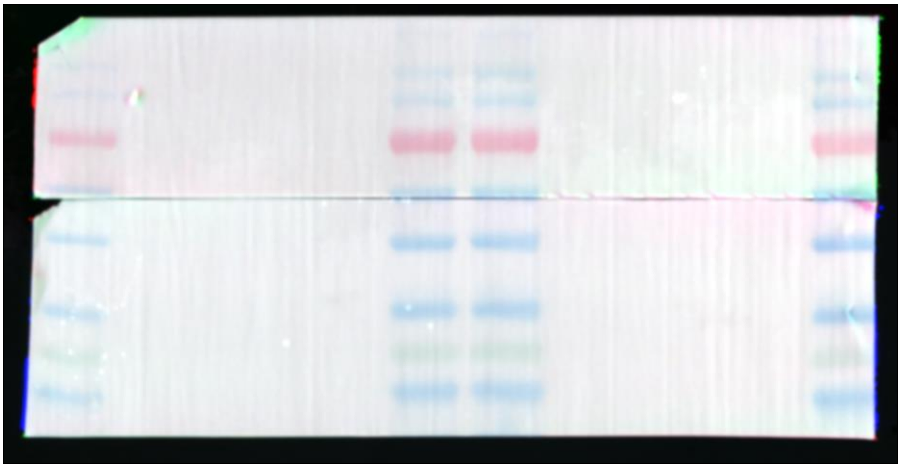

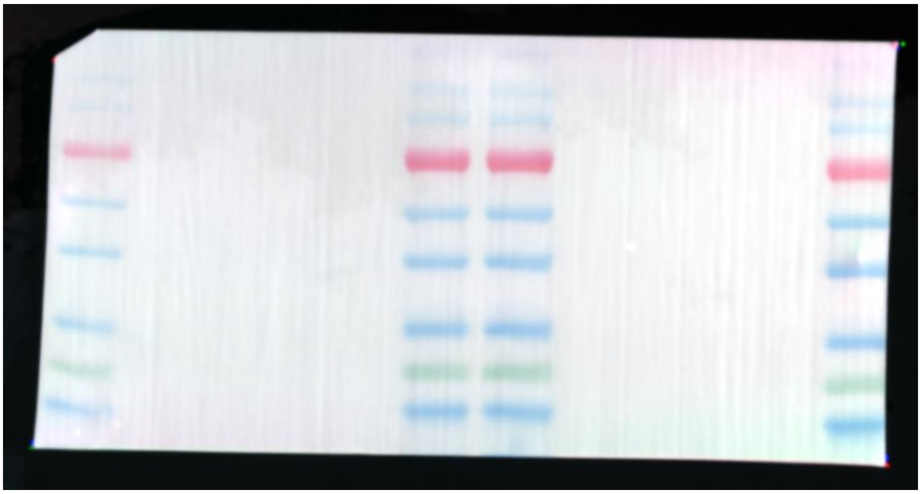

Repeat 3

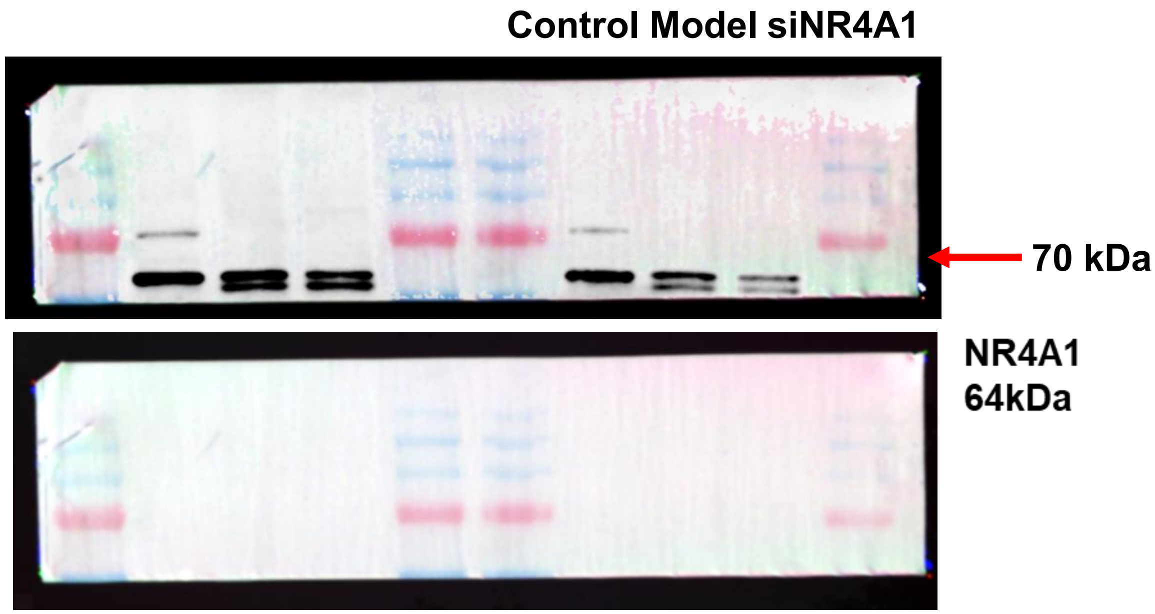

Repeat 3

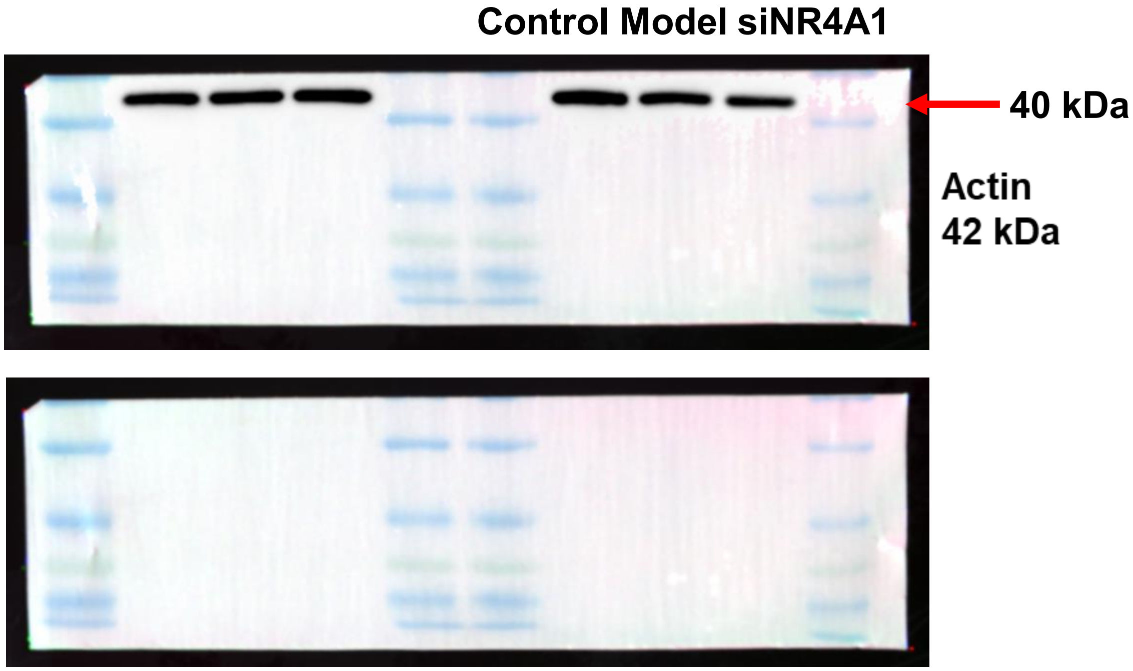

Repeat 3 The merged image

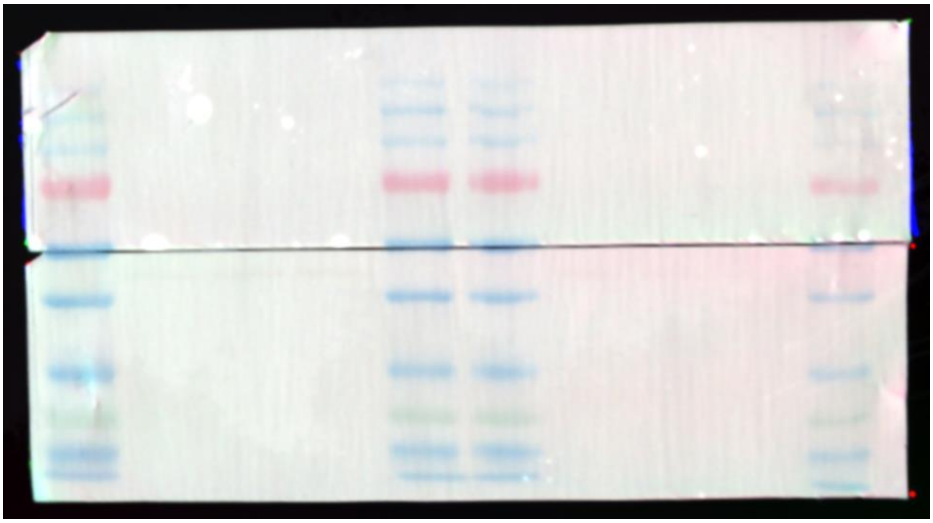

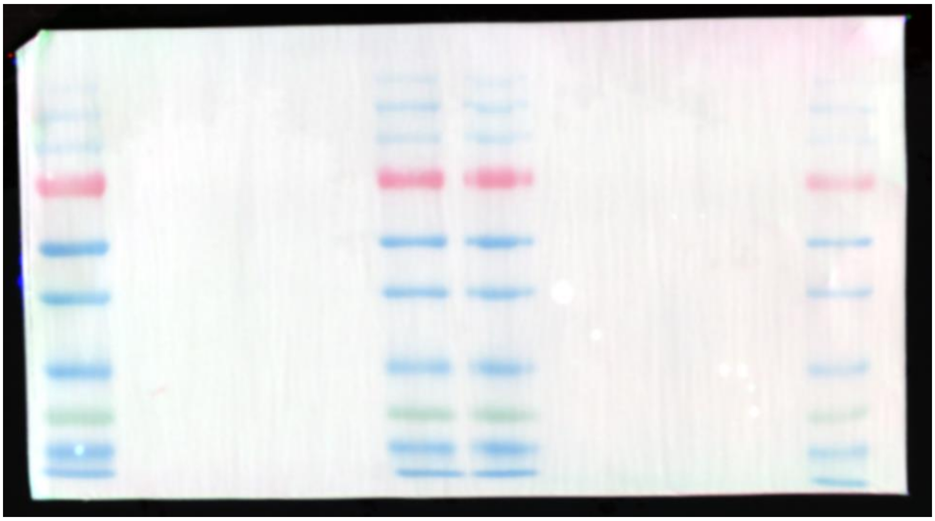

Western blot was used to analyze the effect of NR4A1 knockdown or p-NF-κB protein expression

Repeat 1

Control Model siNR4A1

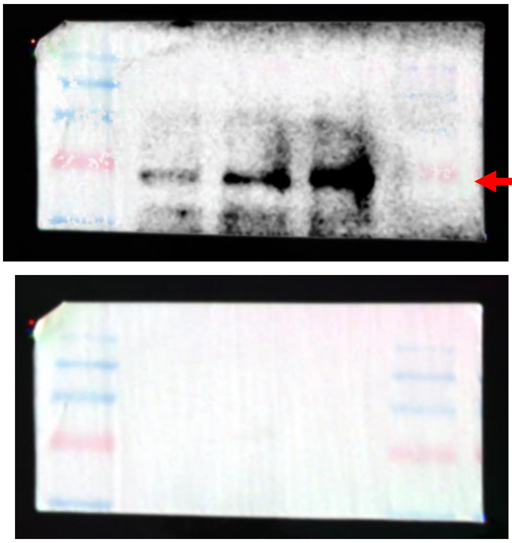

70 kDa

p-NF-κB 65kDa

Repeat 1

Control Model siNR4A1

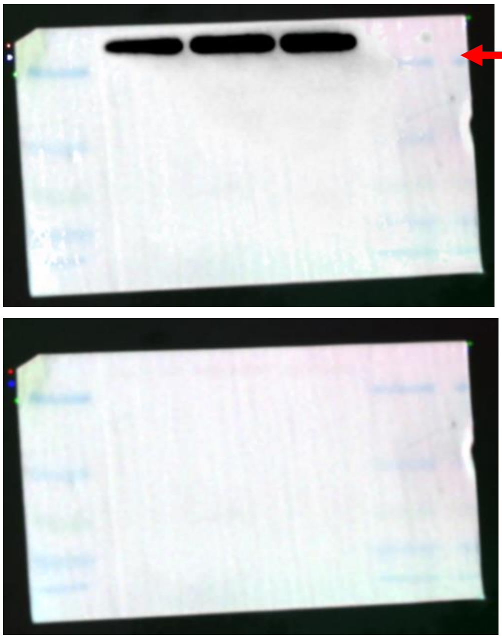

40 kDa

Actin 42 kDa

Repeat 1 The merged image

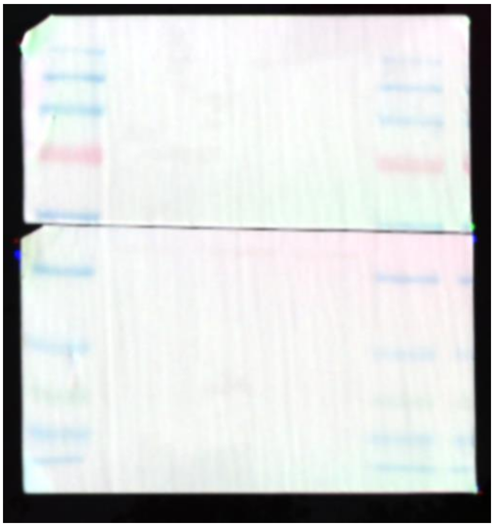

Repeat 2

Control Model siNR4A1

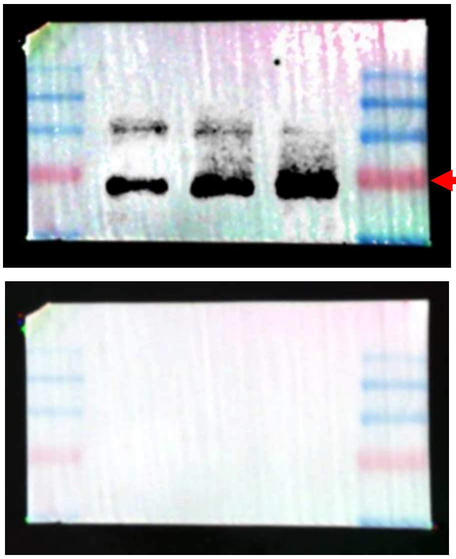

70 kDa

p-NF-κB
65kDa

Repeat 2

Control Model siNR4A1

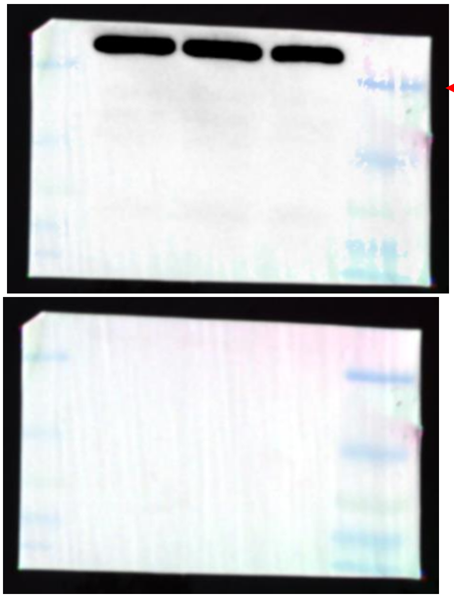

40 kDa

Actin
42 kDa

Repeat 2 The merged image

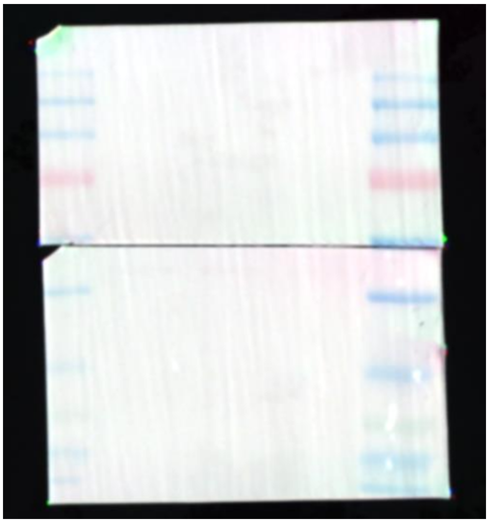

Repeat 1 and Repeat 2 ,the merged image

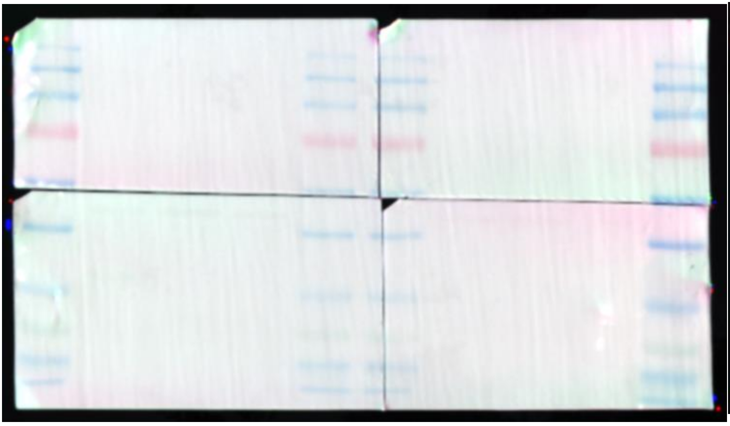

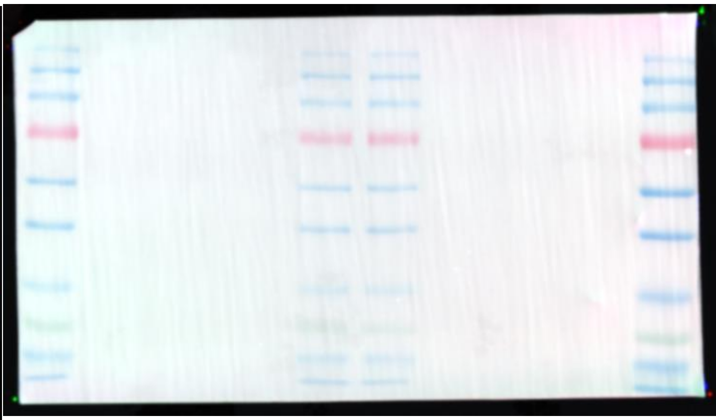

Repeat 3

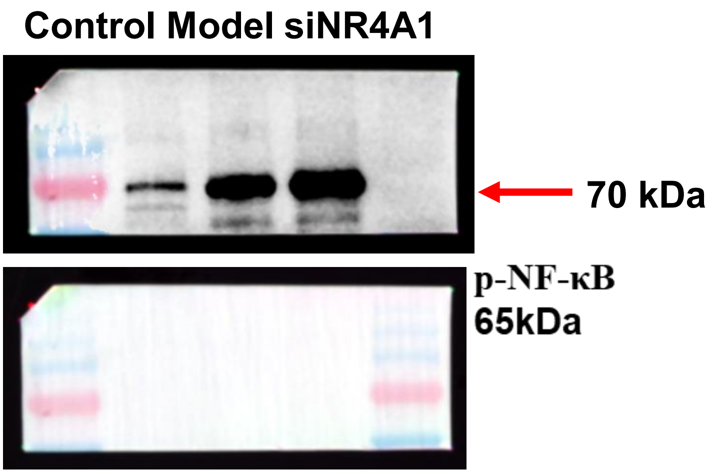

Repeat 3

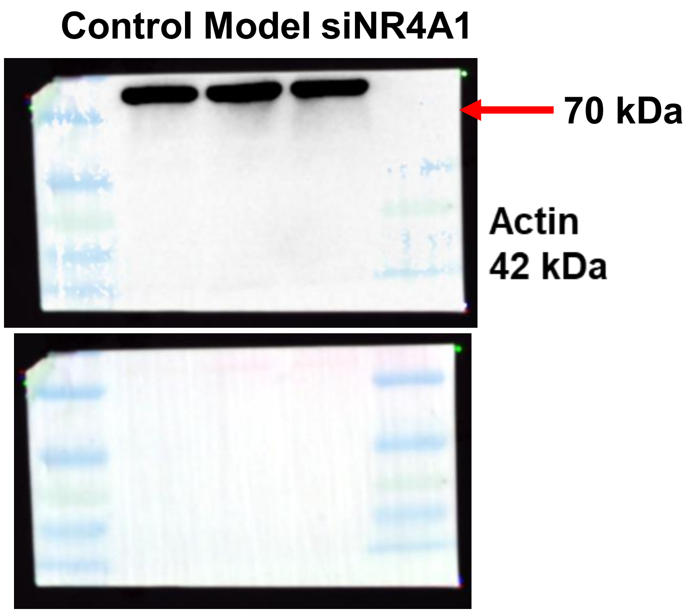

Repeat 3 The merged image

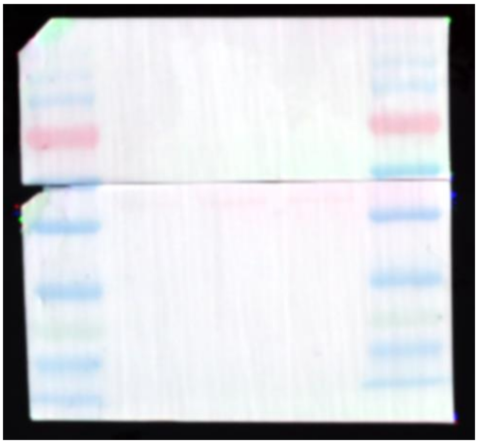

Repeat 4

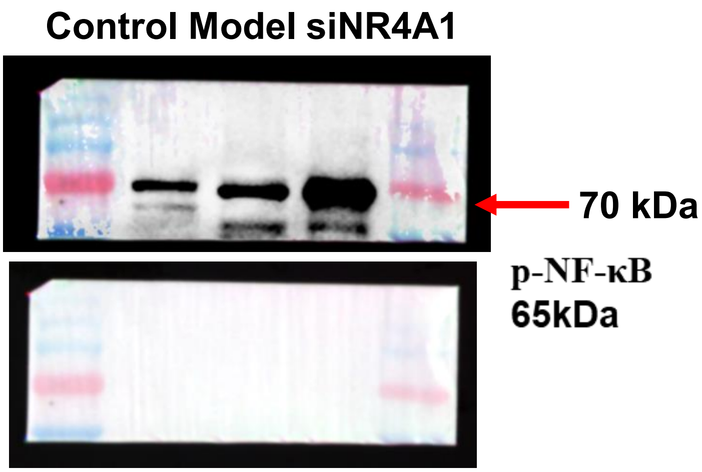

Repeat 4

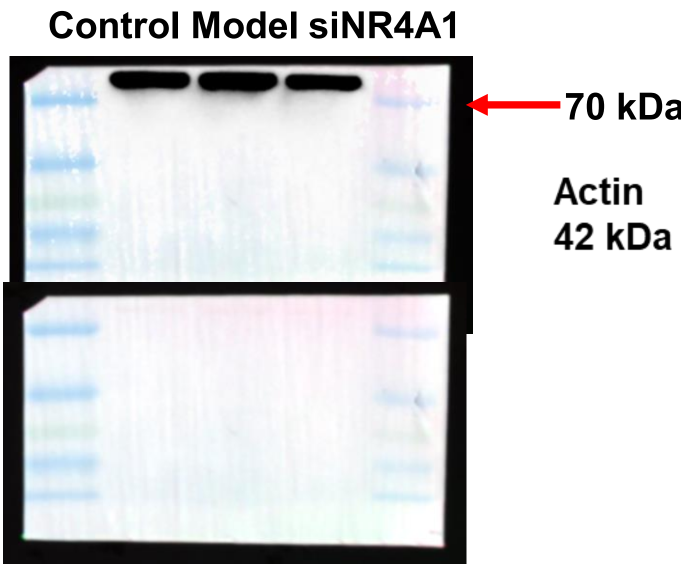

Repeat 4 The merged image

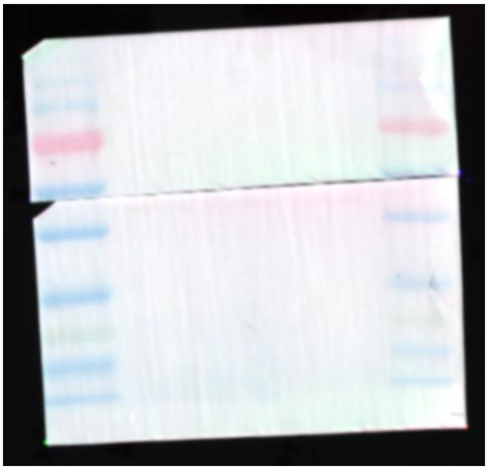

Repeat 3 and Repeat 4 ,the merged image

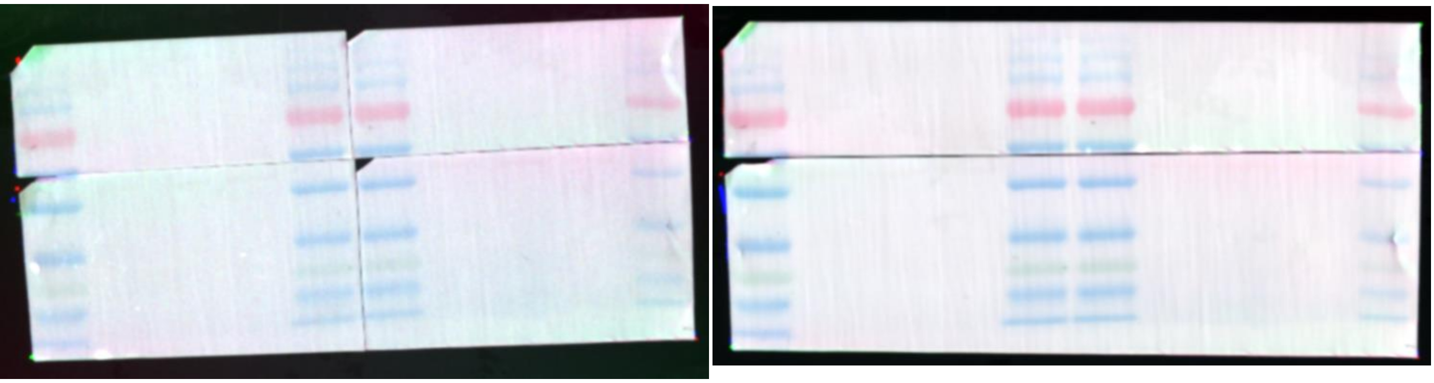

Western blot was used to analyze the effect of NR4A1 knockdown on p-NF-κB protein expression

Repeat 1

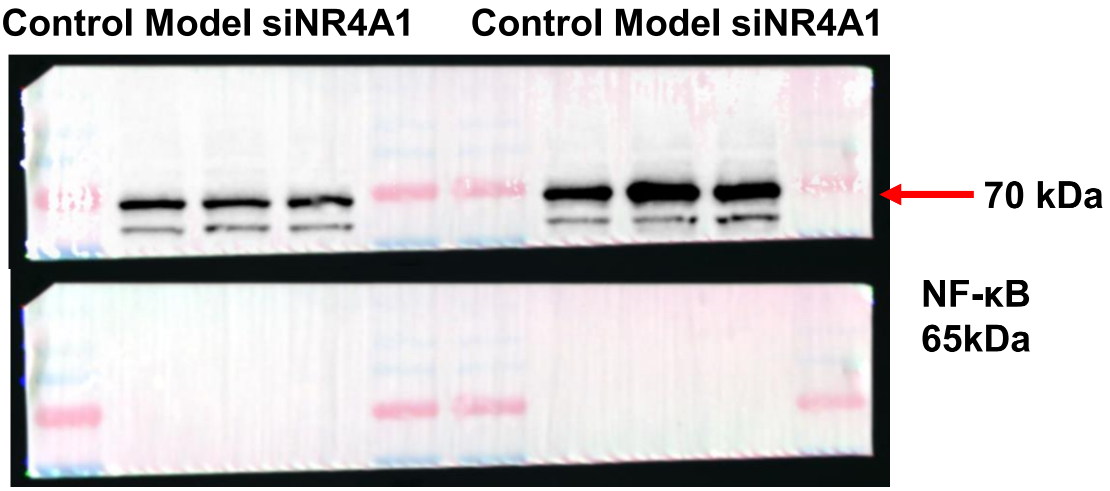

Repeat 1

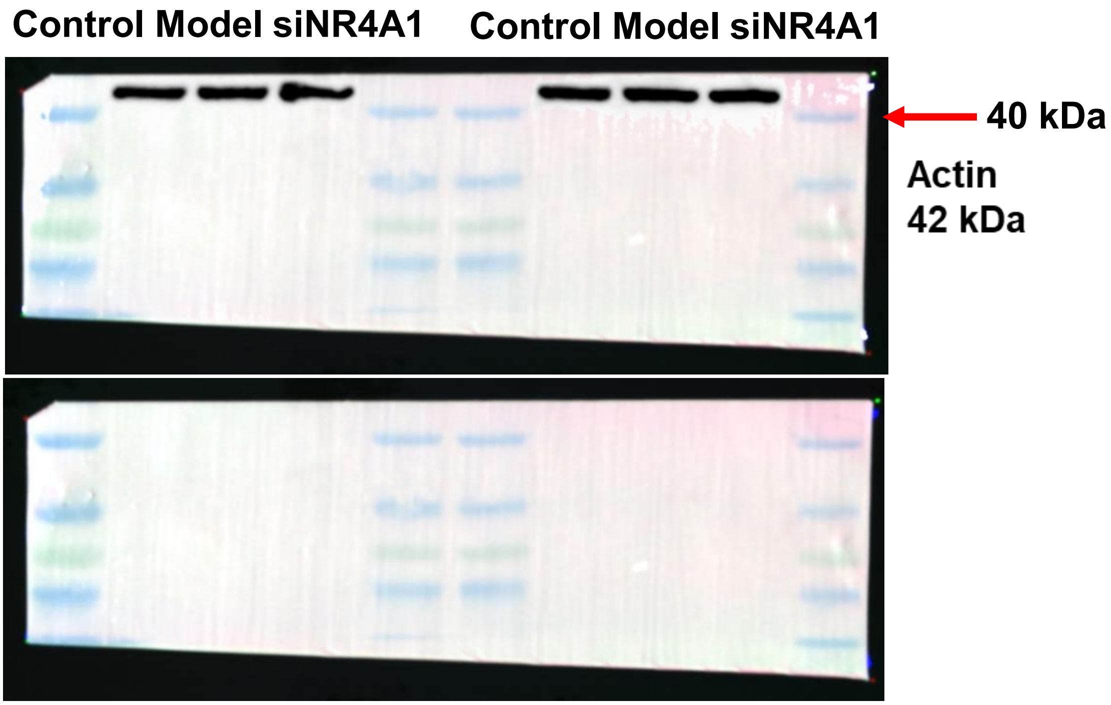

Repeat 1 ,the merged image

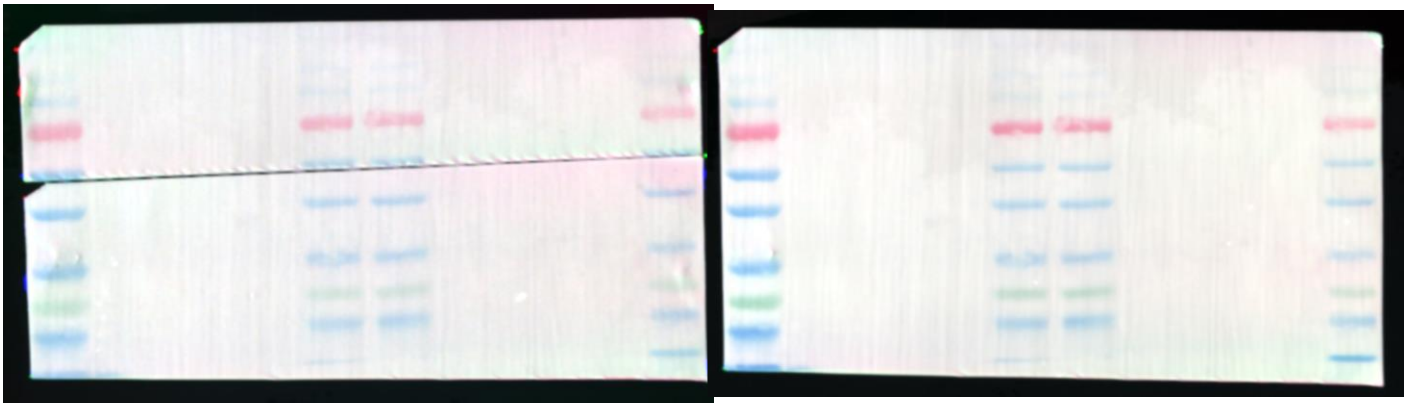

Repeat 2

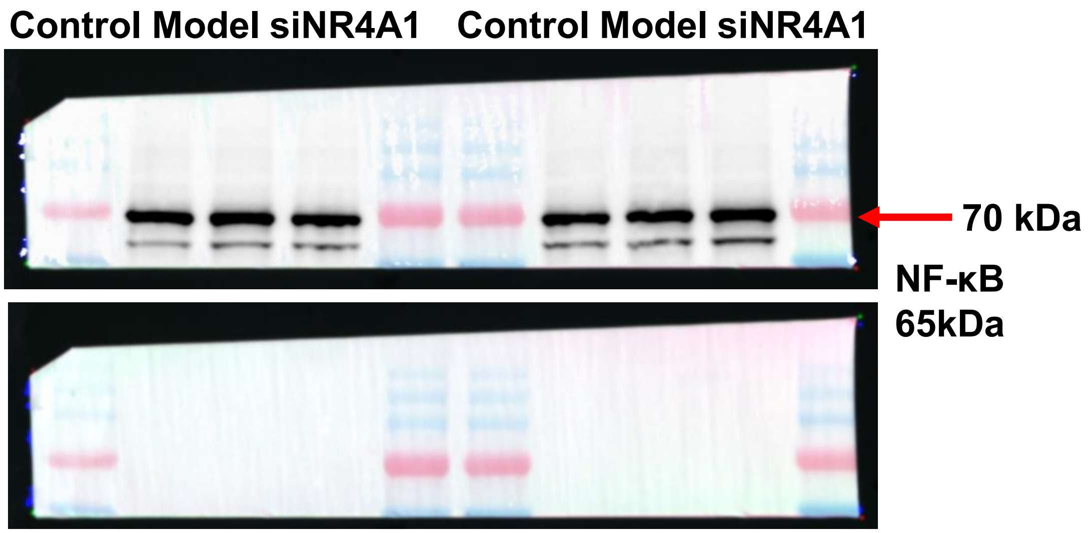

Repeat 2

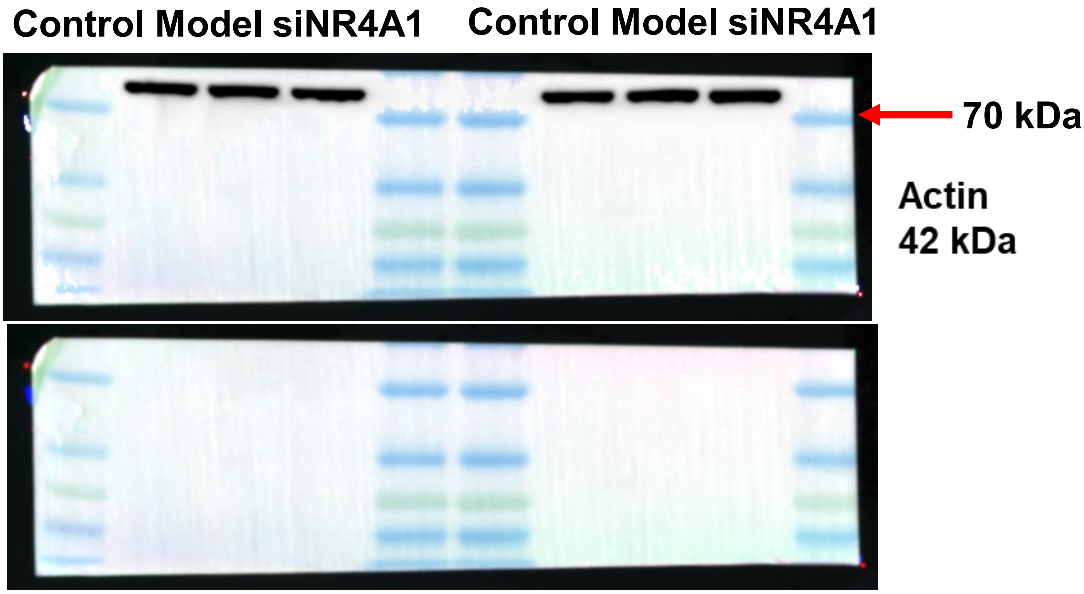

Repeat 2 ,the merged image

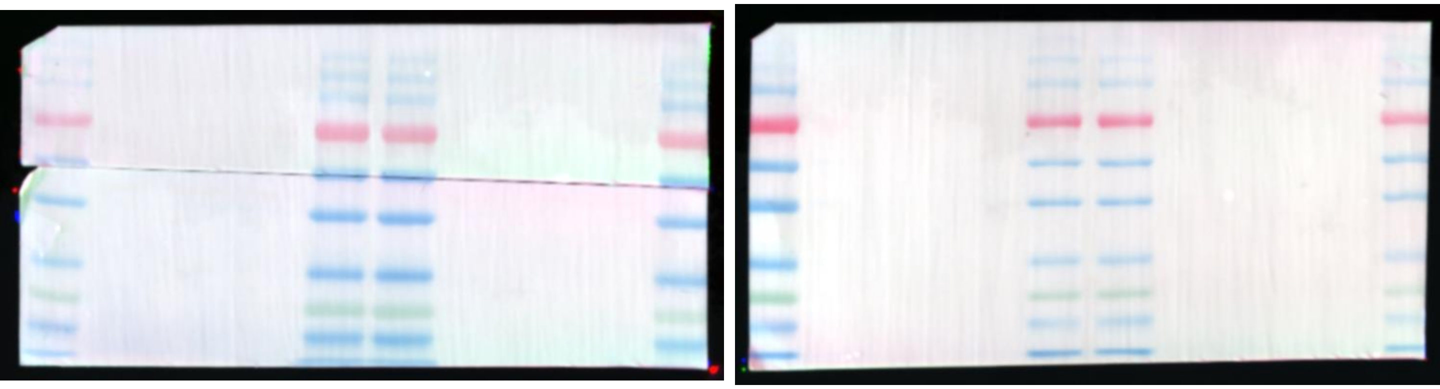

Repeat 3

Control Model siNR4A1

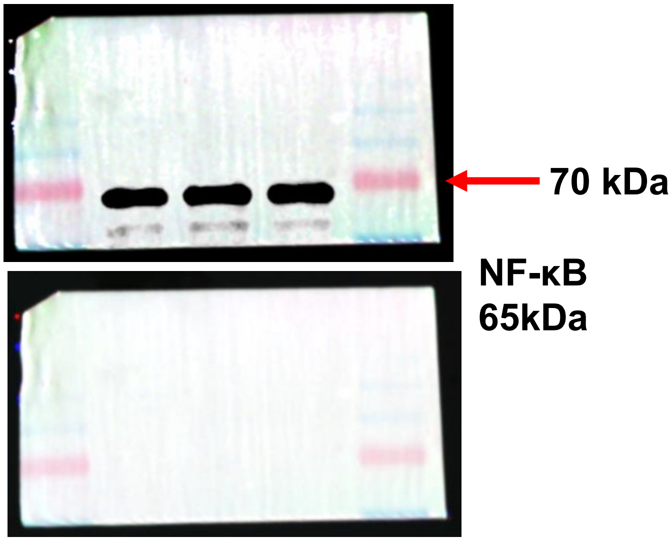

70 kDa

NF-κB
65kDa

Control Model siNR4A1

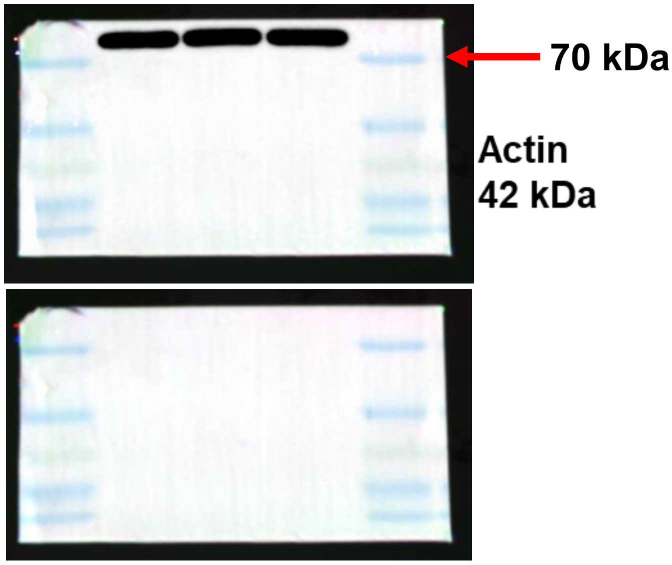

70 kDa

Actin
42 kDa

Repeat 3, the merged image

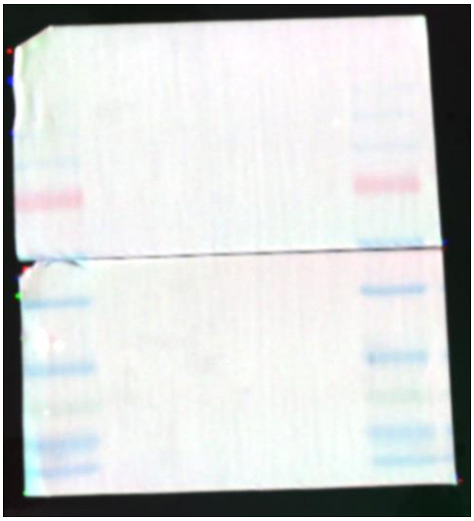

Repeat 4

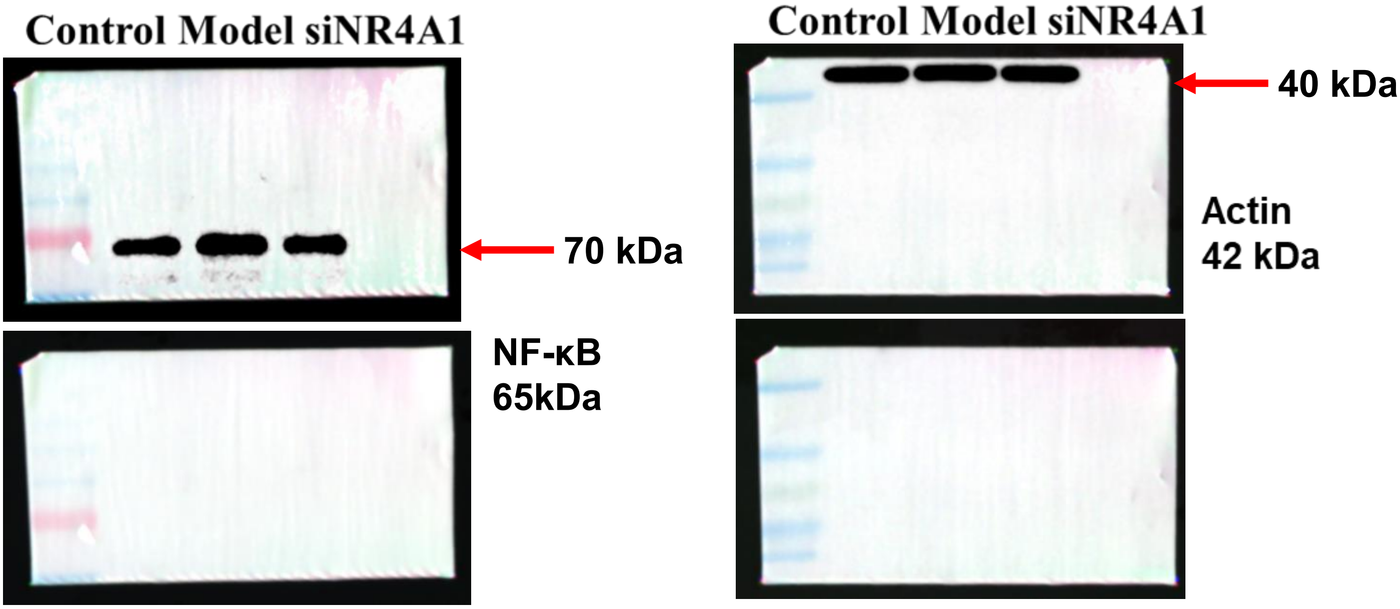

Repeat 4, the merged image

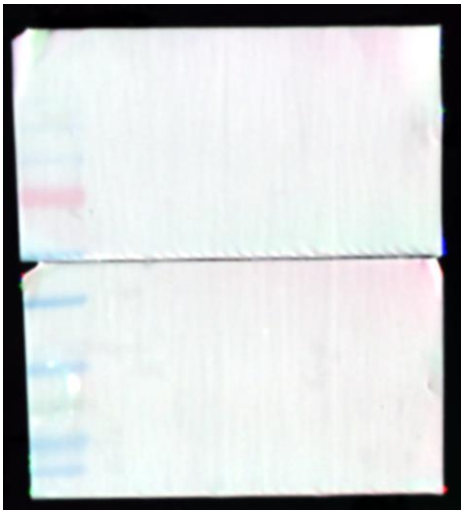

Repeat 3 and Repeat 4, the merged image

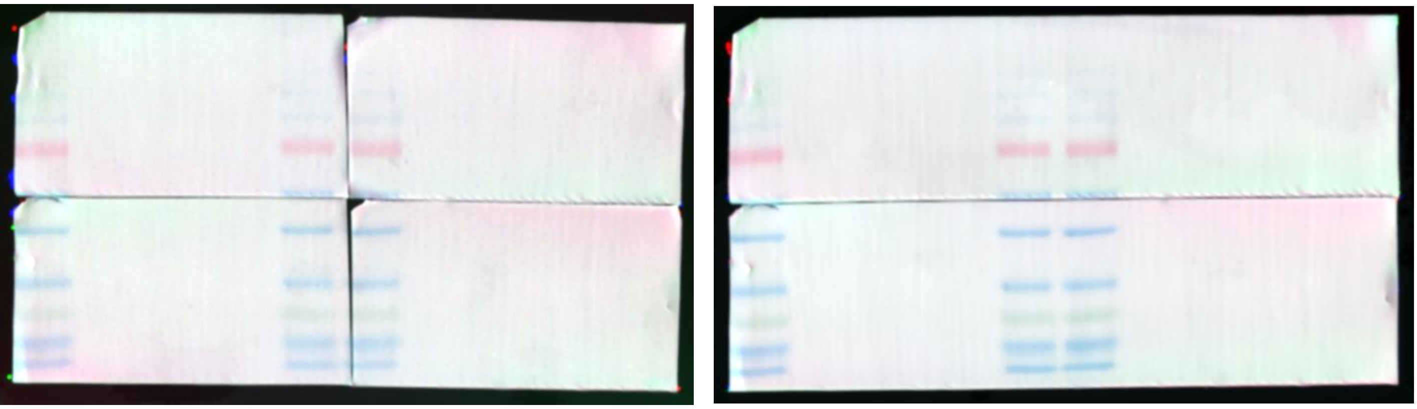

Western blot was used to analyze the effect of an NR4A1 overexpression plasmid on NR4A1 protein *in vitro*

Repeat 1

Control Vector pNR4A1 Control Vector pNR4A1

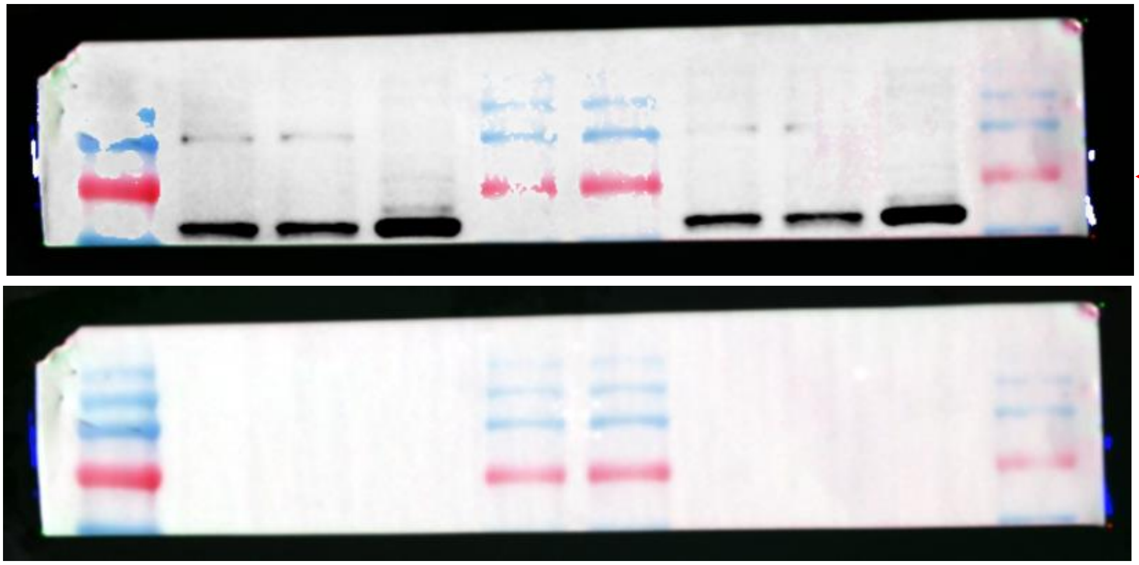

70 kDa

NR4A1
64kDa

Repeat 1

Control Vector pNR4A1 Control Vector pNR4A1

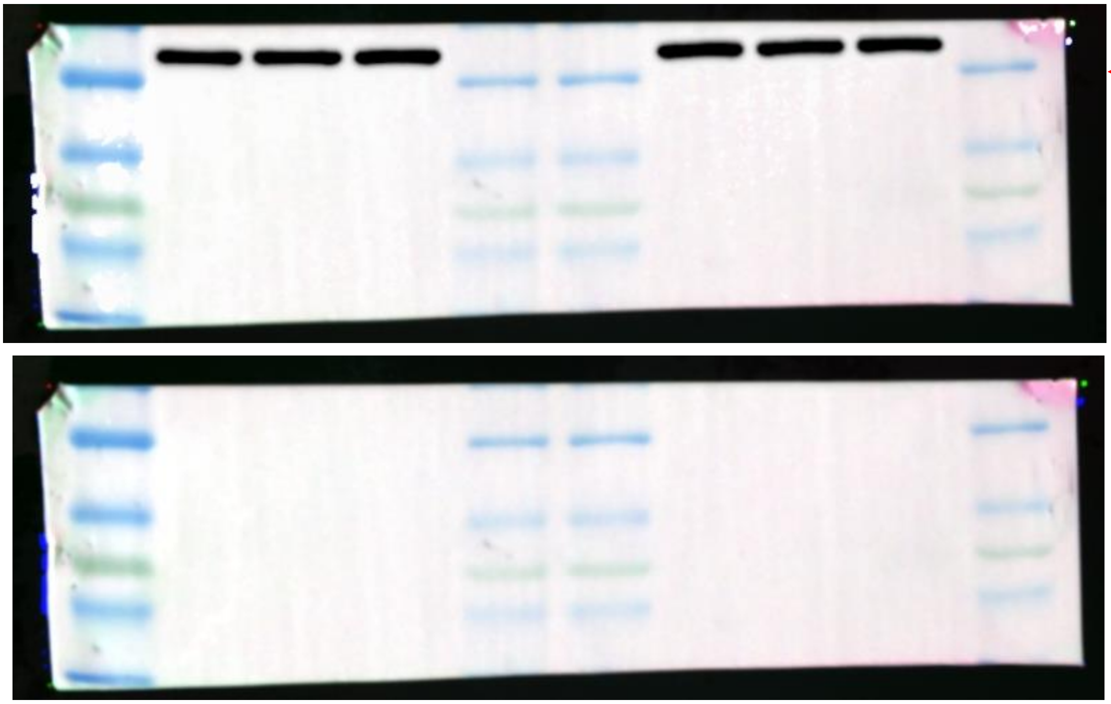

40 kDa

Actin
42kDa

Repeat 1 The merged image

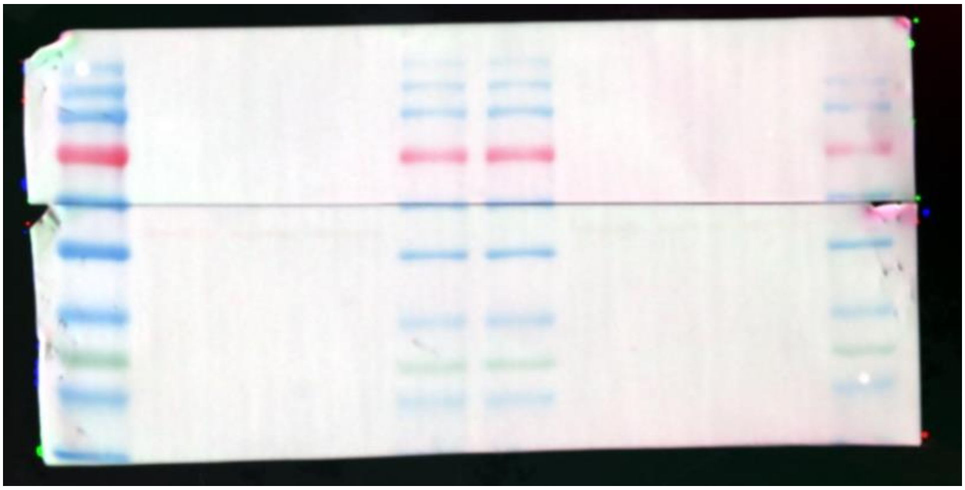

Repeat 2

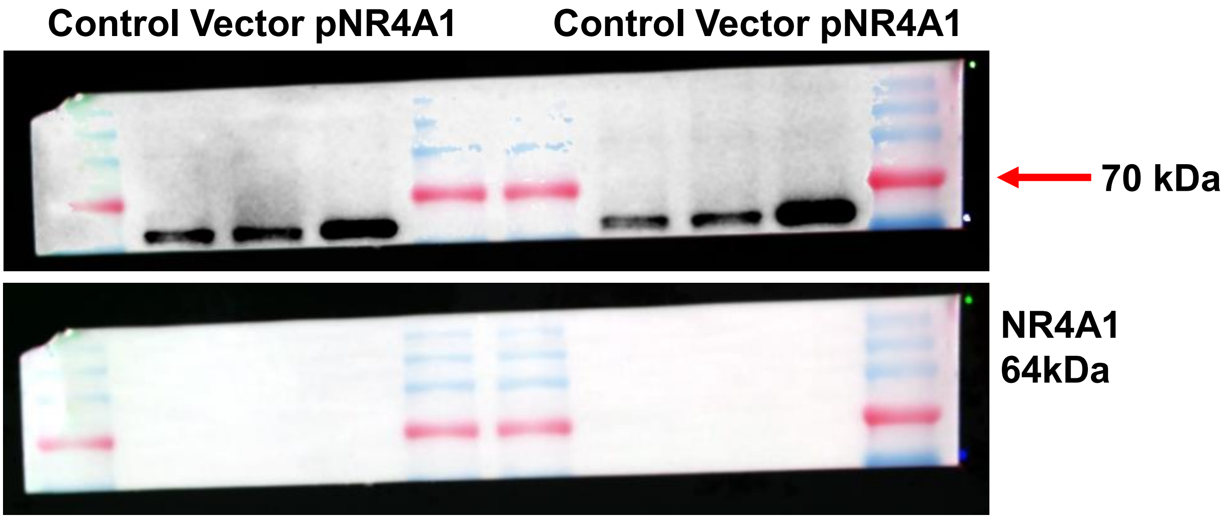

Repeat 2

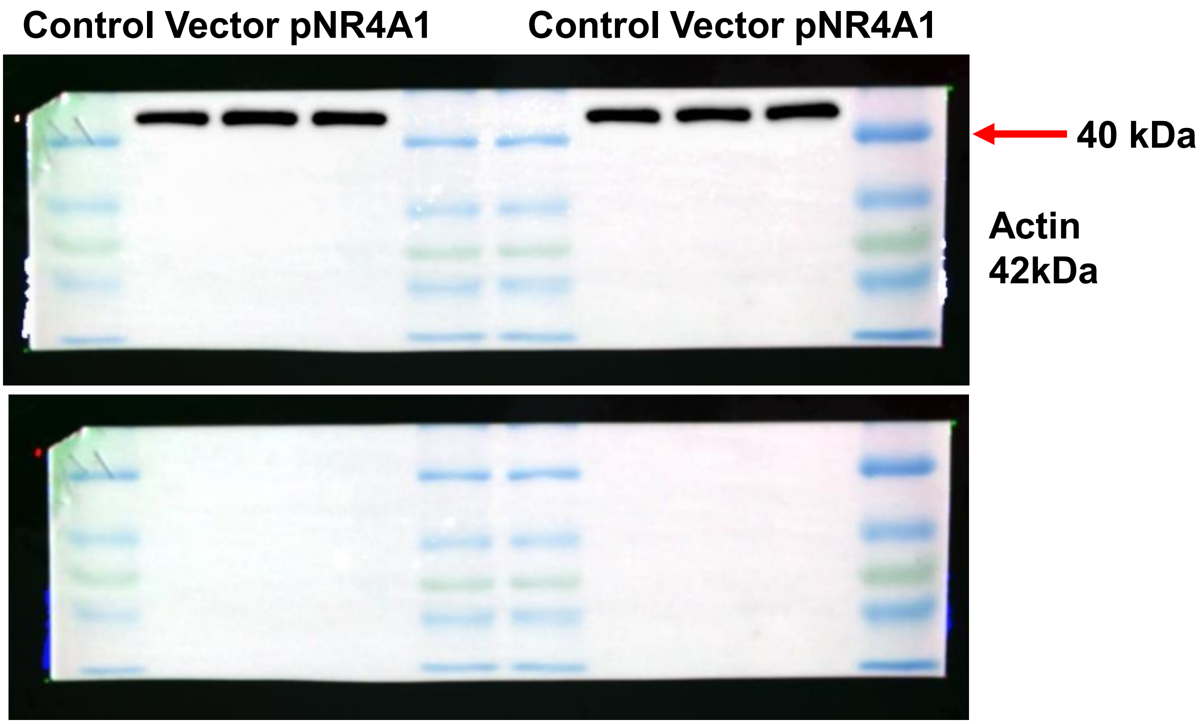

Repeat 2 ,the merged image

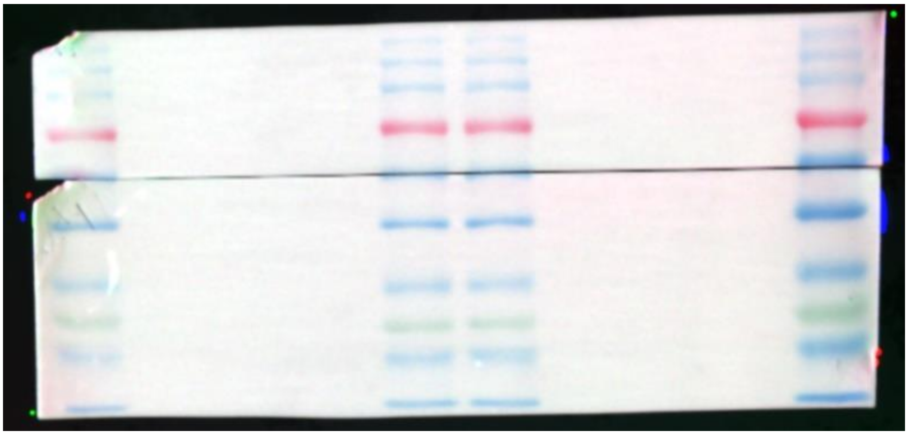

Repeat 3

Control Vector pNR4A1 Control Vector pNR4A1

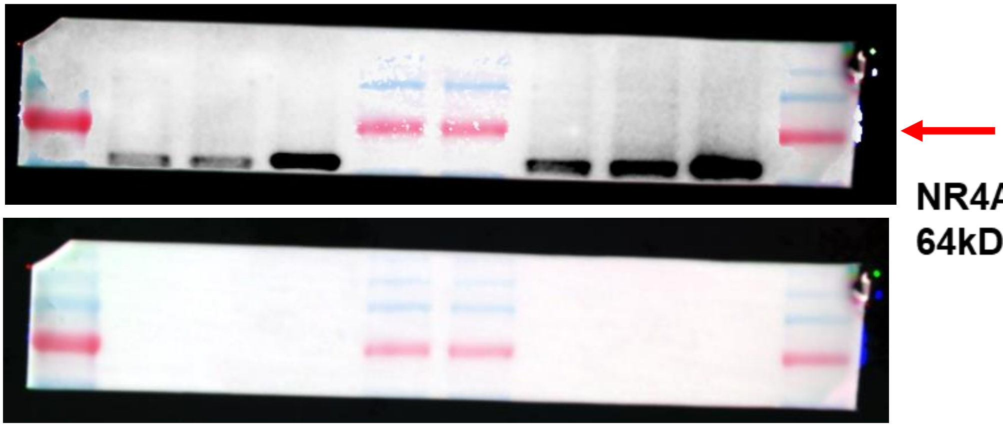

70 kDa

NR4A1 64kDa

Repeat 3

Control Vector pNR4A1 Control Vector pNR4A1

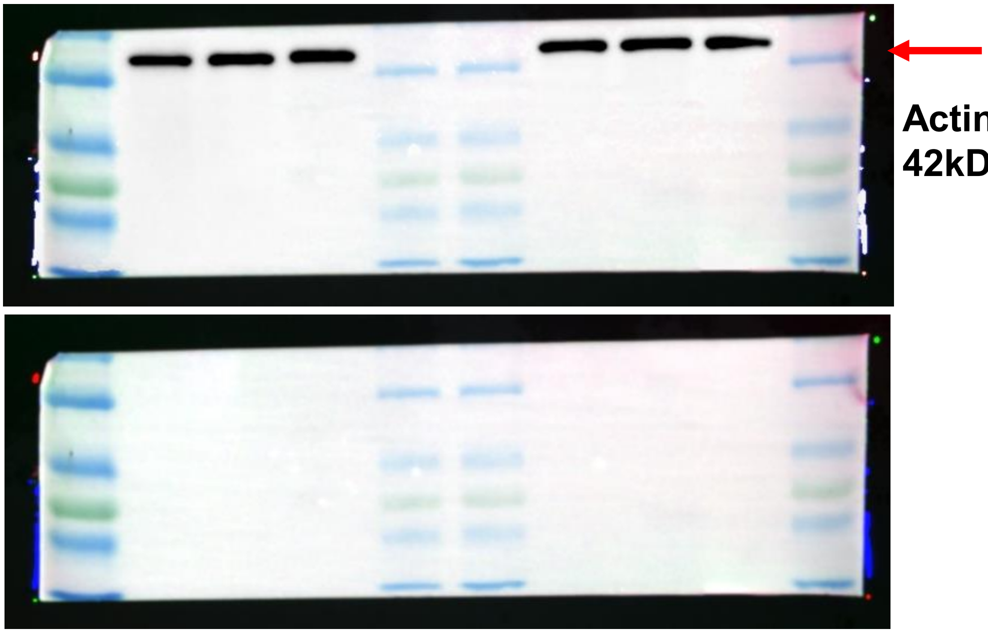

40 kDa

Actin 42kDa

Repeat 3, the merged image

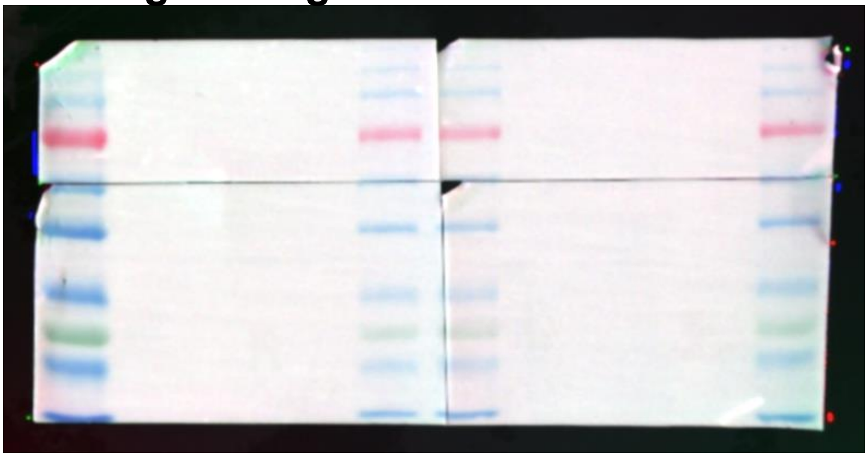

Western blot was used to analyze the effect of an NR4A1 overexpression plasmid on NR4A1 protein expression under model conditions *in vitro*

Repeat 1

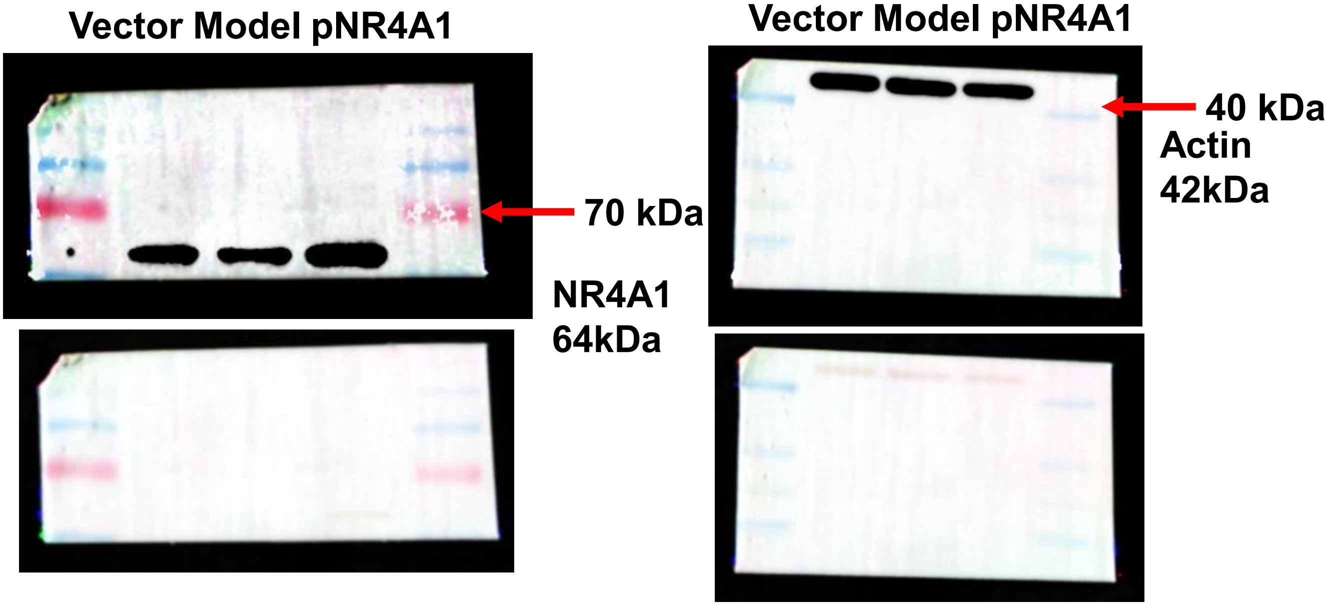

Repeat 1 The merged image

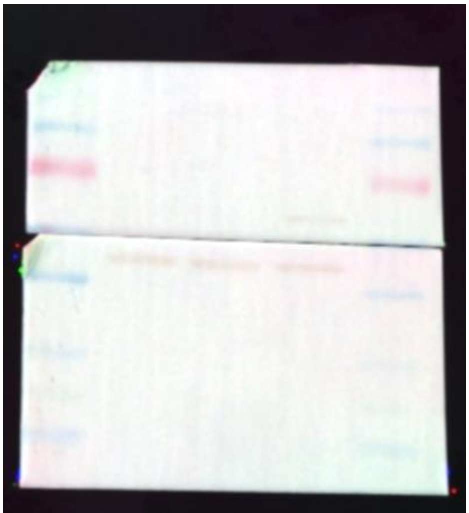

Repeat 2

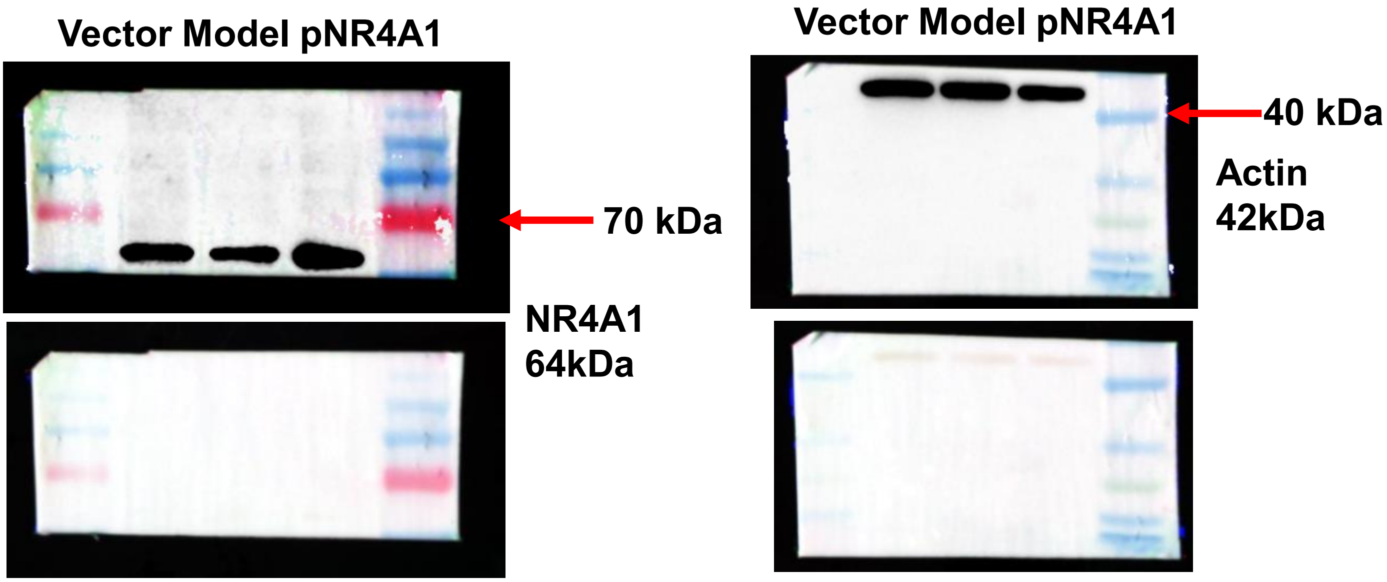

Repeat 2 The merged image

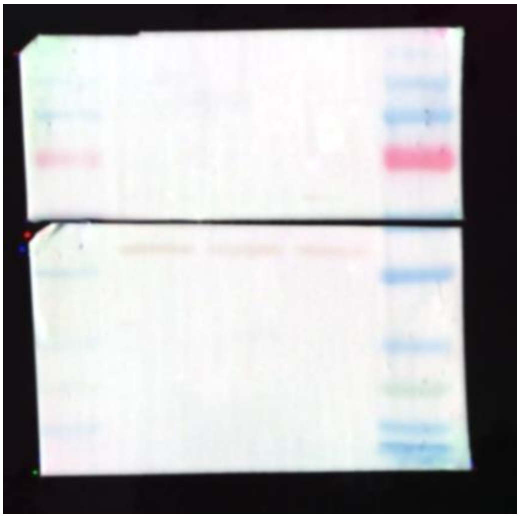

Repeat 1 and Repeat 2 ,the merged image

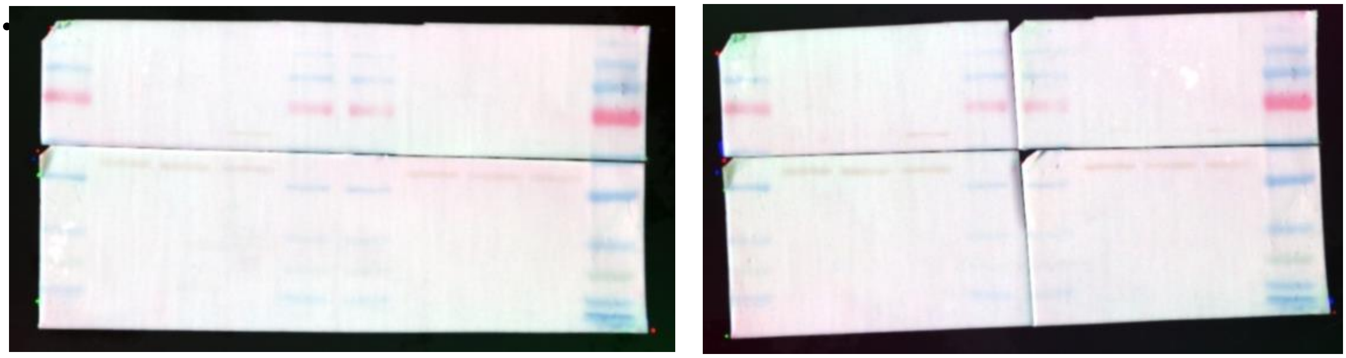

Repeat 3

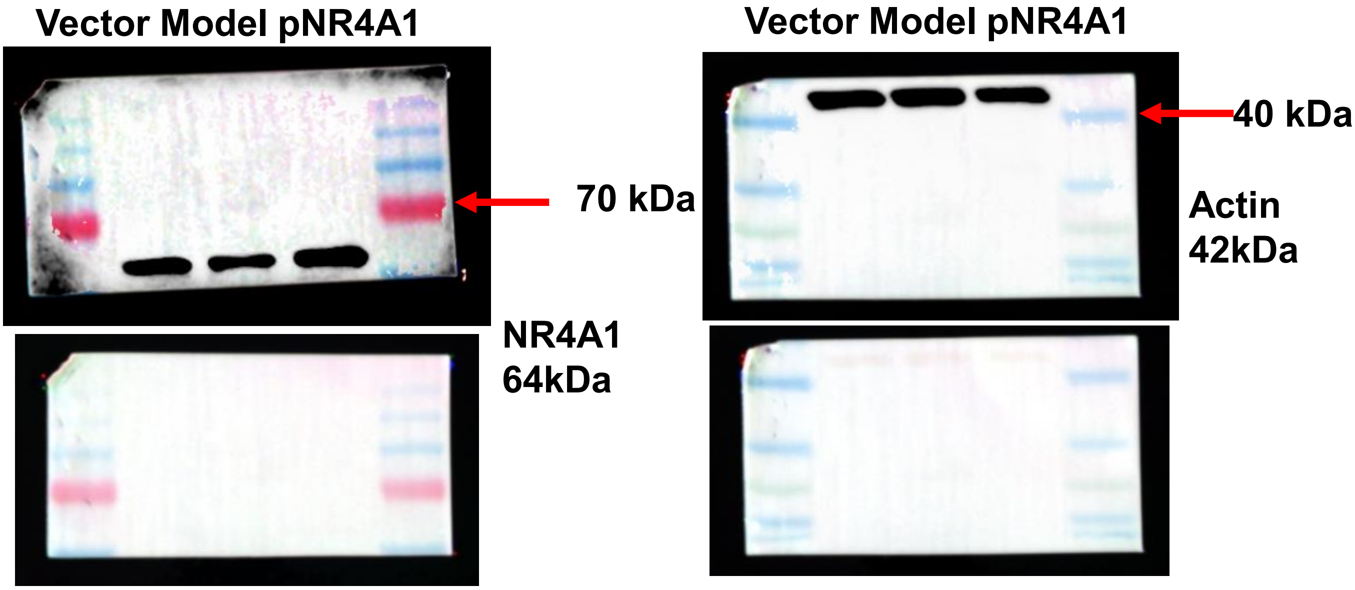

Repeat 3 ,the merged image

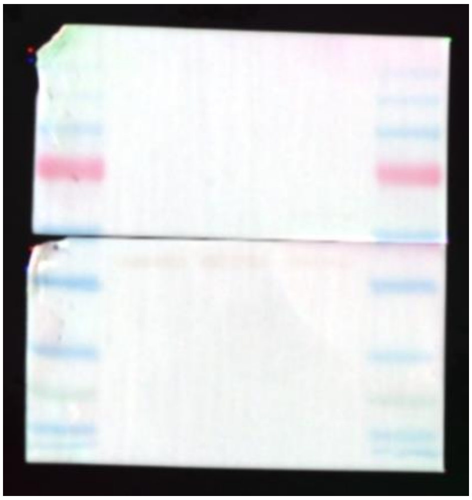

Repeat 4

Vector Model pNR4A1

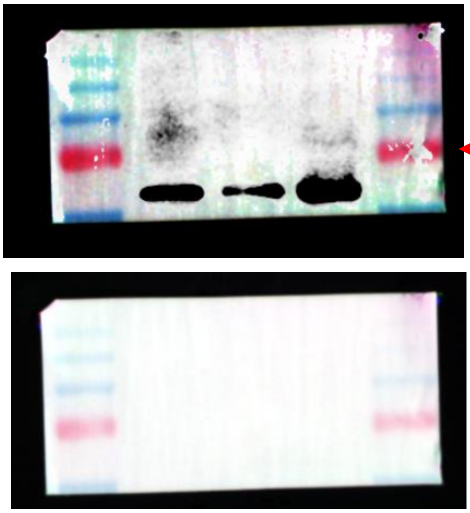

70 kDa

NR4A1
64kDa

Vector Model pNR4A1

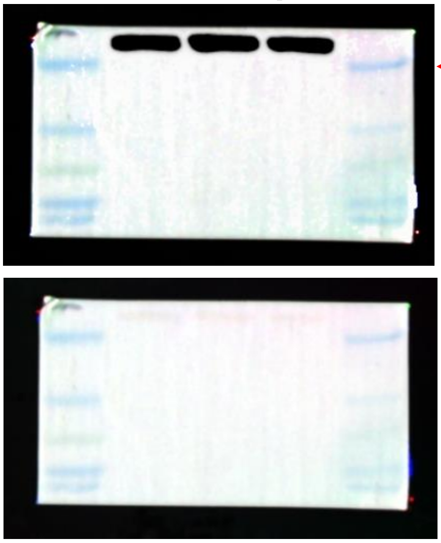

40 kDa

Actin
42kDa

Repeat 4 ,the merged image

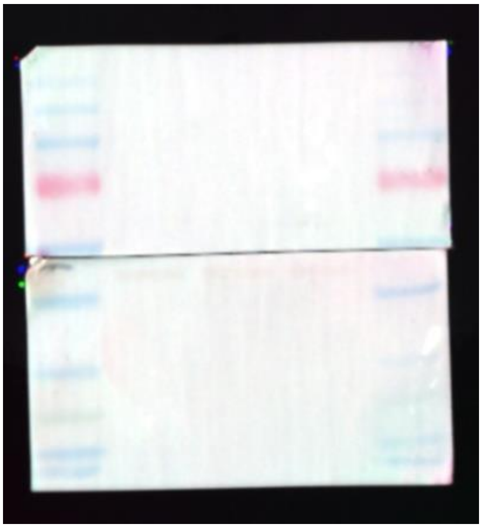

Repeat 3 and Repeat 4 ,the merged image

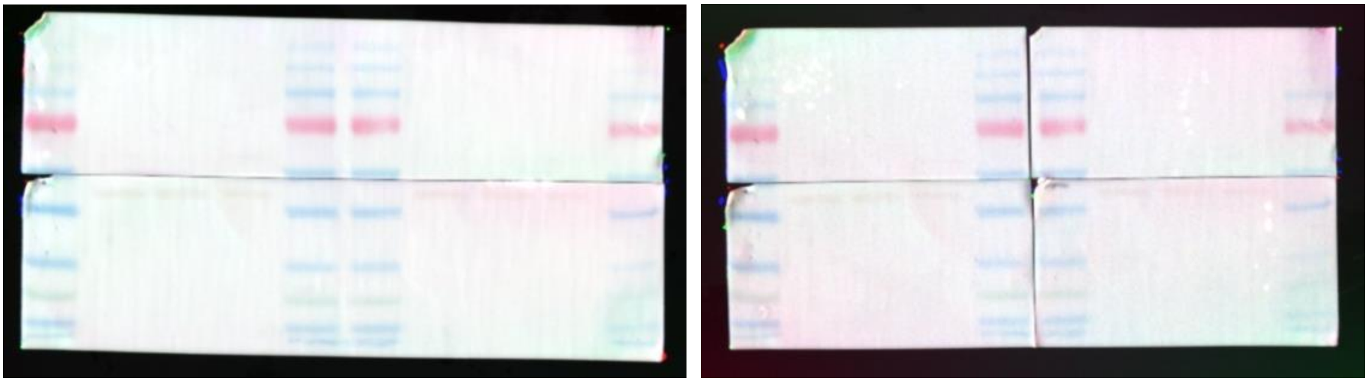

Western blot was used to analyze the effects of the NR4A1 overexpression plasmid on p-NF-κB under model conditions *in vitro*

Repeat 1

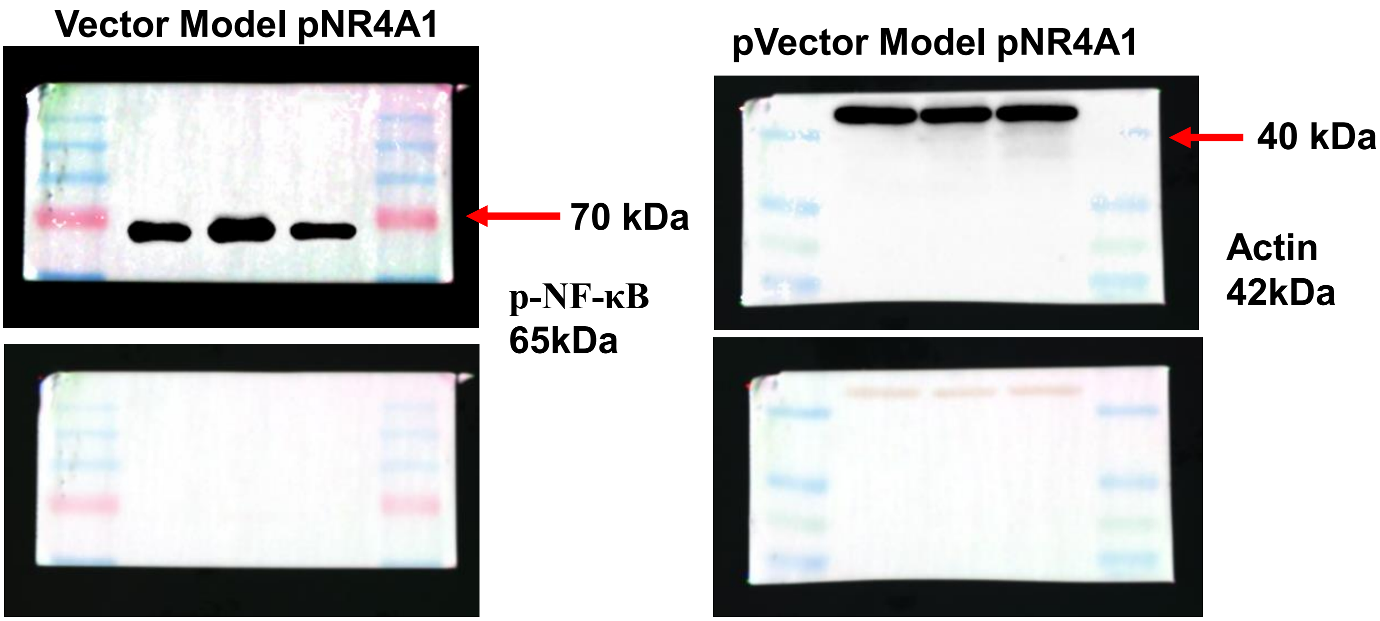

Repeat 1 ,the merged image

•

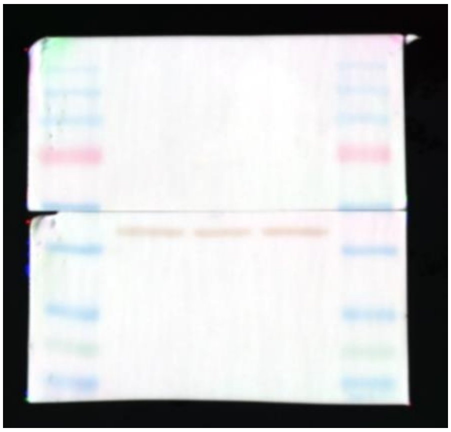

Repeat 2

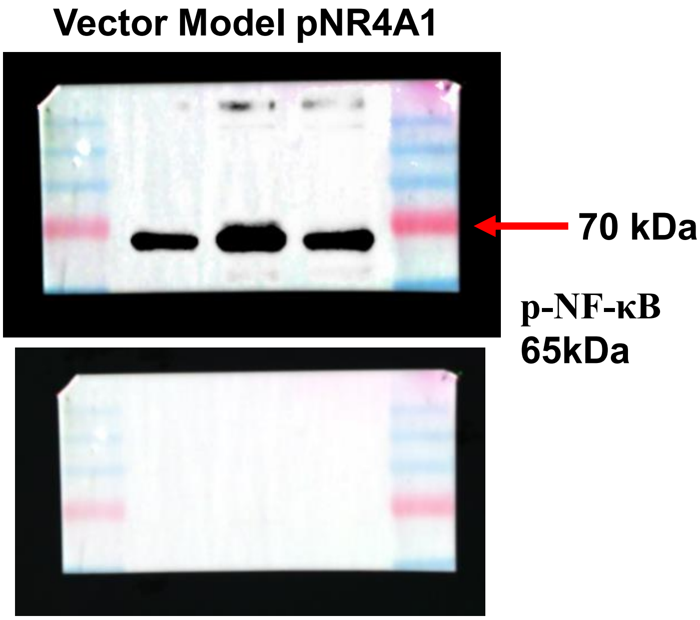

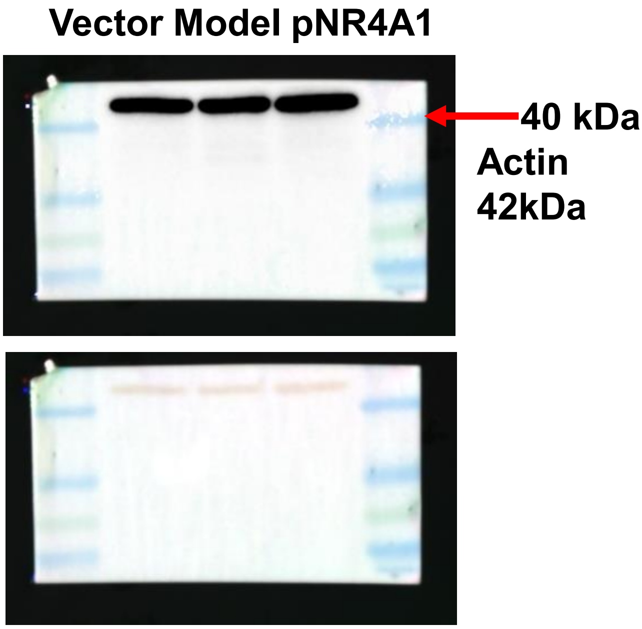

Repeat 2, the merged image

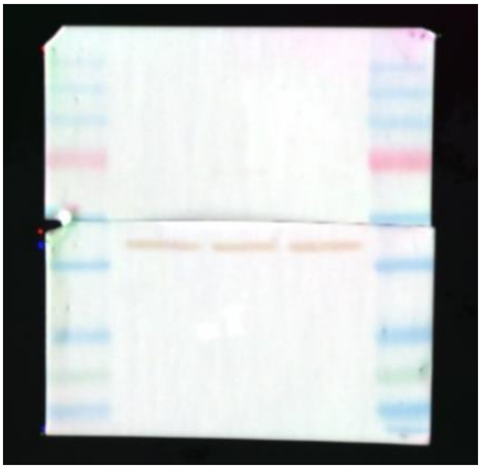

Repeat 1 and Repeat 2 , the merged image

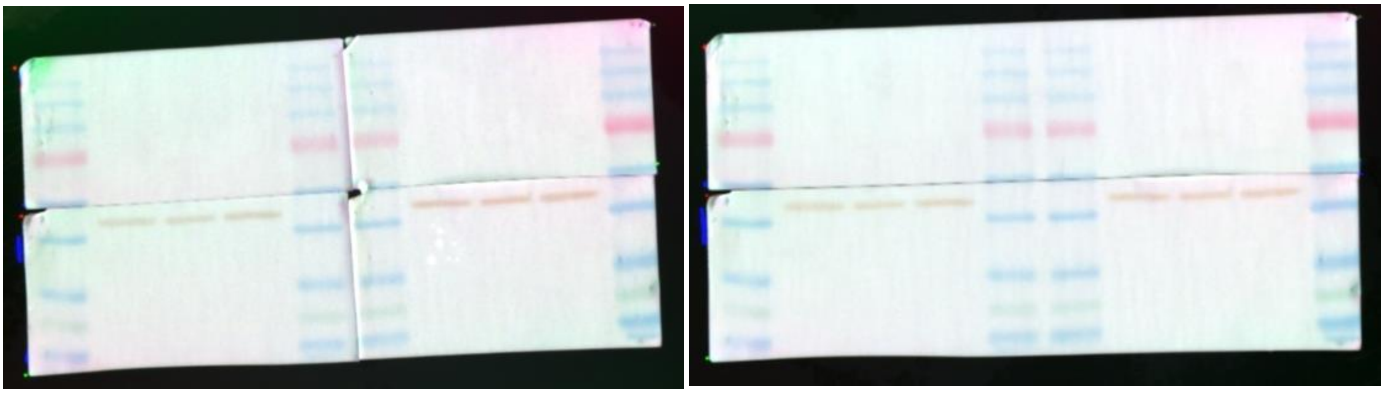

Repeat 3

Vector Model pNR4A1

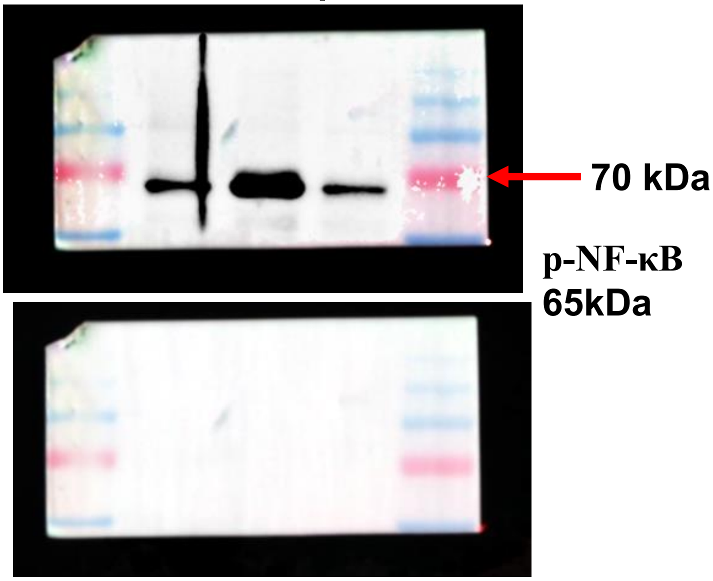

70 kDa

p-NF-κB
65kDa

Vector Model pNR4A1

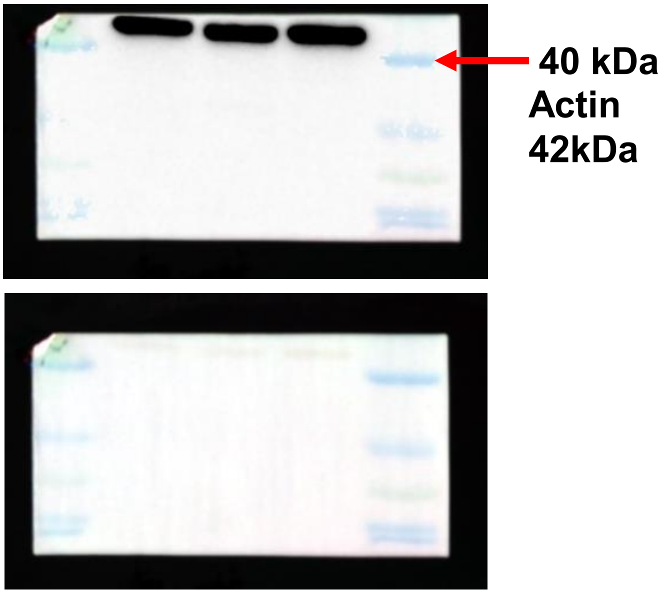

40 kDa
Actin
42kDa

Repeat 3 ,the merged image

•

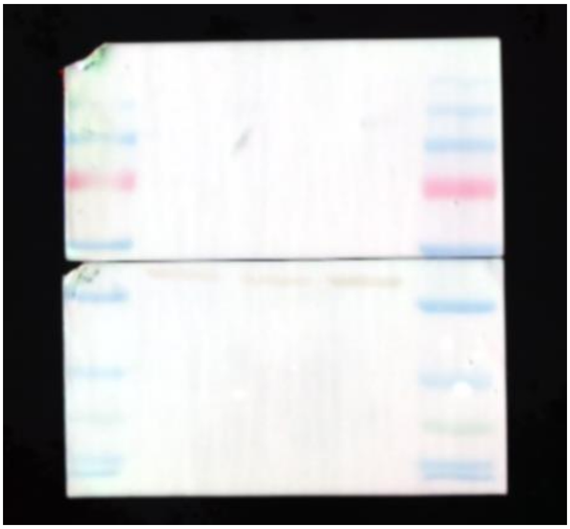

Repeat 4

Vector Model pNR4A1

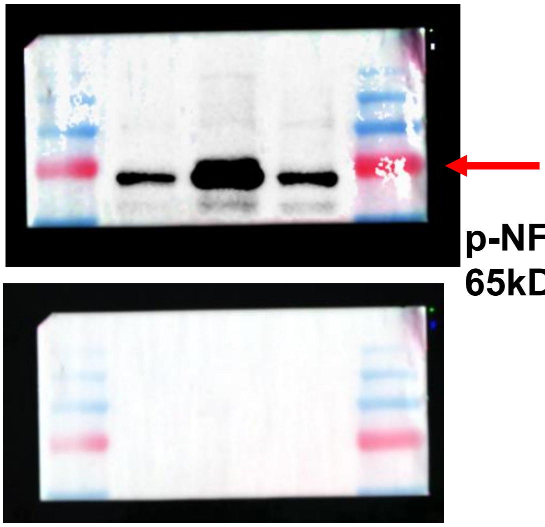

70 kDa

p-NF-κB 65kDa

Vector Model pNR4A1

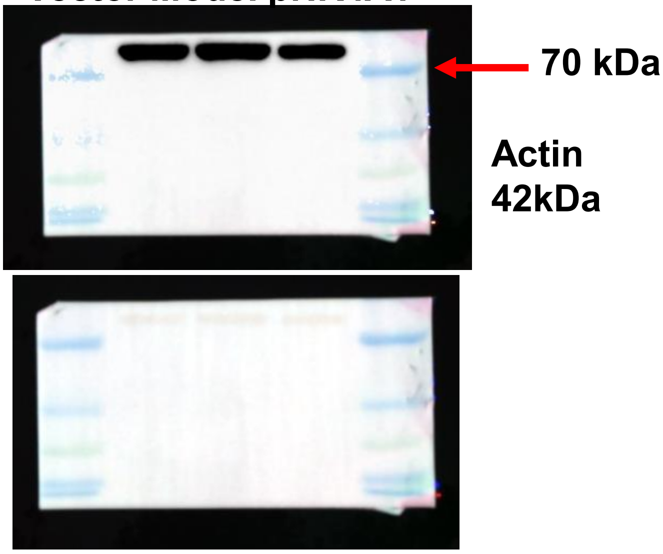

70 kDa

Actin 42kDa

Repeat 4, the merged image

•

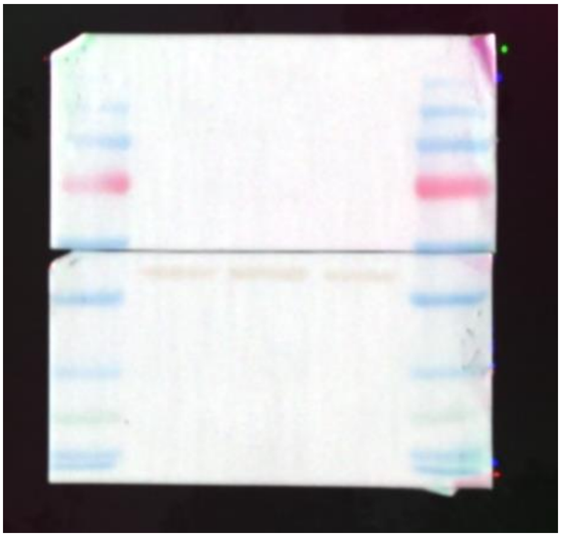

Repeat 3 and Repeat 4 , the merged image

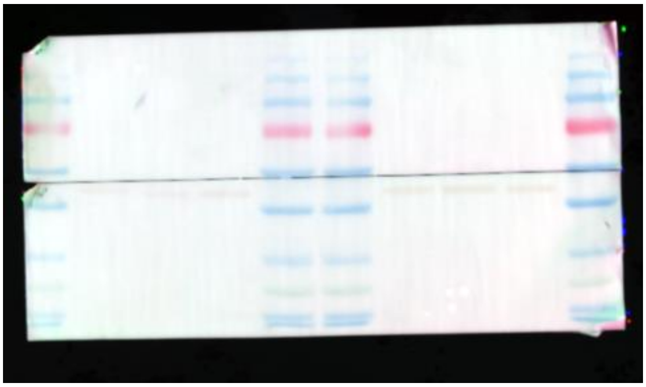

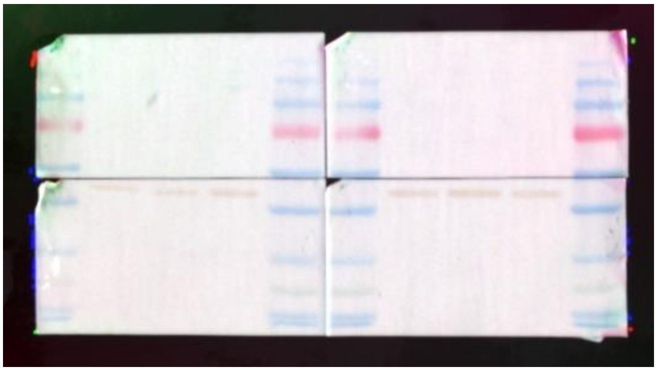

Western blot was used to analyze the effects of the NR4A1 overexpression plasmid on NF-κB under model conditions *in vitro*

Repeat 1

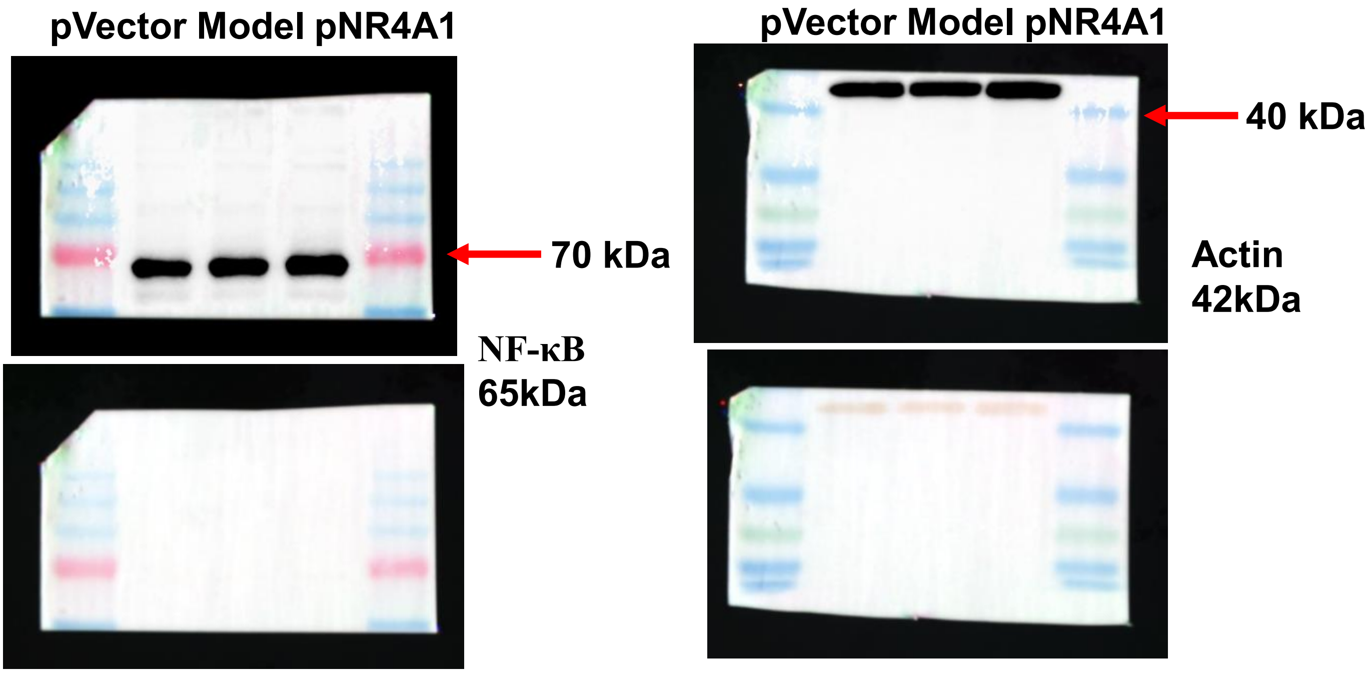

Repeat 1, the merged image

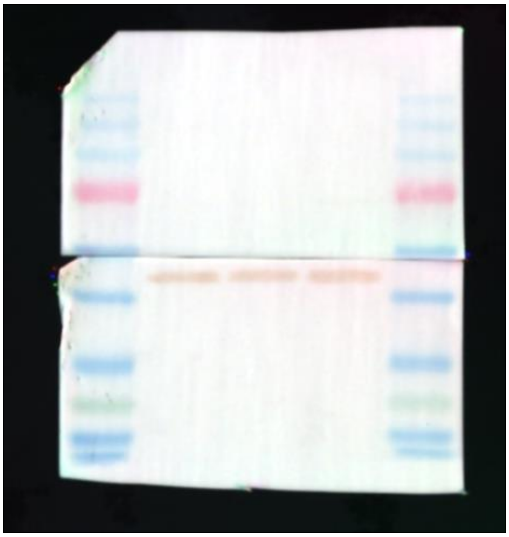

Repeat 2

Vector Model pNR4A1

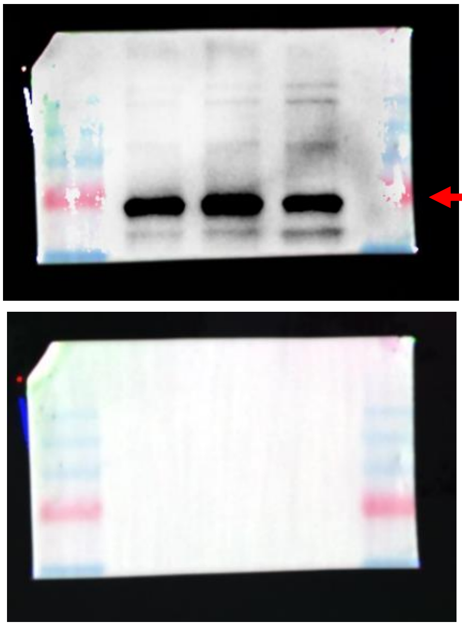

70 kDa

NF-κB 65kDa

Vector Model pNR4A1

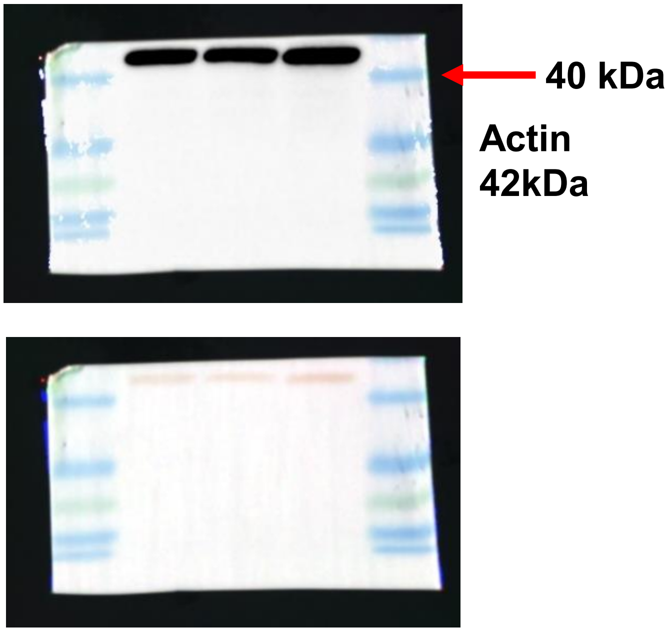

40 kDa

Actin 42kDa

Repeat 2, the merged image

•

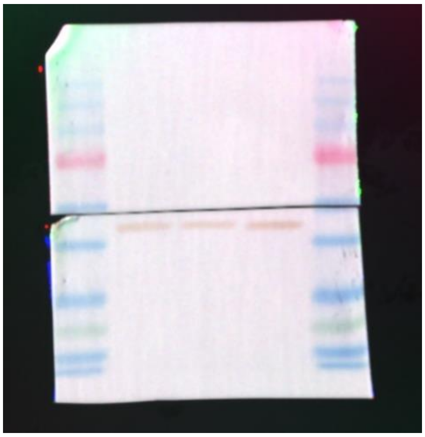

Repeat 1 and Repeat 2, the merged image

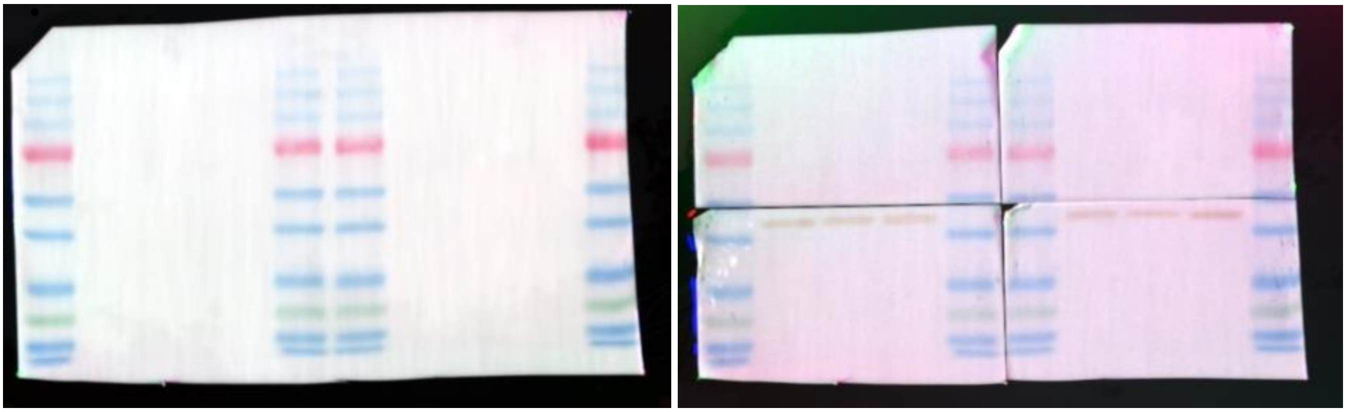

Repeat 3

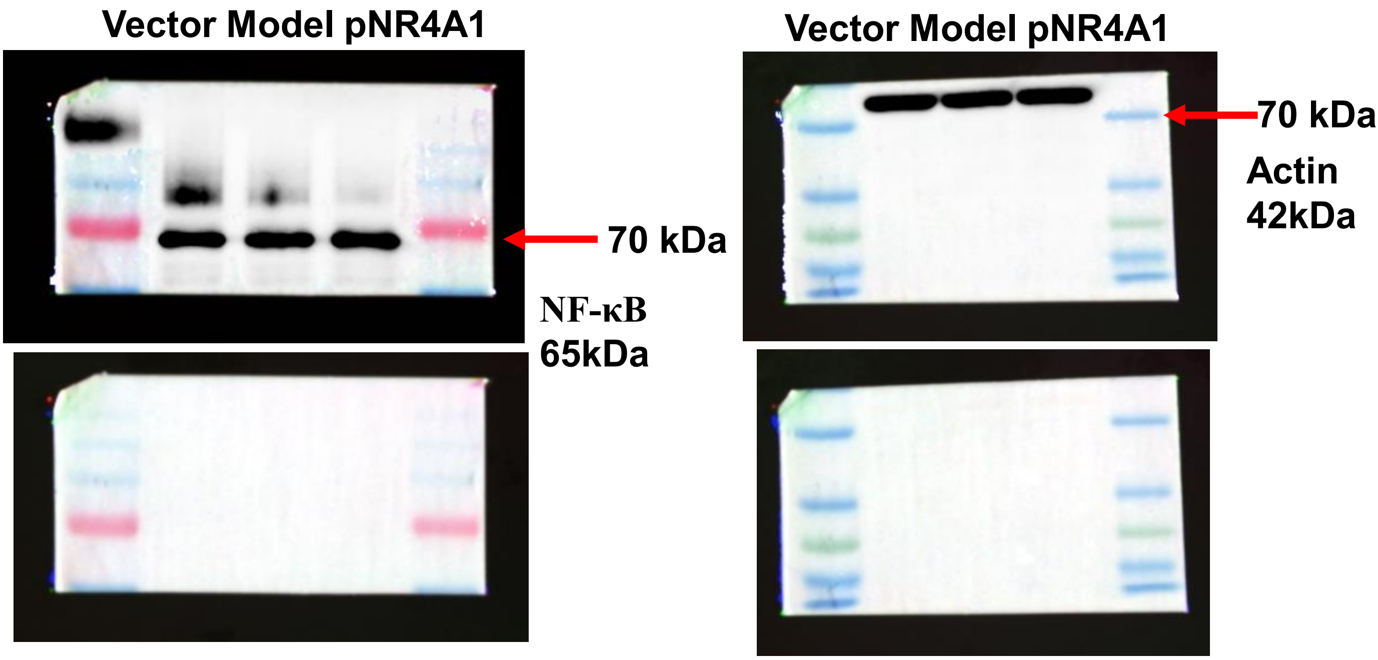

Repeat 3, the merged image

•

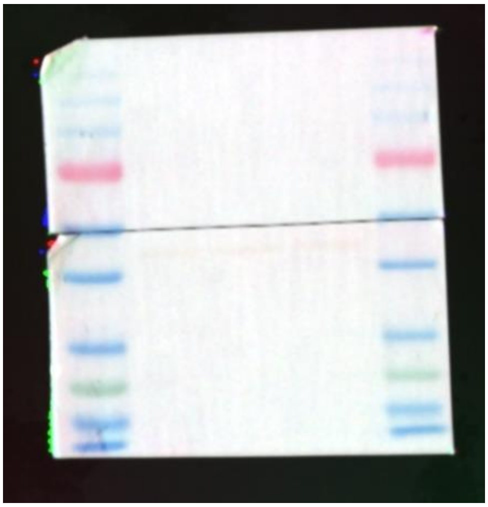

Repeat 4

Vector Model pNR4A1

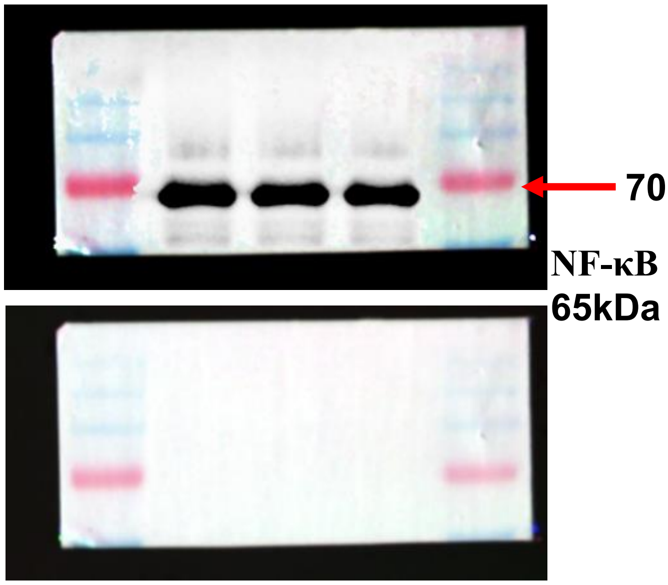

70 kDa

NF-κB 65kDa

Vector Model pNR4A1

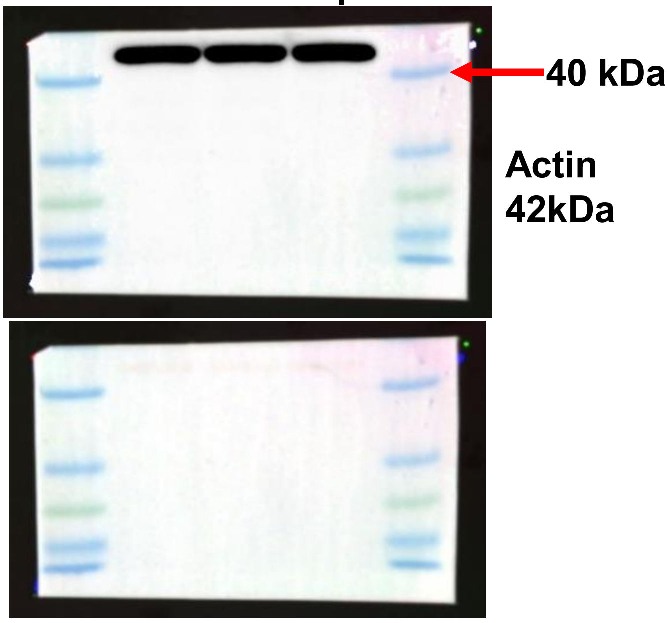

40 kDa

Actin 42kDa

Repeat 4, the merged image

•

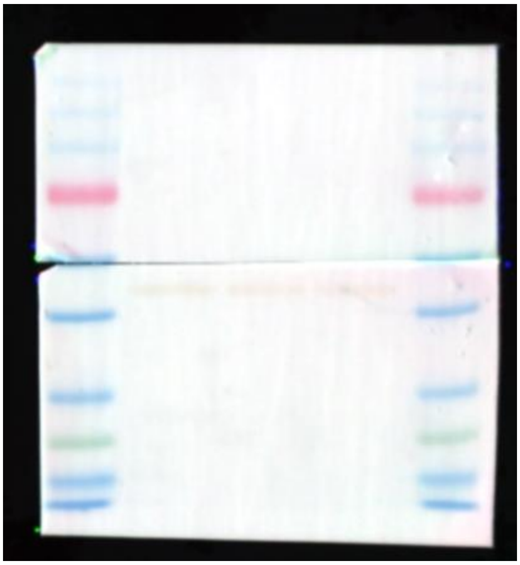

Western blot analysis was performed to examine the effect of AAV9-mediated NR4A1 overexpression on NR4A1 protein expression in renal tissues of anti-Thy1 nephritis rats

4 sample/group

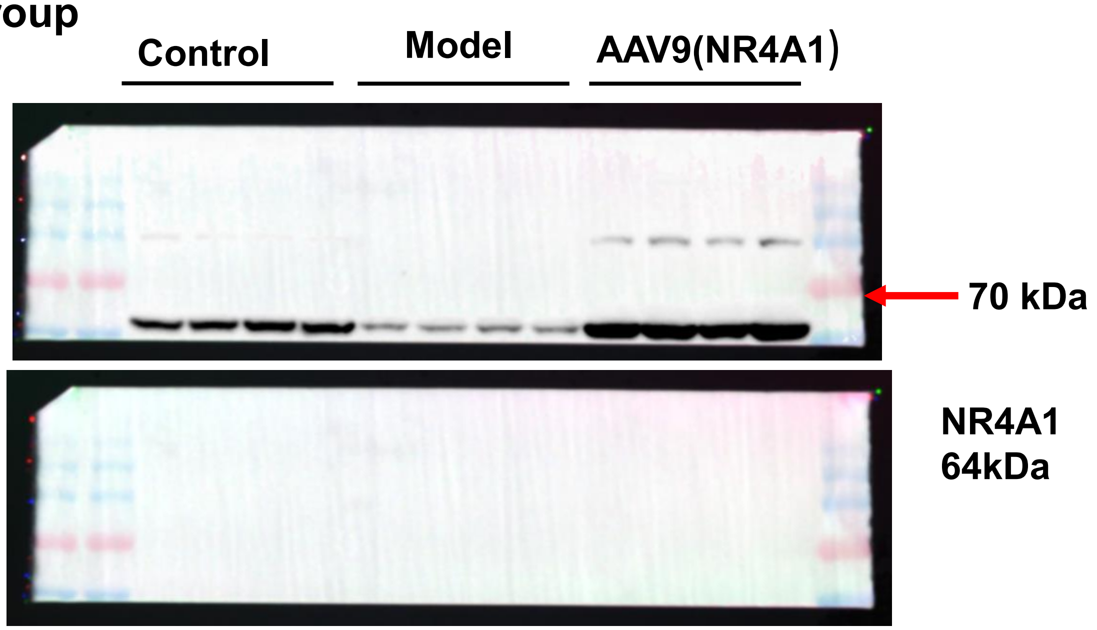

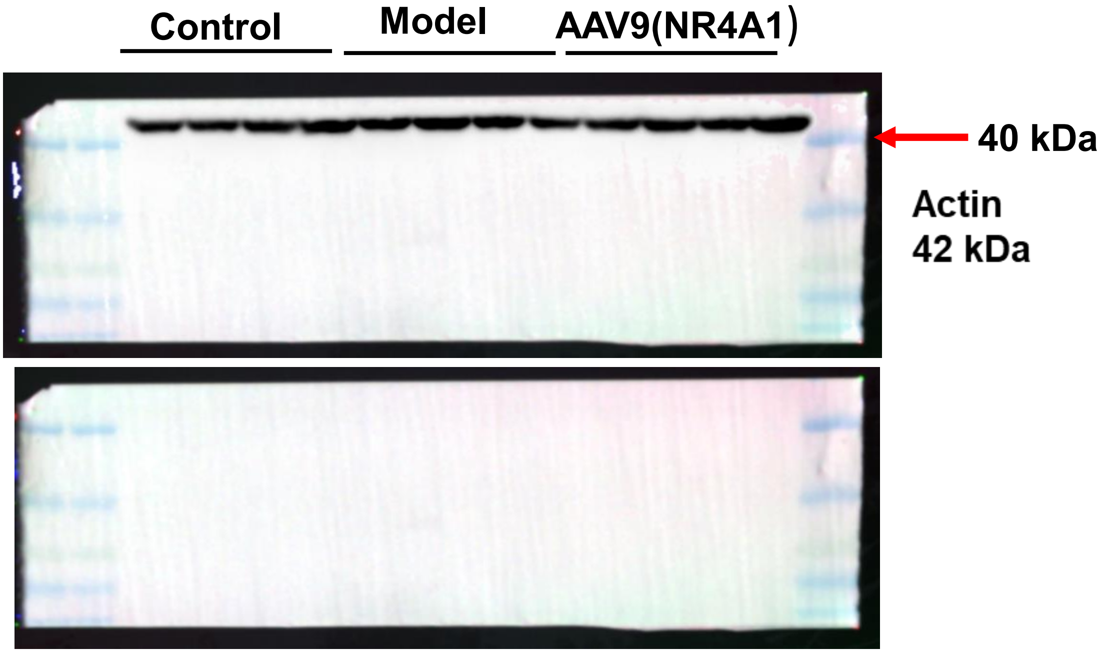

The merged image

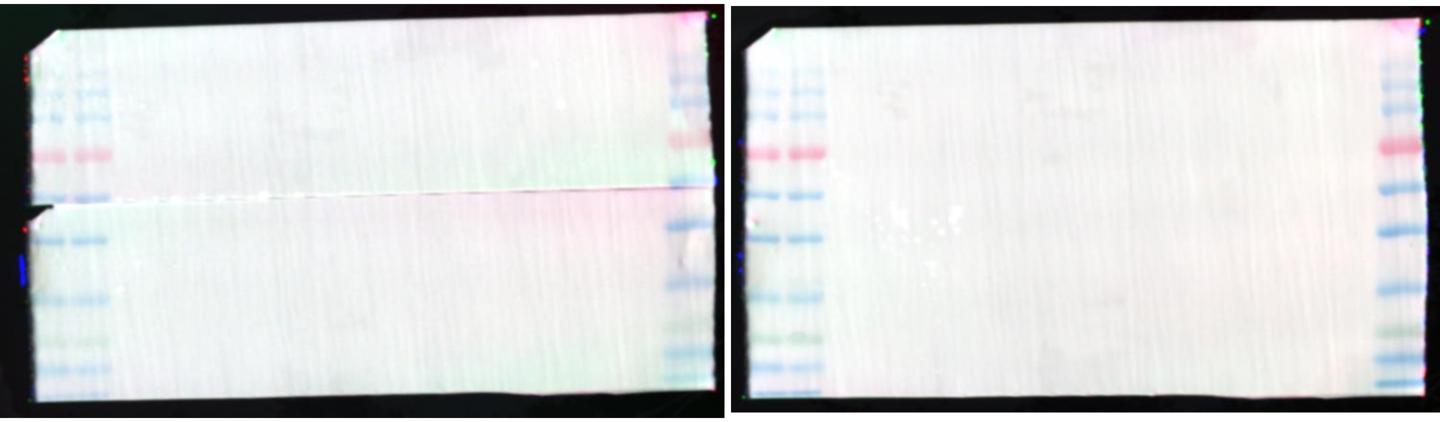

4 sample/group

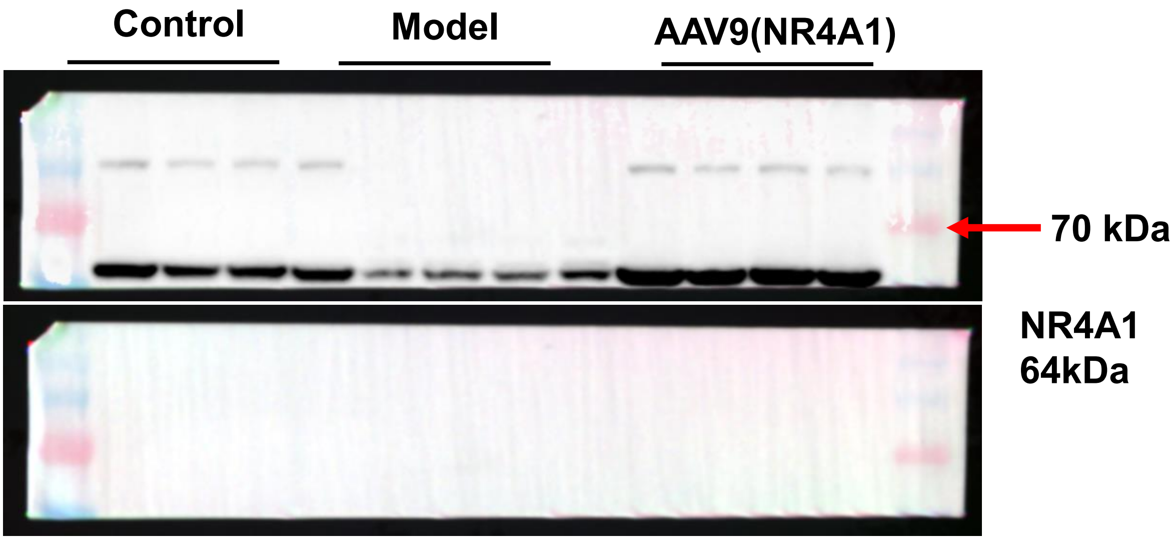

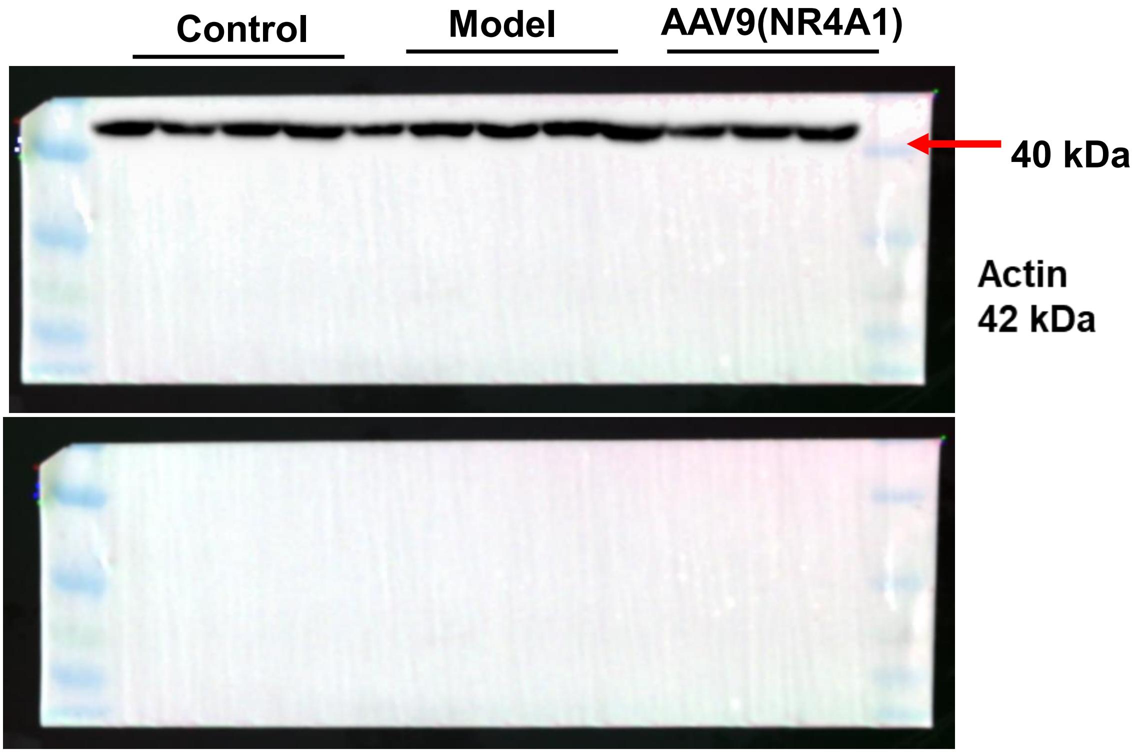

The merged image

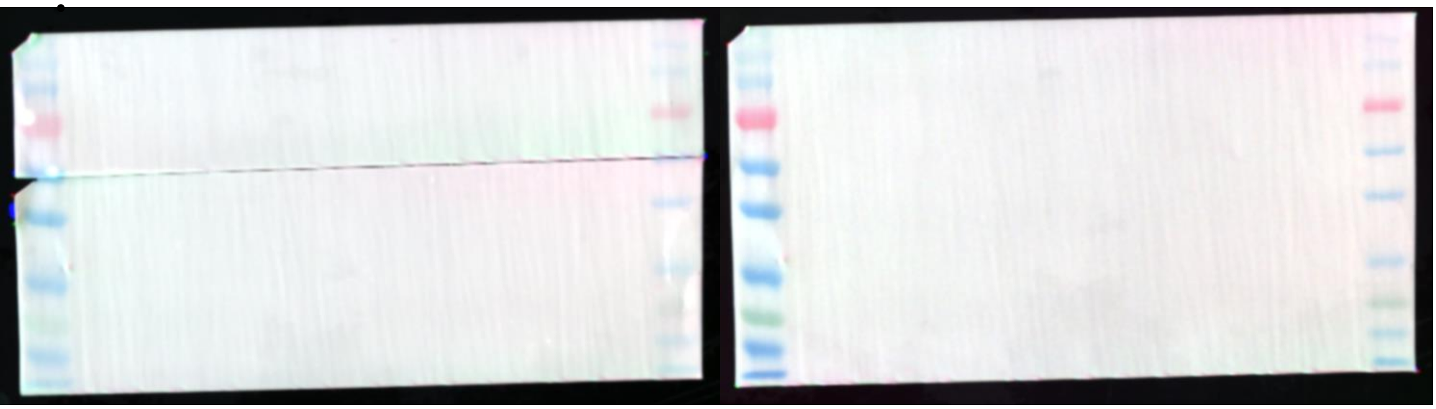

Western blot analysis was performed to examine the effect of AAV9-mediated NR4A1 overexpression on p-NF-κB protein expression in renal tissues of anti-Thy1 nephritis rats

4 sample/group

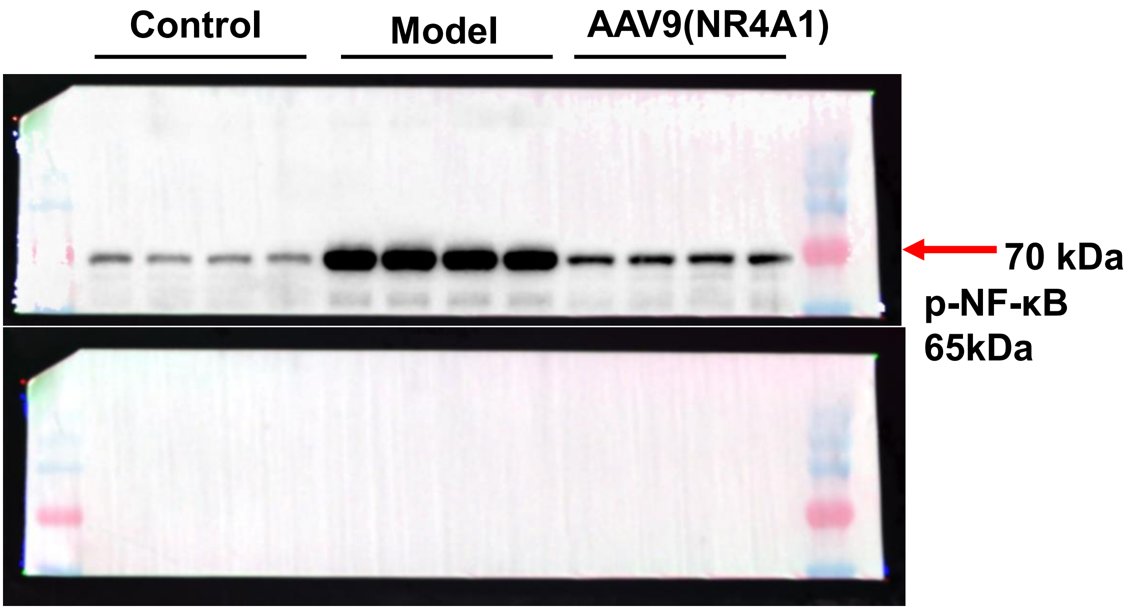

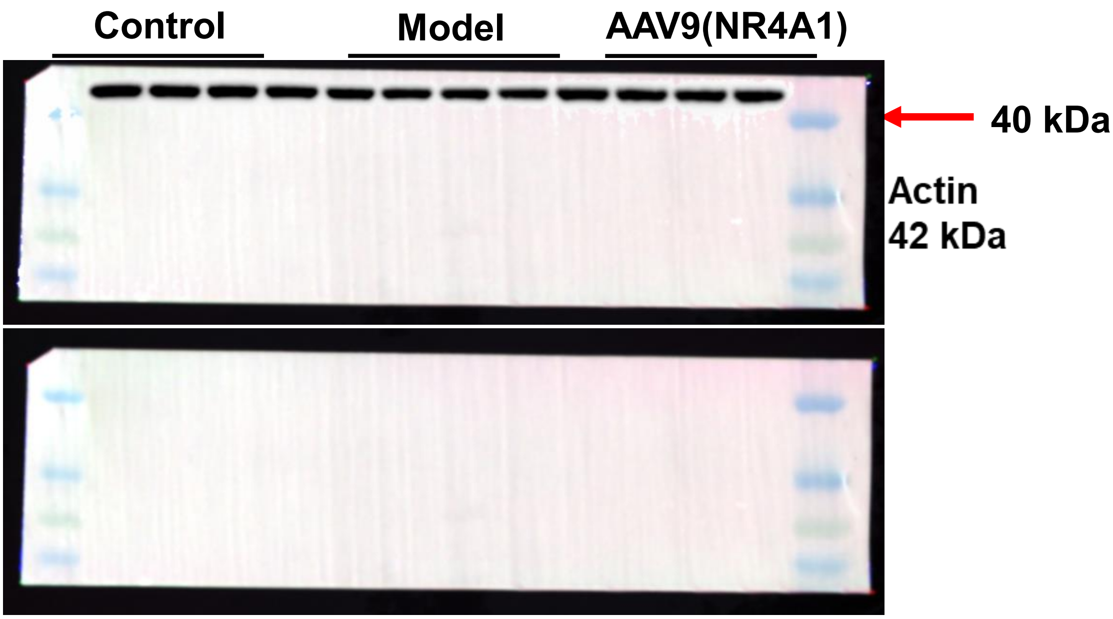

The merged image

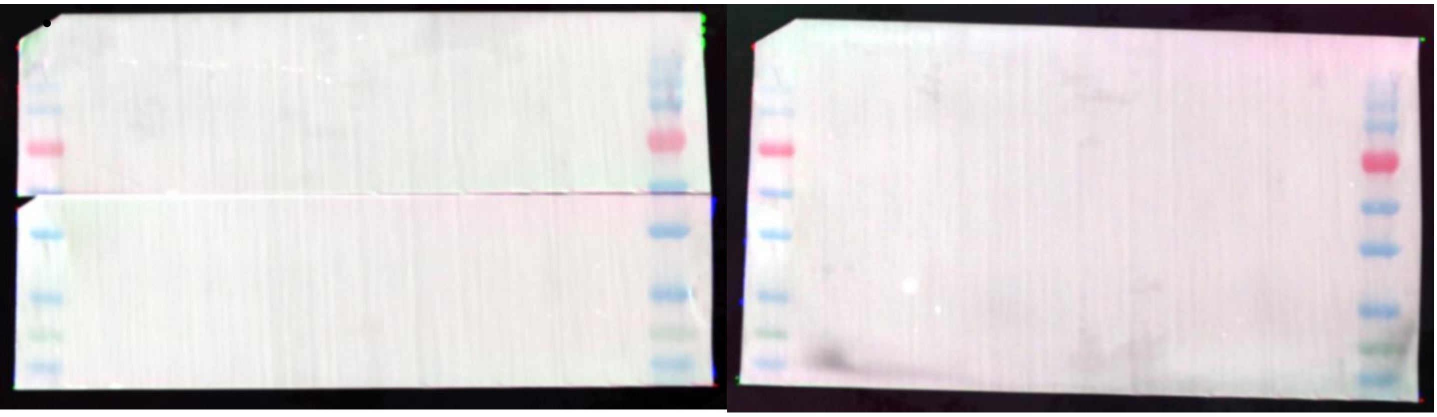

4 sample/group

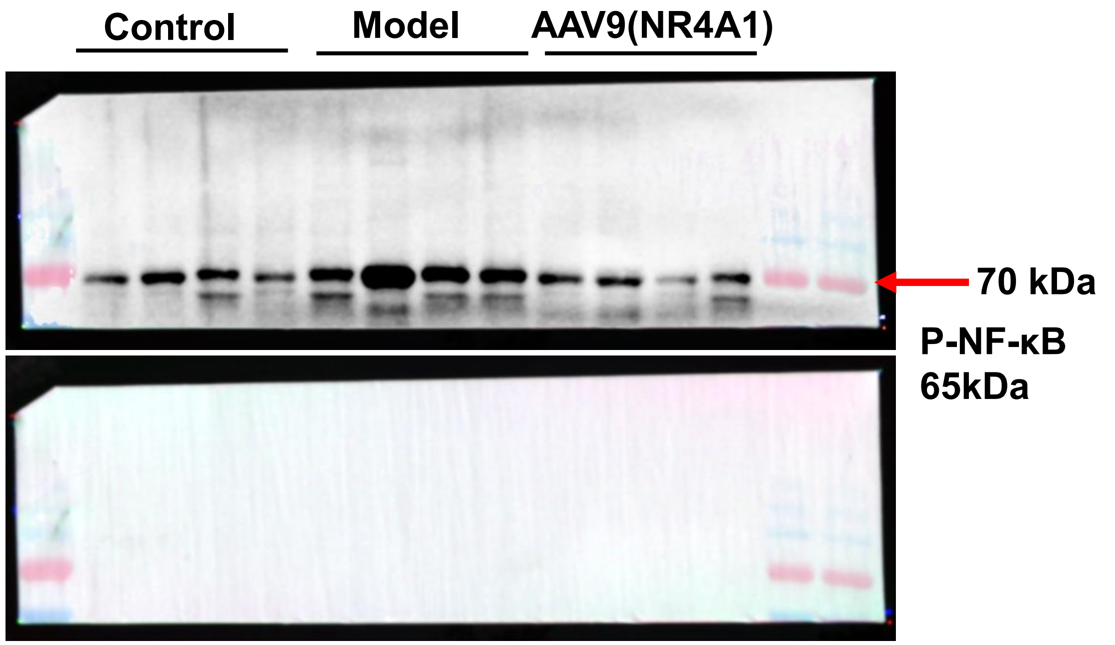

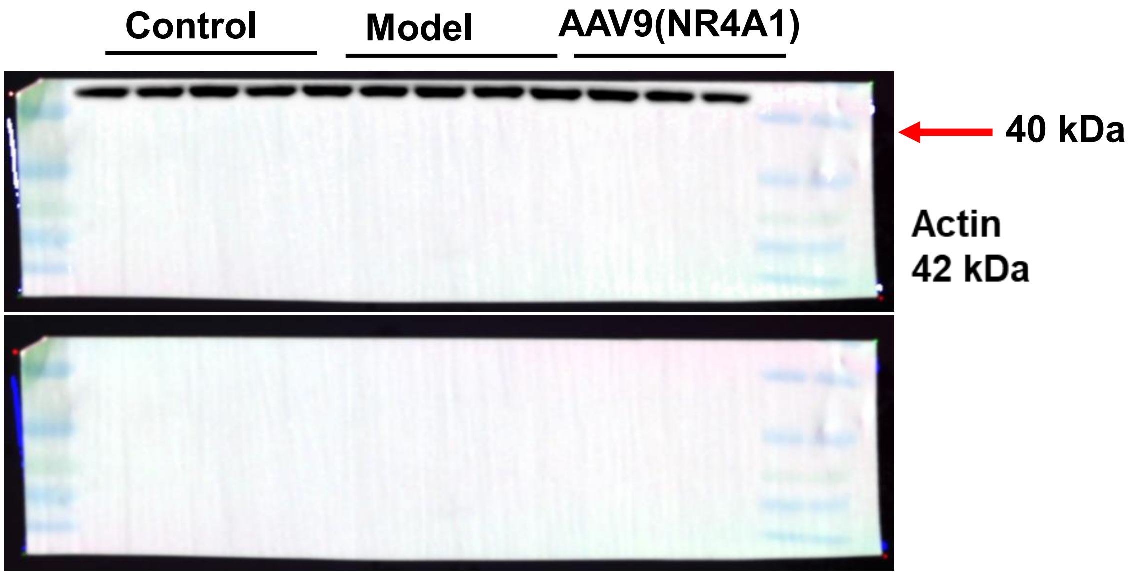

The merged image

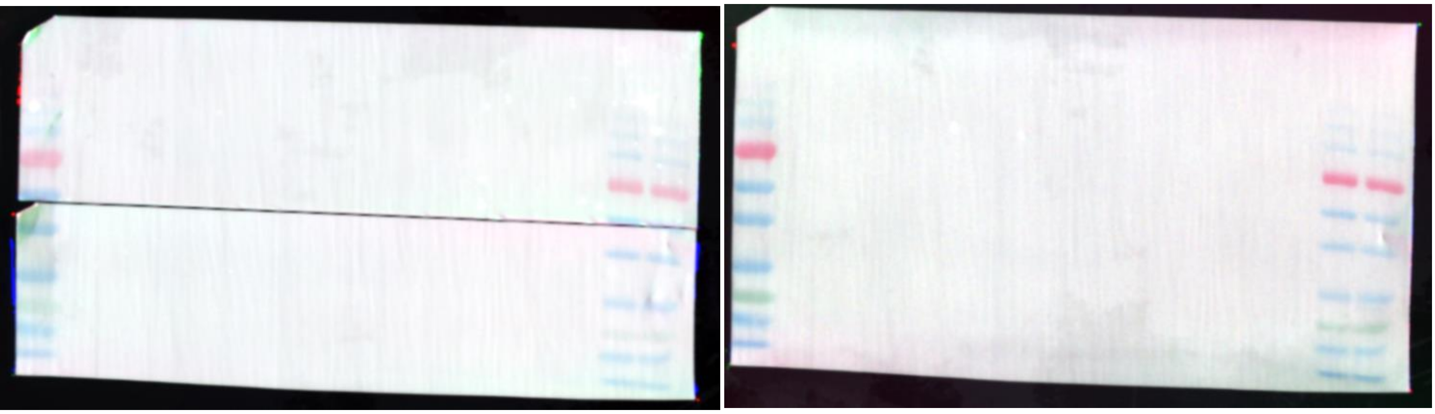

Western blot analysis was performed to examine the effect of AAV9-mediated NR4A1 overexpression on NF-κB protein expression in renal tissues of anti-Thy1 nephritis rats

4 sample/group

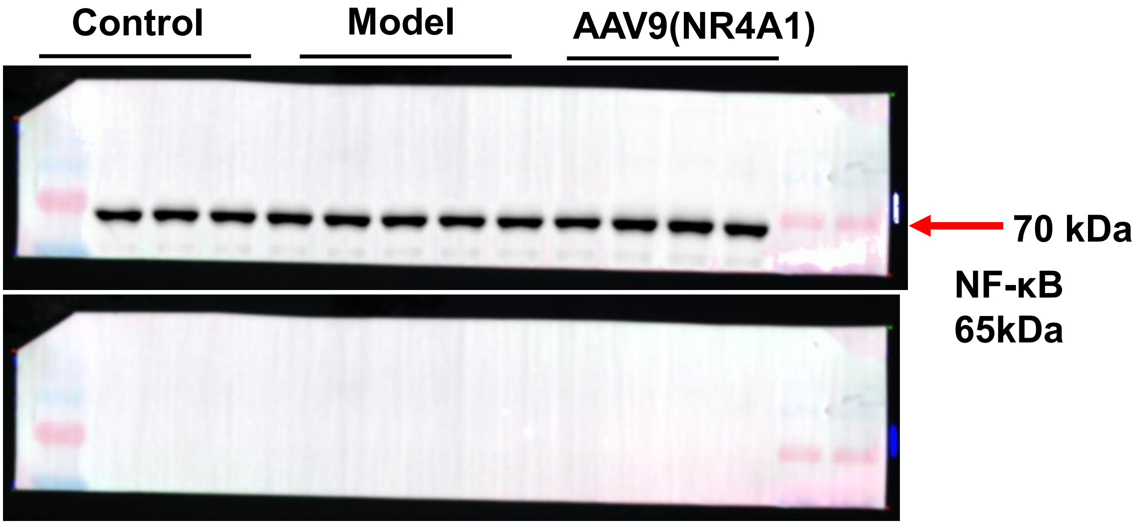

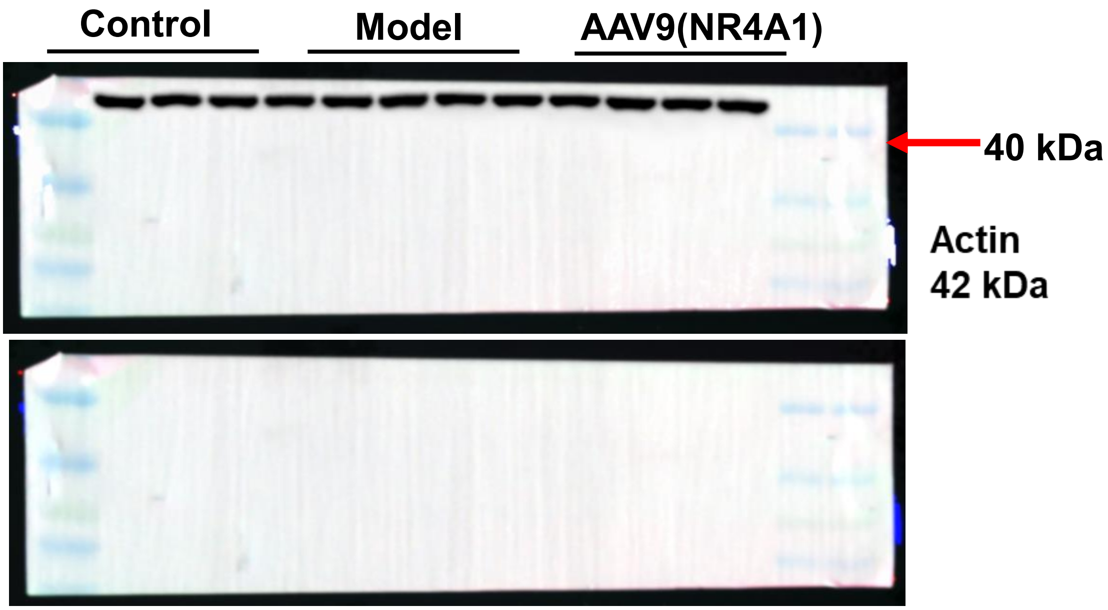

The merged image

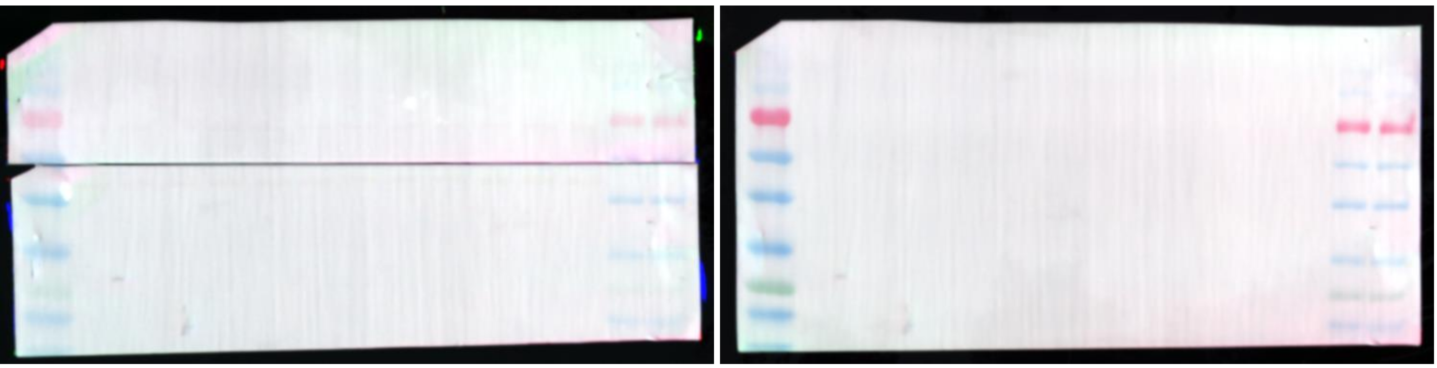

4 sample/group

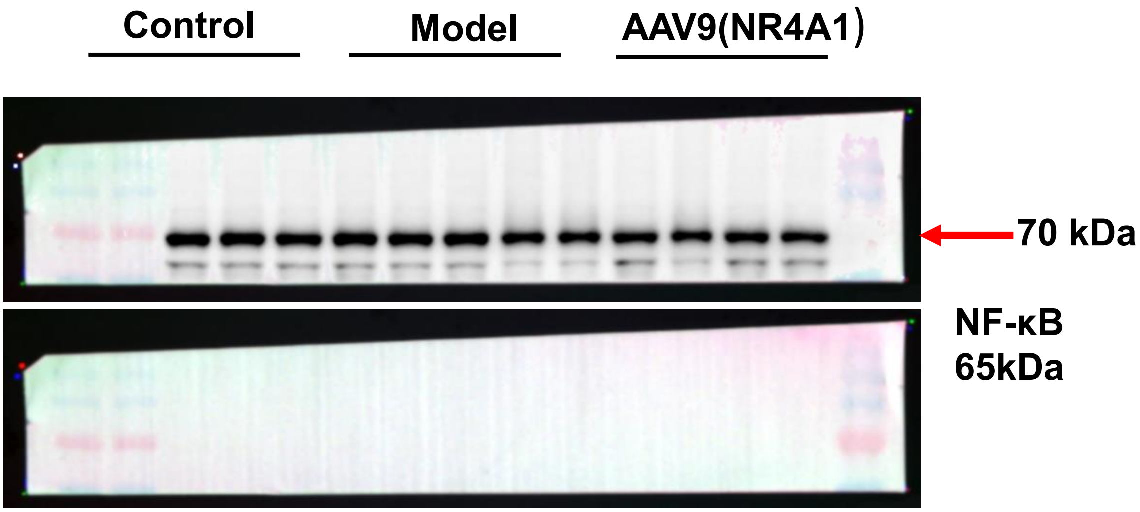

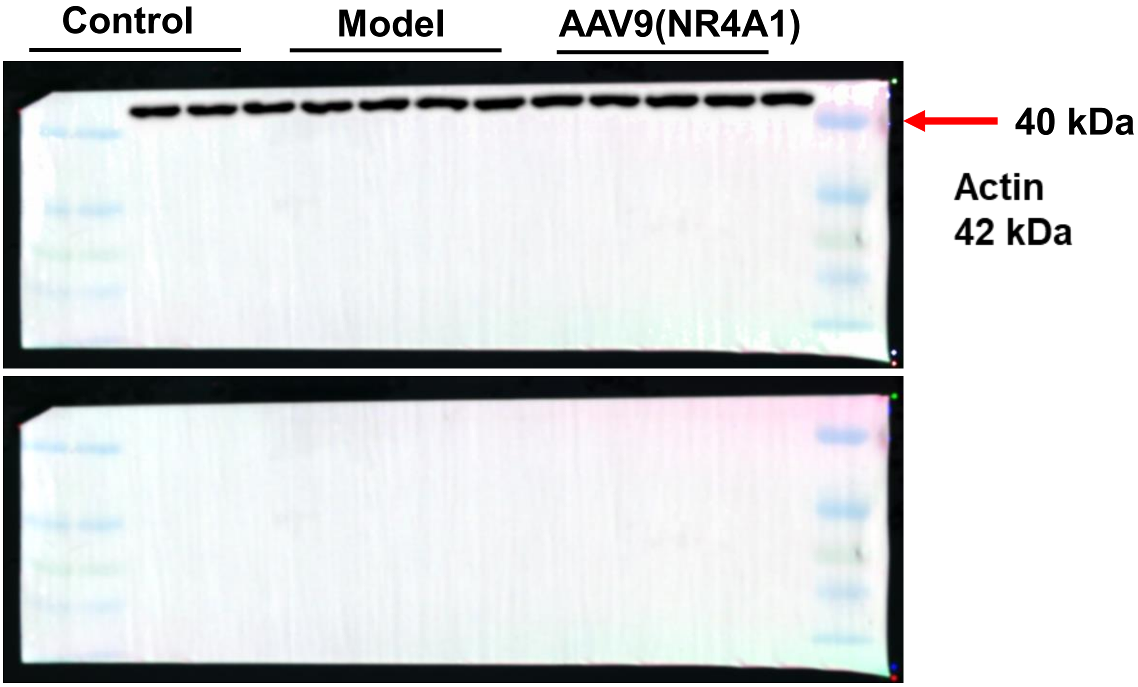

The merged image

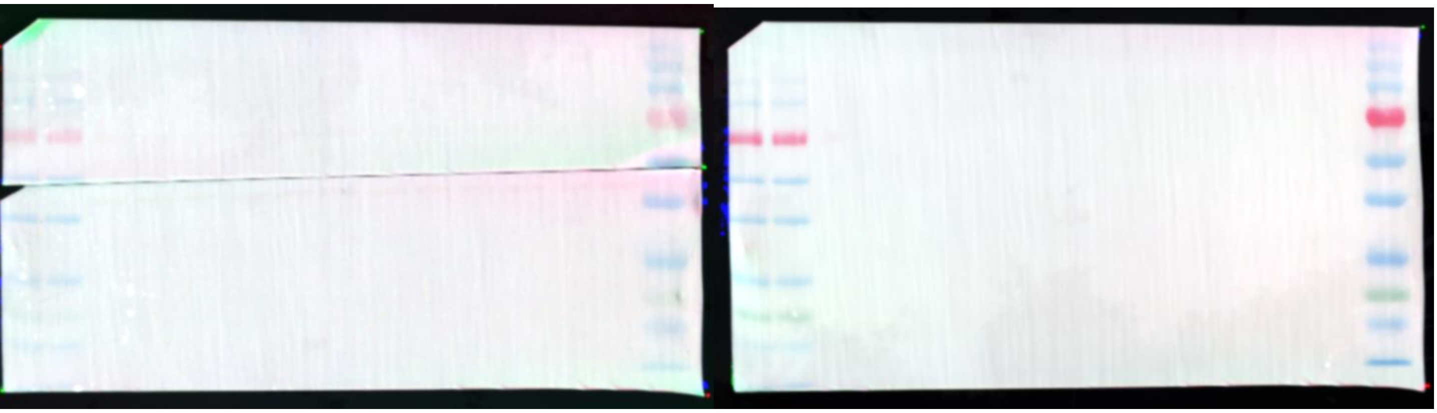

DARTS experiments

Repeat 1

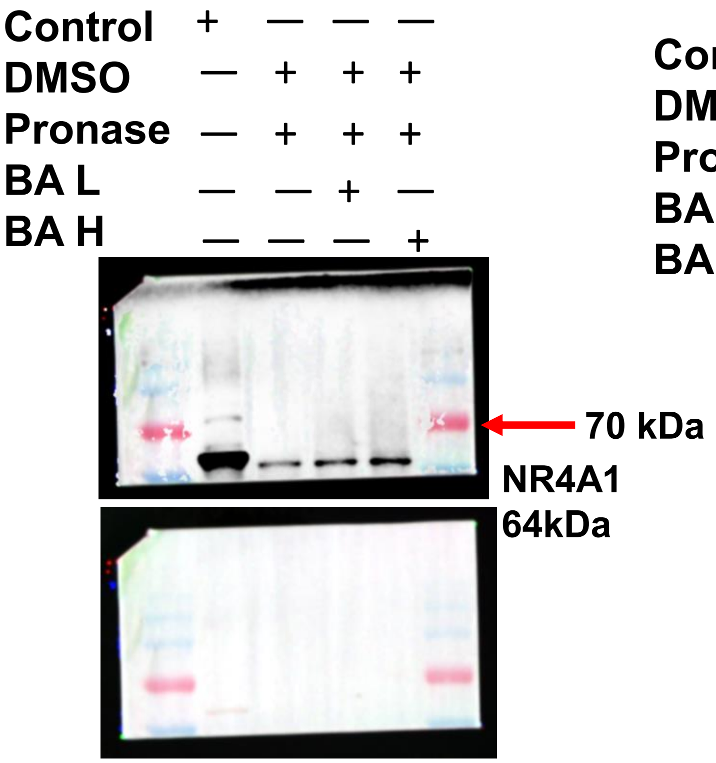

Repeat 1

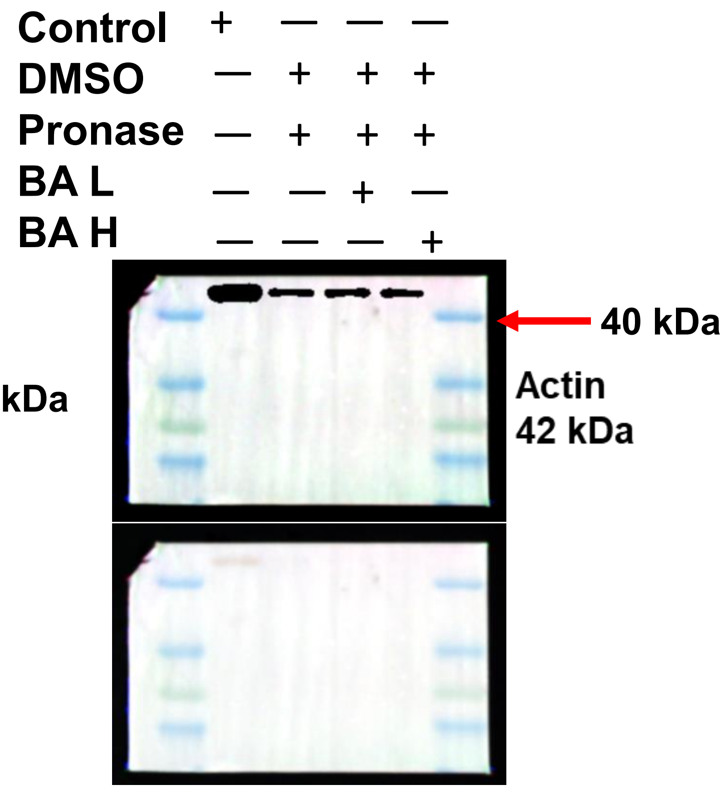

Repeat 1, the merged image

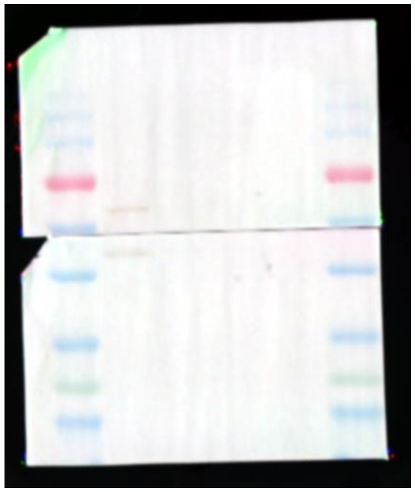

Repeat 2

Repeat 2

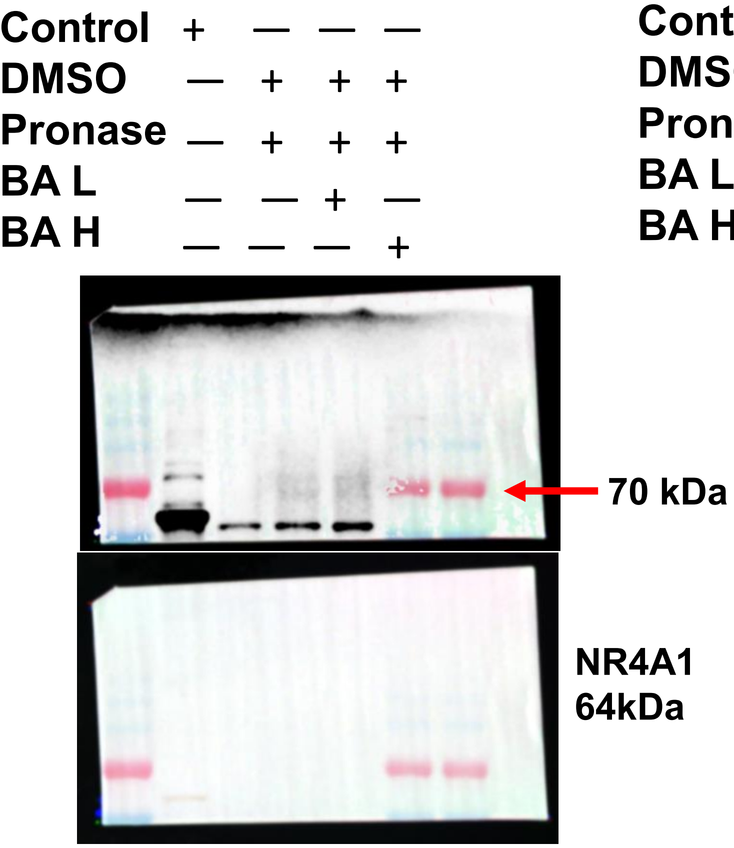

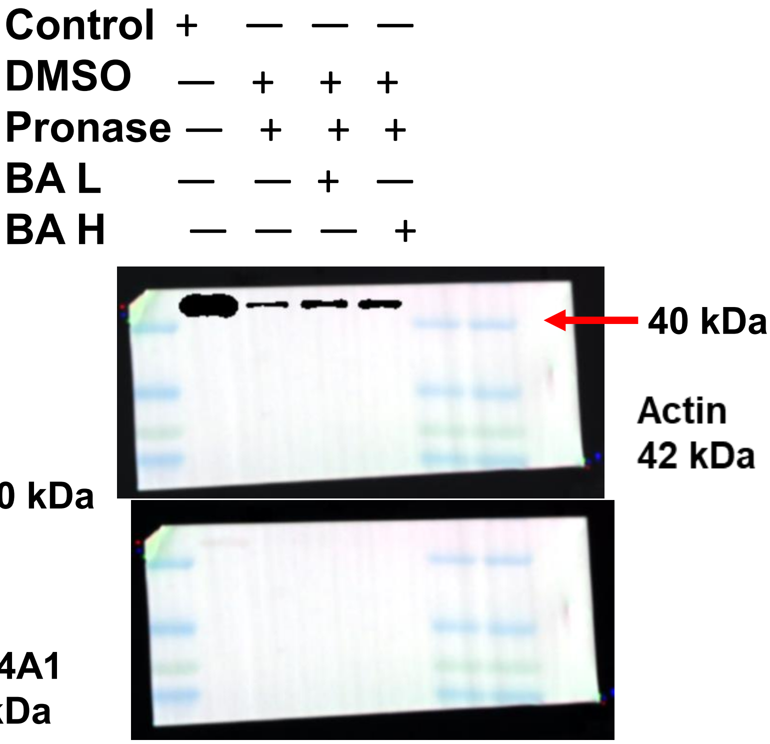

Repeat 2 ,the merged image

•

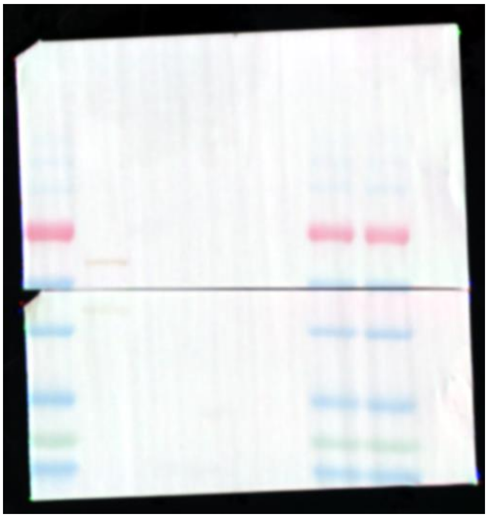

Repeat 1 and Repeat 2 ,the merged image

•

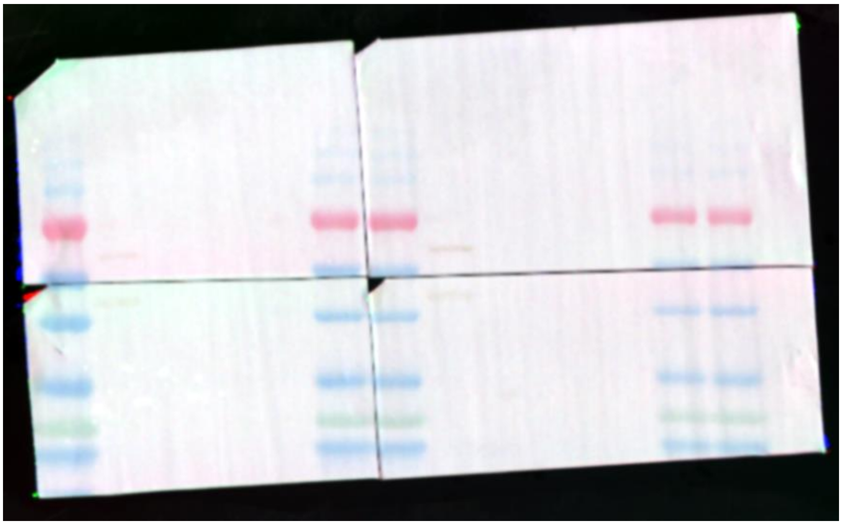

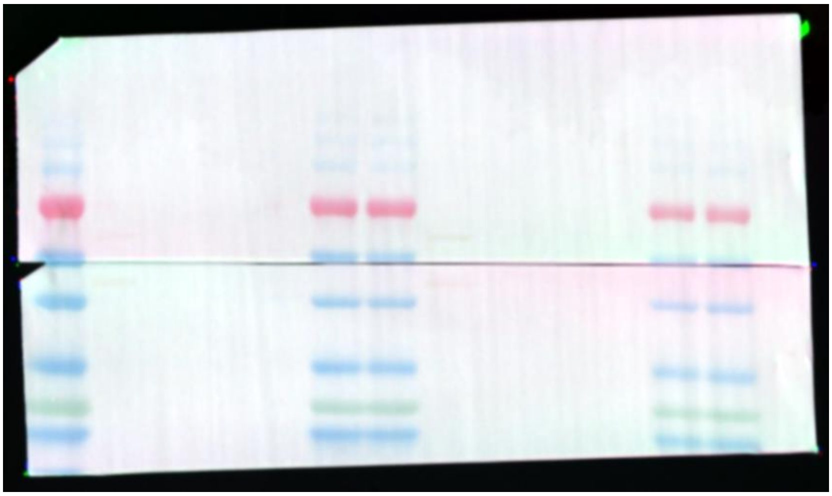

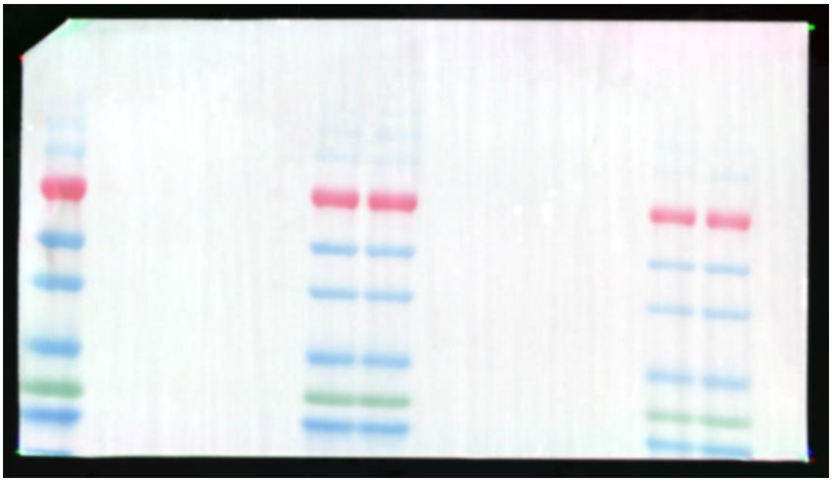

Repeat 3

Control	+	—	—	—
DMSO	—	+	+	+
Pronase	—	+	+	+
BA L	—	—	+	—
BA H	—	—	—	+

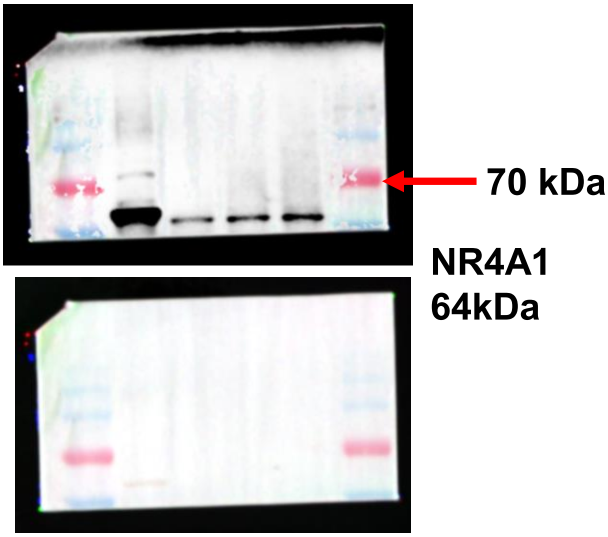

Repeat 3

Control	+	—	—	—
DMSO	—	+	+	+
Pronase	—	+	+	+
BA L	—	—	+	—
BA H	—	—	—	+

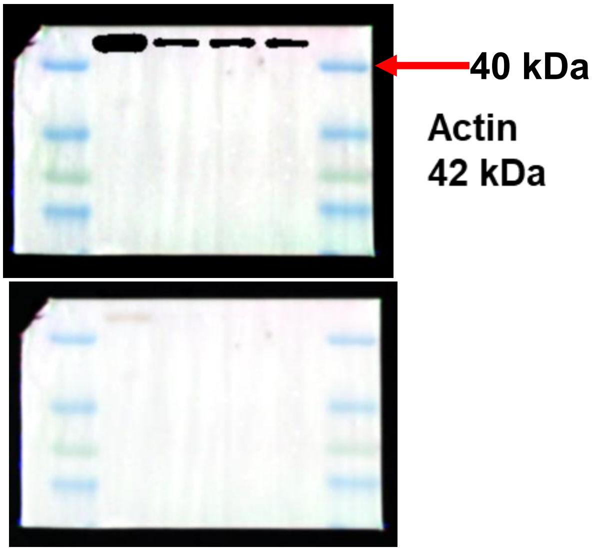

Repeat 3 ,the merged image

•

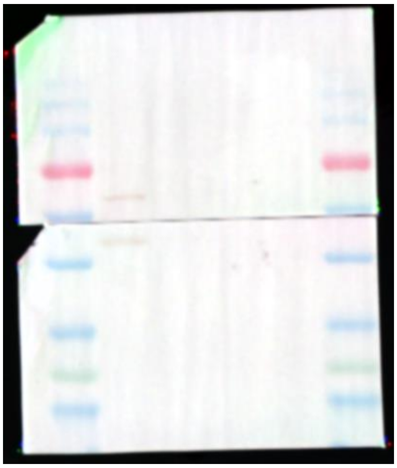

Repeat 4

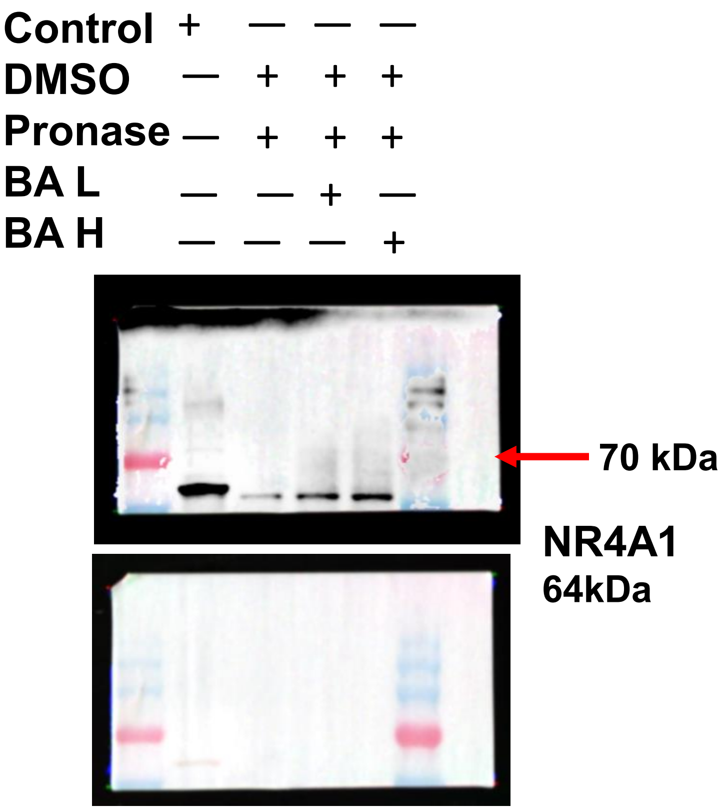

Repeat 4

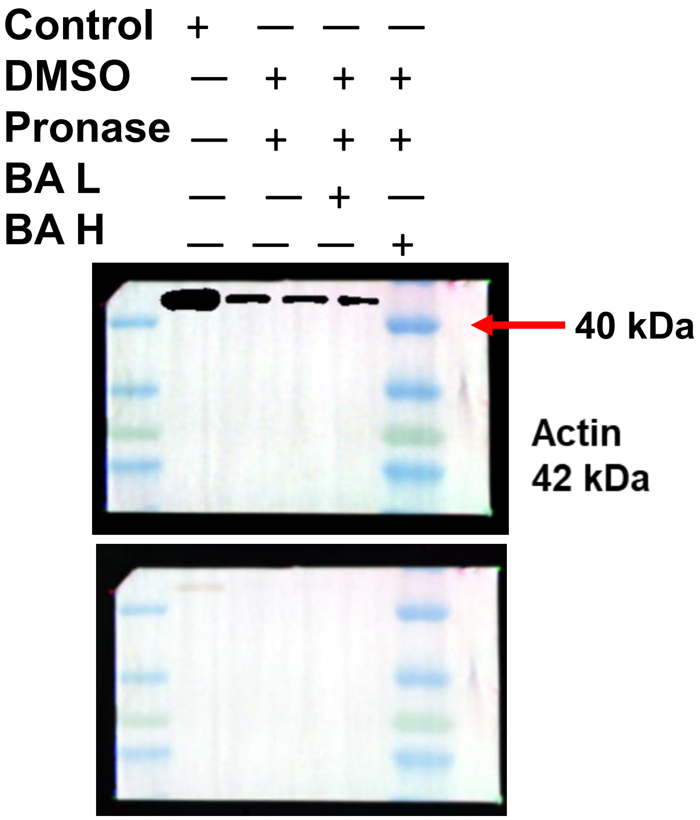

Repeat 4 ,the merged image

•

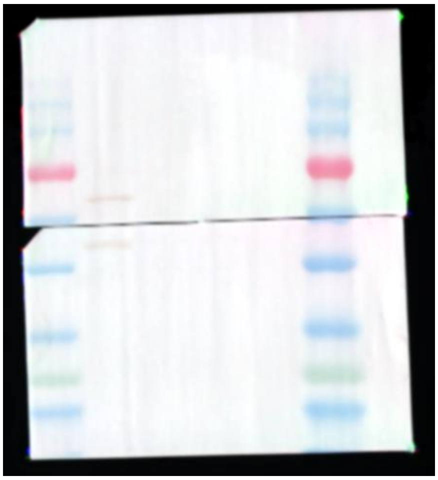

Repeat 3 and Repeat 4 , the merged image

-

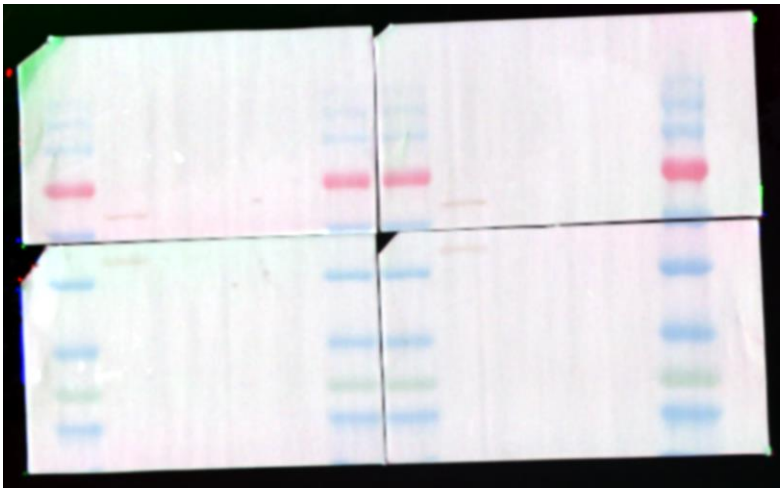

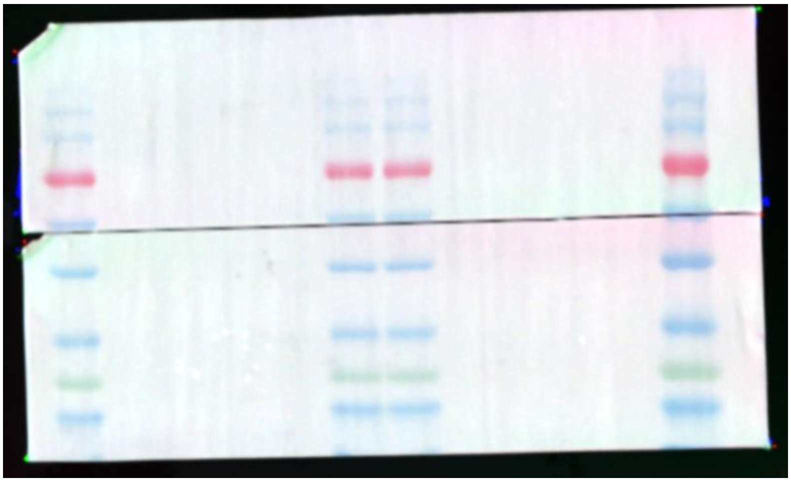

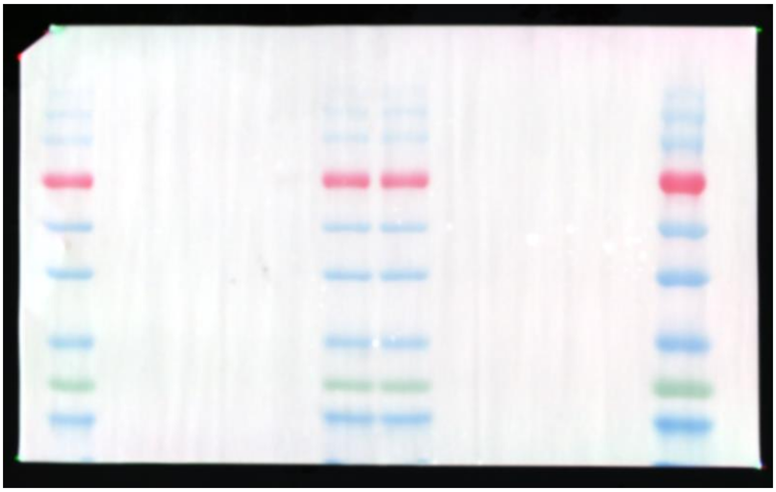

CETSA experiments

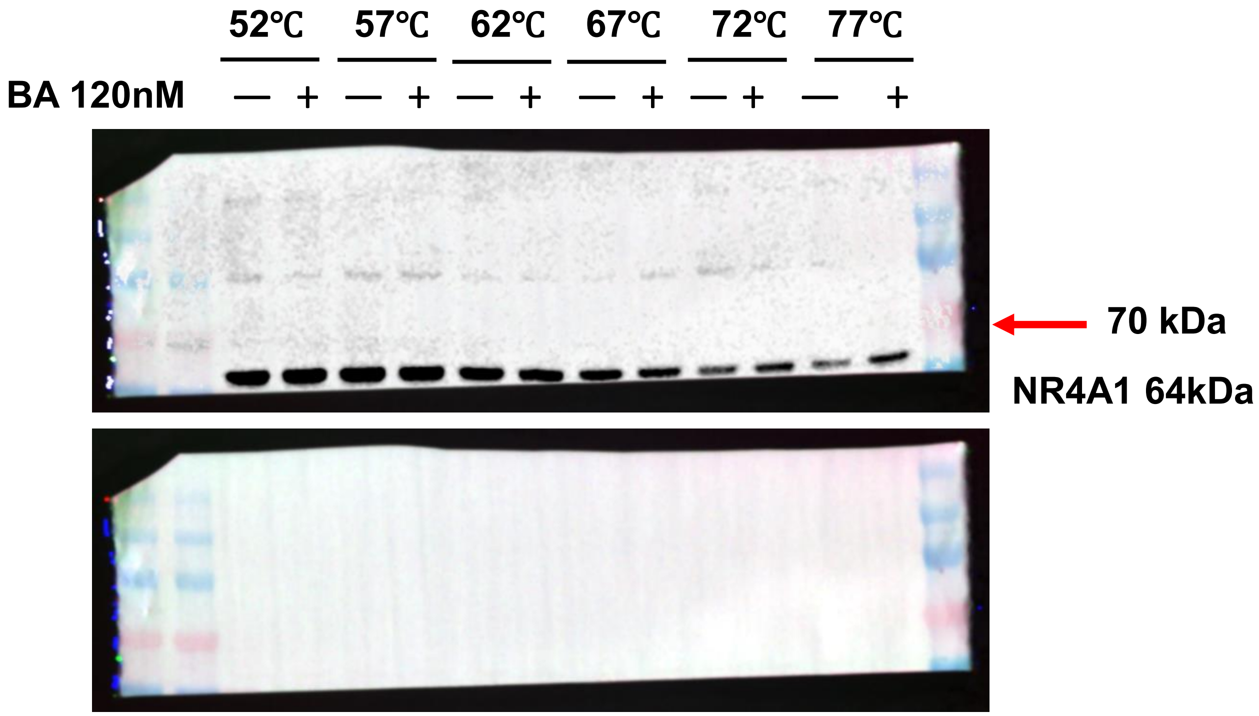

Repeat 1

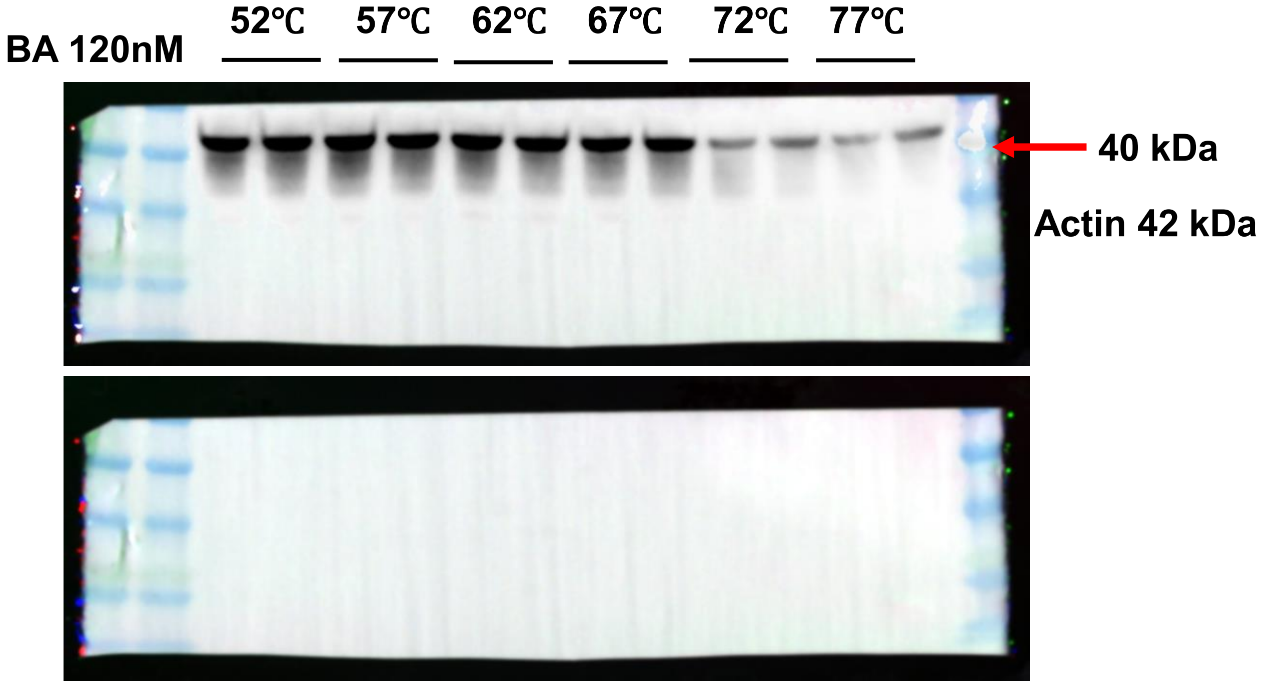

Repeat 1, the merged image

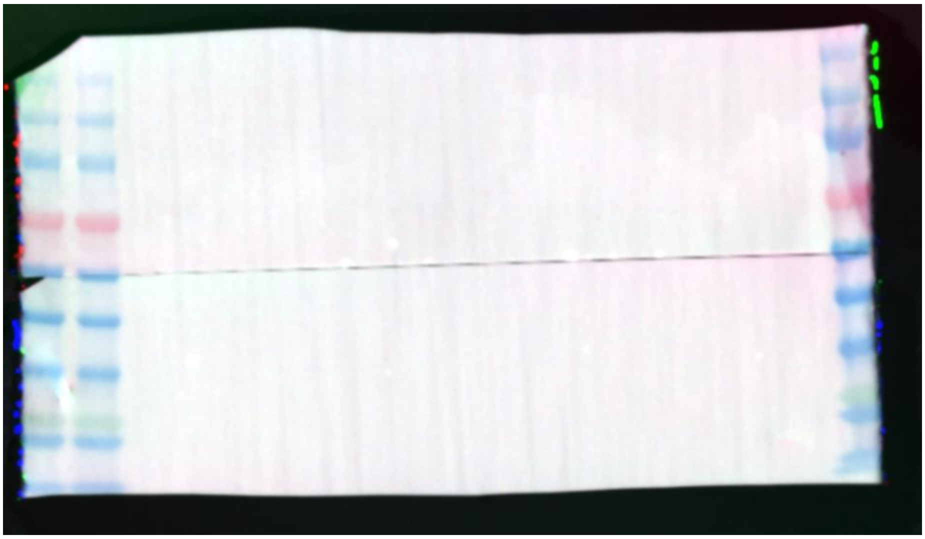

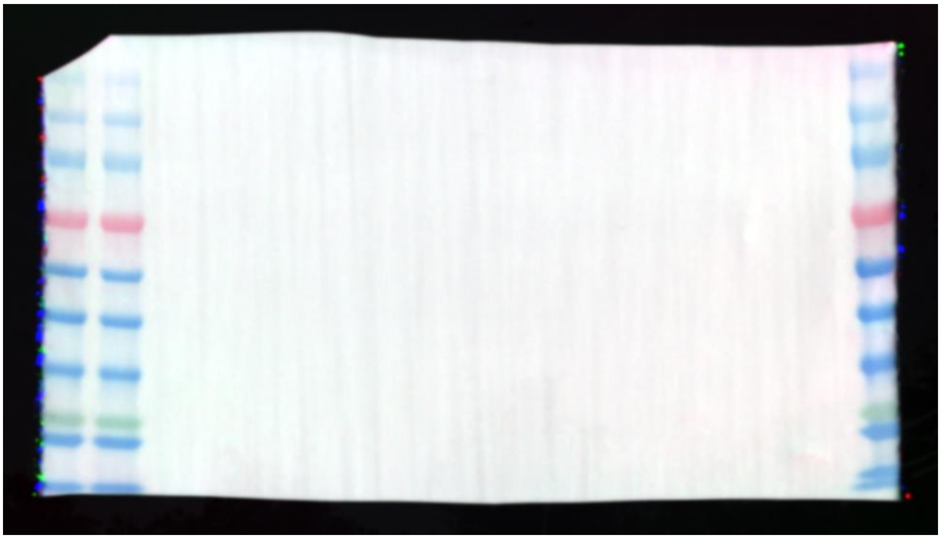

Repeat 2

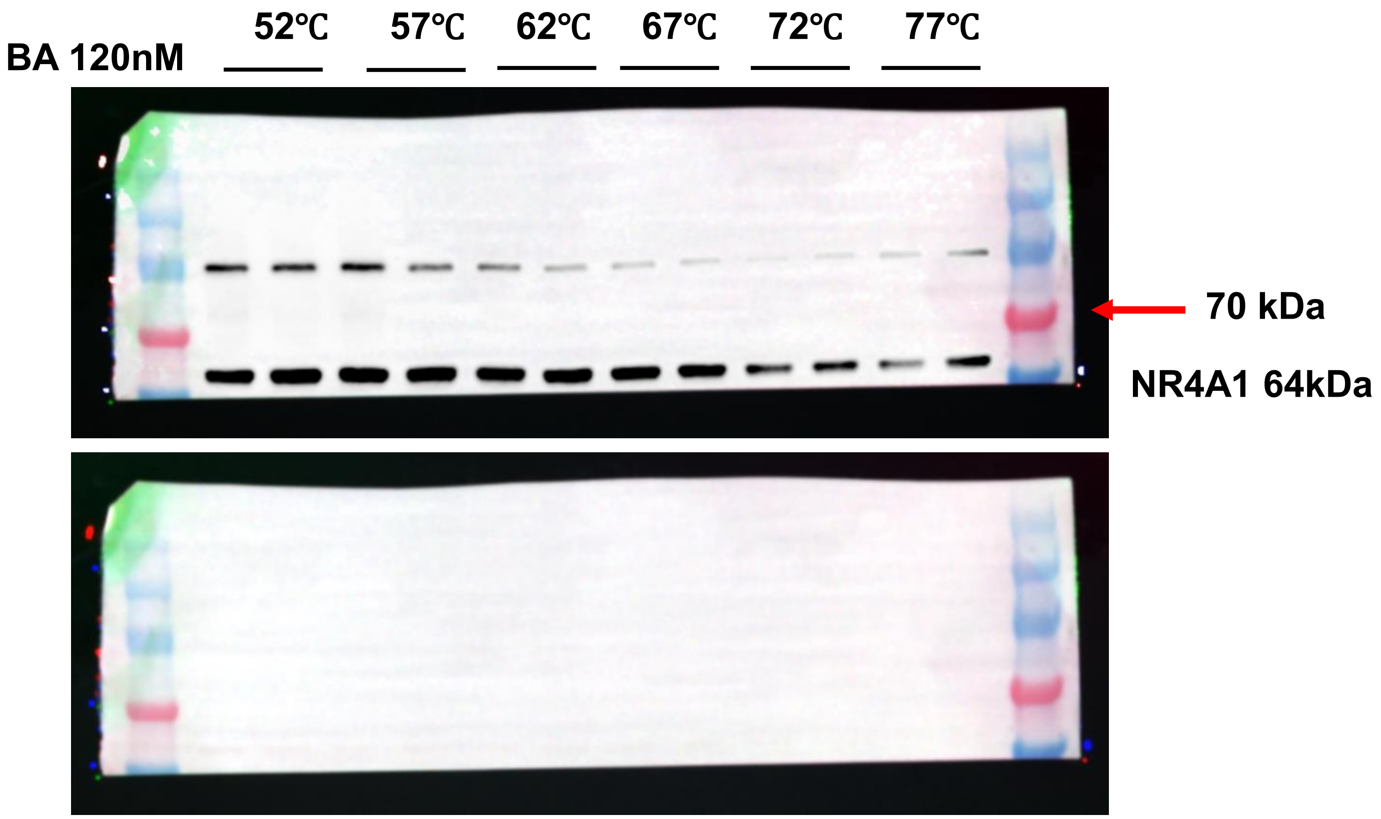

Repeat 2

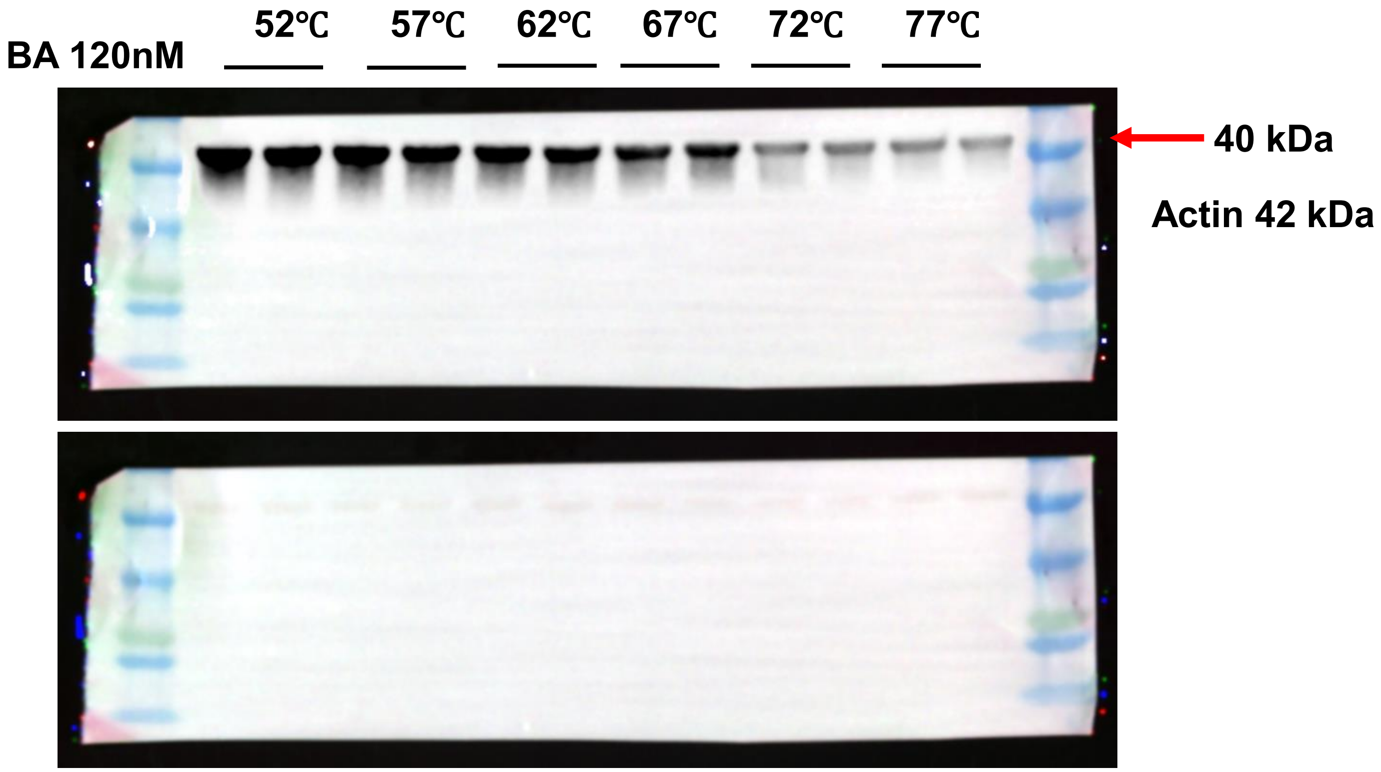

Repeat 2 ,the merged image

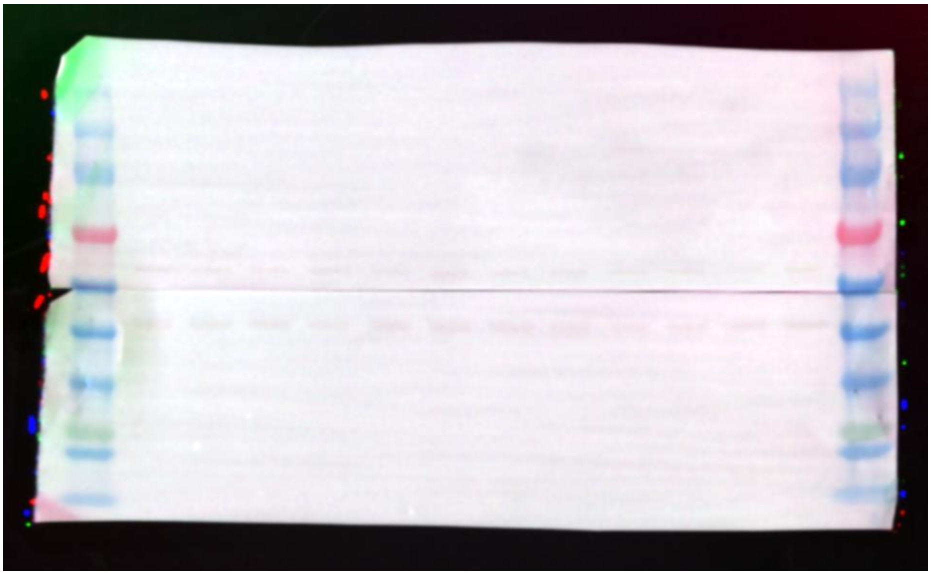

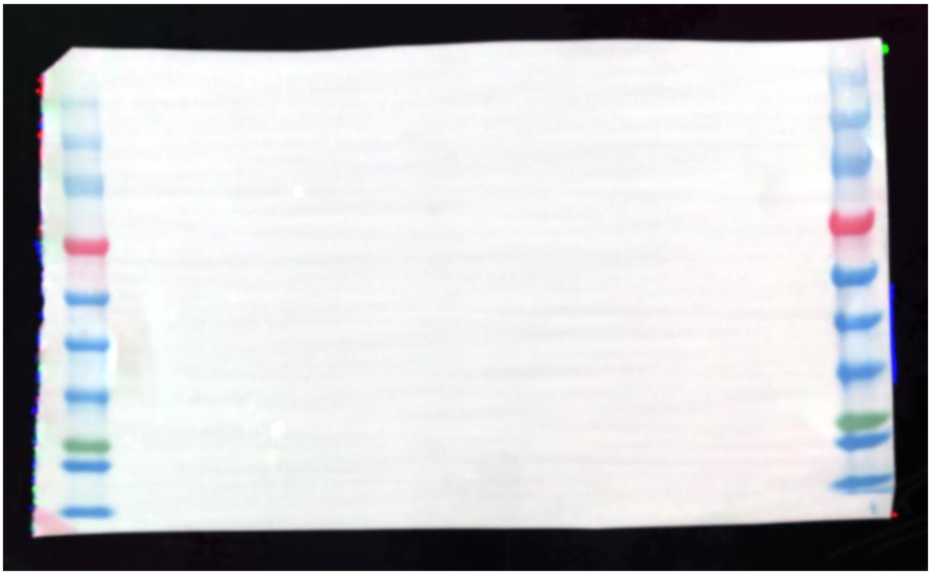

Repeat 3

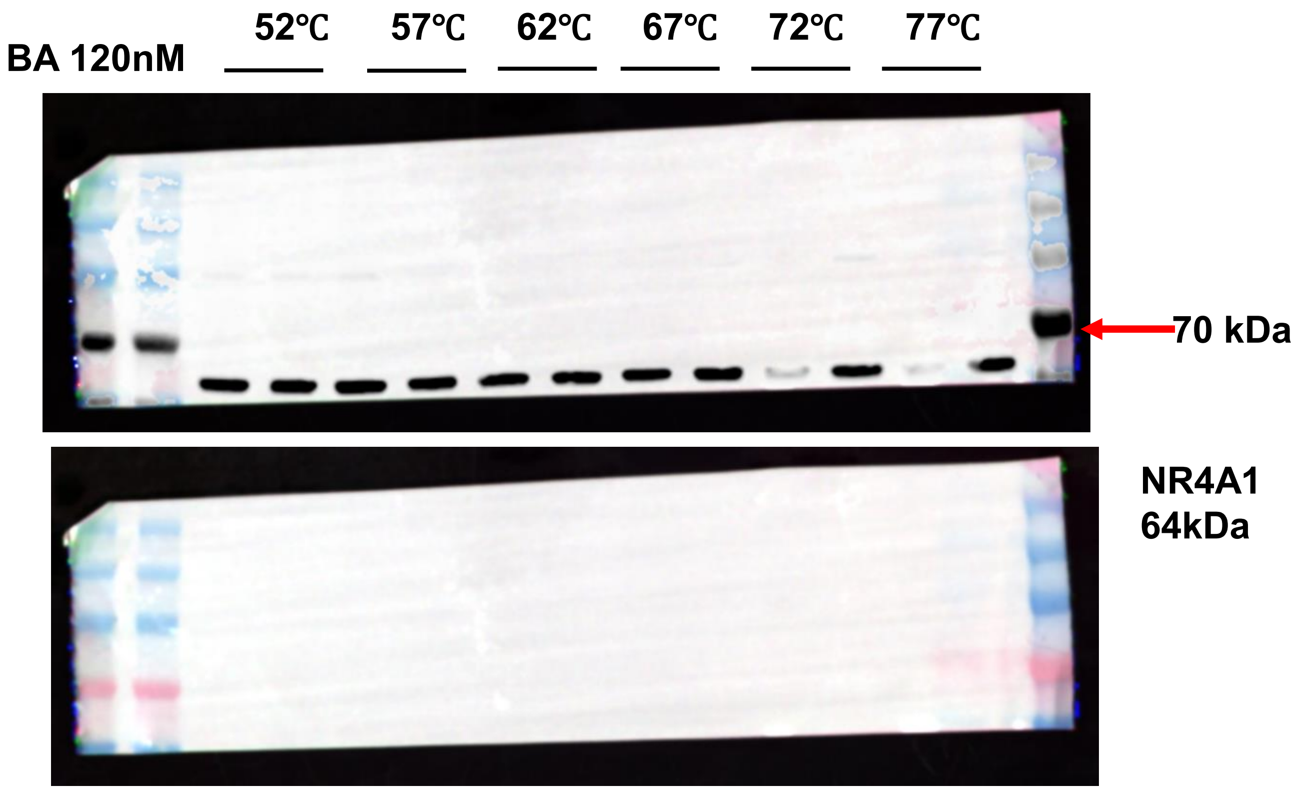

Repeat 3

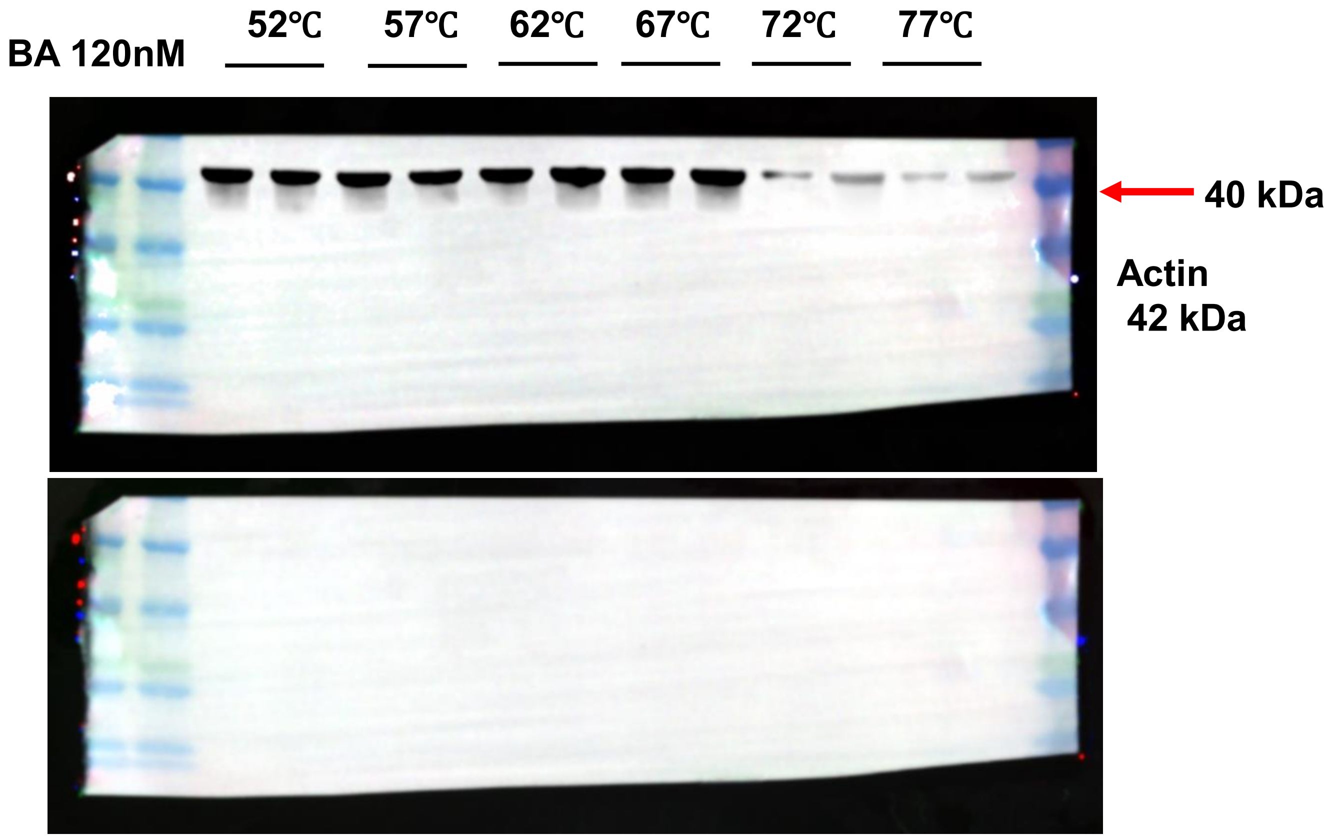

Repeat 3, the merged image

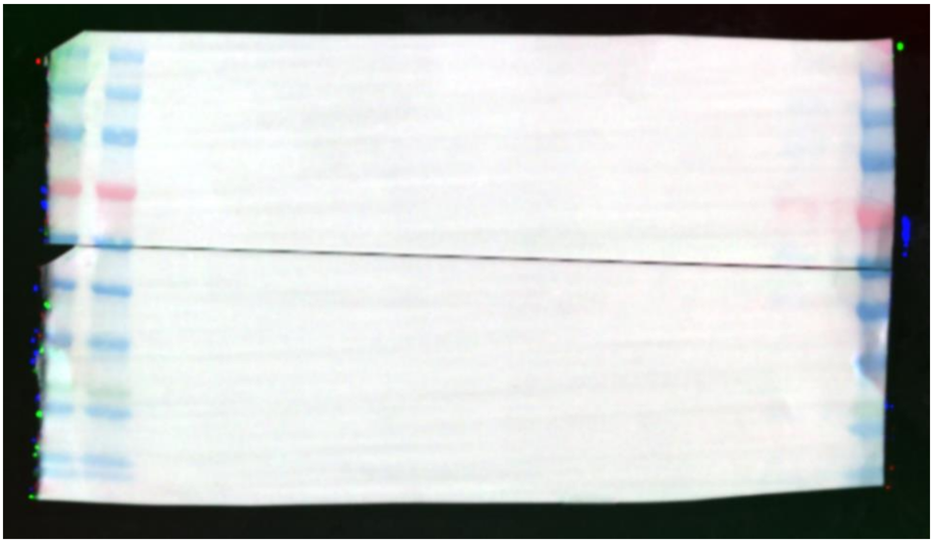

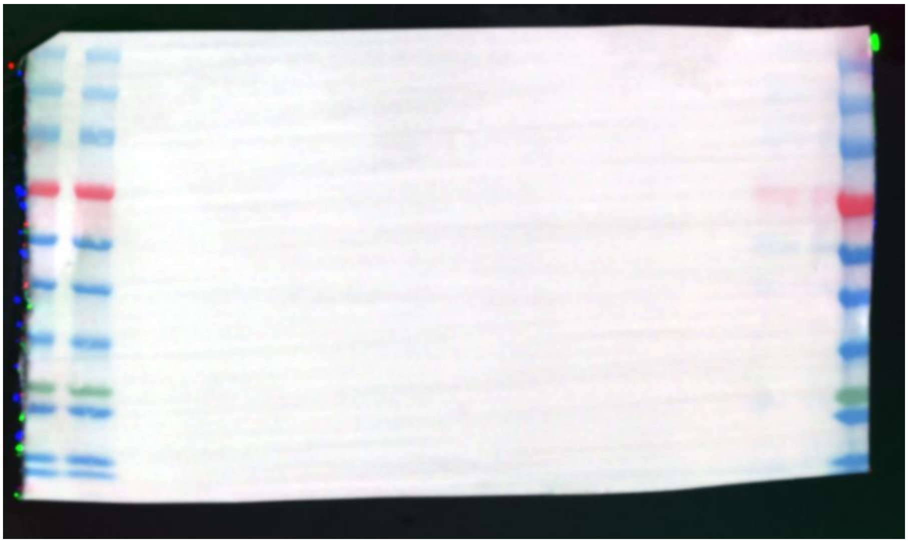

Western blot was performed to evaluate the effects of BA on NR4A1 protein expression in renal tissues of anti-Thy1 nephritis rats *in vivo*

4 samples/per group

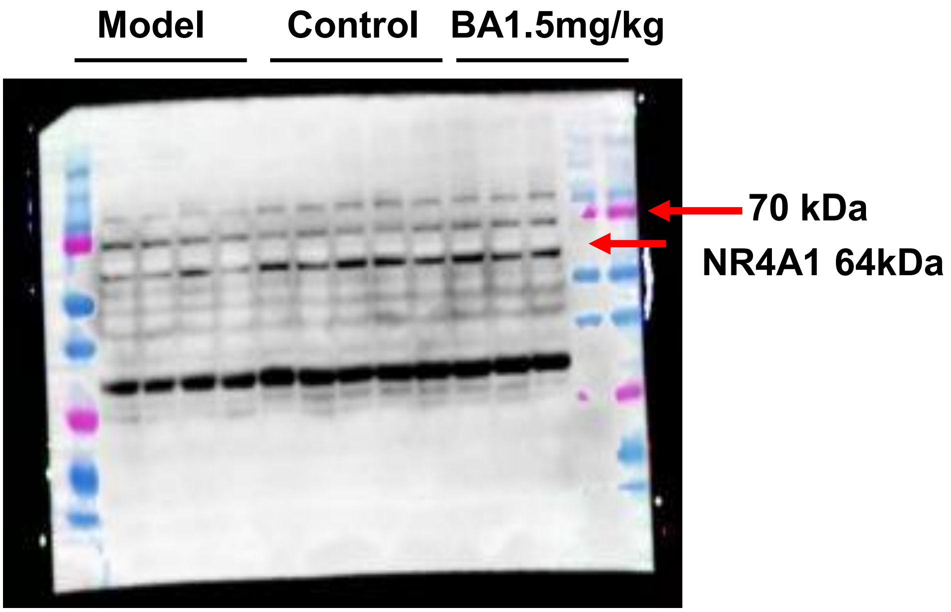

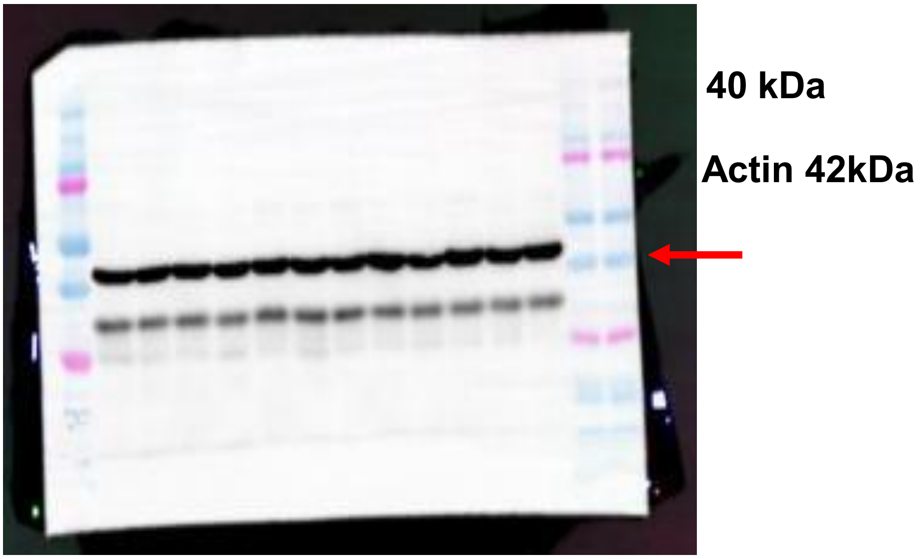

Western blot was performed to evaluate the effects of BA on NR4A1 protein expression in renal tissues of anti-Thy1 nephritis rats *in vivo*

4 samples/per group

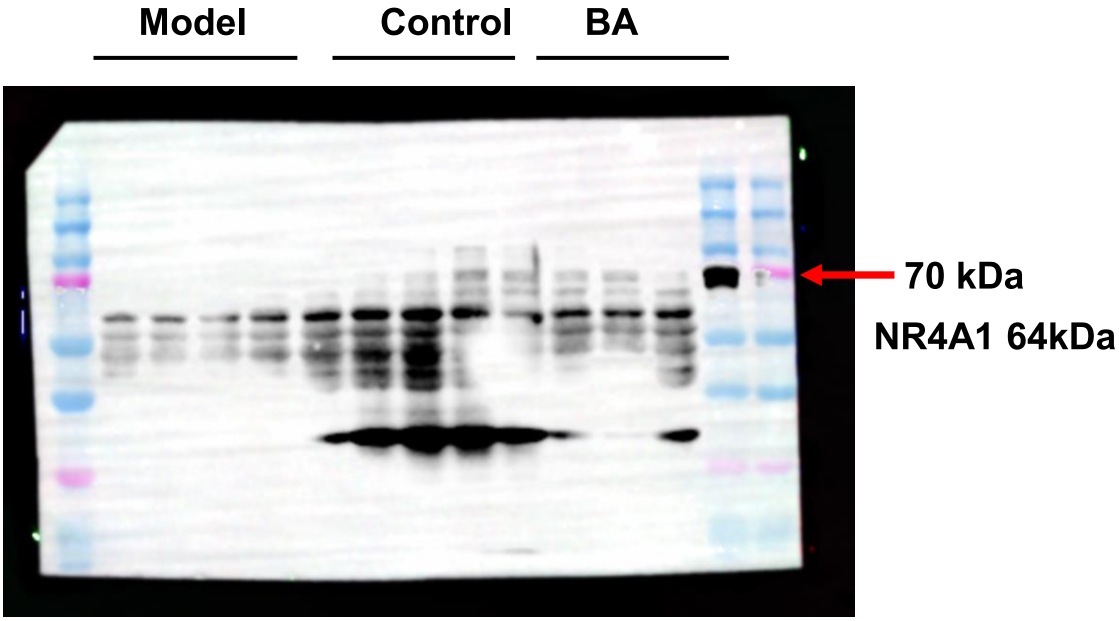

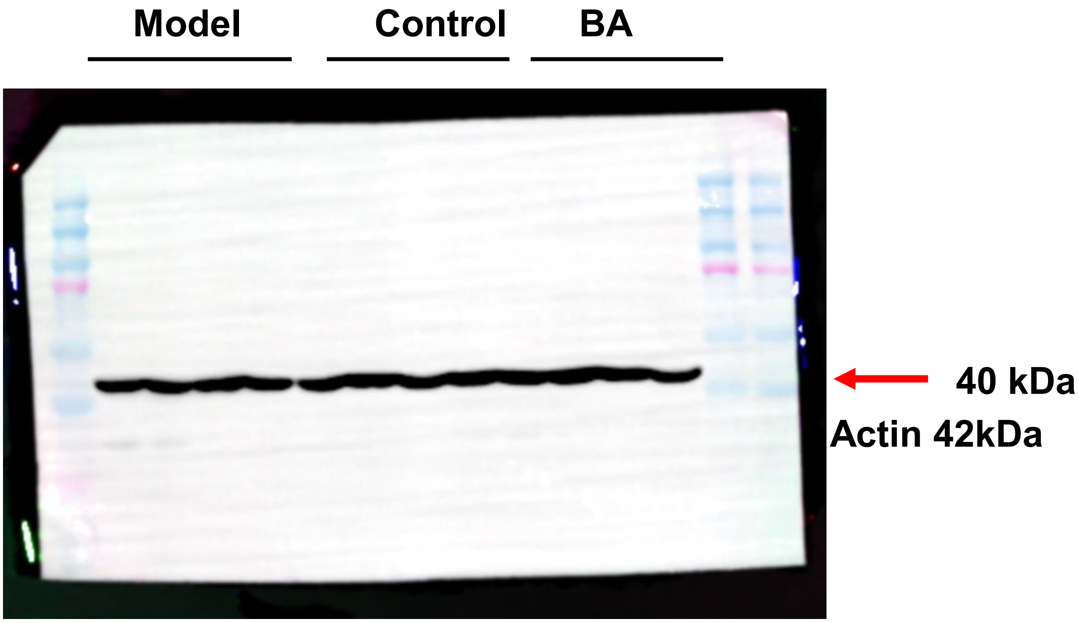

Western blot was performed to evaluate the effects of BA on NR4A1 protein expression in mesangial cell *in vitro*

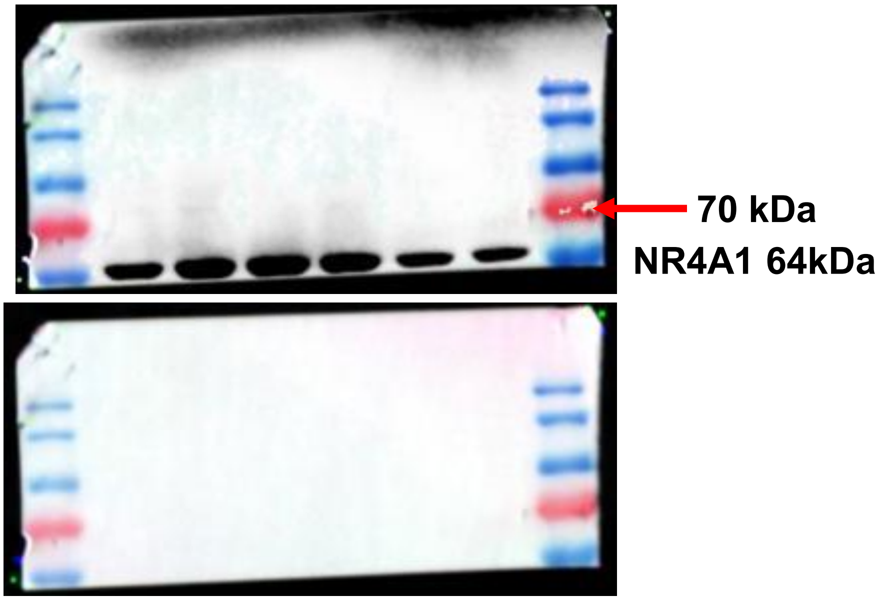

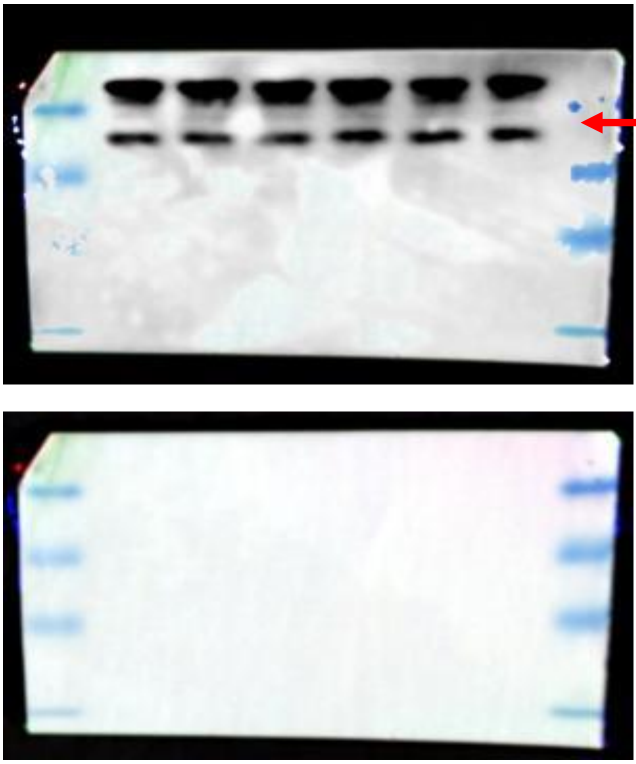

Repeat 1, the merged image

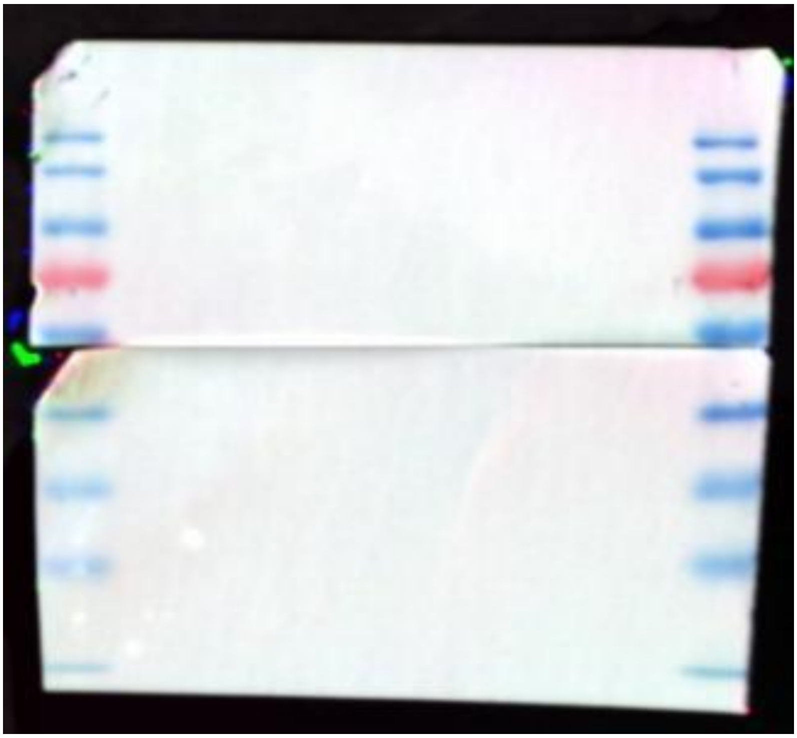

Repeat 2

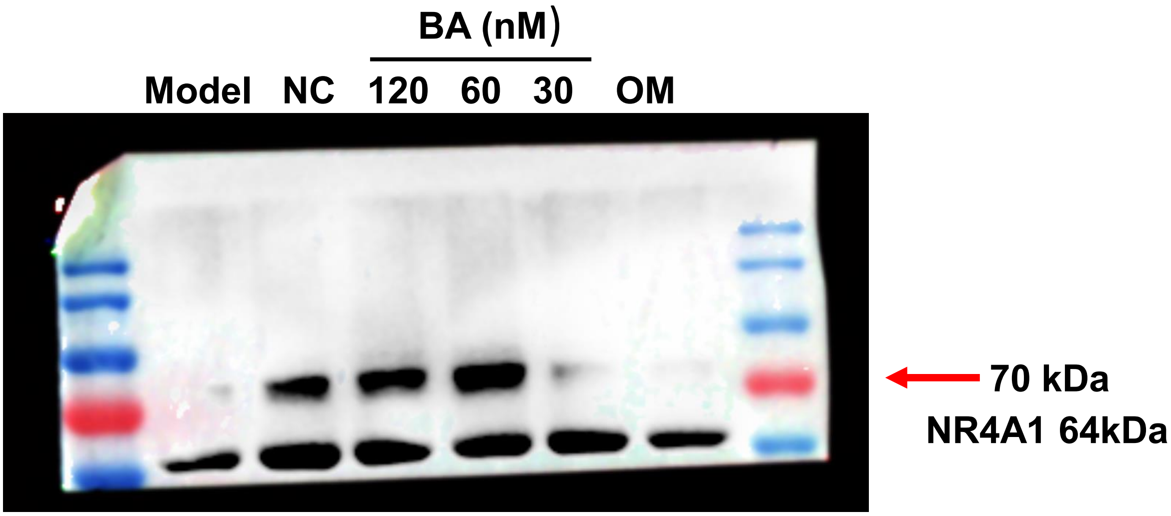

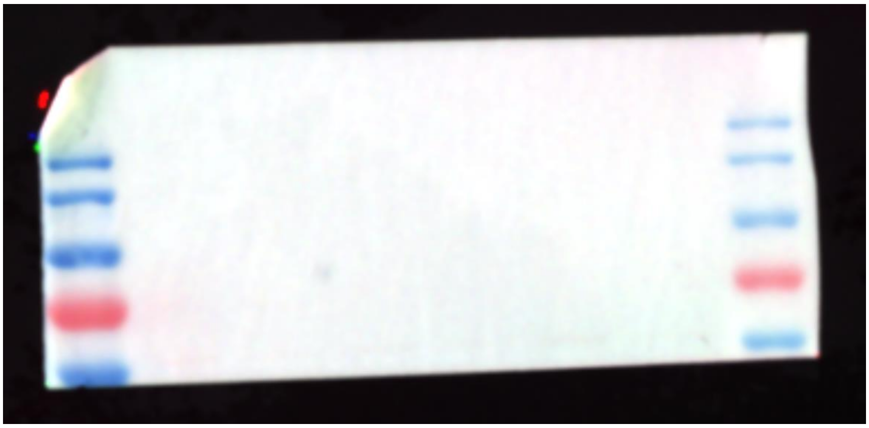

Repeat 2

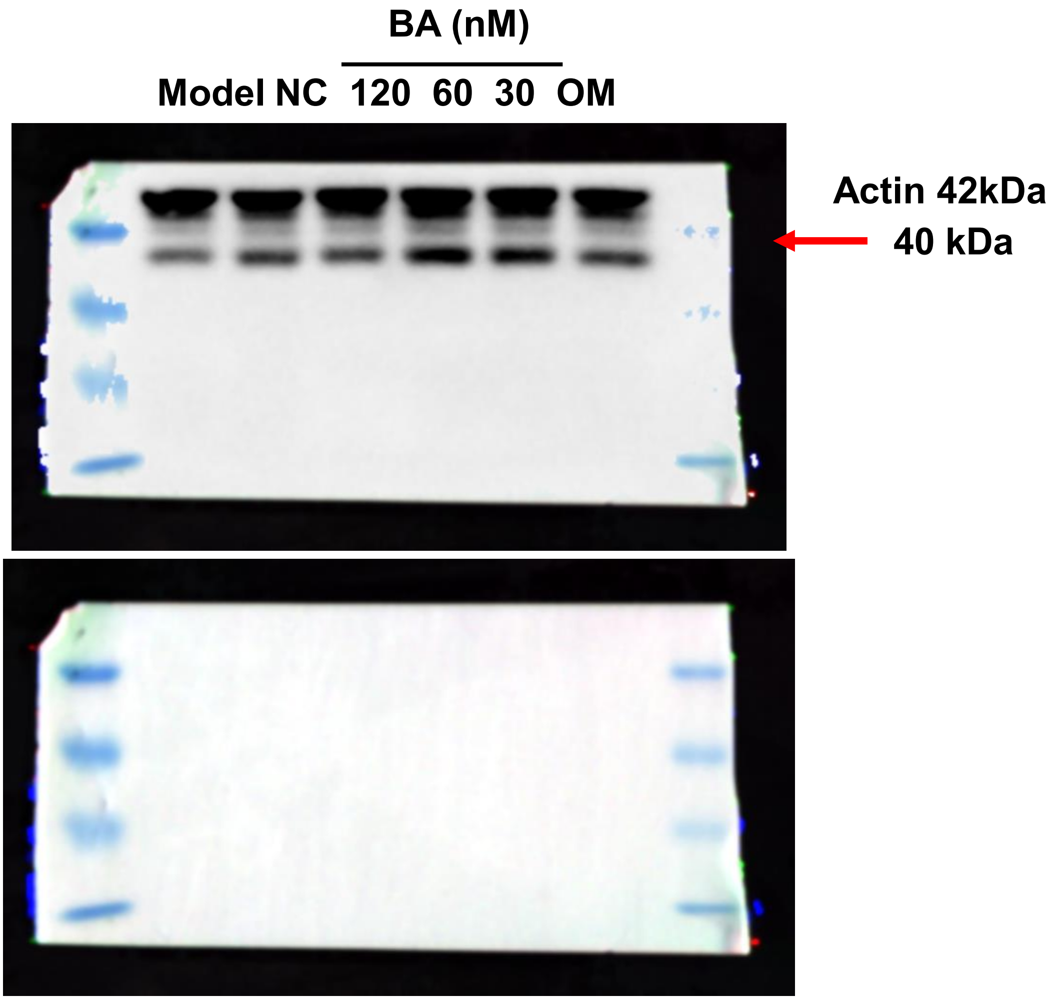

Repeat 2, the merged image

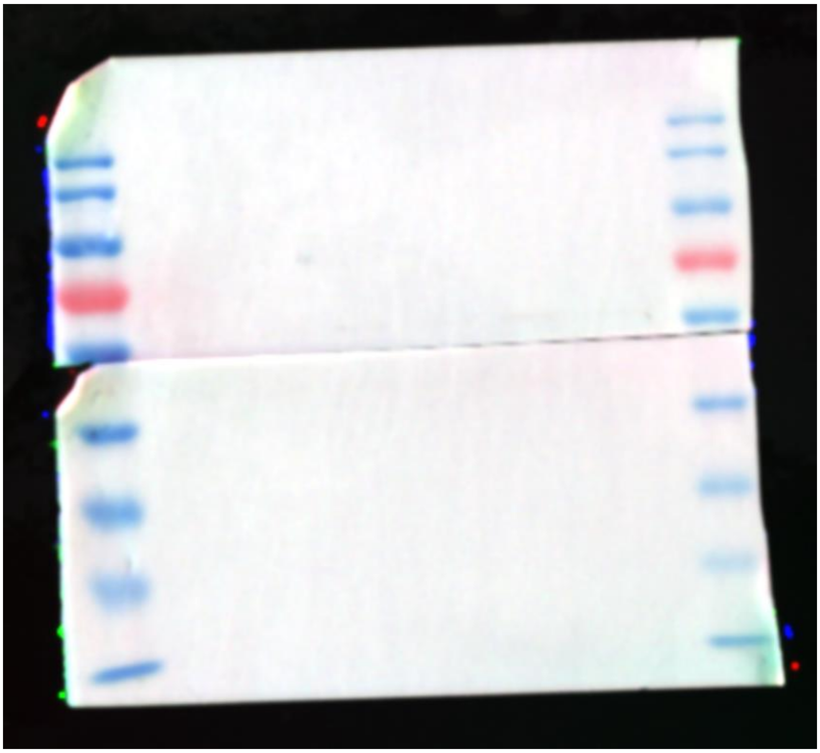

Repeat 3

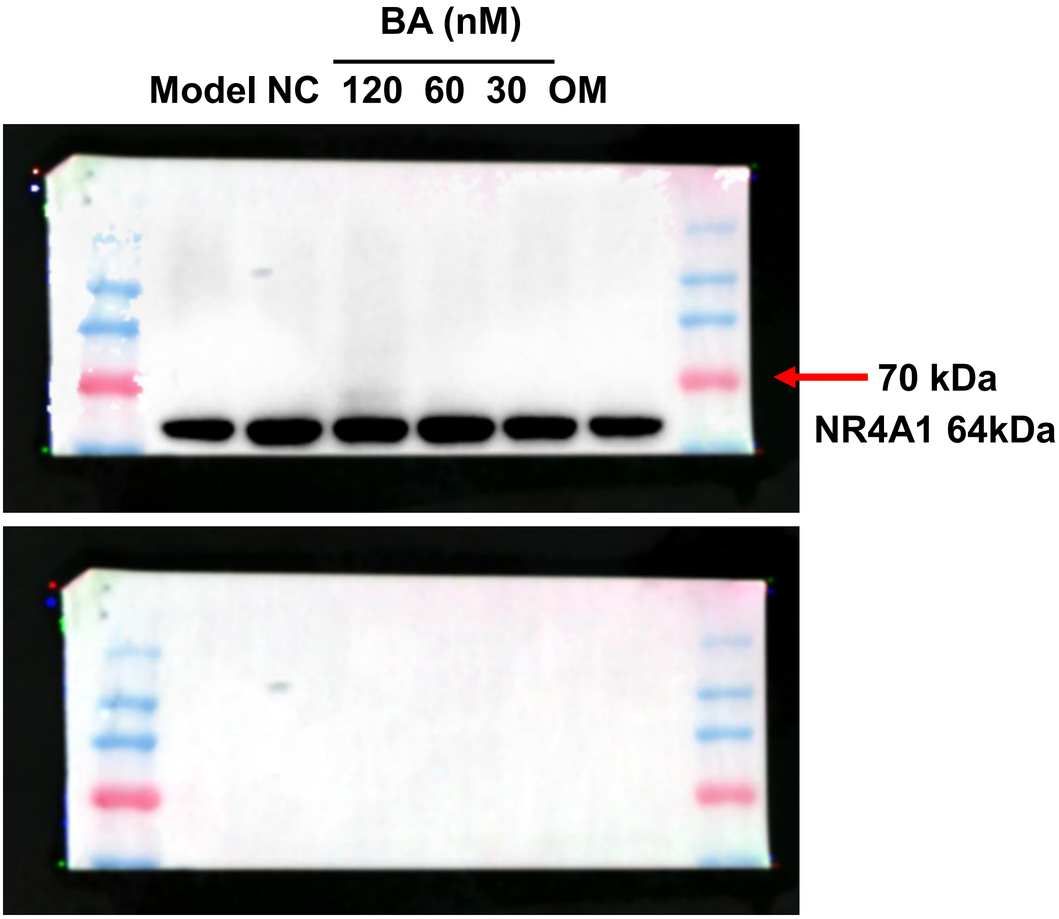

Repeat 3

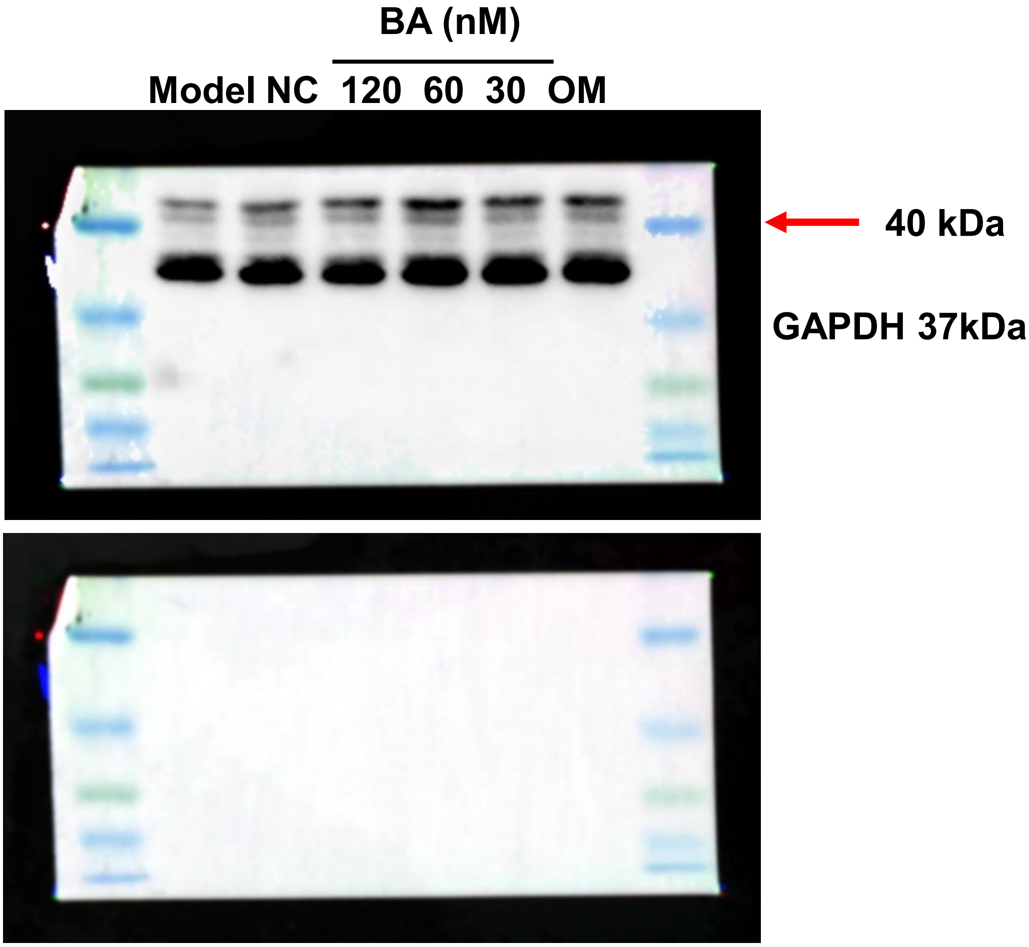

Repeat 3, the merged image

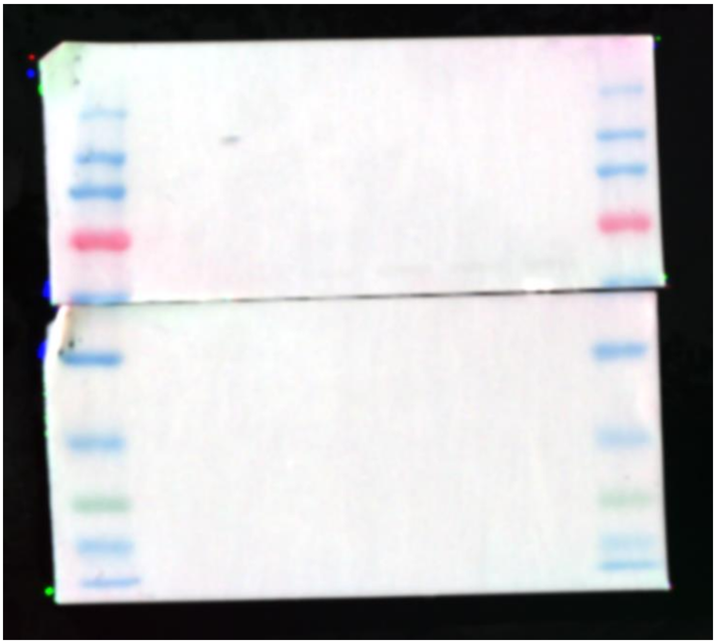

Western blot was performed to evaluate the effects of BA on NF-κB protein expression in mesangial cell *in vitro*

Repeat 1

BA (nM)

Model NC 120 60 30 OM

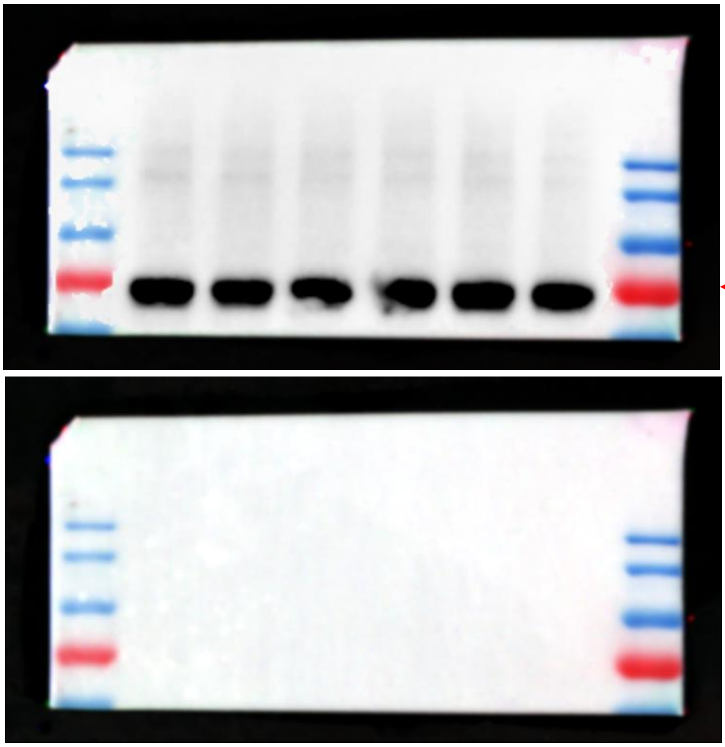

70 kDa

NF-κB 65kDa

Repeat 1

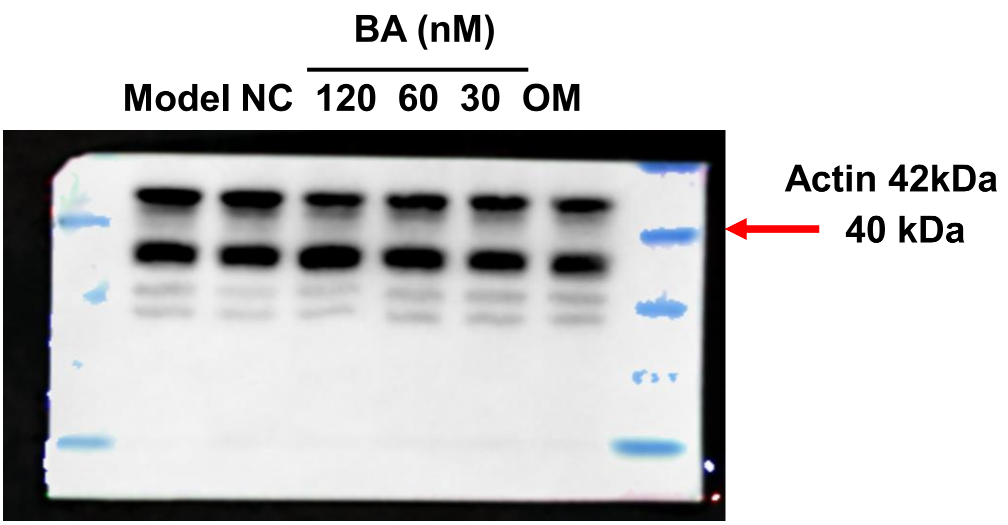

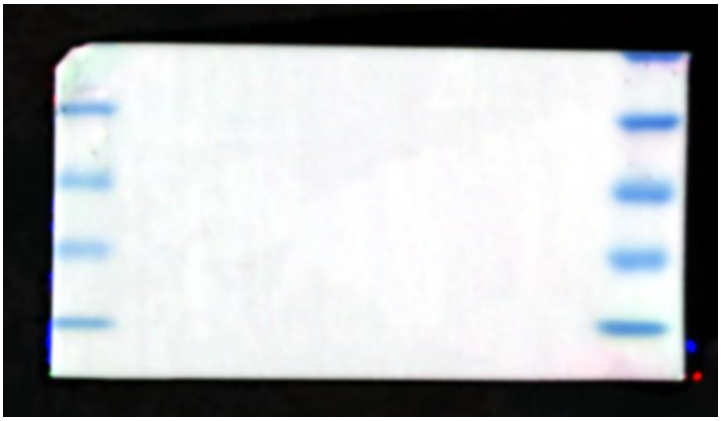

Repeat 1, the merged image

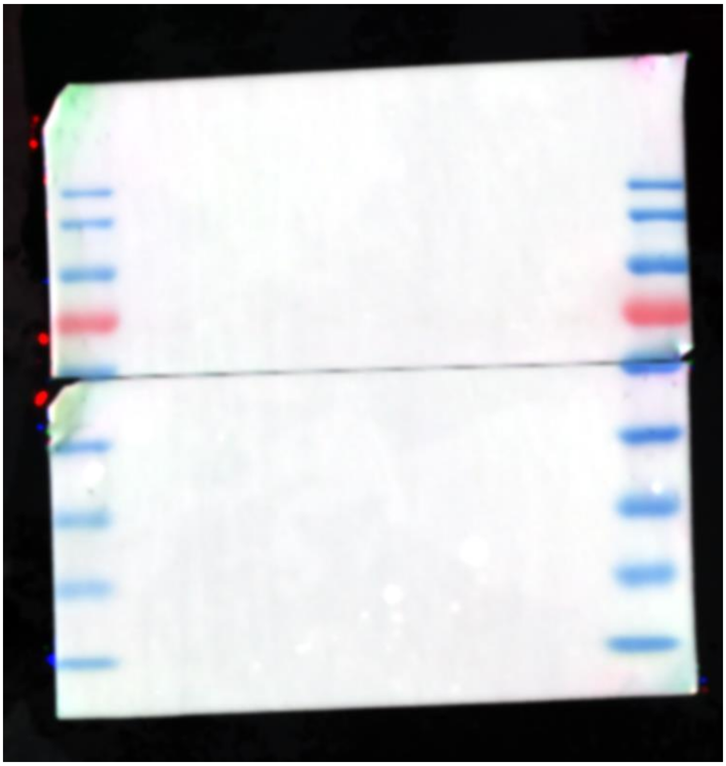

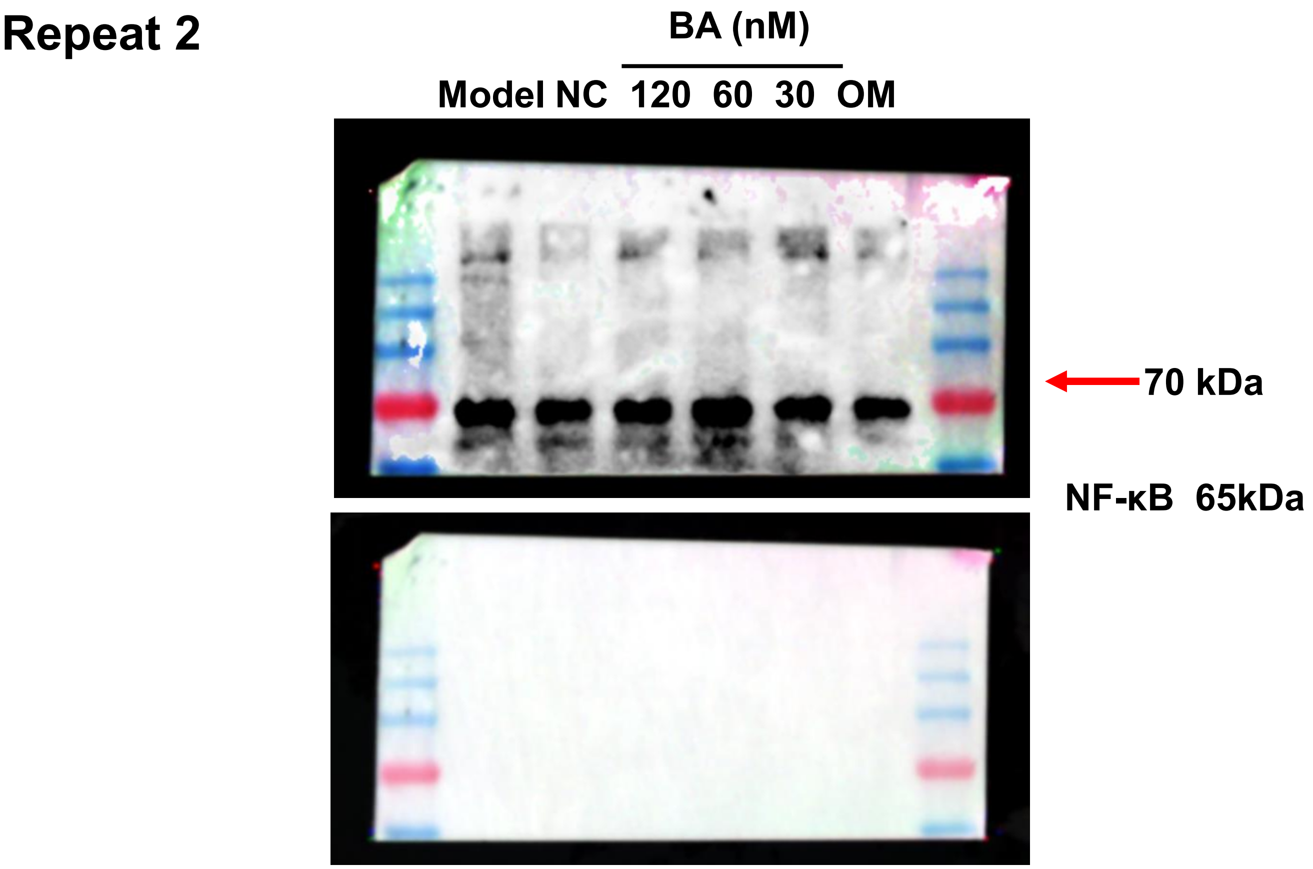
Repeat 2
BA (nM)
Model NC 120 60 30 OM
70 kDa
NF-κB 65kDa

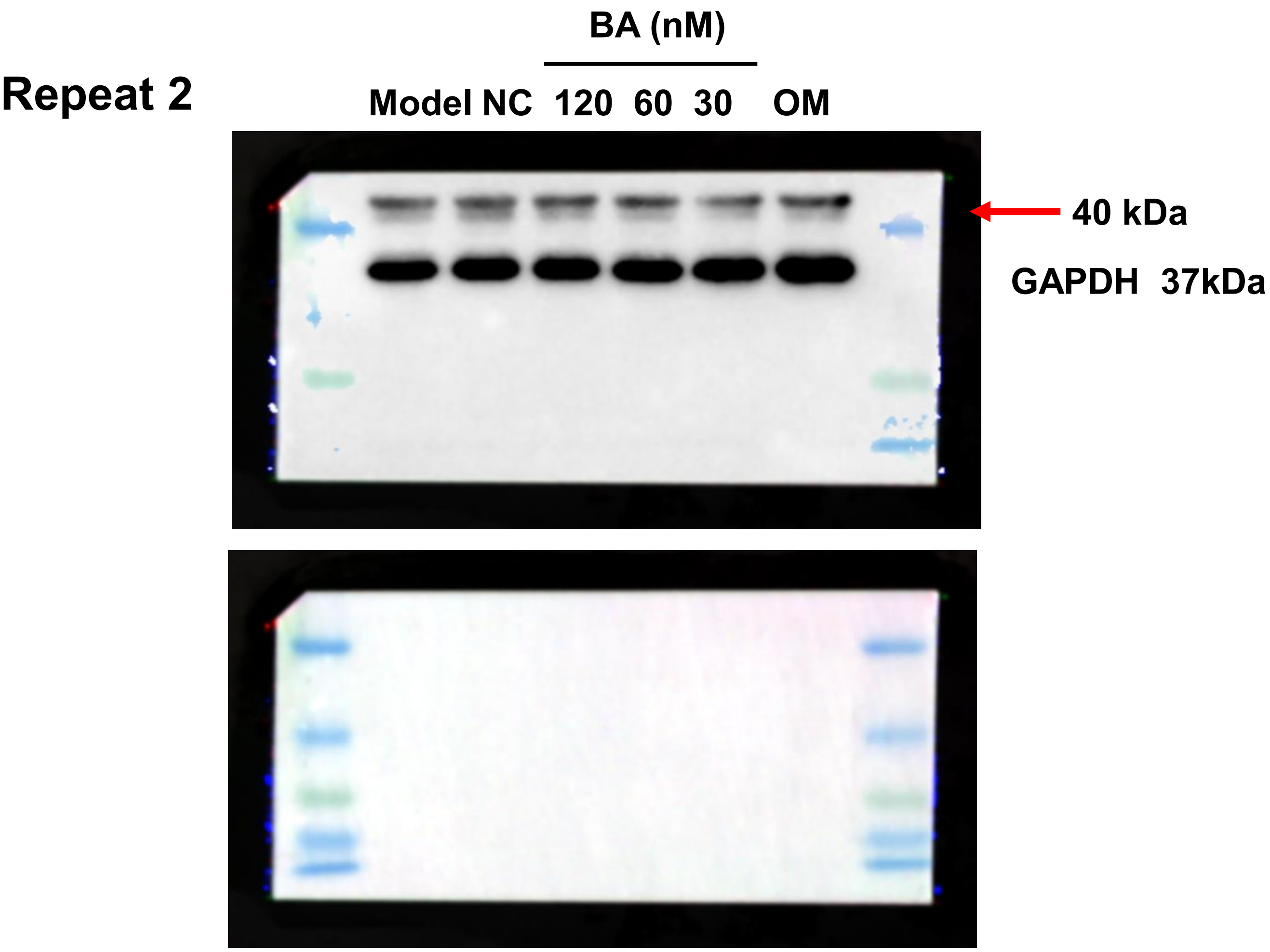
BA (nM)
Repeat 2
Model NC 120 60 30 OM
40 kDa
GAPDH 37kDa

Repeat 2, the merged image

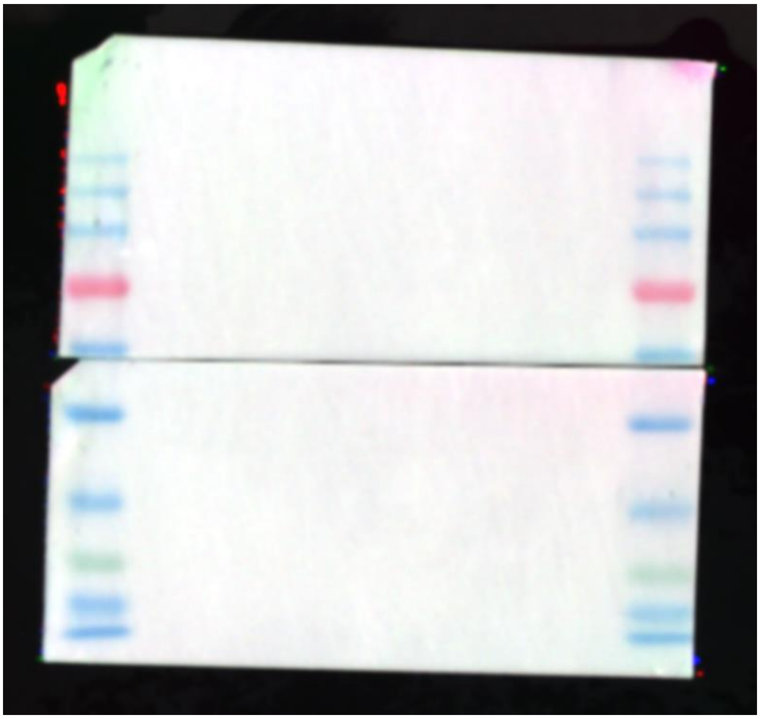

Repeat 3

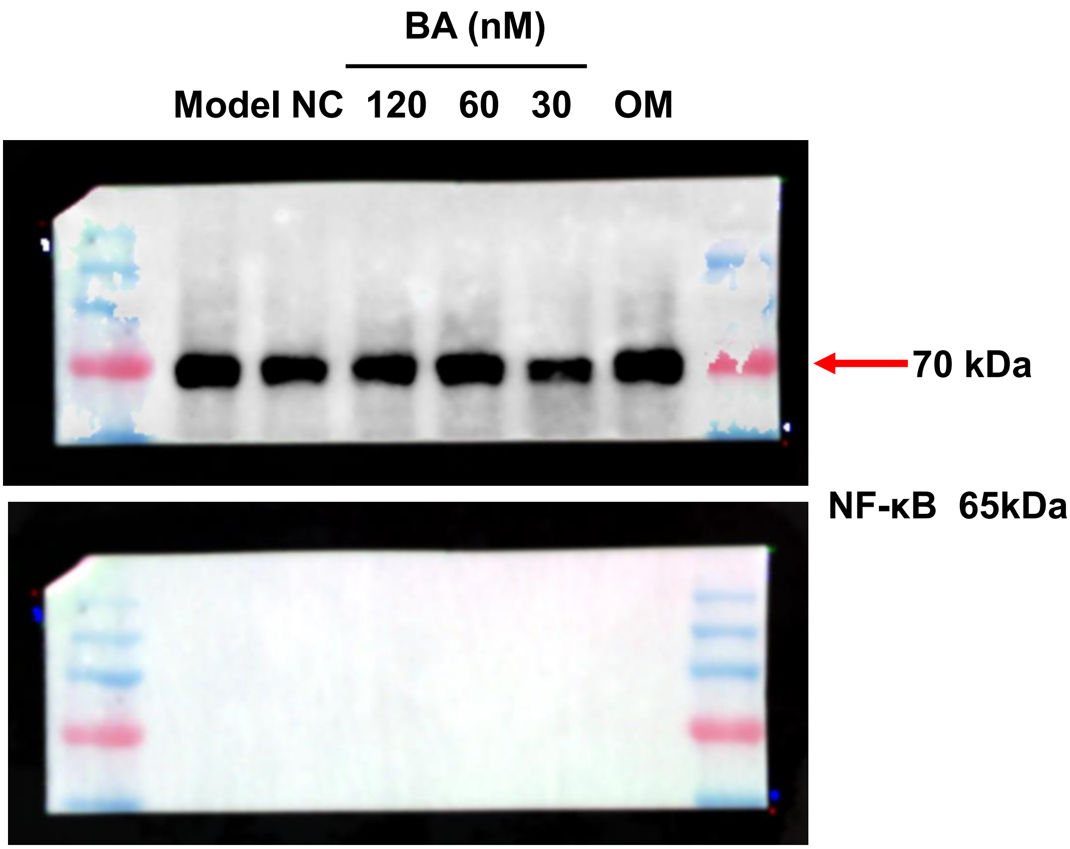

Repeat 3

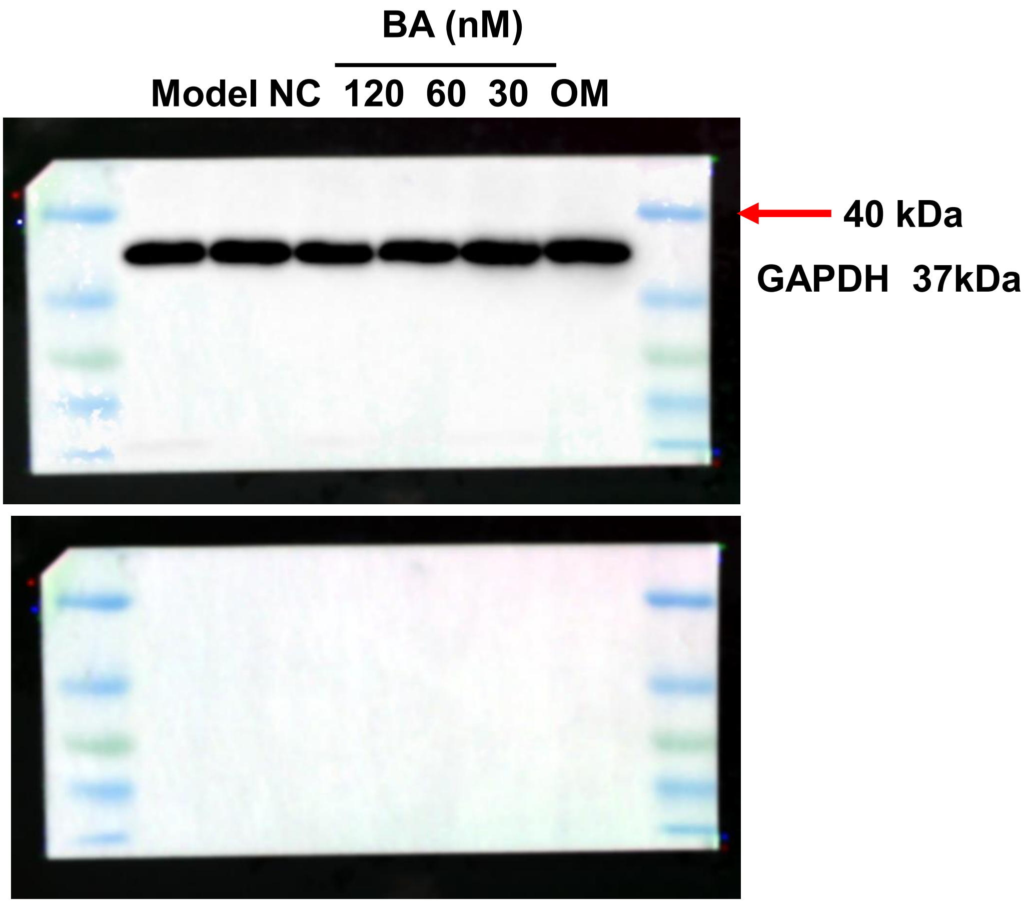

Repeat 3, the merged image

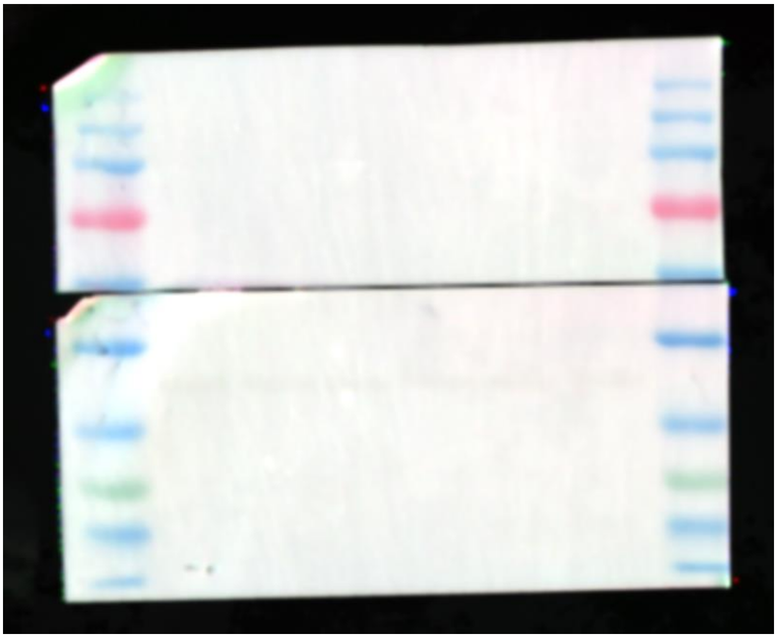

Western blot was performed to evaluate the effects of BA on p- NF-κB protein expression in mesangial cell *in vitro*

Repeat 1

BA (nM)

Model NC 120 60 30 OM

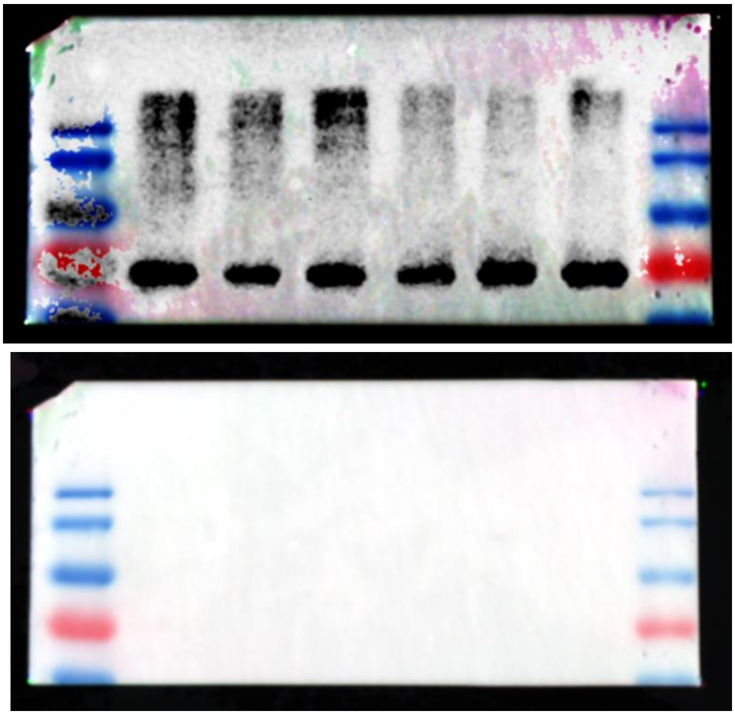

70 kDa

p-NF-κB 65kDa

Repeat 1

BA (nM)

Model NC 120 60 30 OM

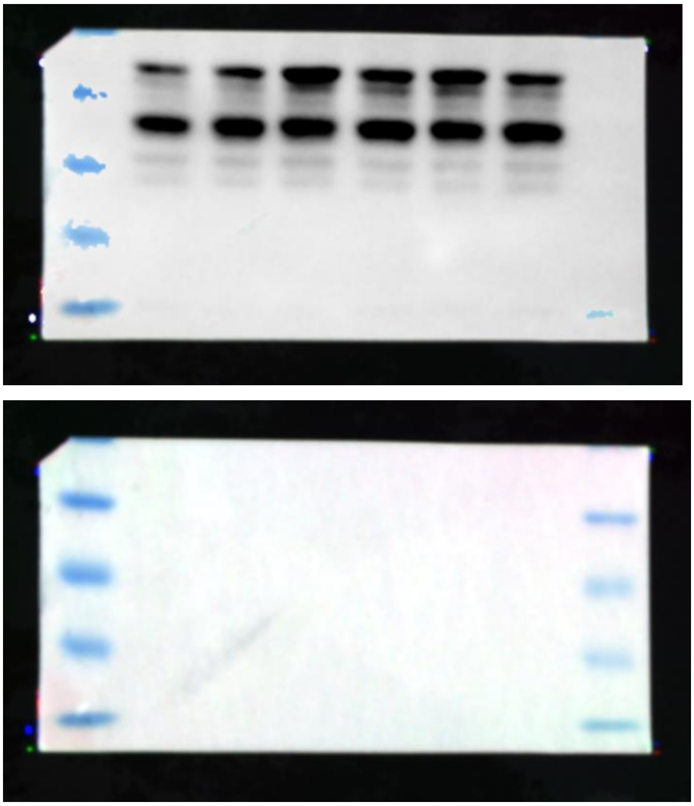

40 kDa

GAPDH 37kDa

Repeat 1, the merged image

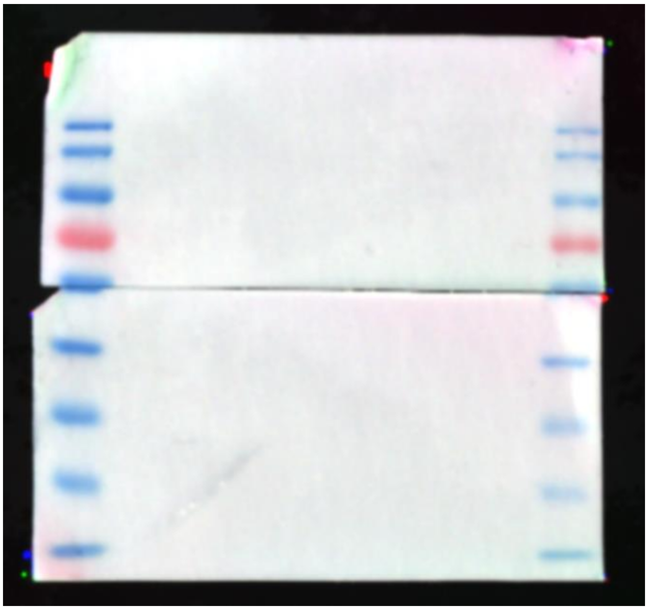

Repeat 2

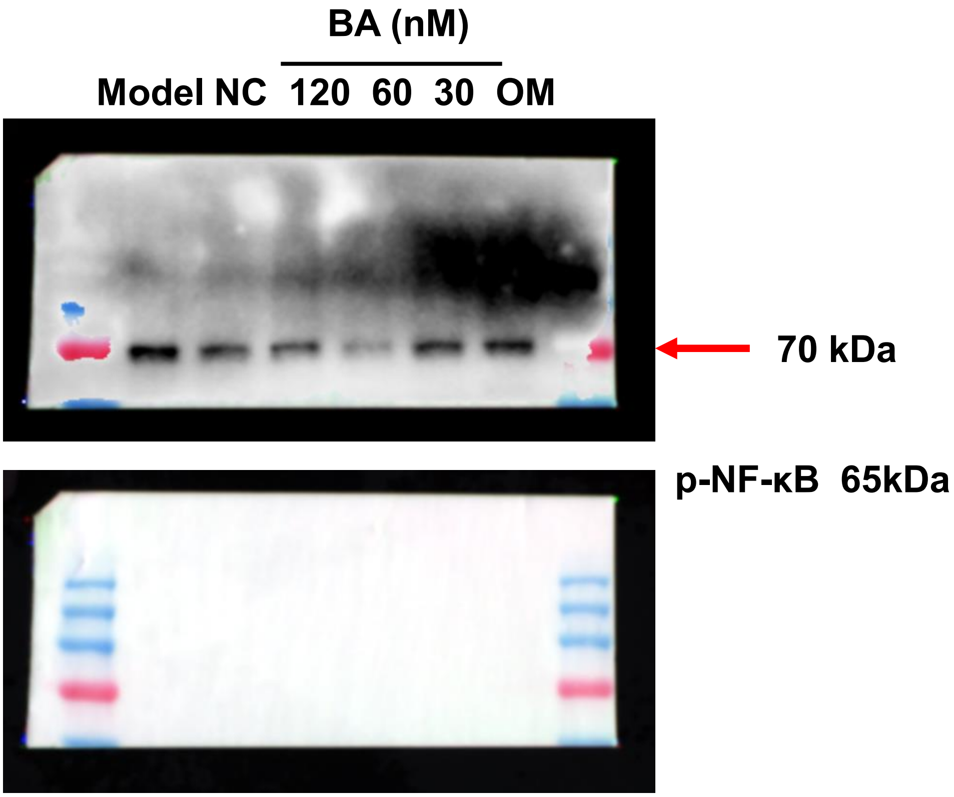

Repeat 2

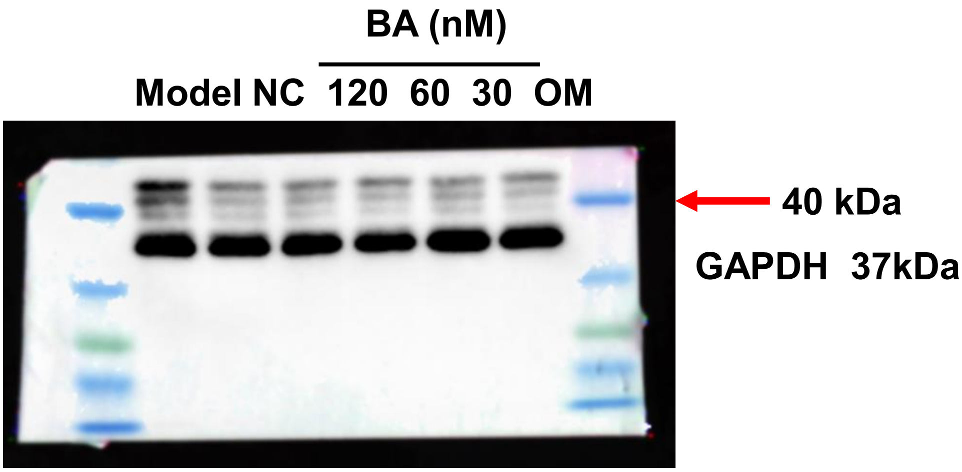

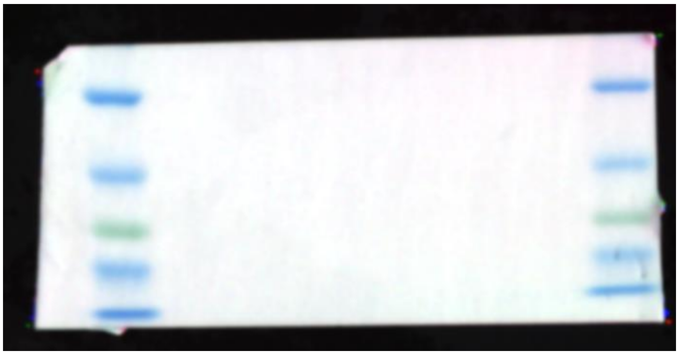

Repeat 2, the merged image

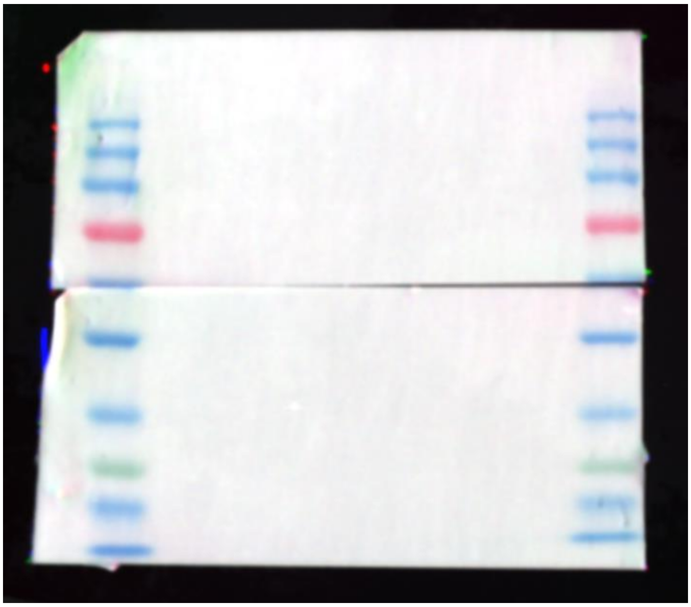

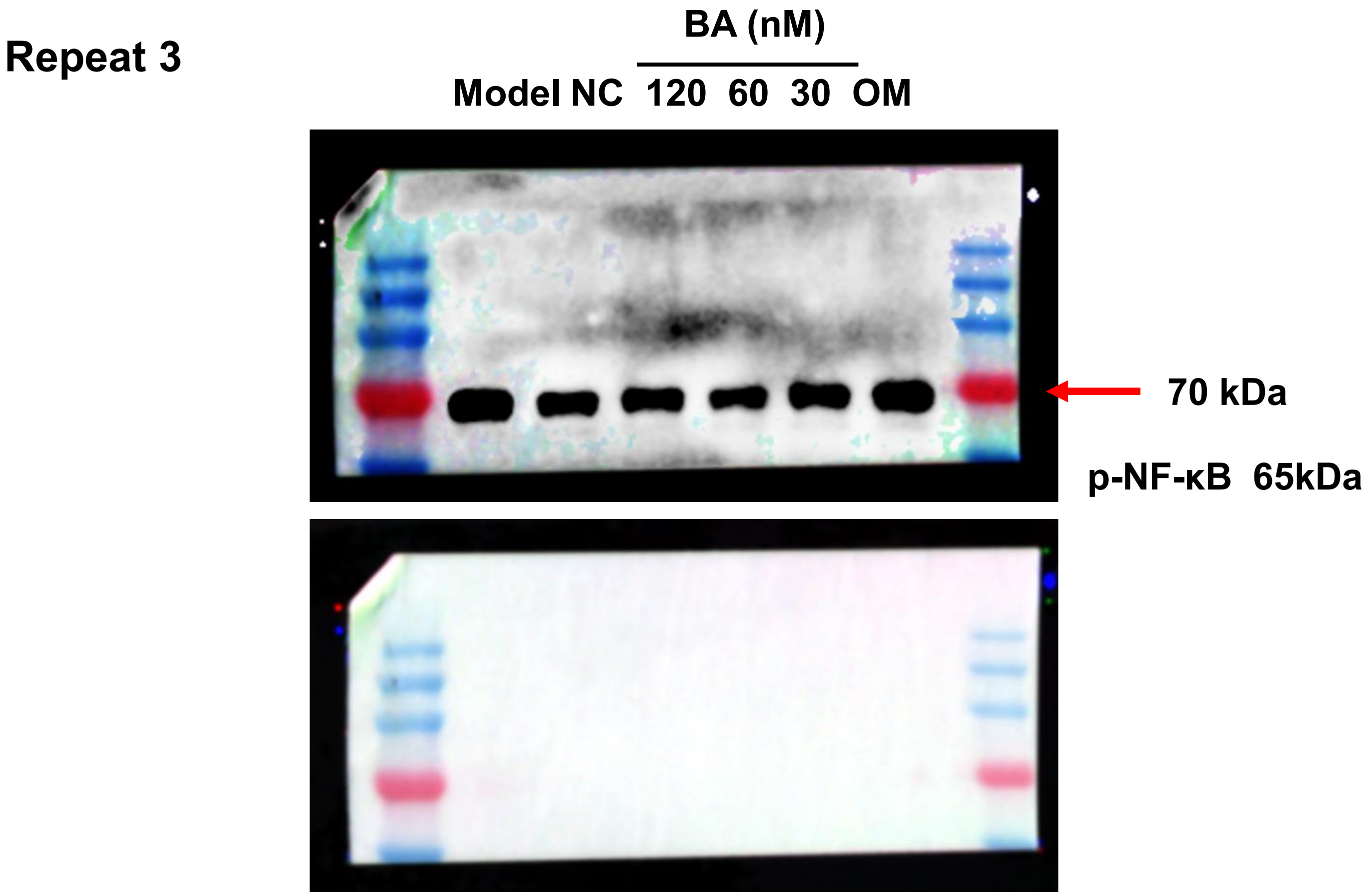
Repeat 3
BA (nM)
Model NC 120 60 30 OM
70 kDa
p-NF-κB 65kDa

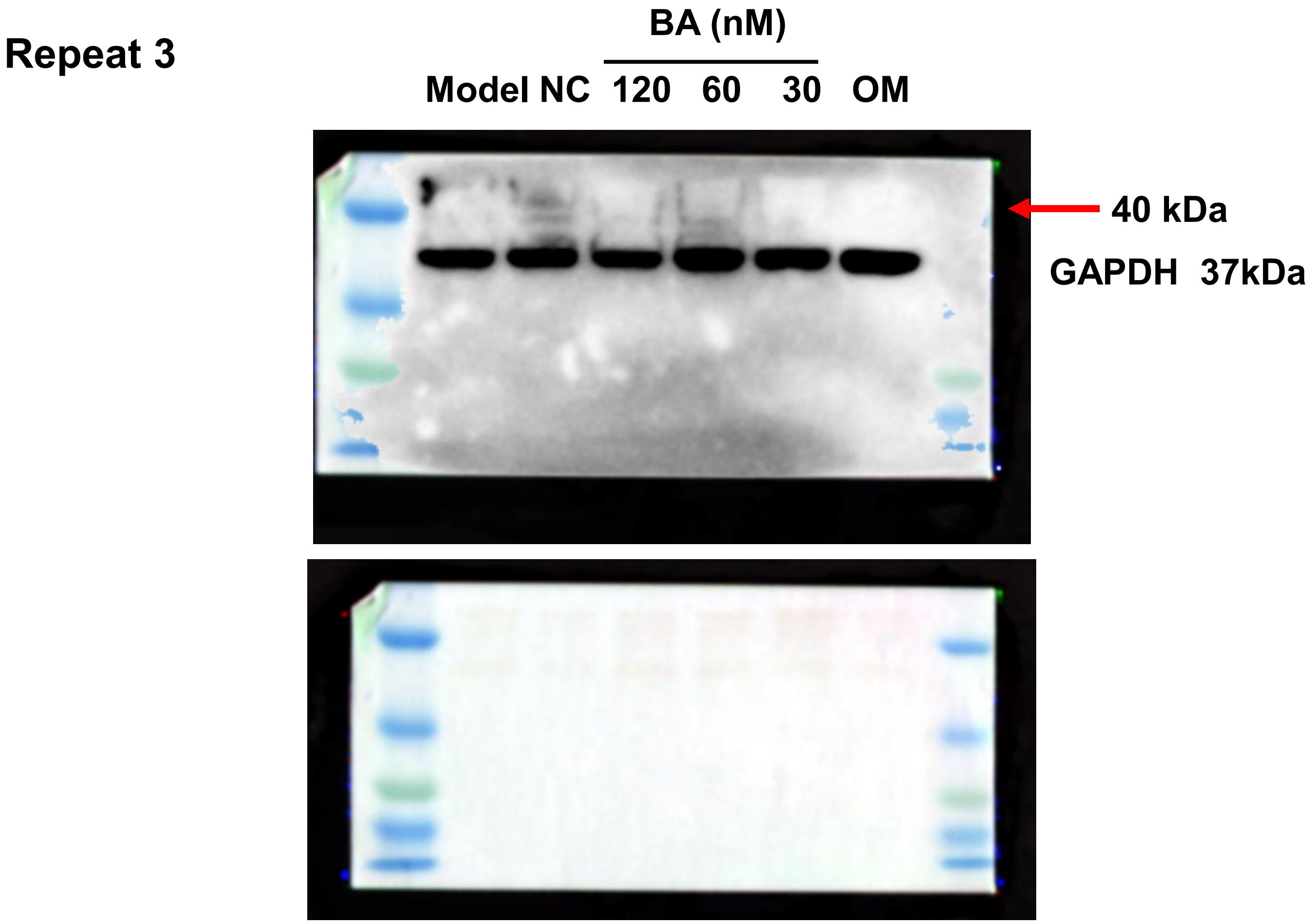
Repeat 3
BA (nM)
Model NC 120 60 30 OM
40 kDa
GAPDH 37kDa

Western blot was performed to evaluate the effects of BA or CsnB on NR4A1 protein expression in mesangial cell *in vitro*

Repeat 1

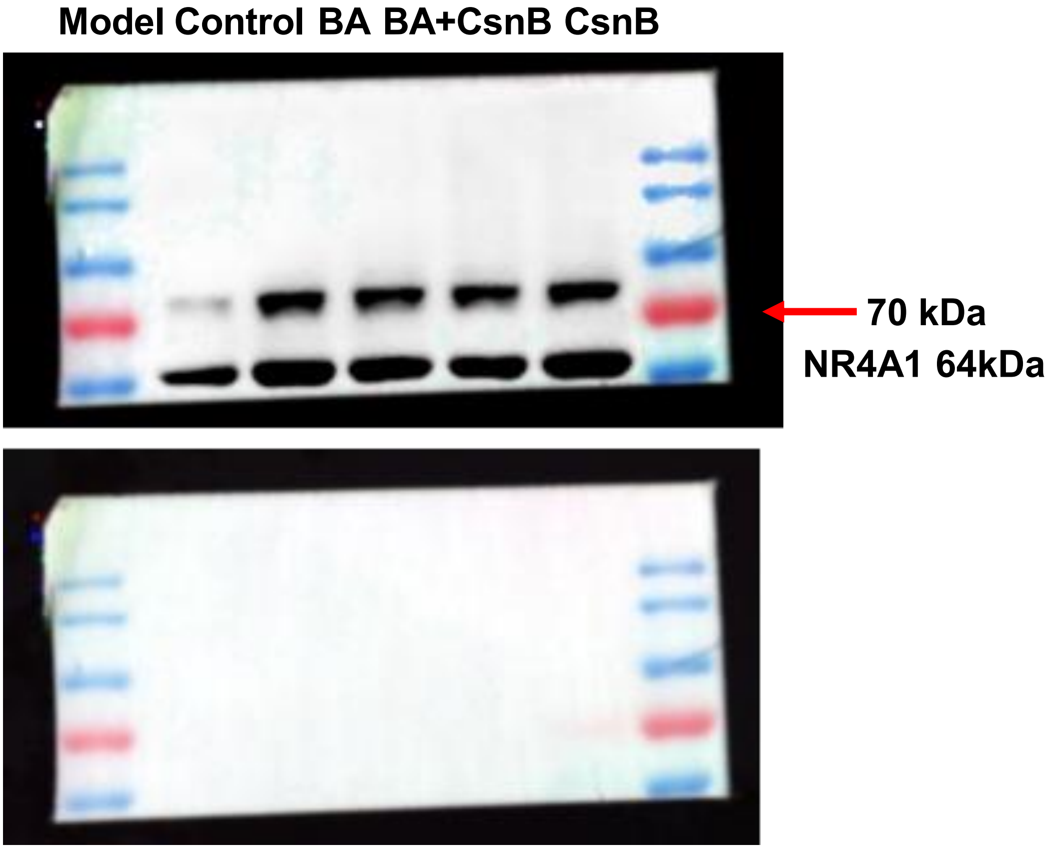

Repeat 1

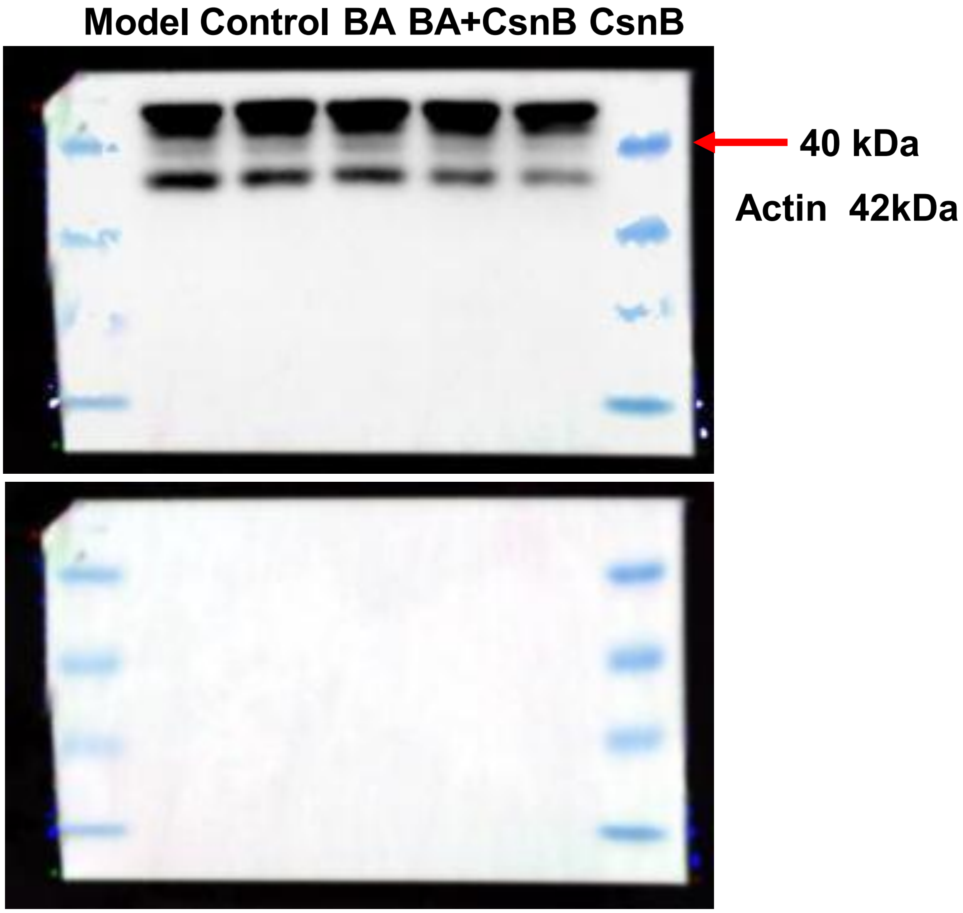

Repeat 2

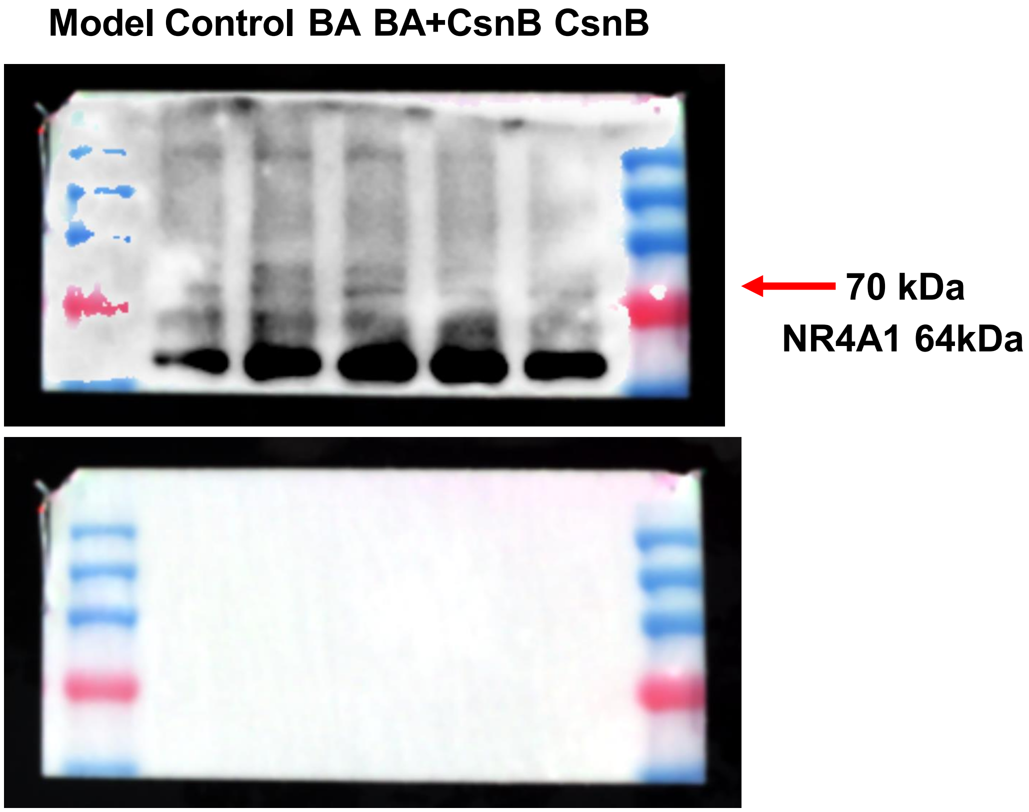

Repeat 2

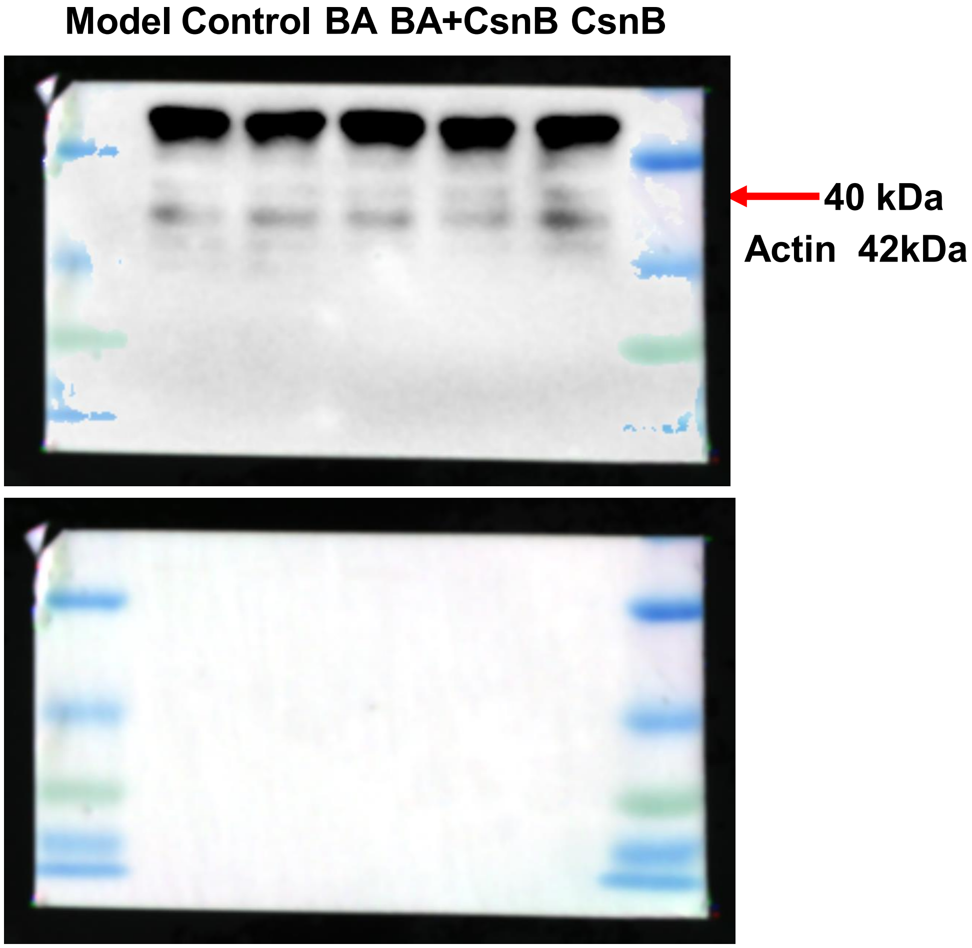

Repeat 2, the merged image

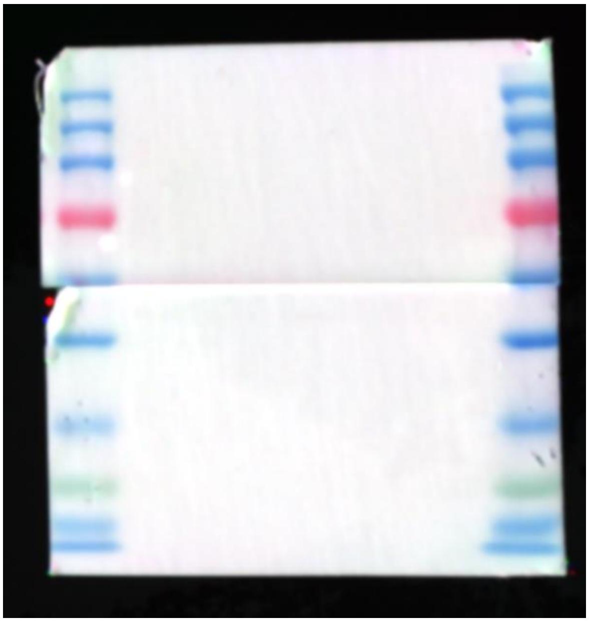

Repeat 3

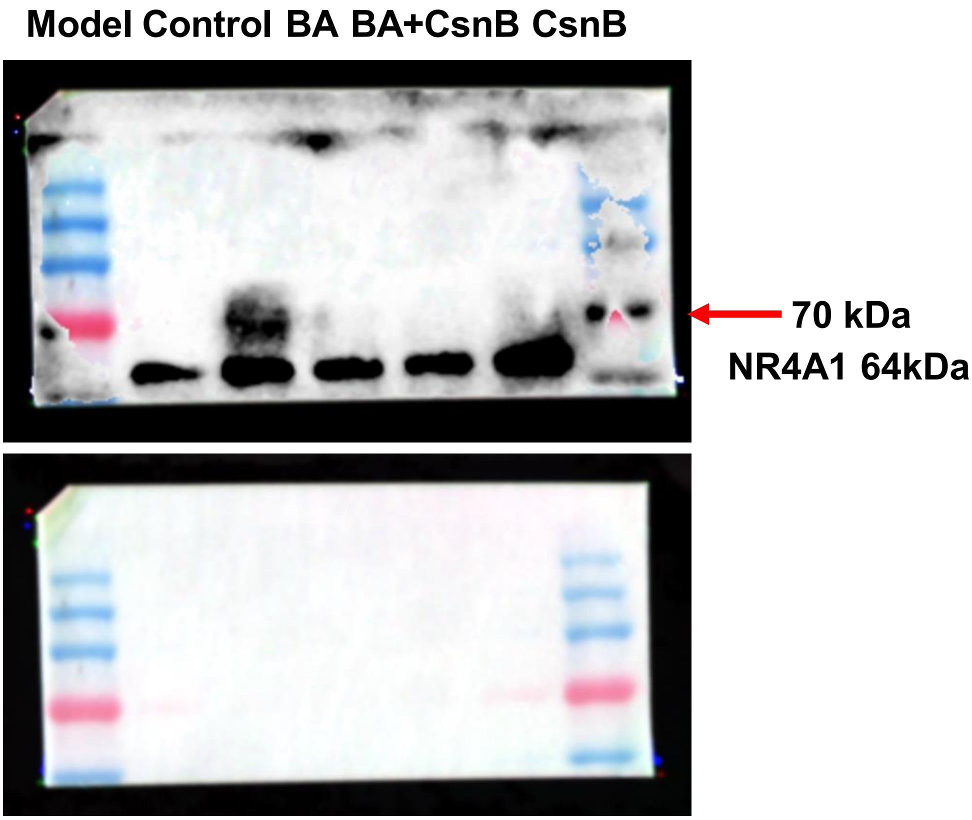

Repeat 3

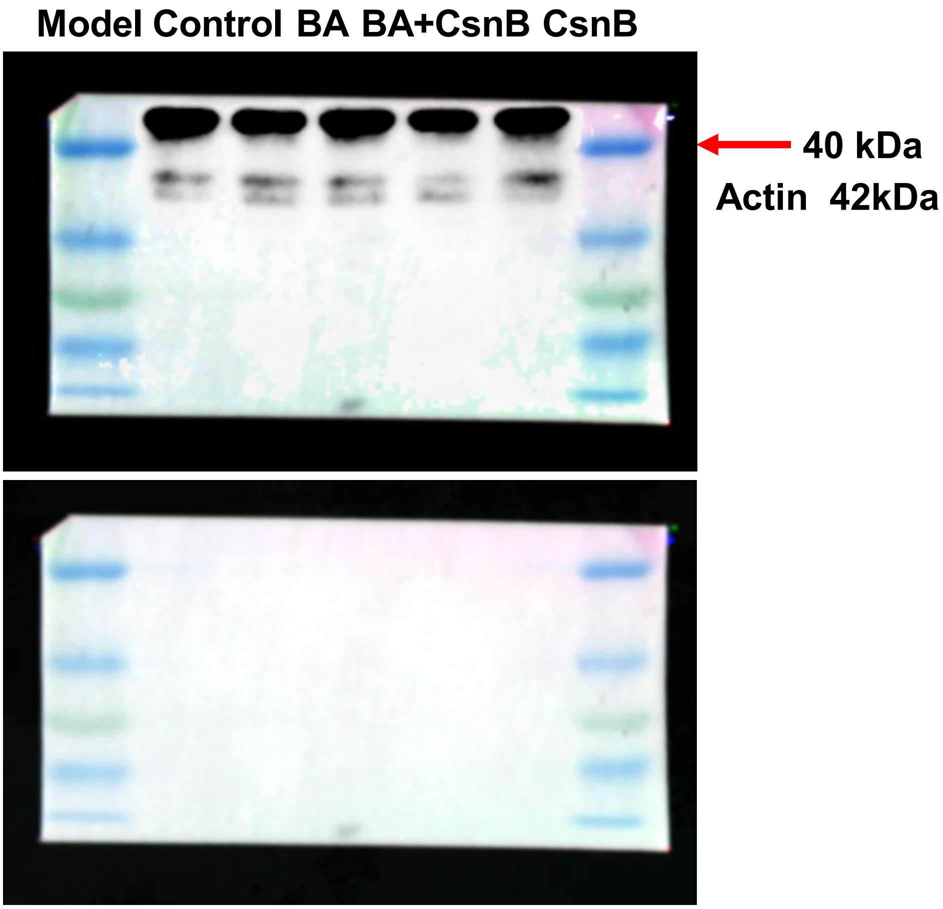

Repeat 3, the merged image

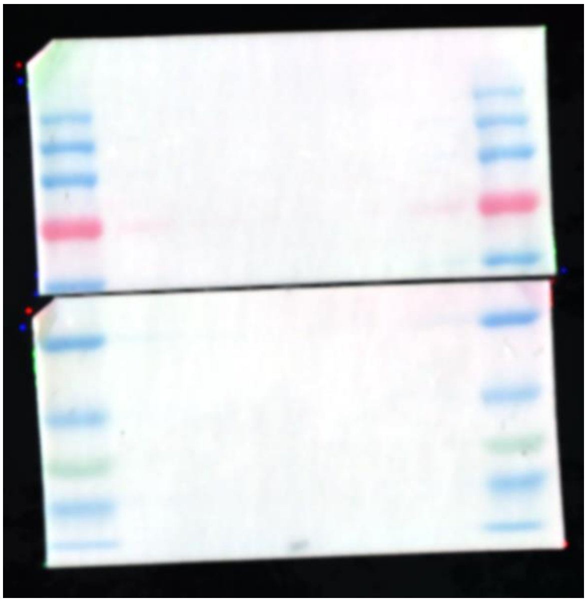

Western blot was performed to evaluate the effects of BA or CsnB on NF-κB protein expression in mesangial cell *in vitro*

Repeat 1

Model Control BA BA+CsnB CsnB

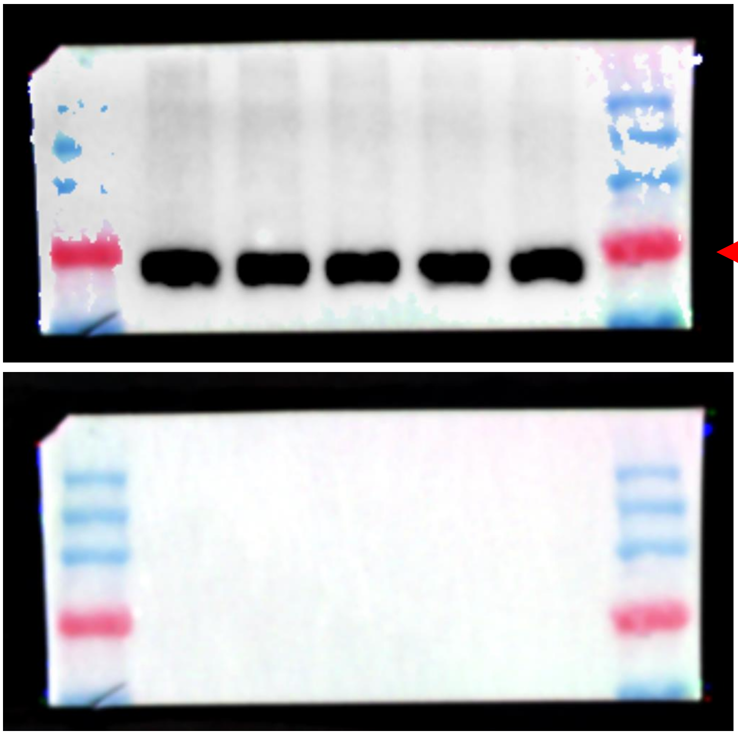

70 kDa

NF-κB 65kDa

Repeat 1

Model Control BA BA+CsnB CsnB

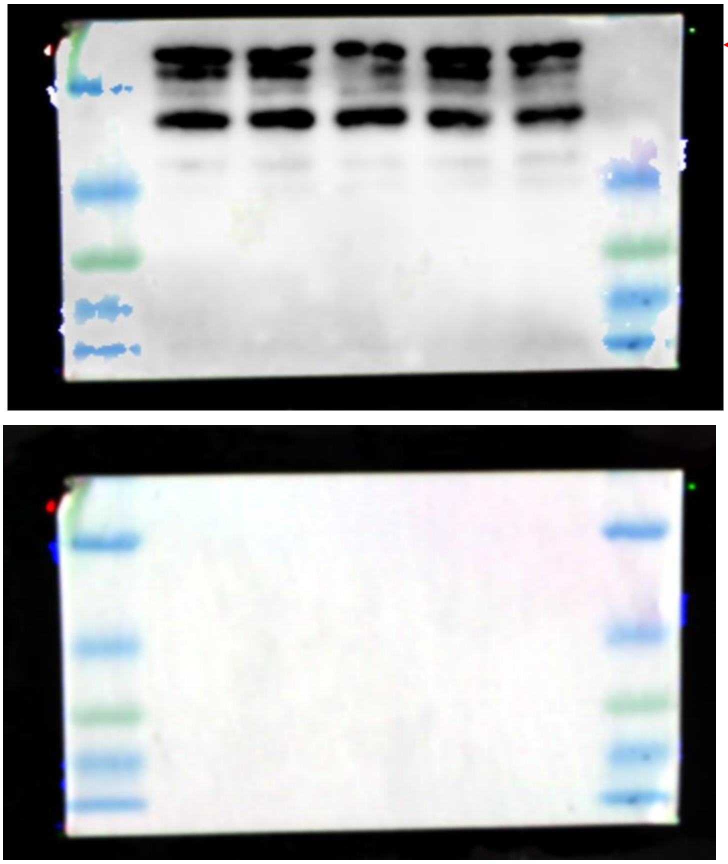

40 kDa

GAPDH 37kDa

Repeat 1, the merged image

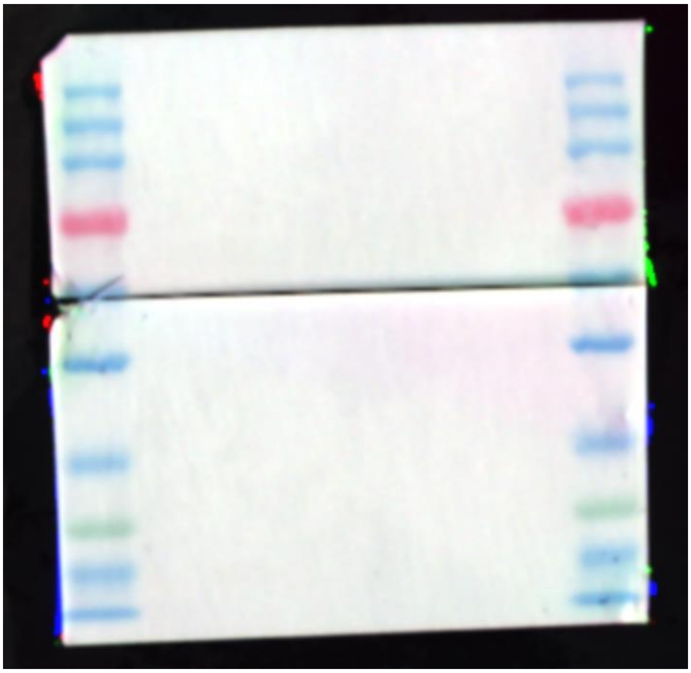

Repeat 2

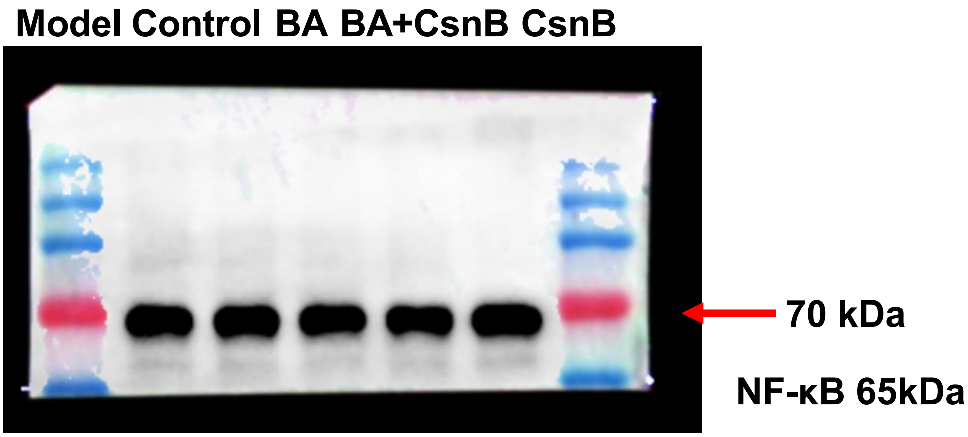

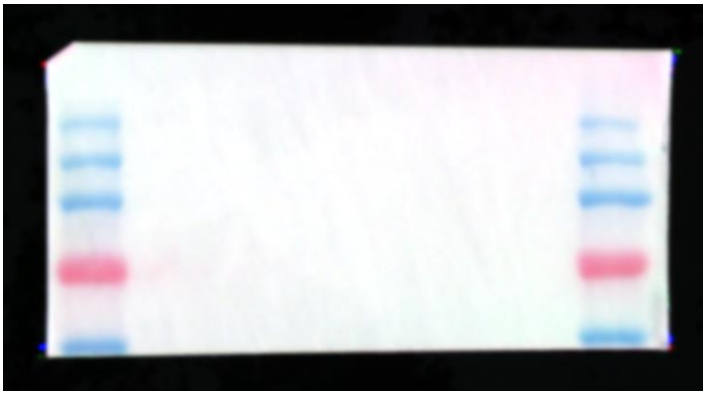

Repeat 2

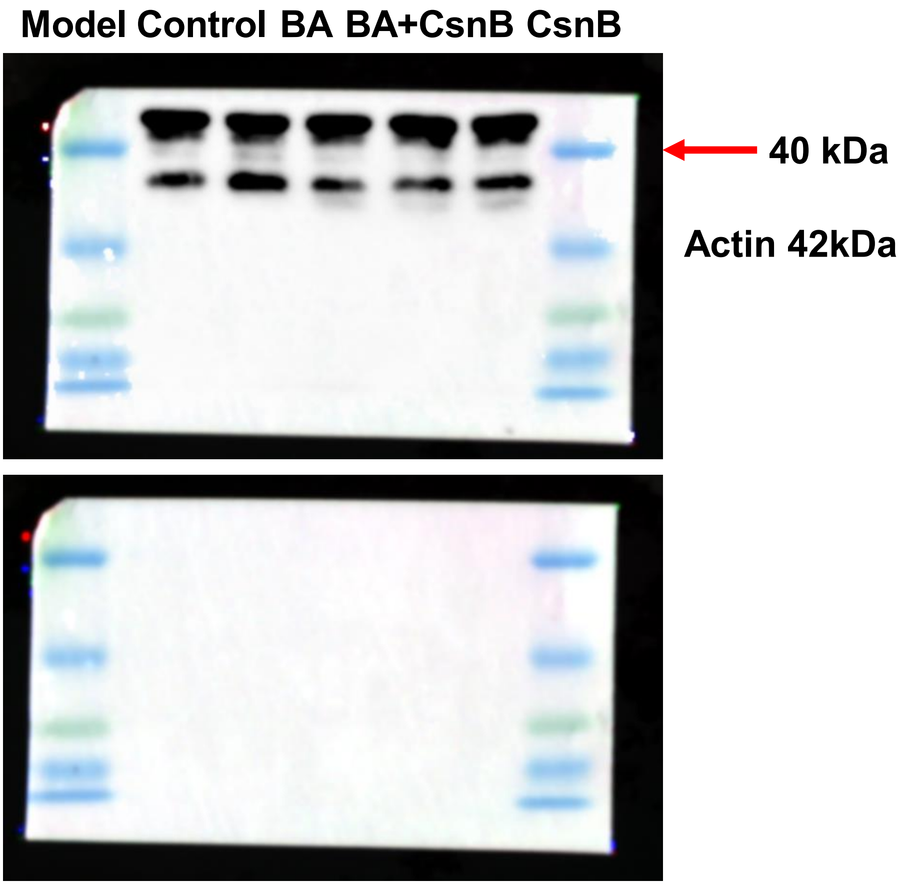

Repeat 2, the merged image

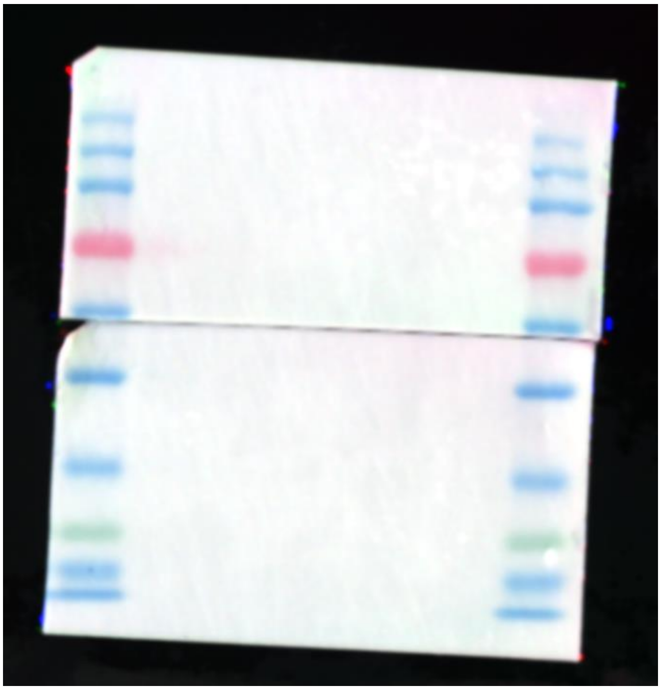

Repeat 3

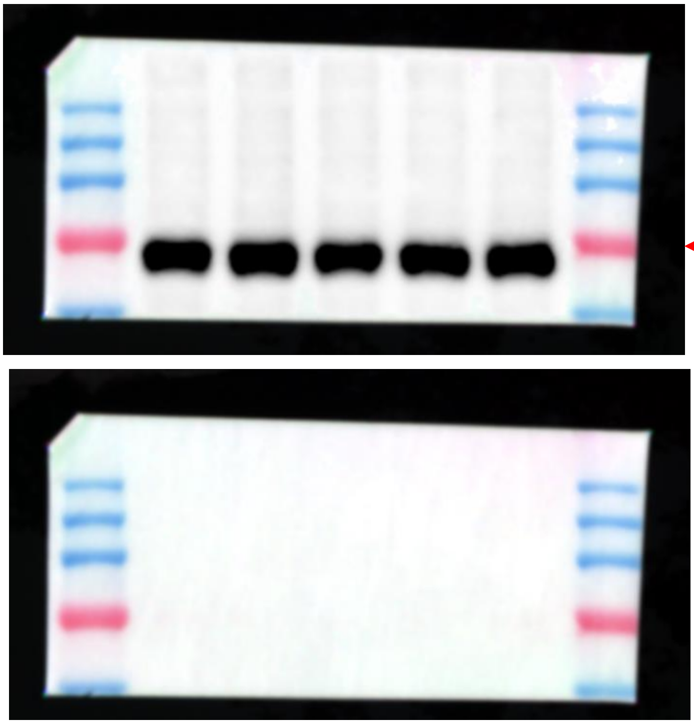

Repeat 3

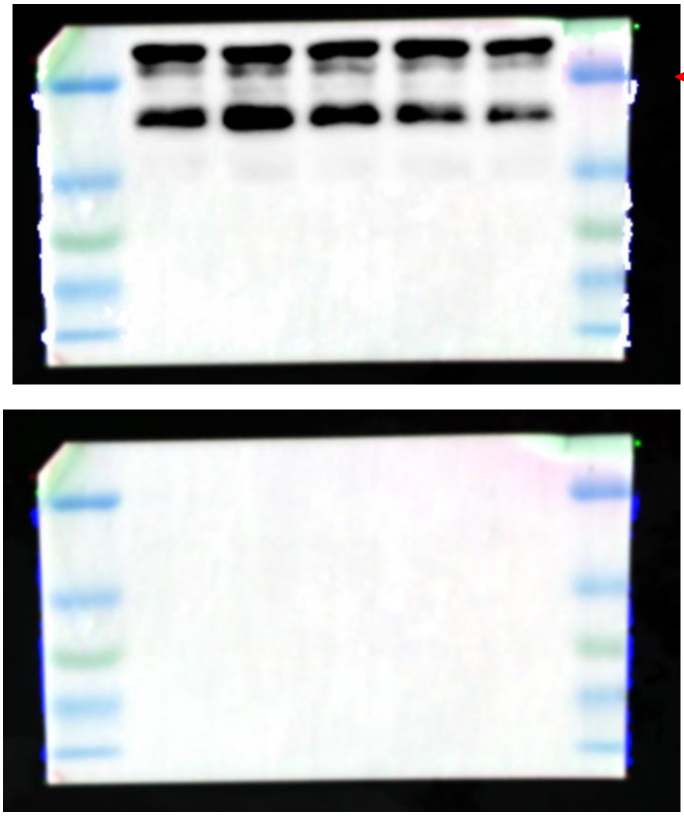

Repeat 3, the merged image

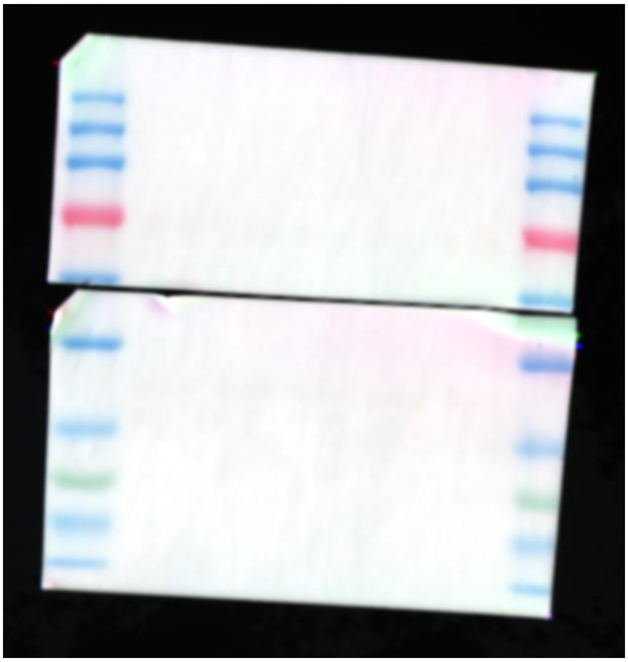

Western blot was performed to evaluate the effects of BA or CsnB on p-NF-κB protein expression in mesangial cell *in vitro*

Repeat 1

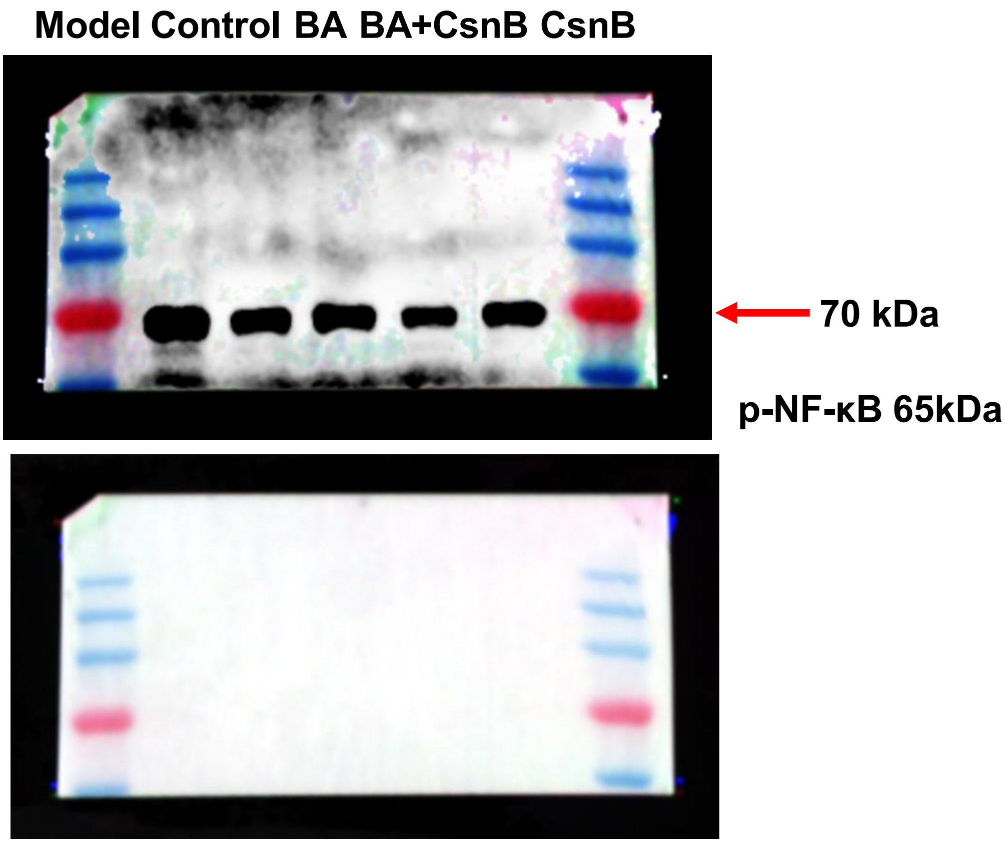

Repeat 1

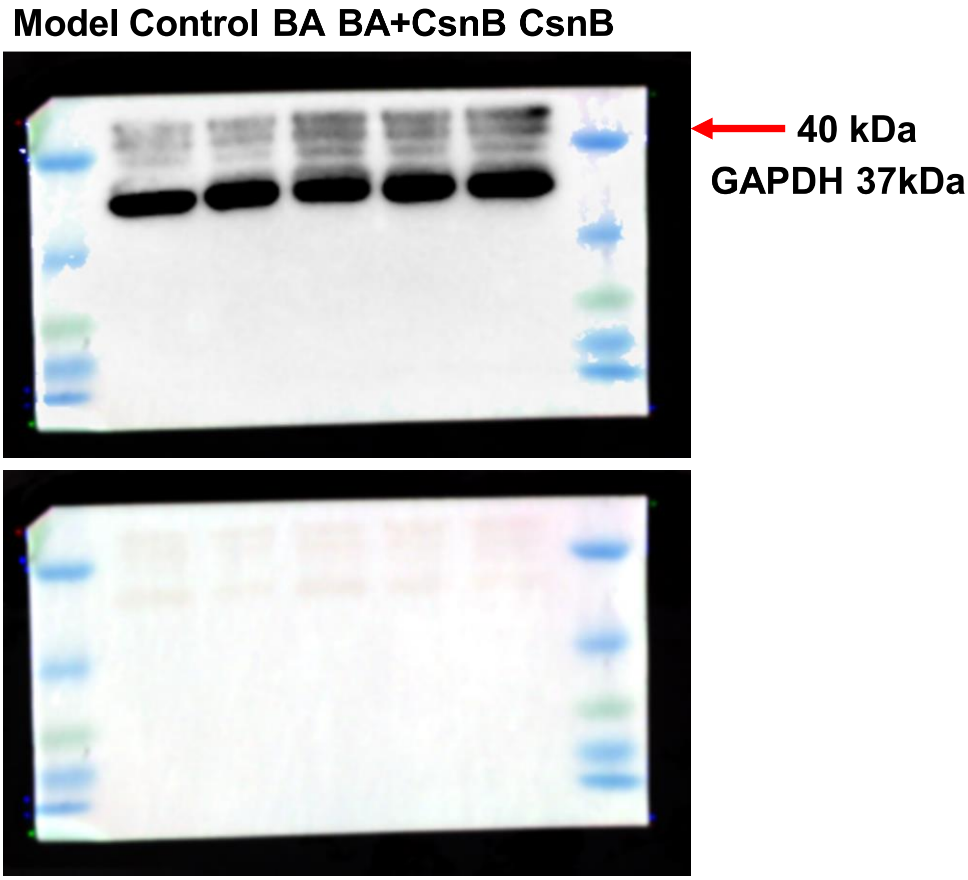

Repeat 1, the merged image

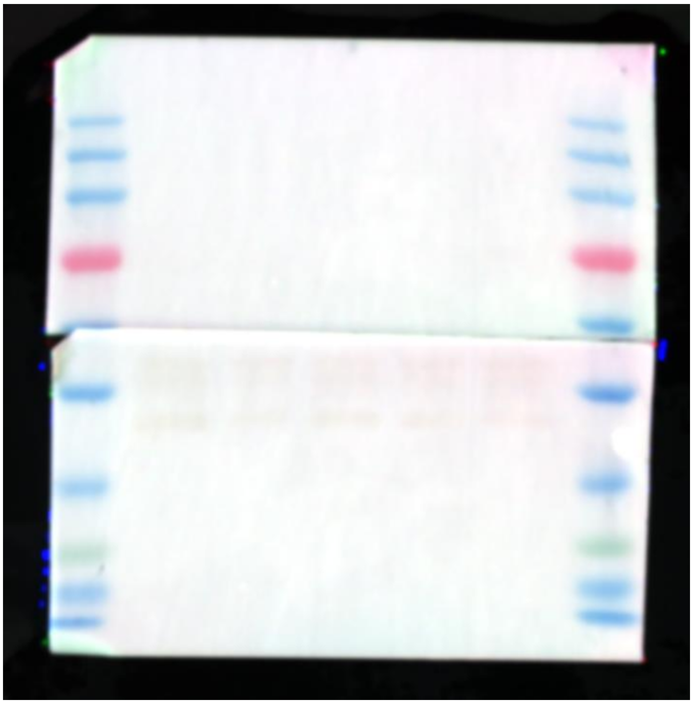

Repeat 2

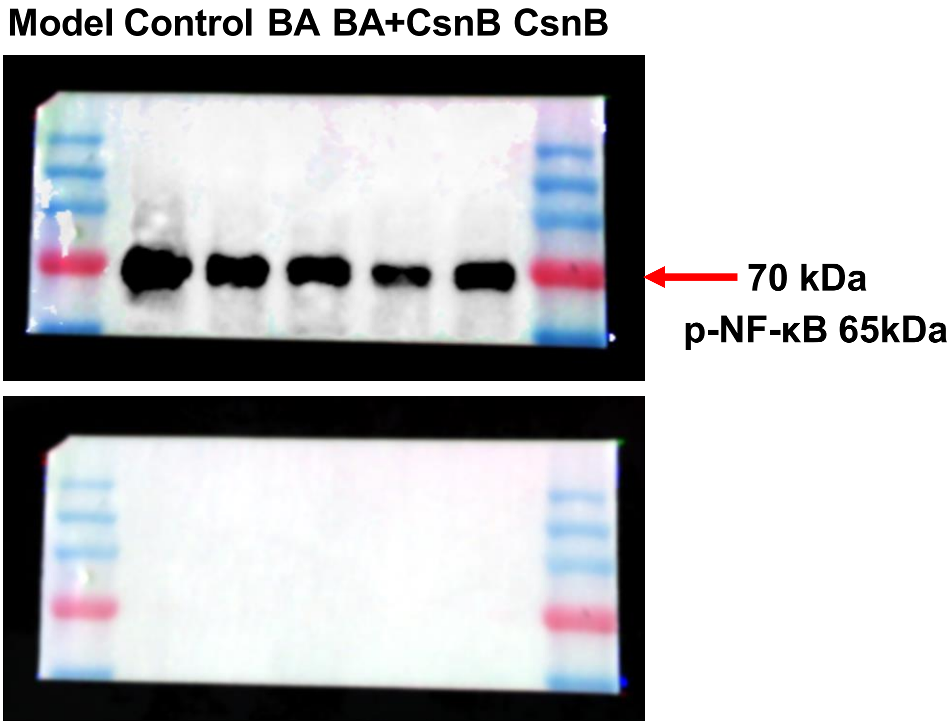

Repeat 2

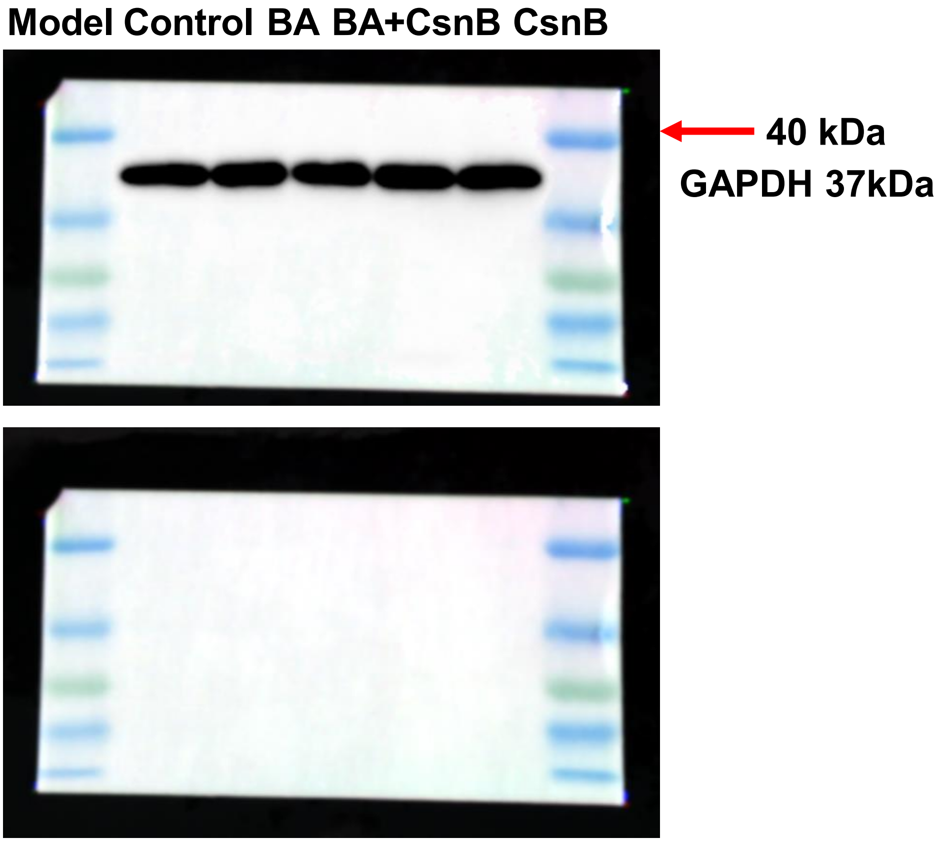

Repeat 2, the merged image

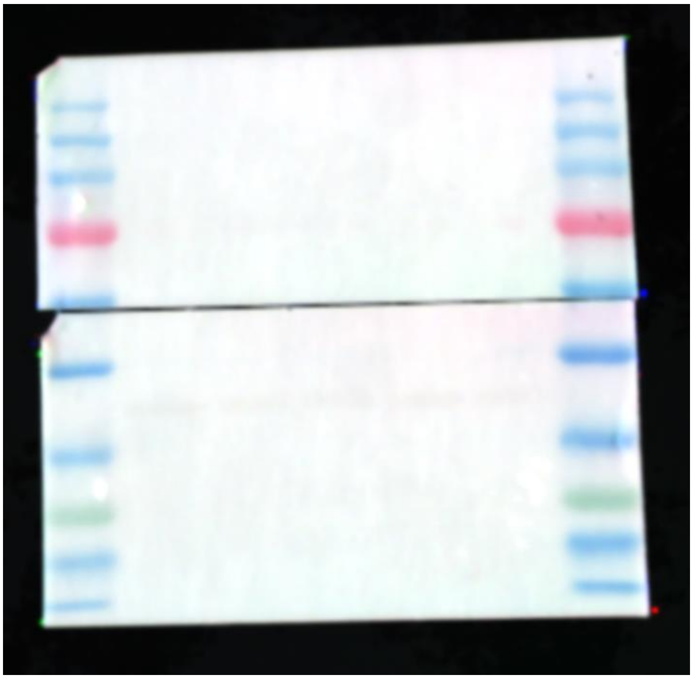

Repeat 3

Model Control BA BA+CsnB CsnB

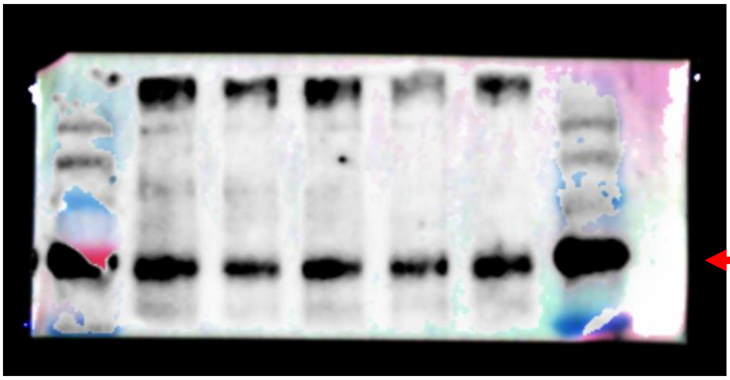

70 kDa

p-NF-κB 65kDa

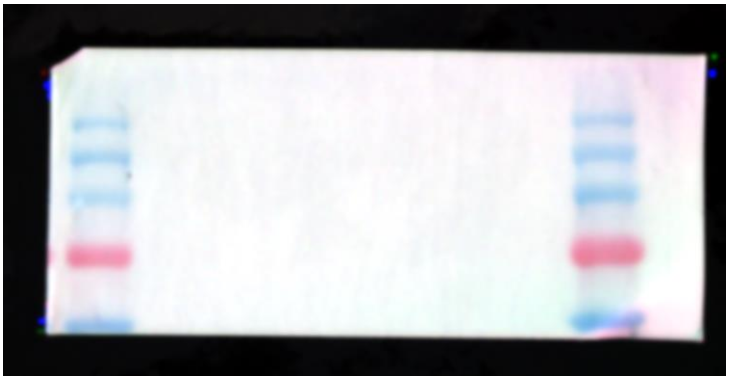

Repeat 3

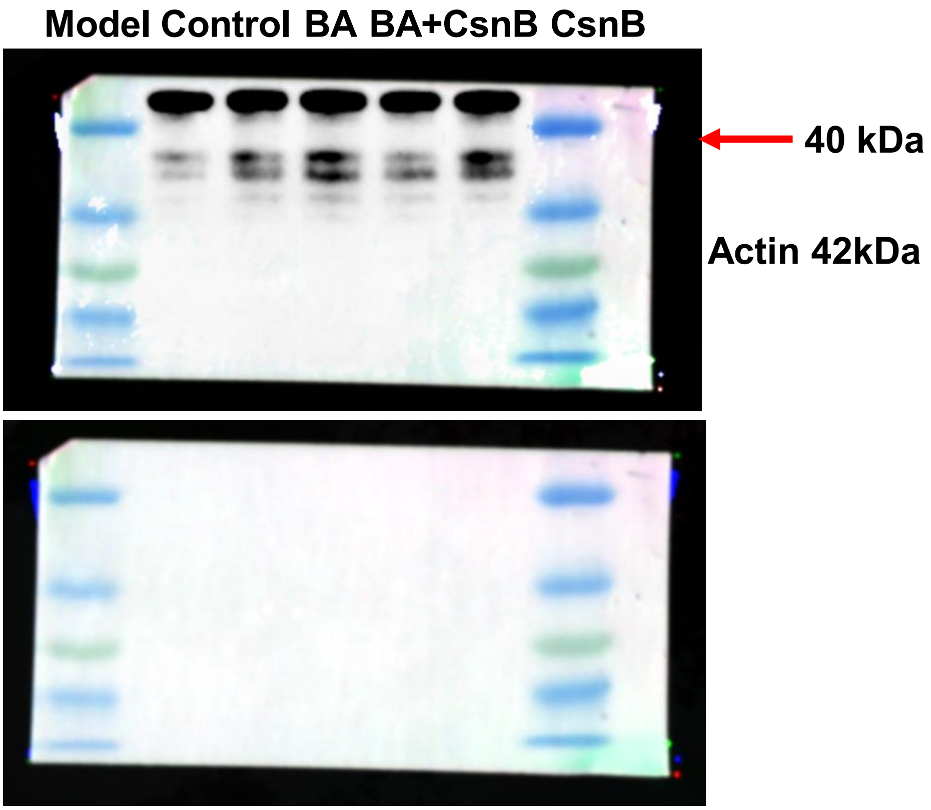

Repeat 3, the merged image

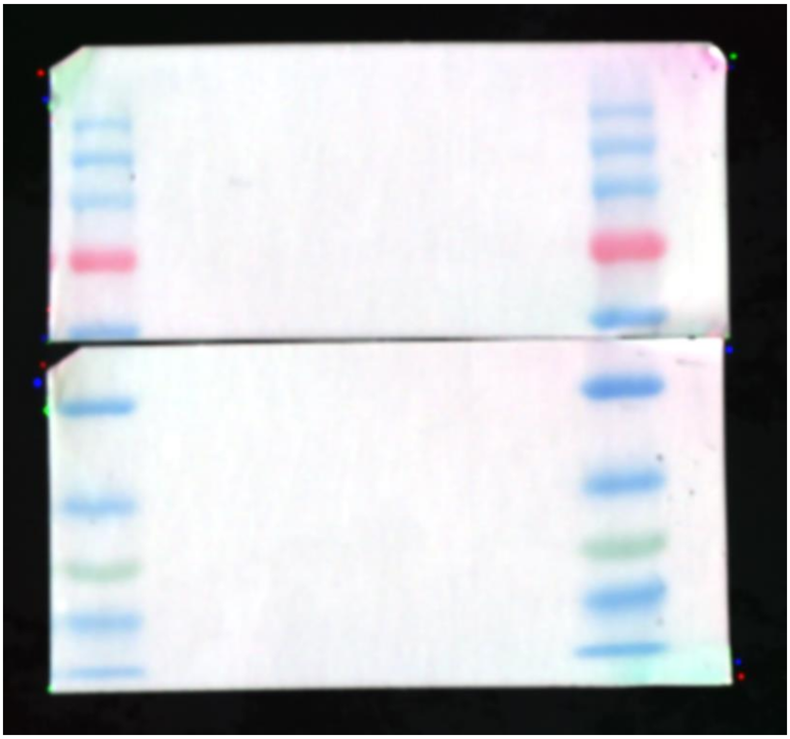

Western blot was performed to evaluate the effects of BA or CsnB on NR4A1 protein expression in mesangial cell *in vivo*

Repeat 1

Model Control BA BA+CsnB CsnB

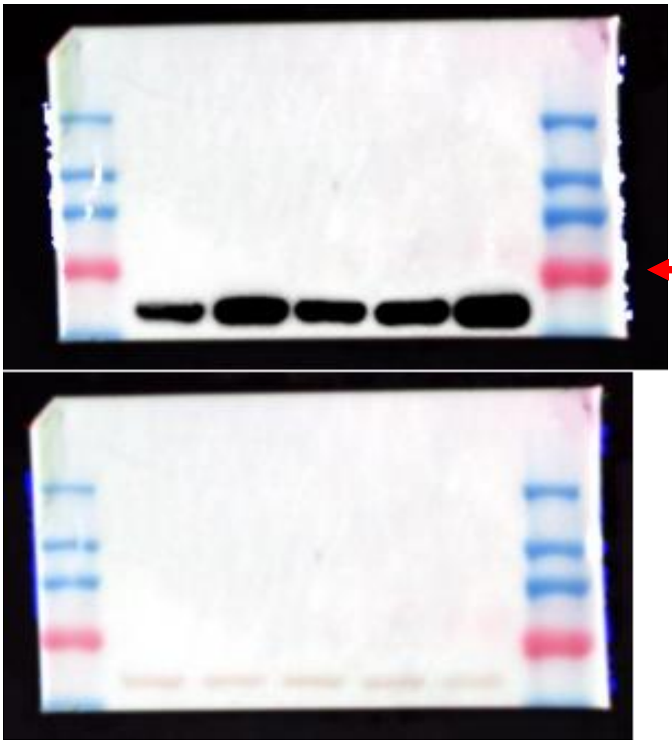

70 kDa

NR4A1 64kDa

Repeat 1

Model Control BA BA+CsnB CsnB

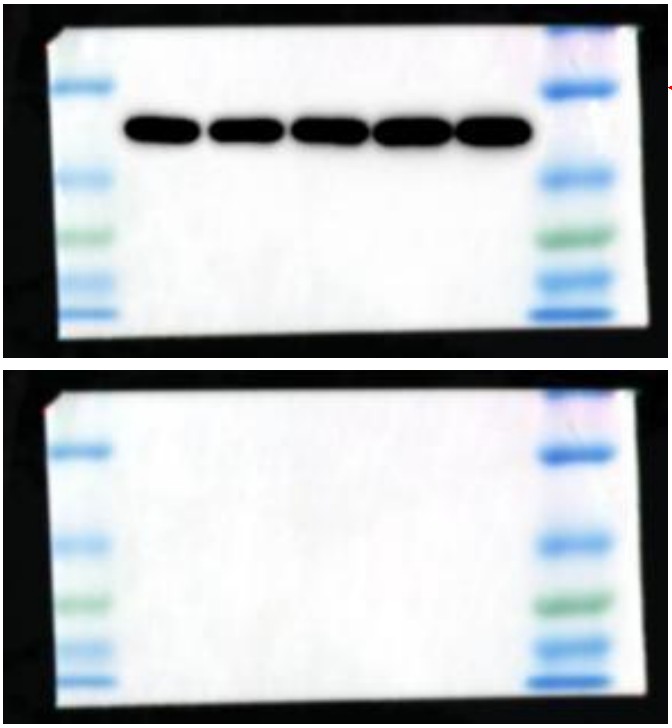

40 kDa

GAPDH 37kDa

Repeat 1, the merged image

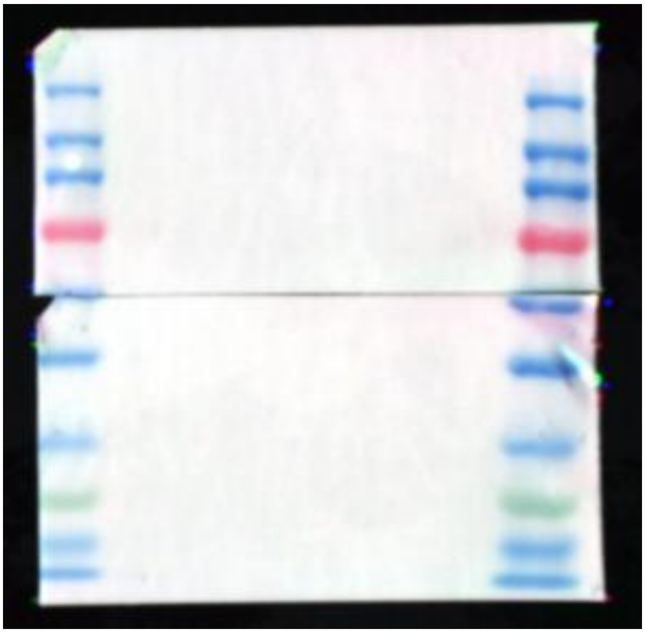

Repeat 2

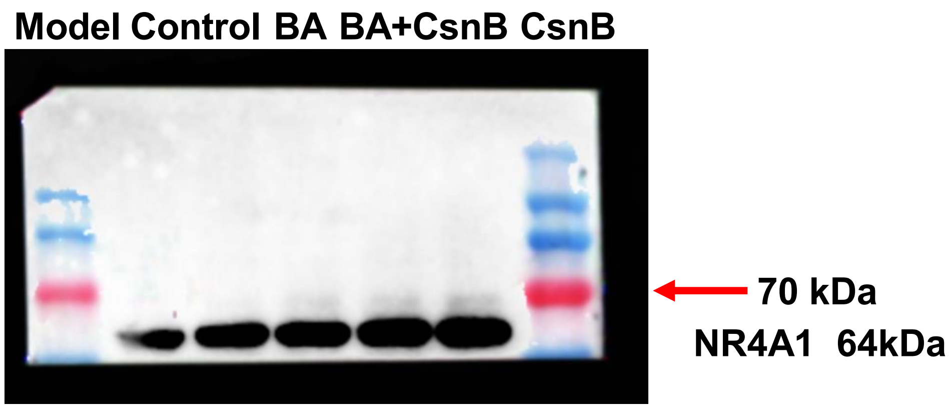

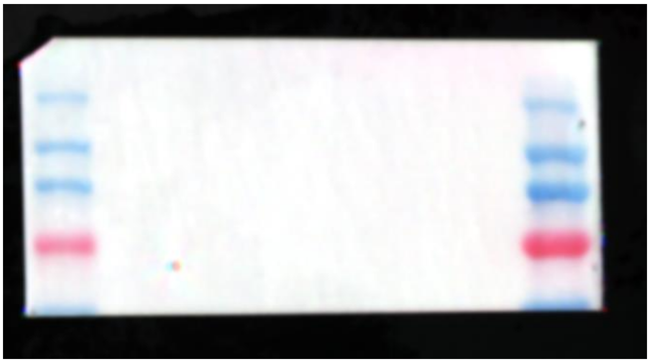

Repeat 2

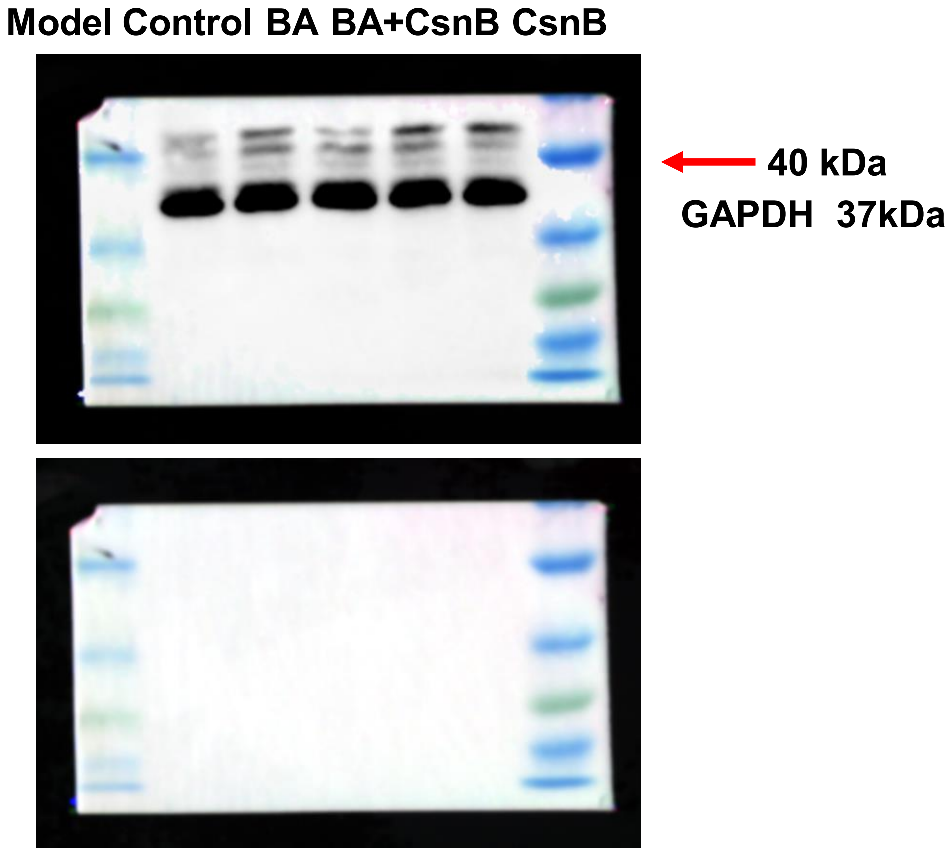

Repeat 2, the merged image

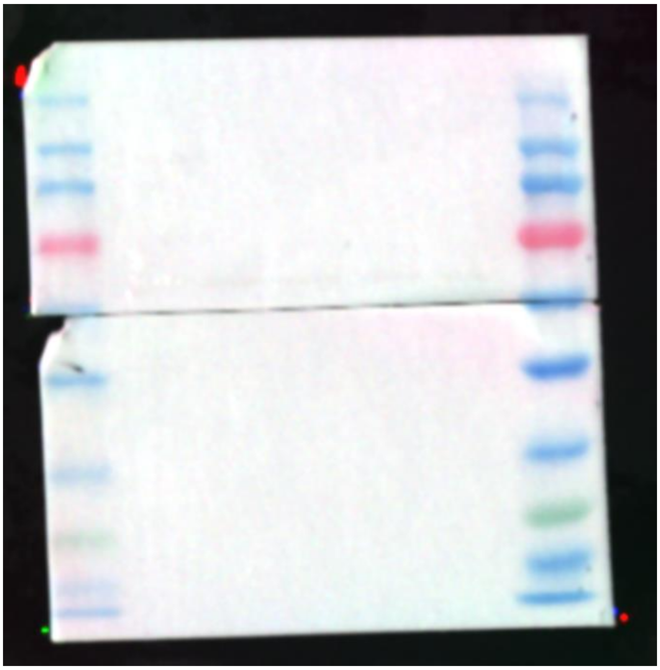

Repeat 3

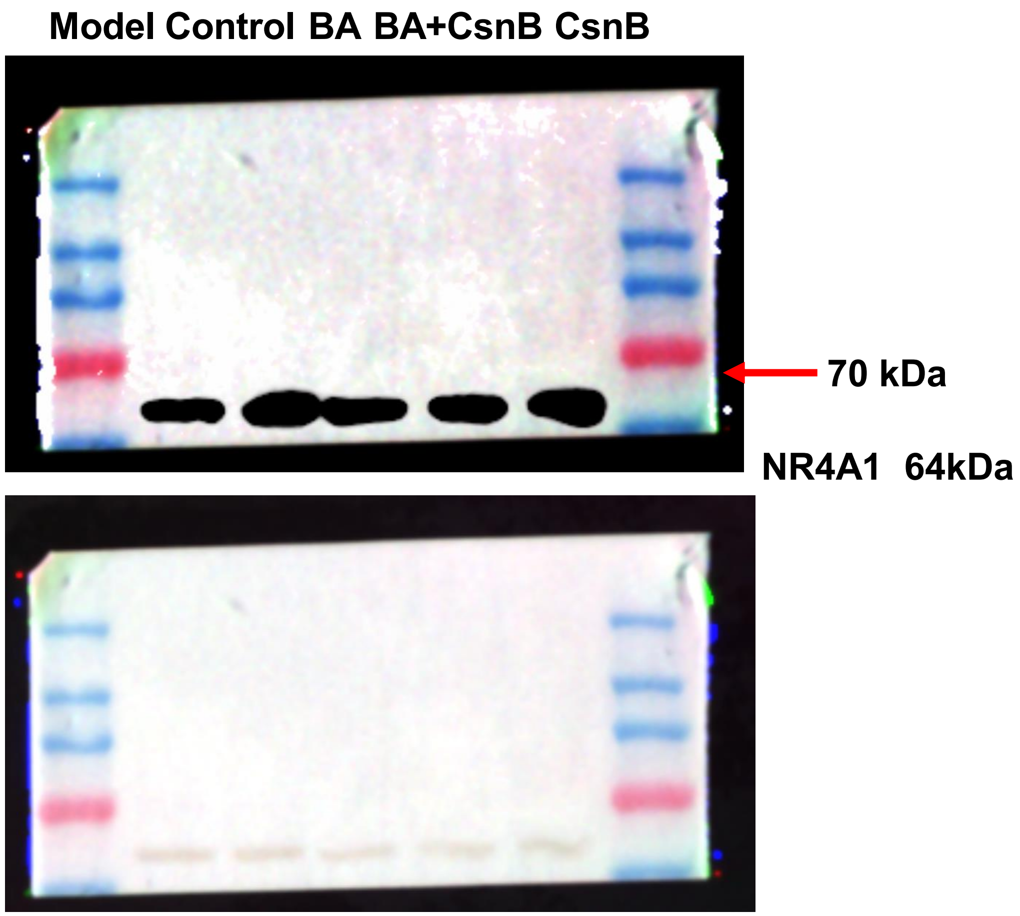

Repeat 3

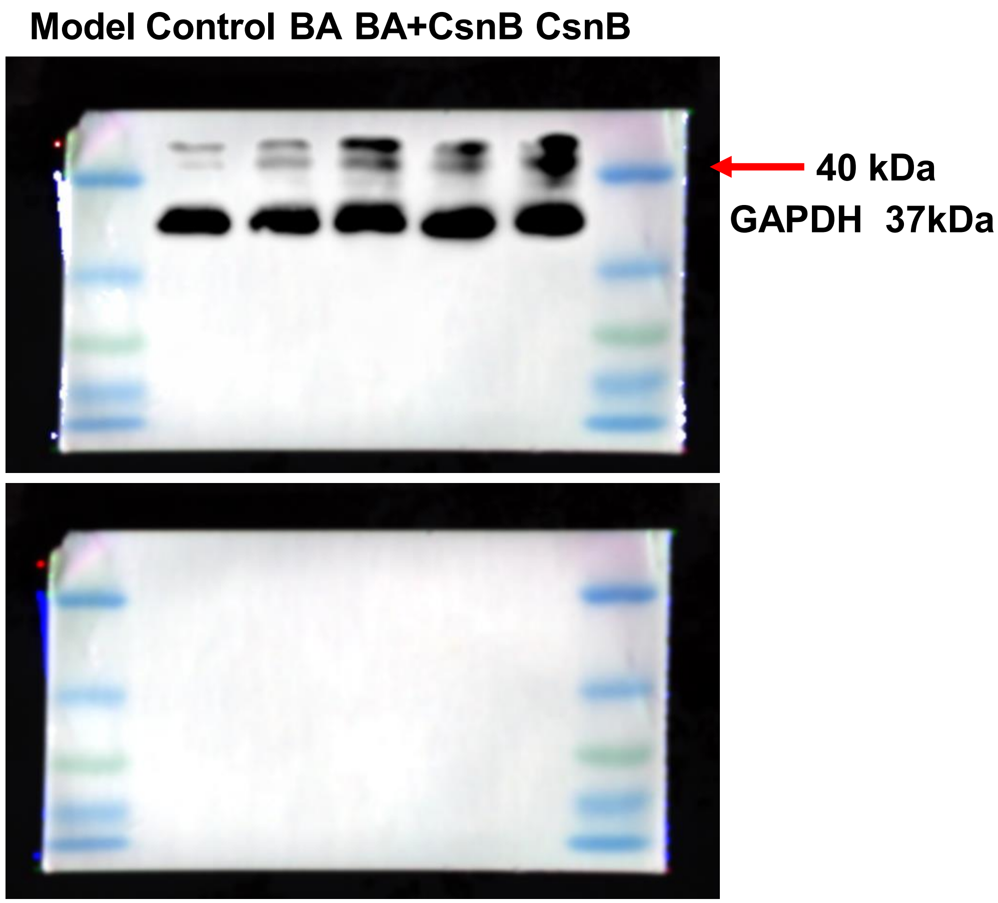

Repeat 3, the merged image

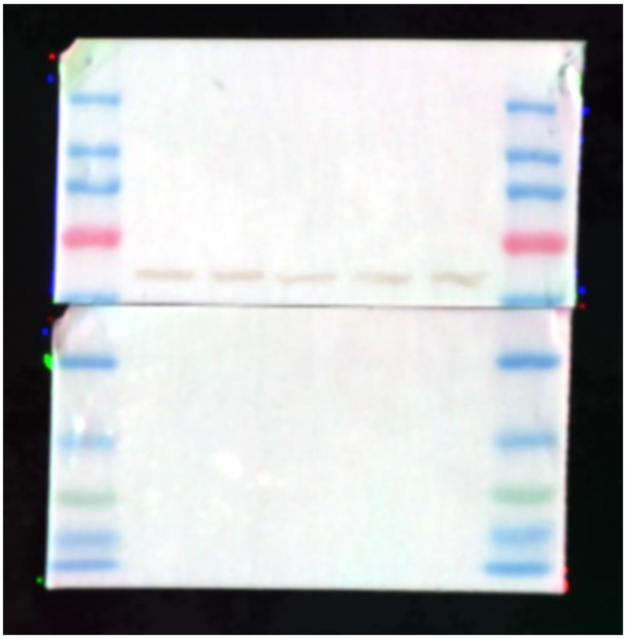

Western blot was performed to evaluate the effects of NR4A1 knockout on NR4A1 protein expression in renal tissues from anti-Thy1 nephritis rats in vivo.

Repeat 1

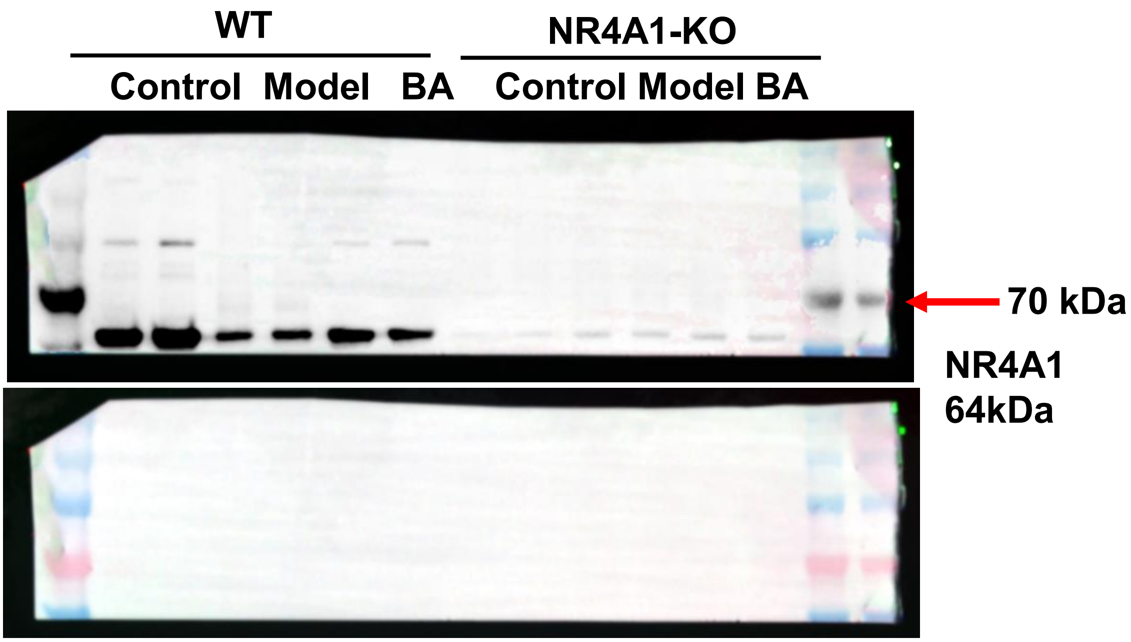

Repeat 1

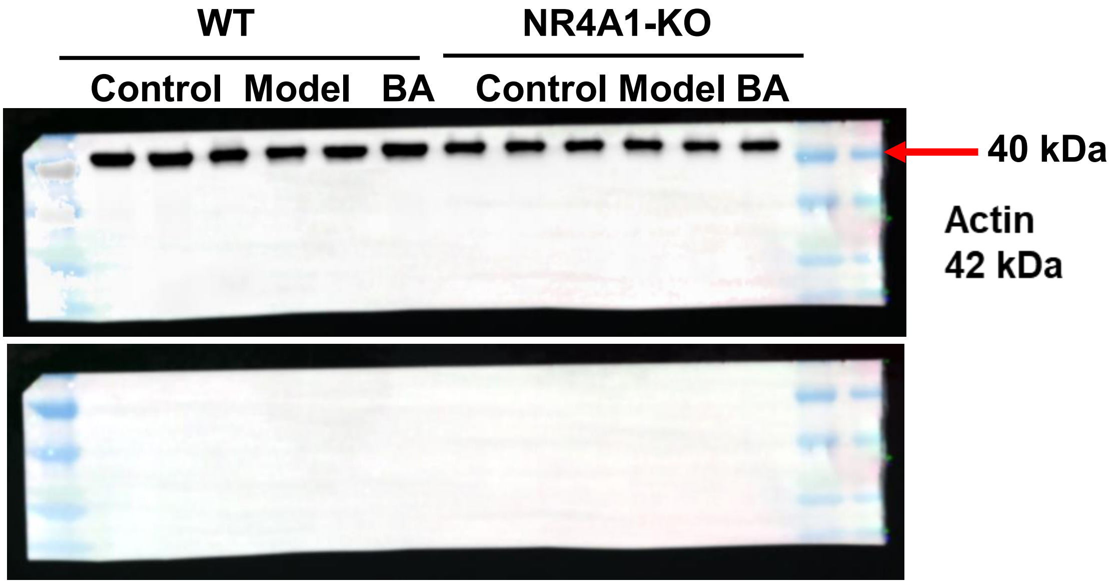

Repeat 1, the merged image

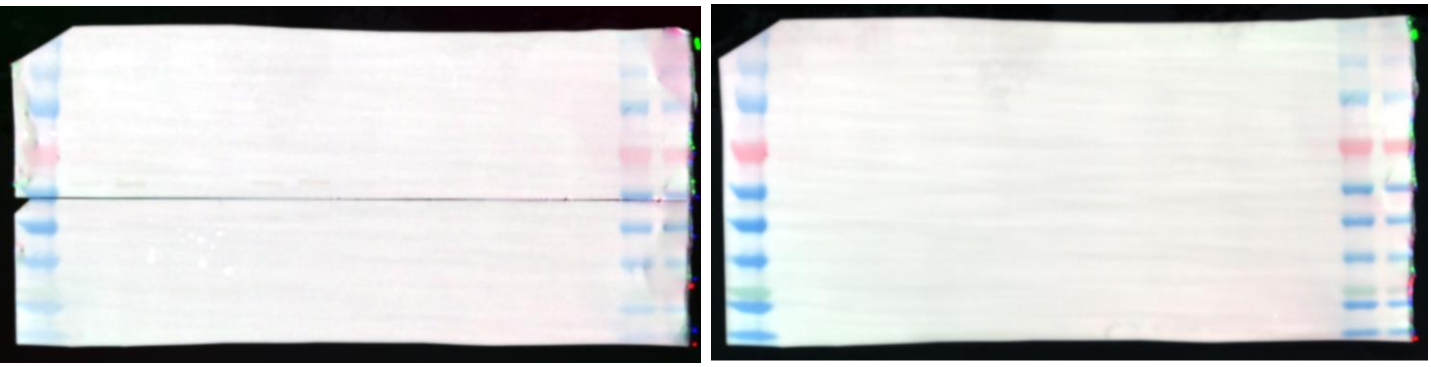

Repeat 2

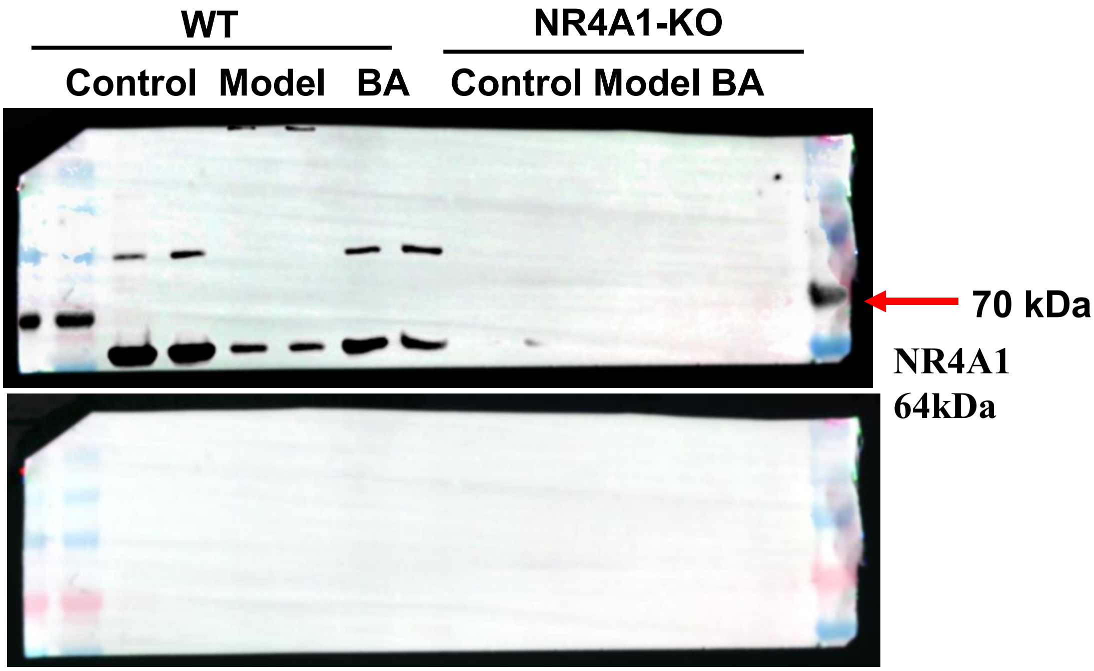

Repeat 2

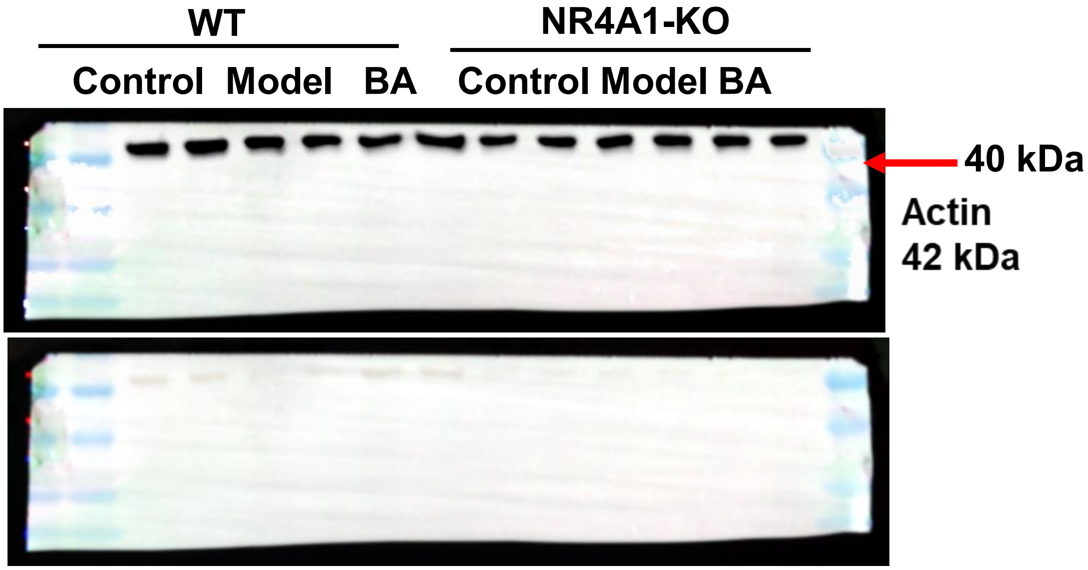

Repeat 2, the merged image

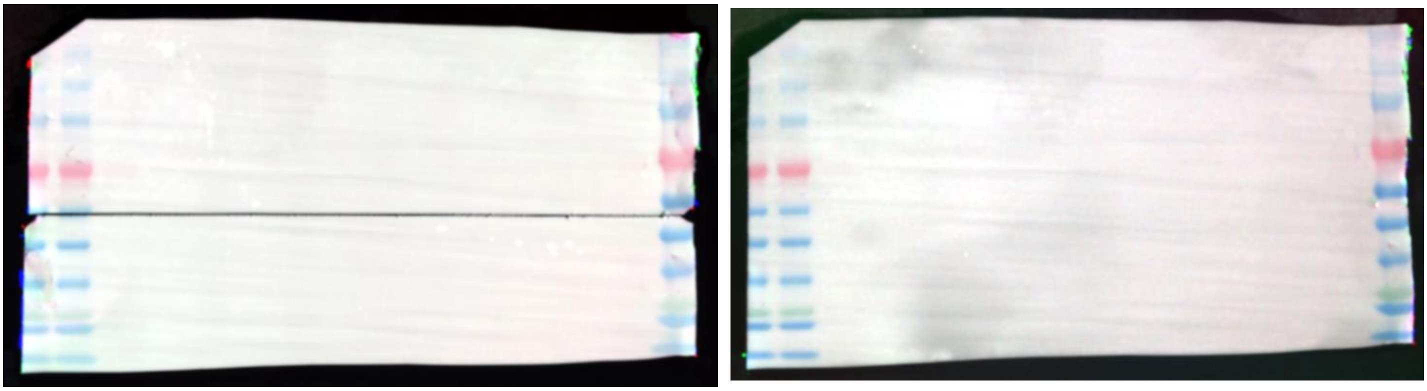

Repeat 3

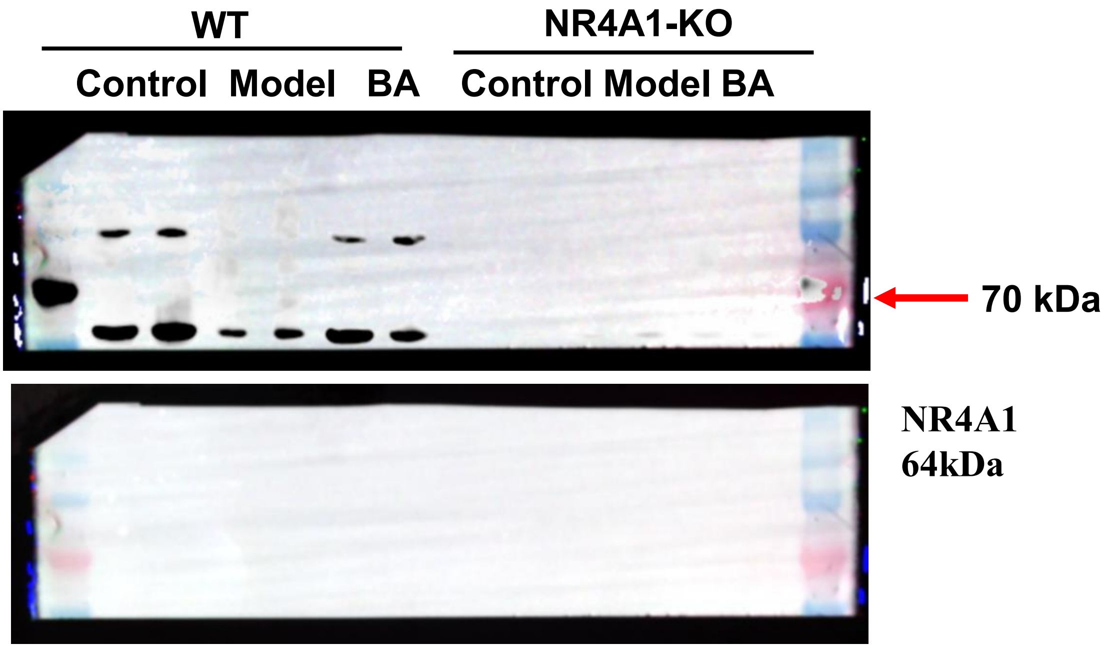

Repeat 3

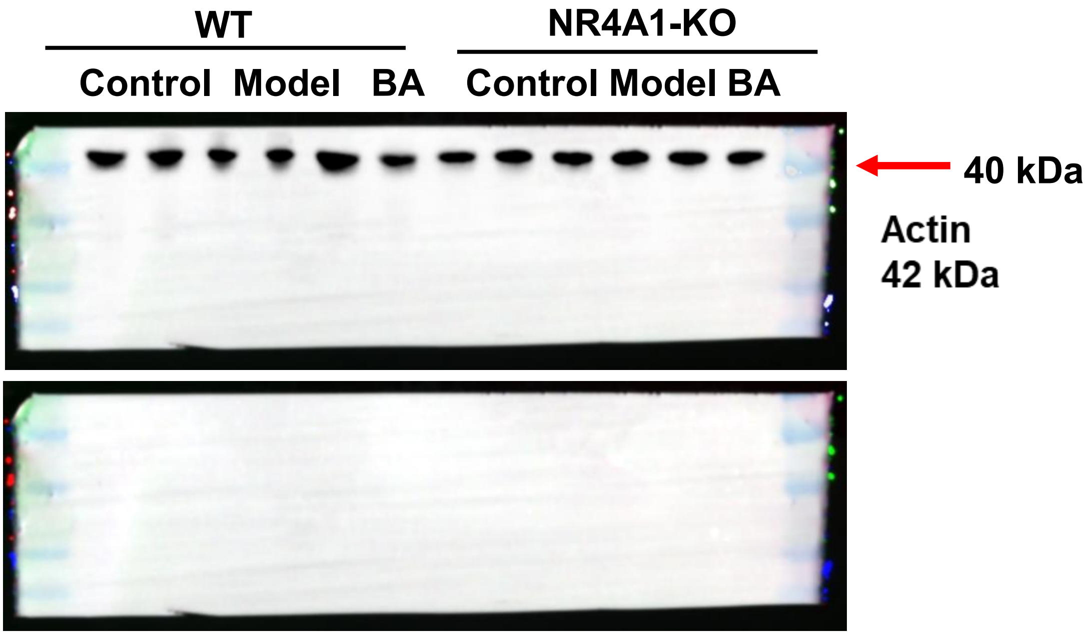

Repeat 3, the merged image

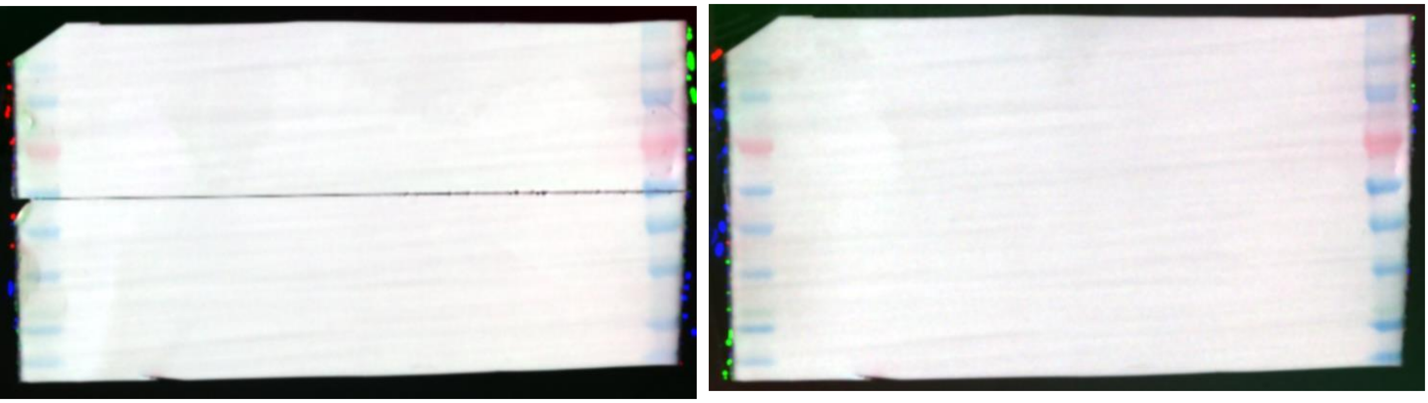

Western blot was performed to evaluate the effect of NR4A1 knockout on p-NF-κB protein expression in renal tissues from anti-Thy1 nephritis rats *in vivo.*

Repeat 1

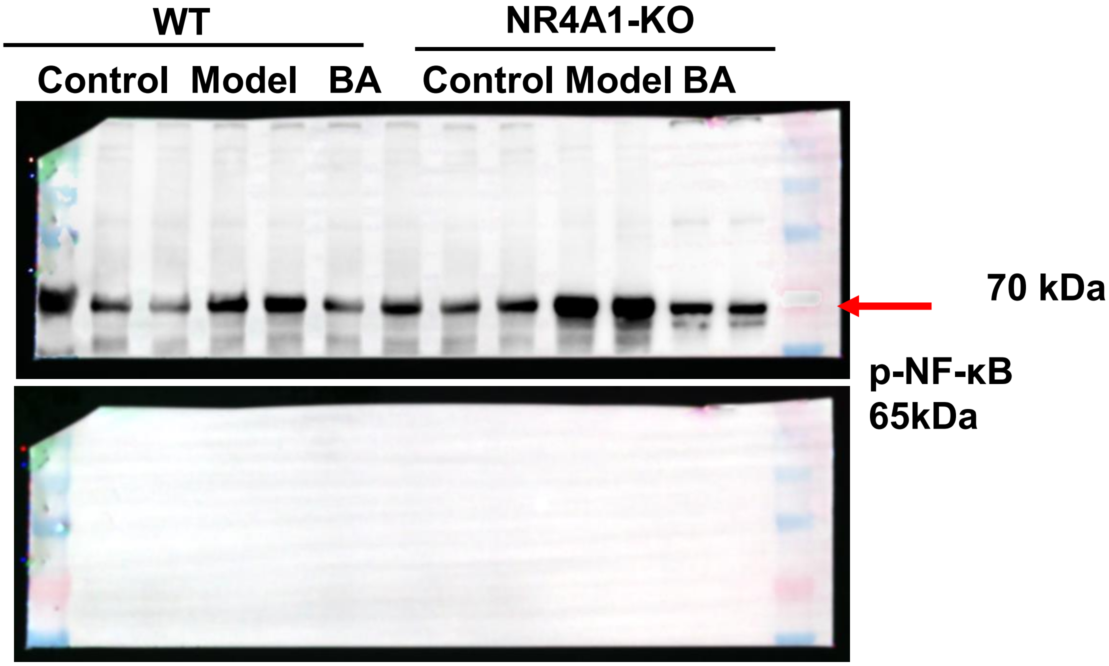

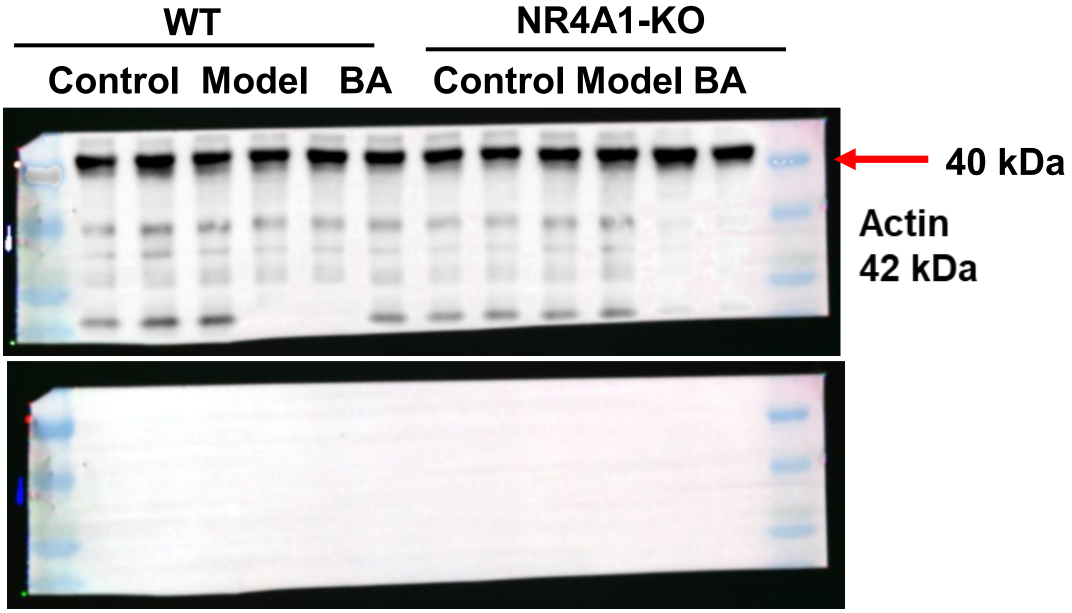

Repeat 1, the merged image

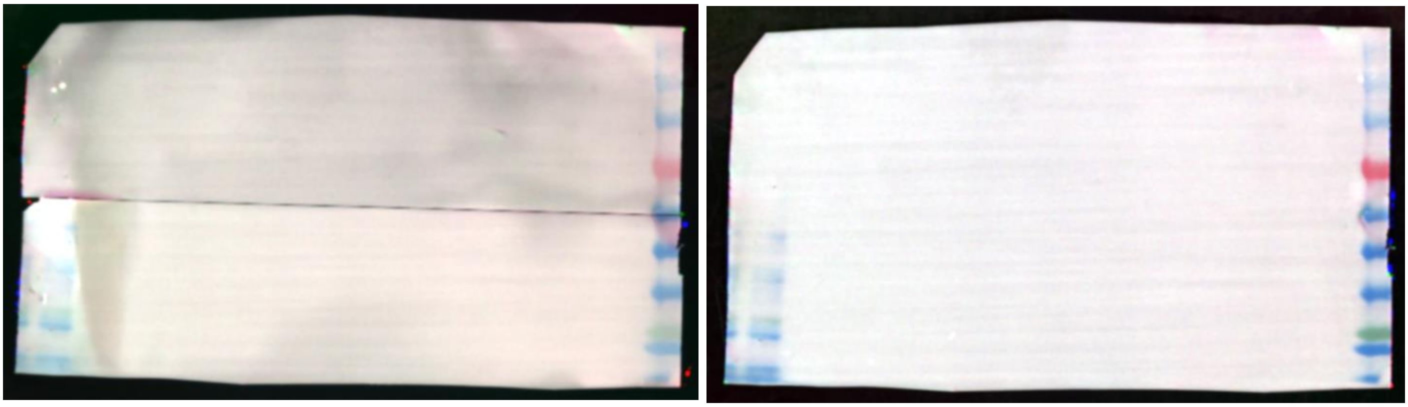

Repeat 2

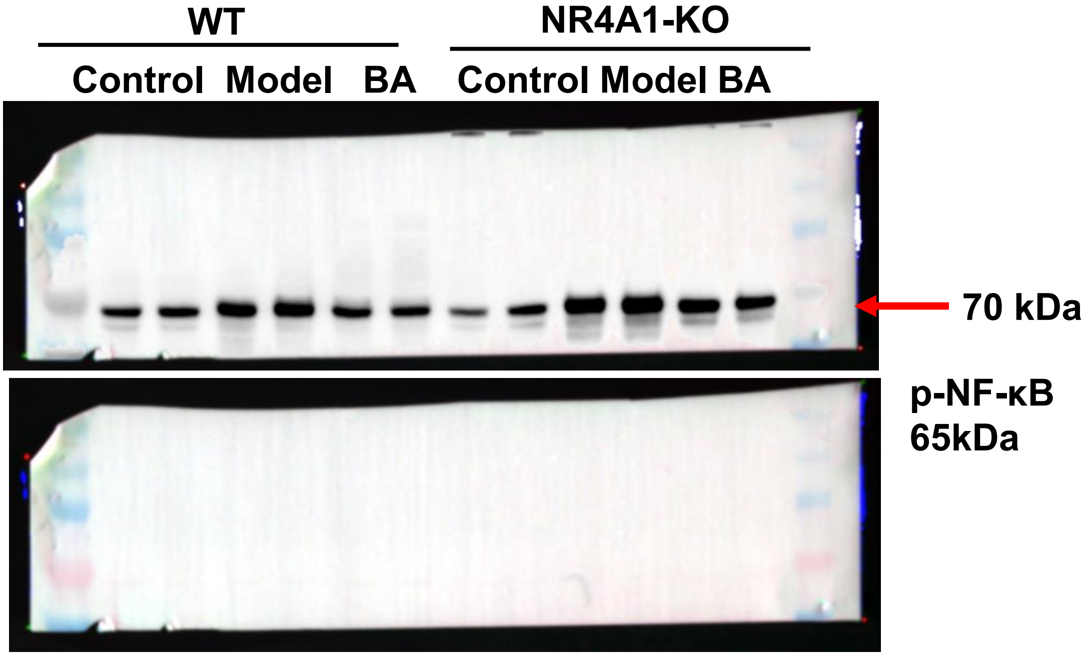

Repeat 2

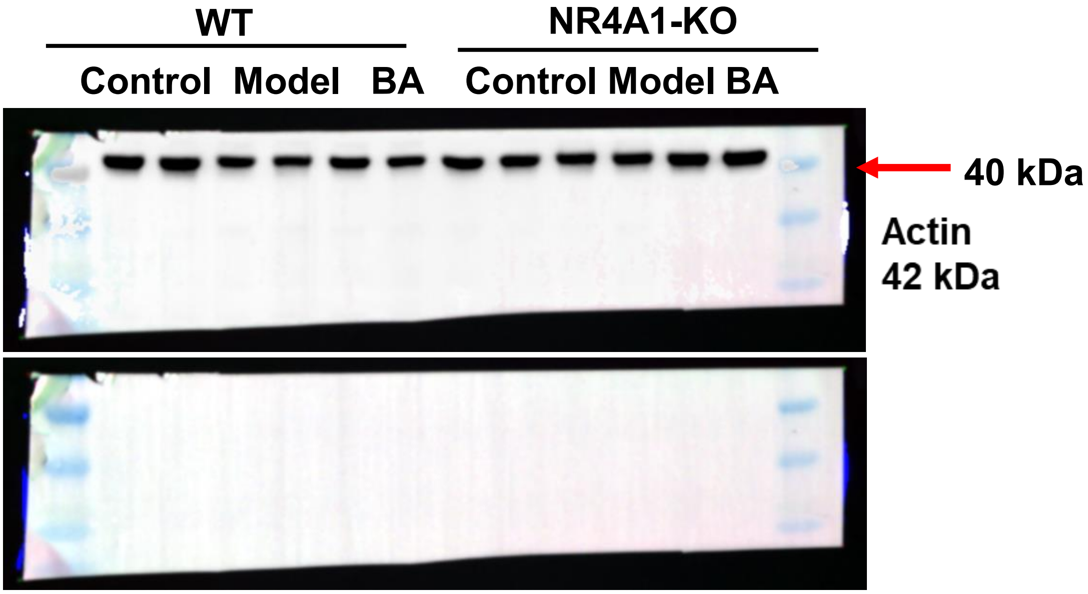

Repeat 2, the merged image

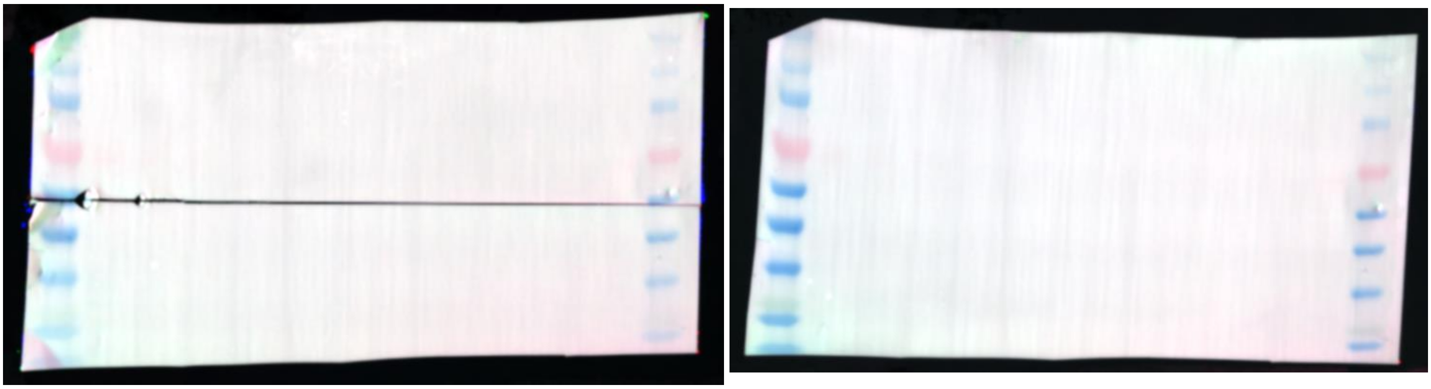

Repeat 3

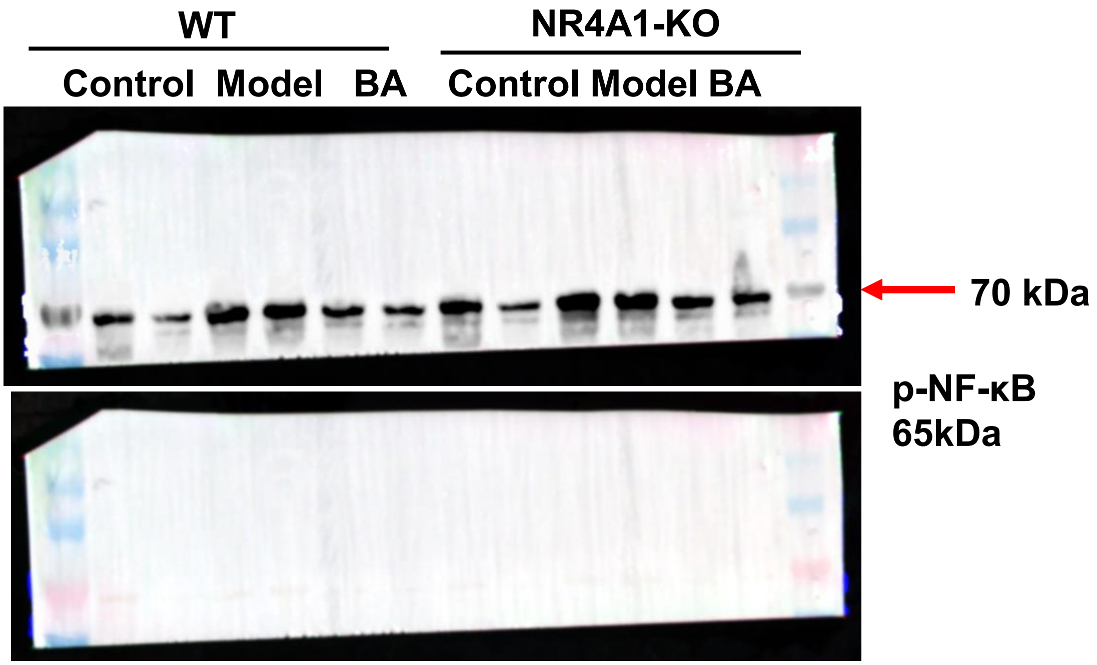

Repeat 3

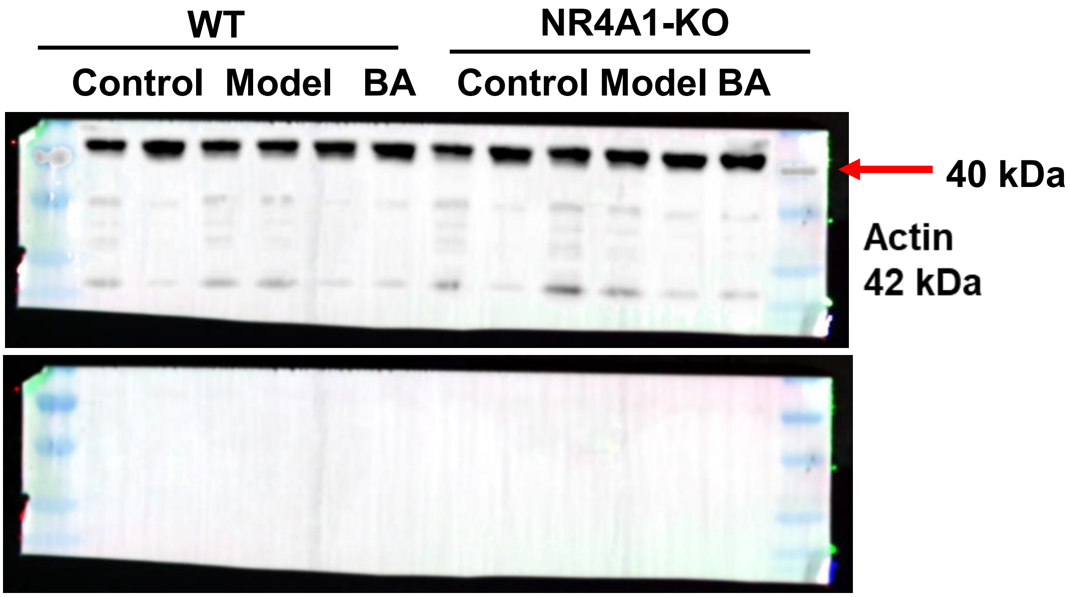

Repeat 3, the merged image

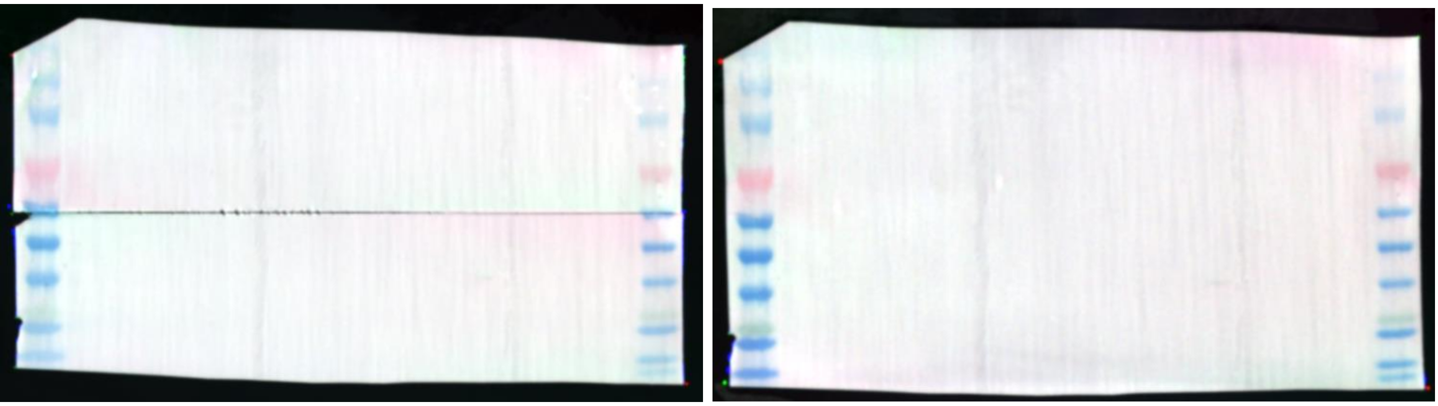

Western blot was performed to evaluate the effects of NR4A1 knockout on NF-κB protein expression in renal tissues from anti-Thy1 nephritis rats *in vivo.*

Repeat 1

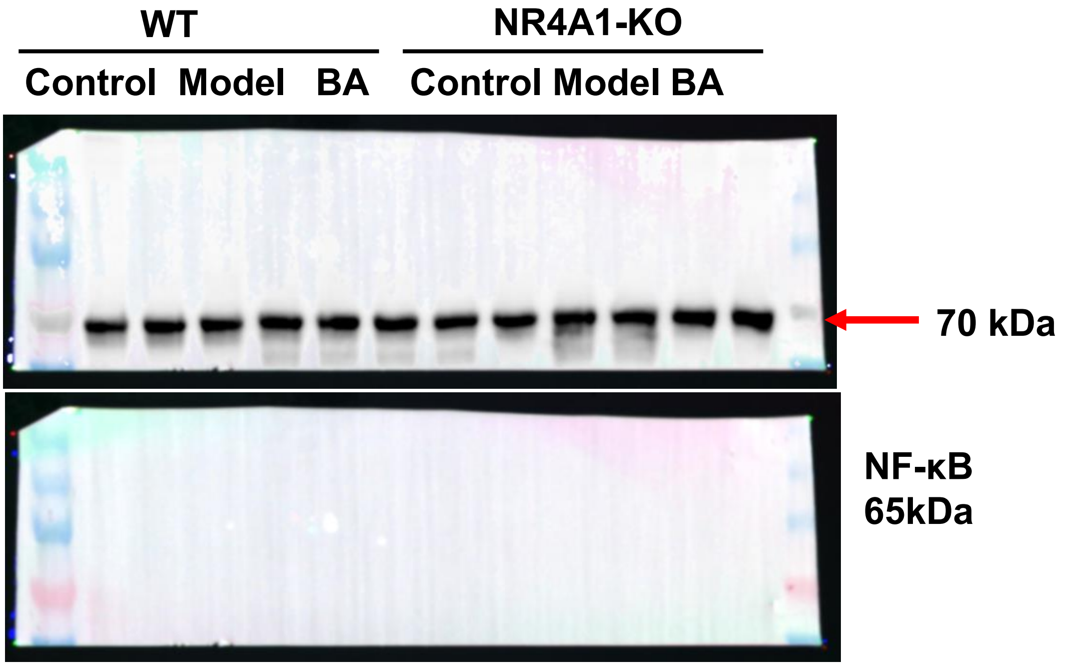

Repeat 1

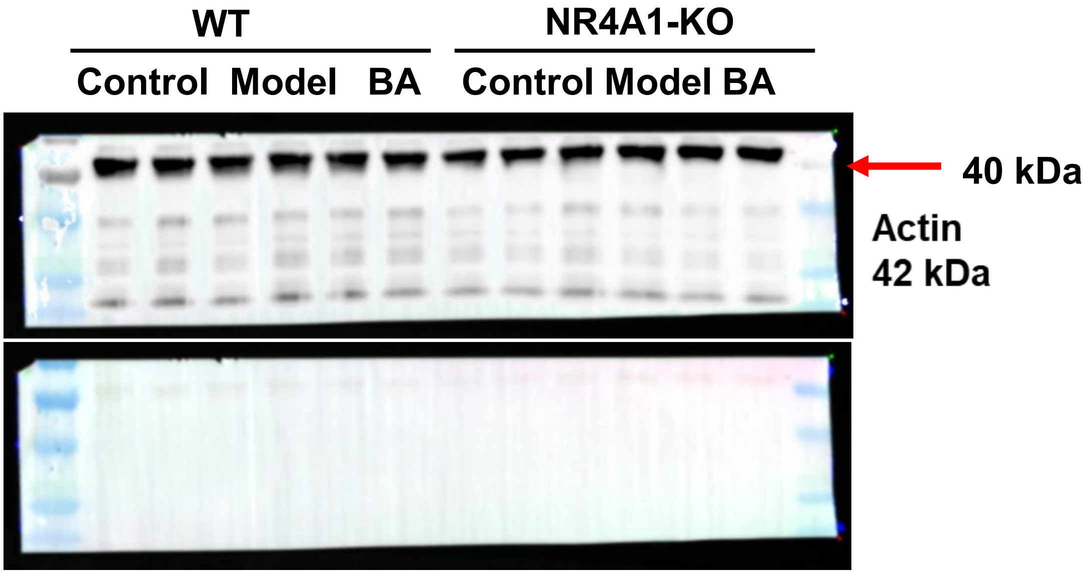

Repeat 1, the merged image

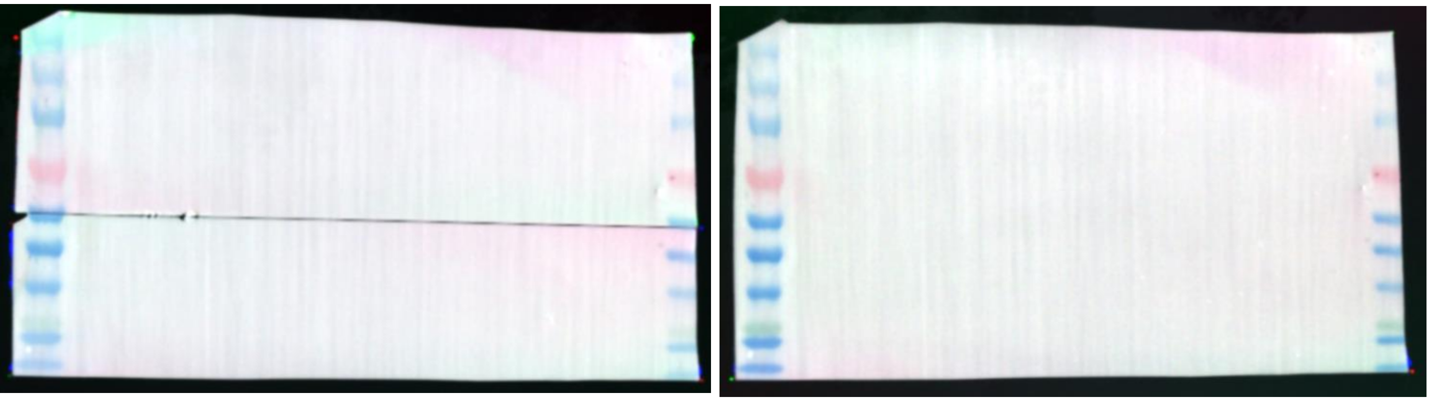

Repeat 2

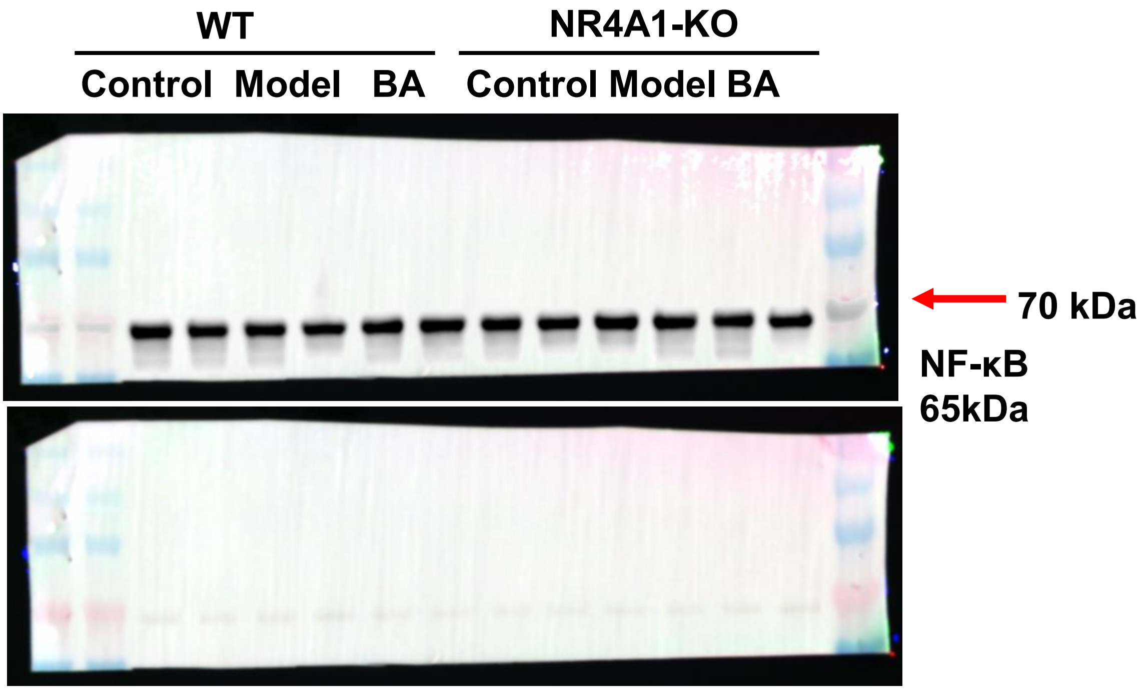

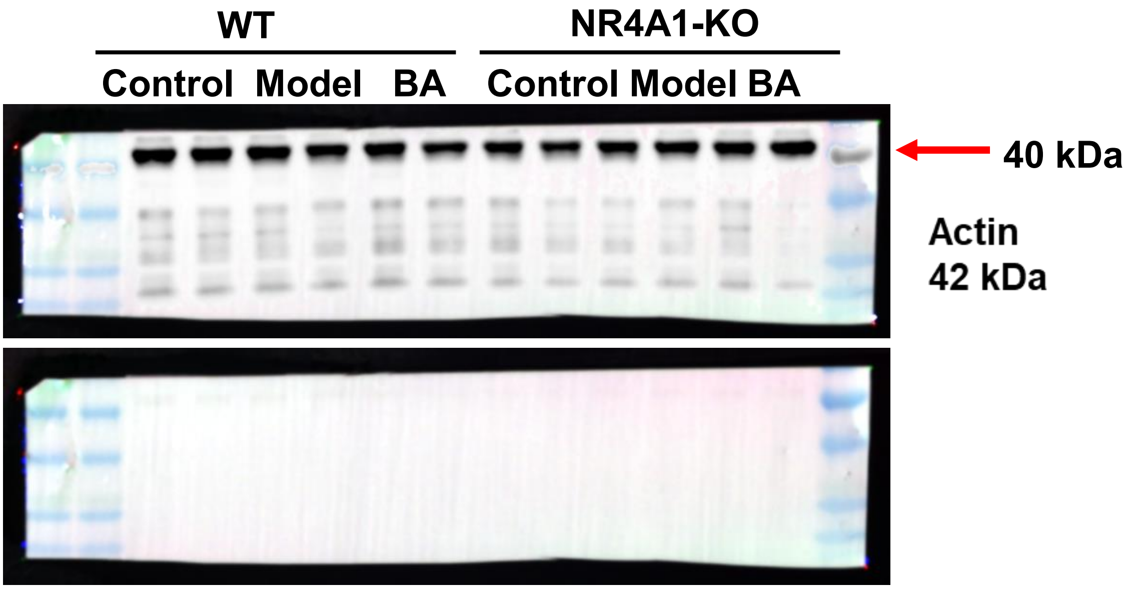

Repeat 2, the merged image

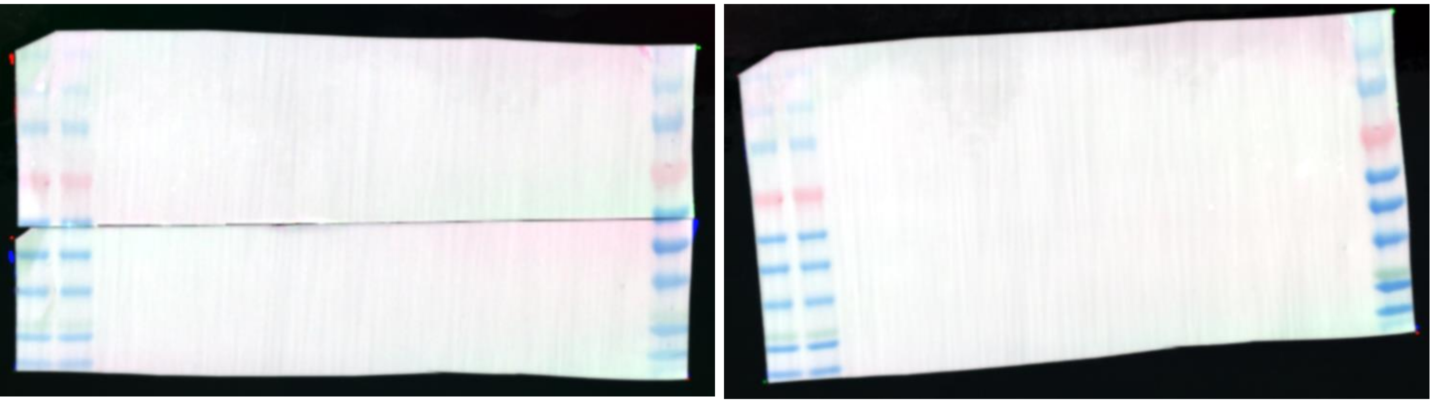

Repeat 3

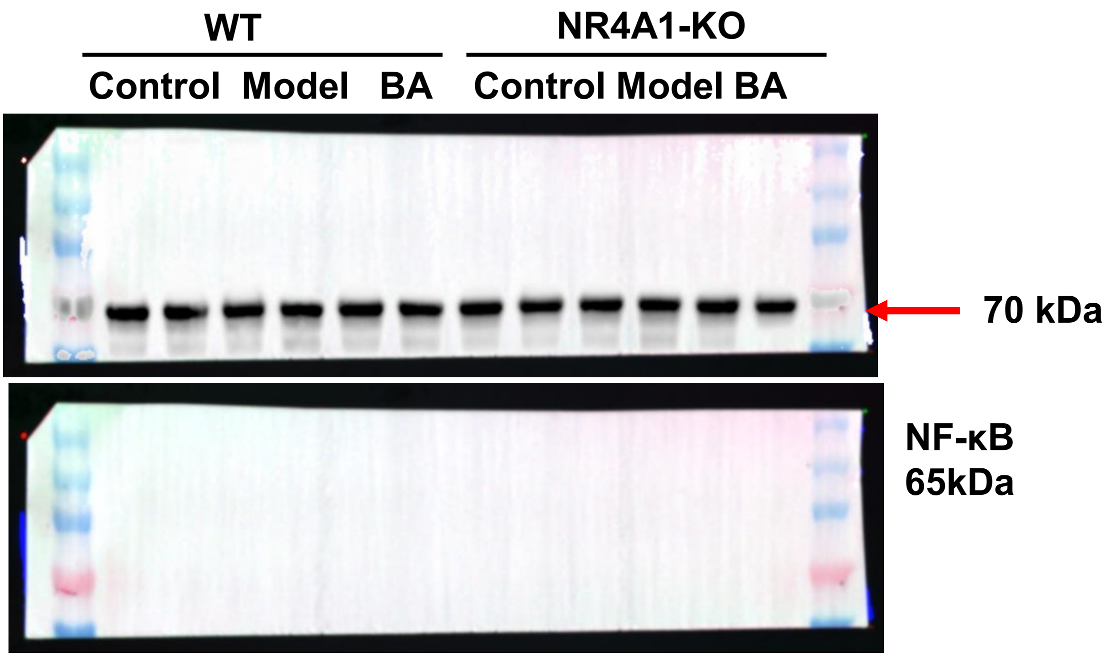

Repeat 3

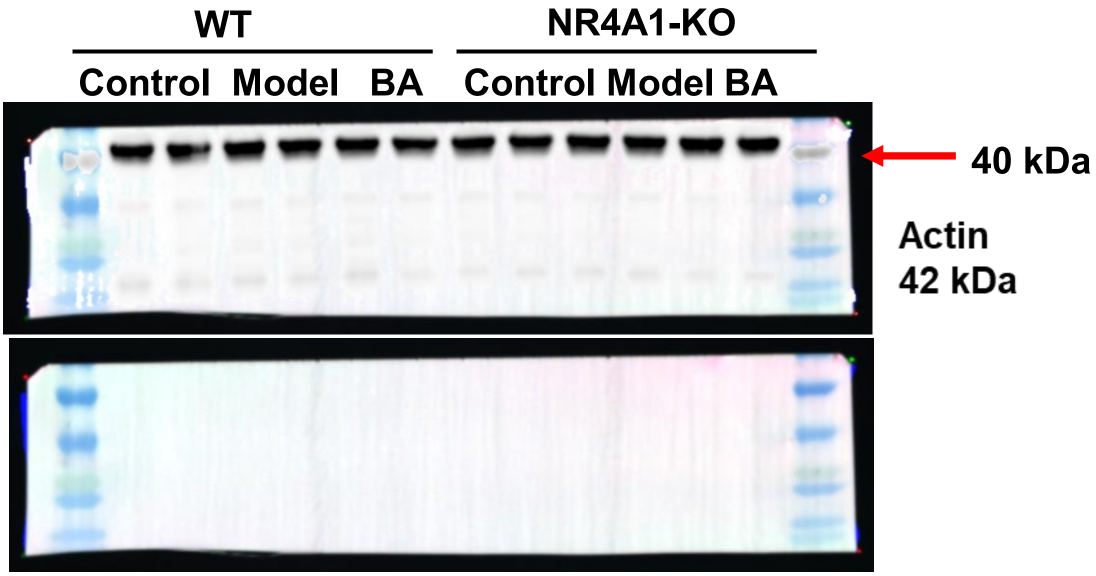

Repeat 3, the merged image

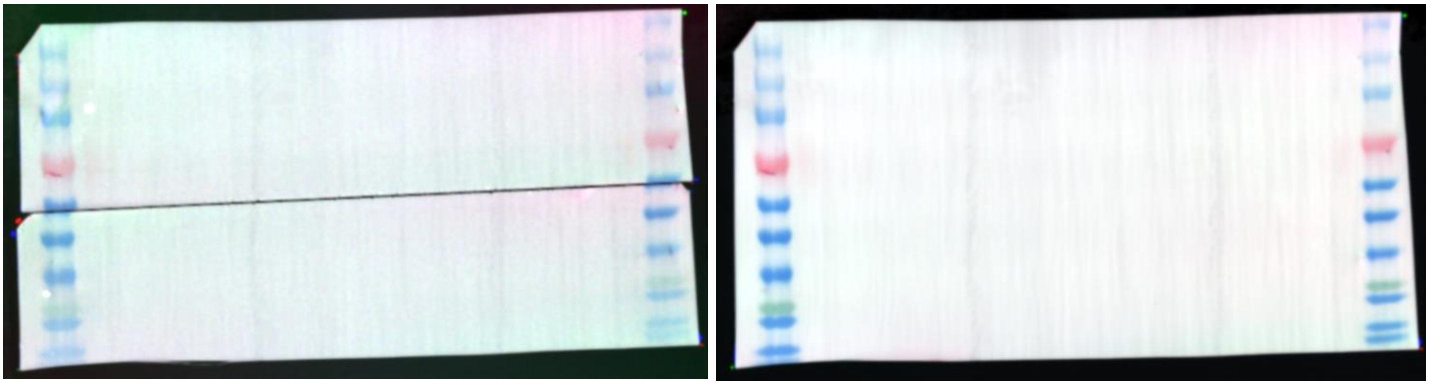

Cycloheximide tracking (CHX Chase) experiment validation

Repeat 1

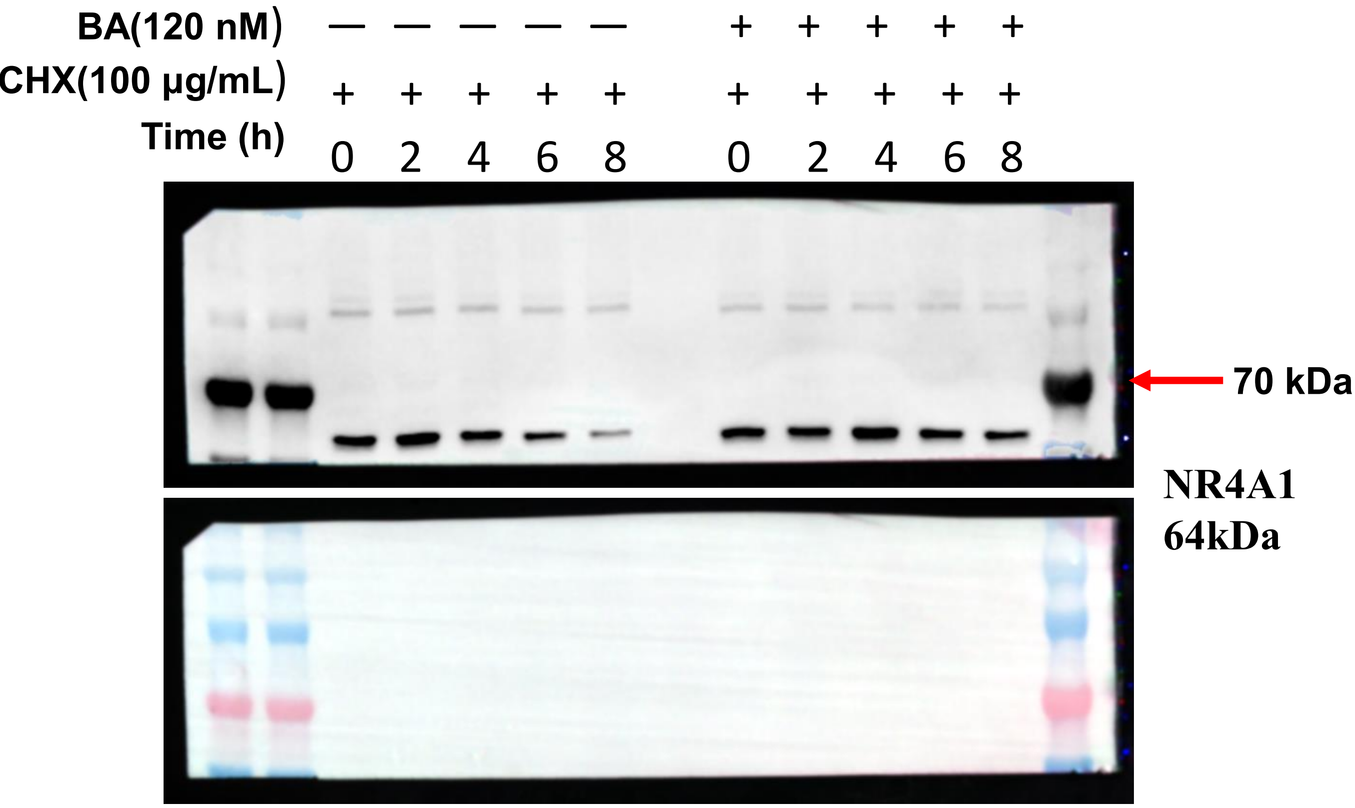

Repeat 1

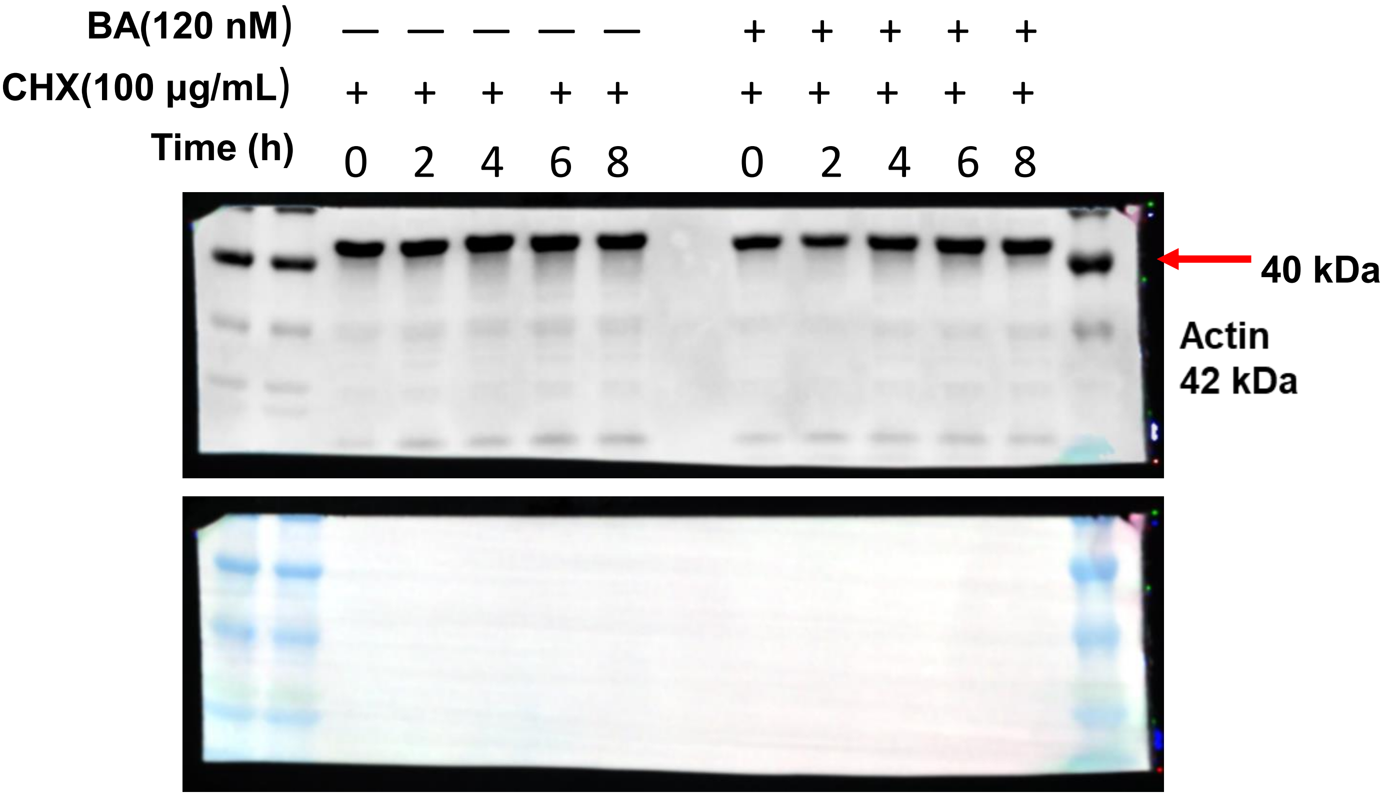

Repeat 1, the merged image

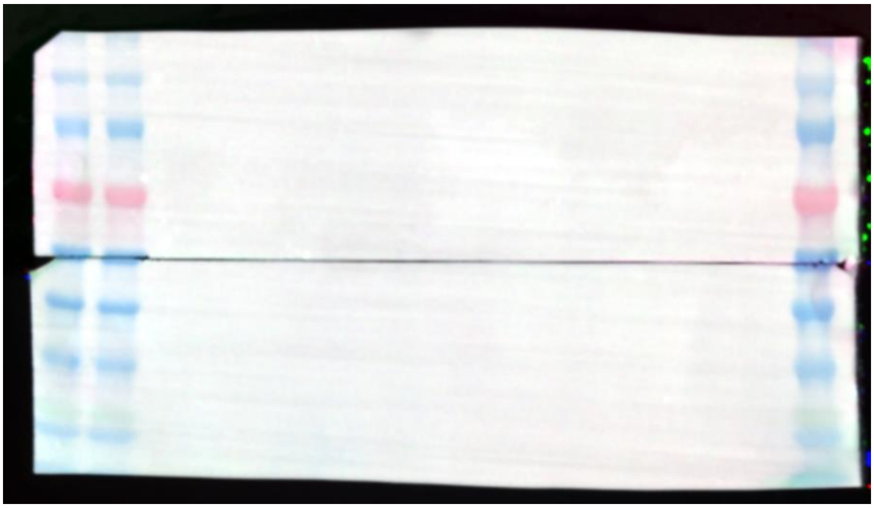

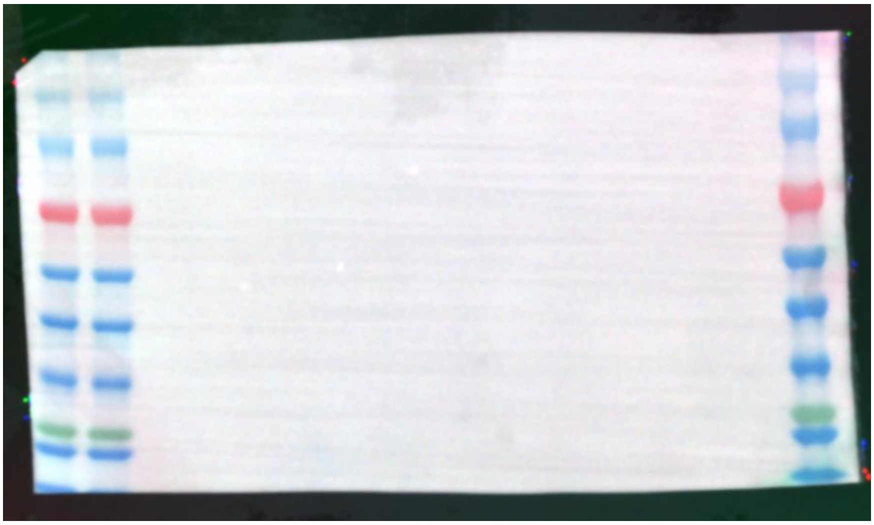

Repeat 2

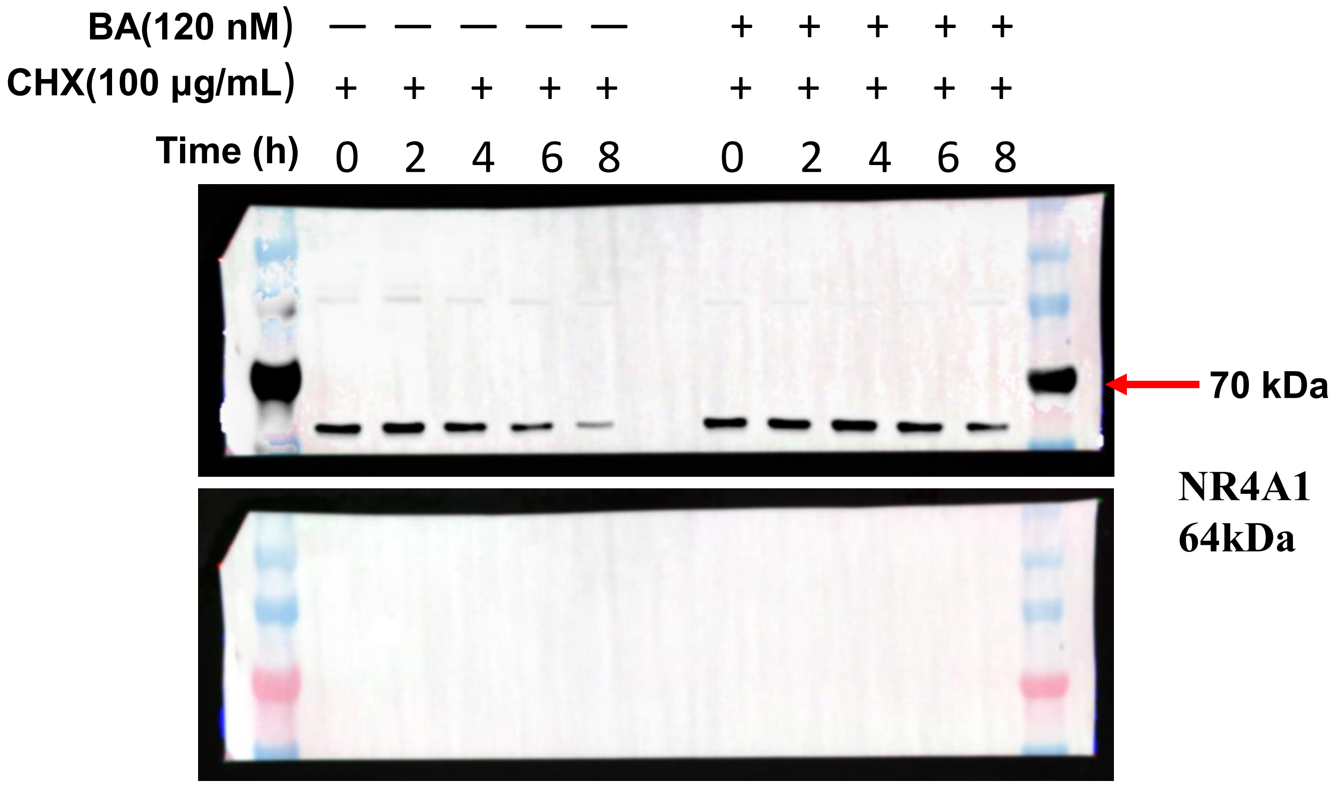

Repeat 2

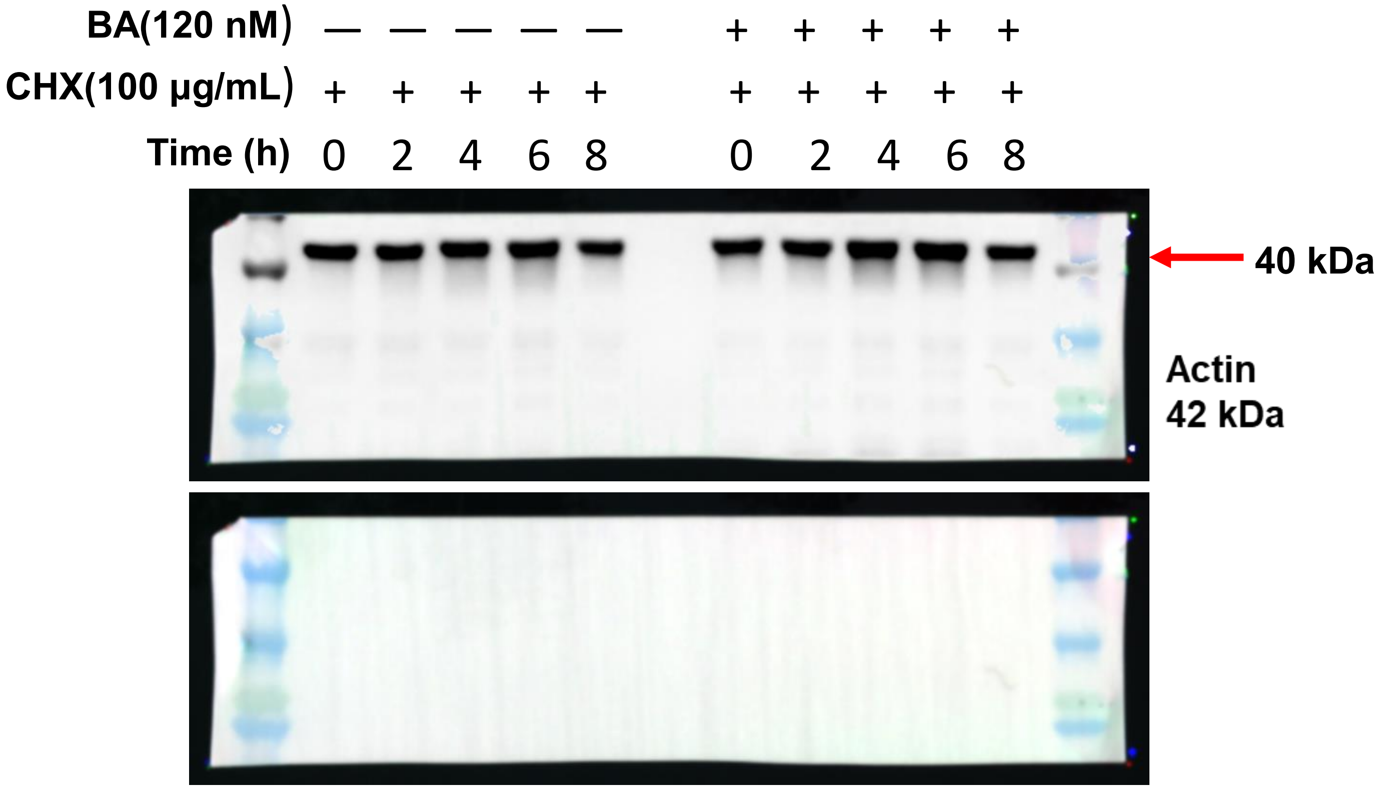

Repeat 2, the merged image

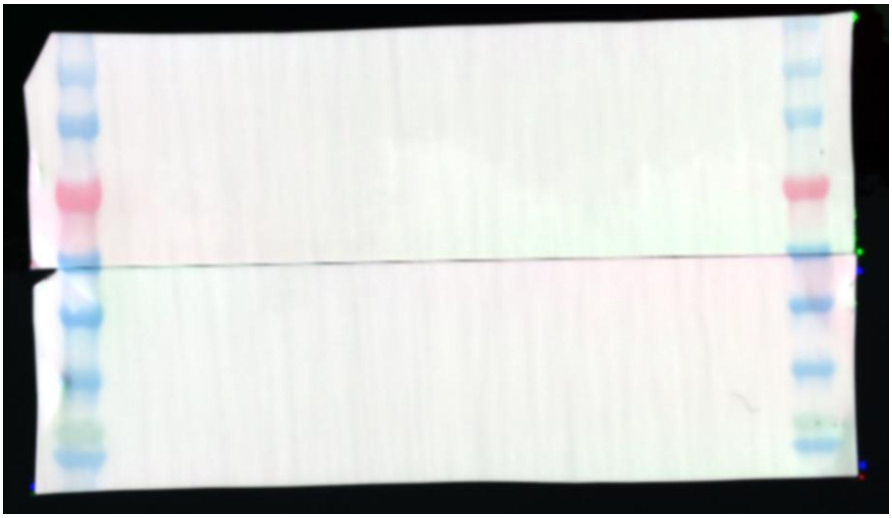

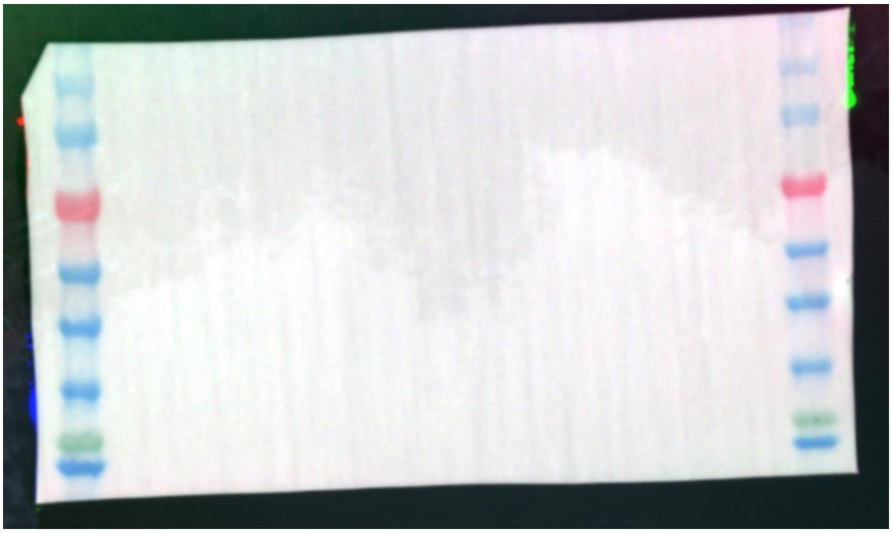

Repeat 3

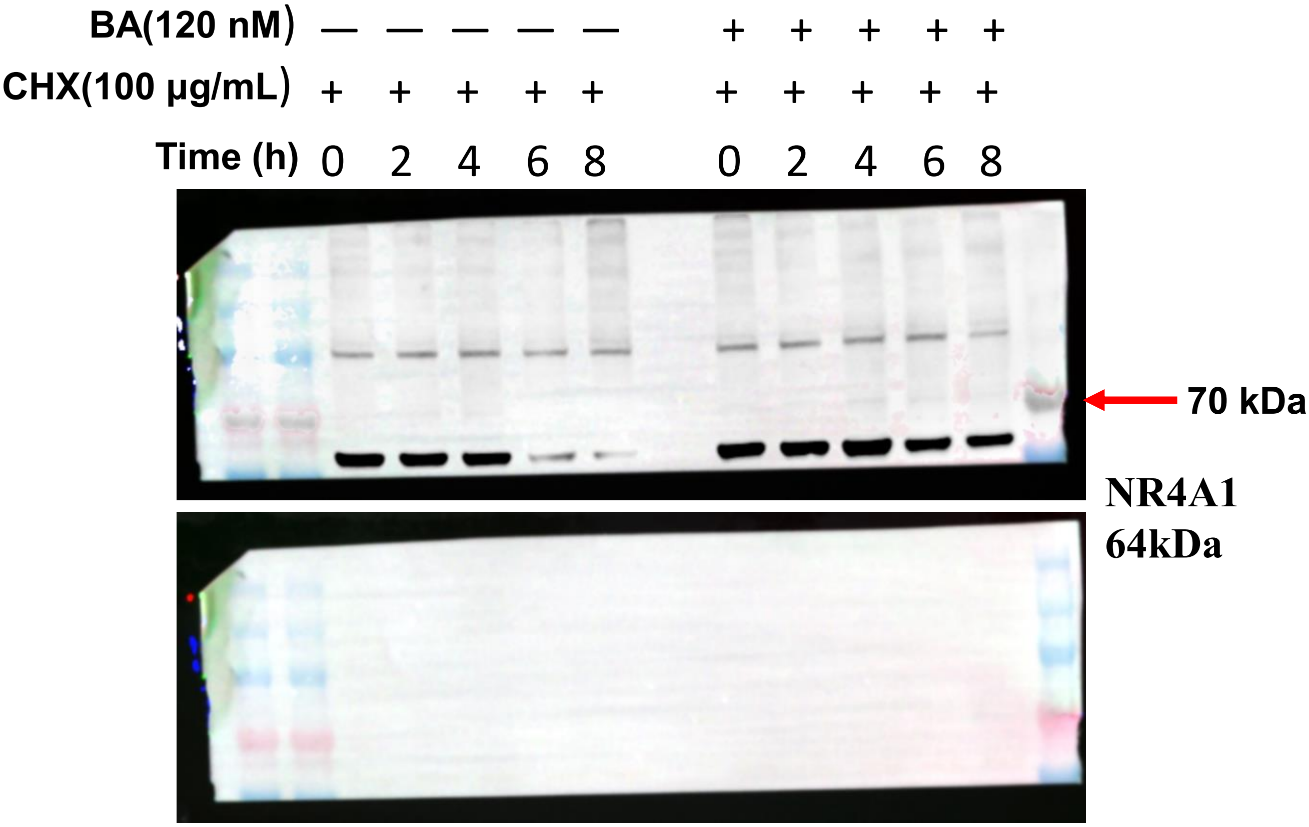

Repeat 3

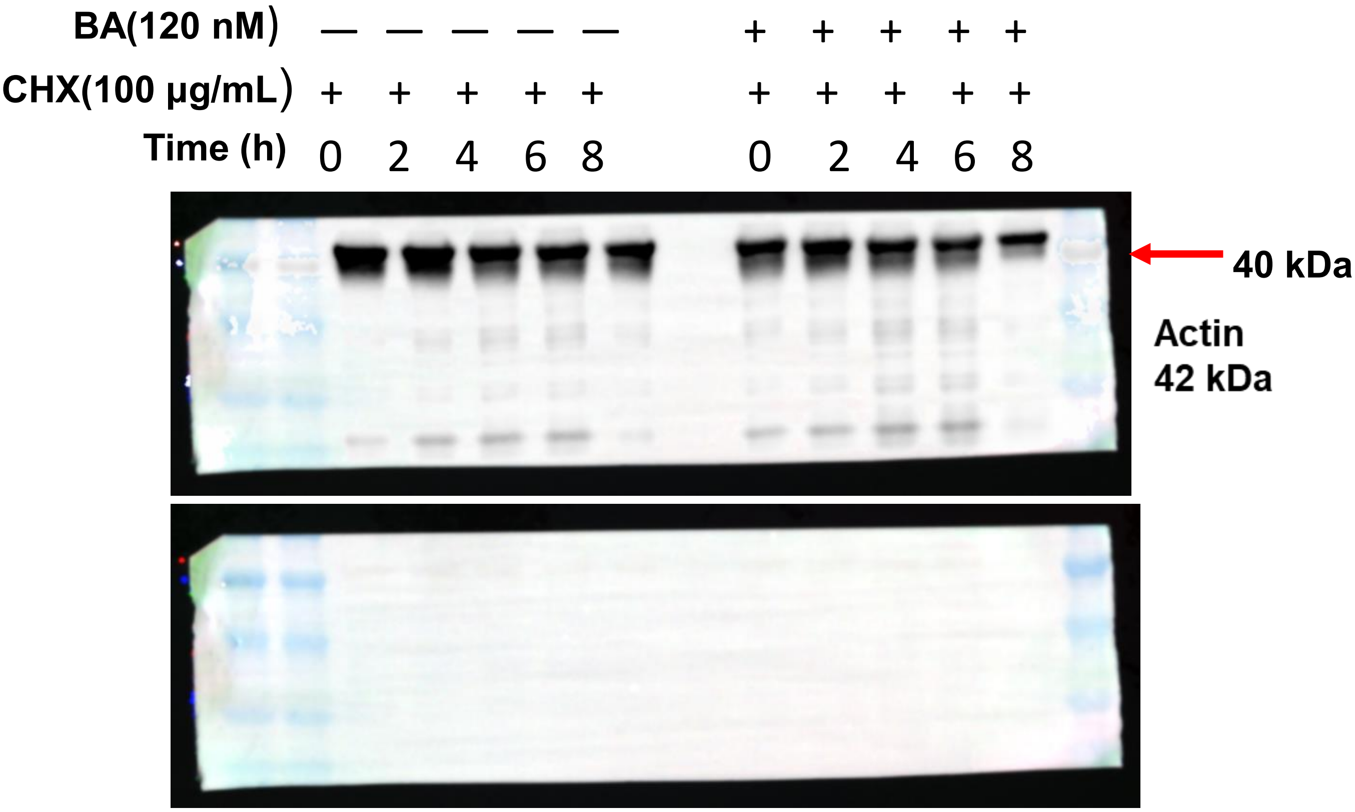

Repeat 3, the merged image

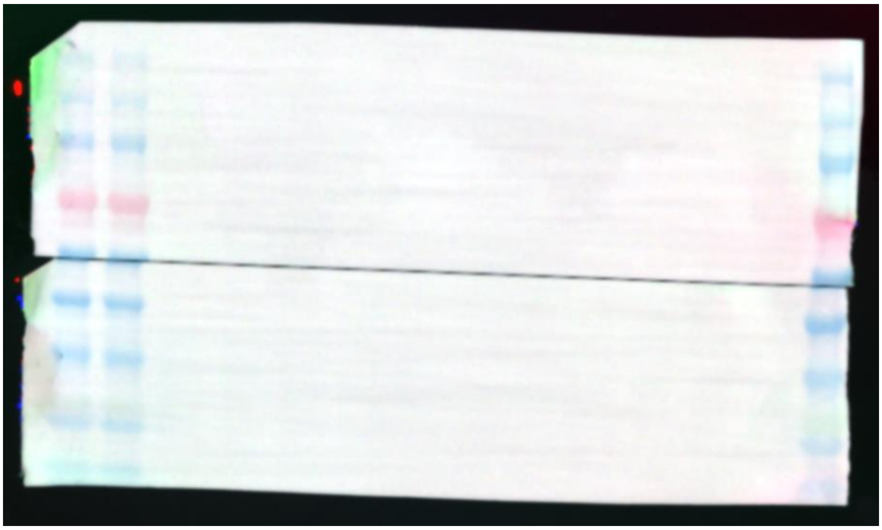

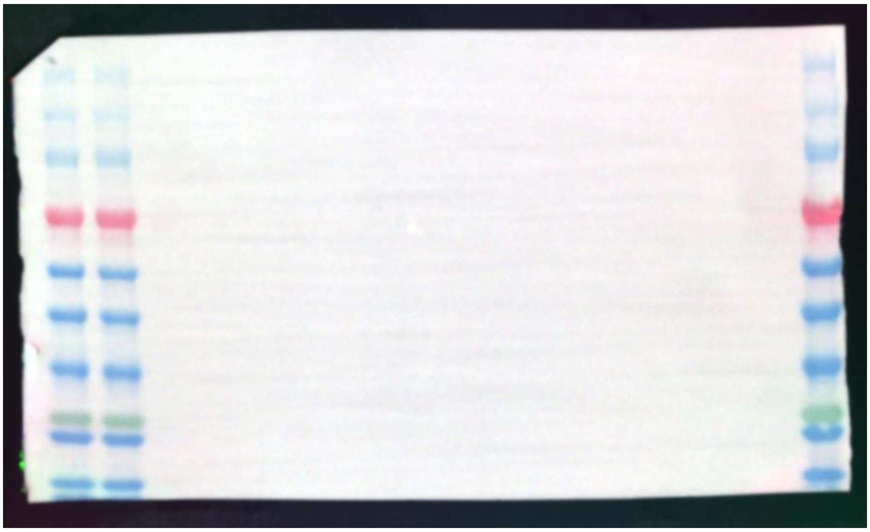

Using MG132 to determine the degradation pathway of the NR4A1 protein

Repeat 1

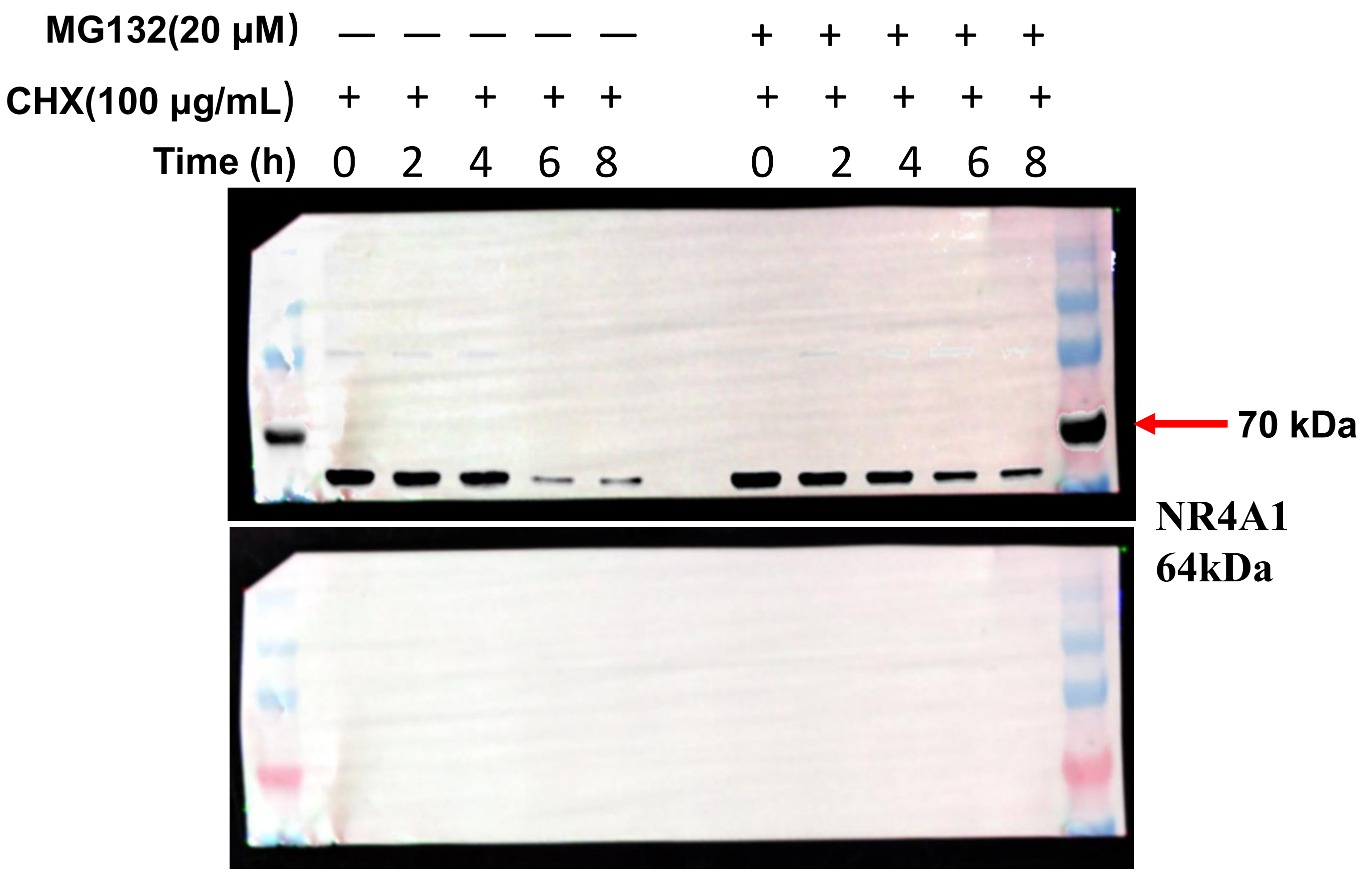

Repeat 1

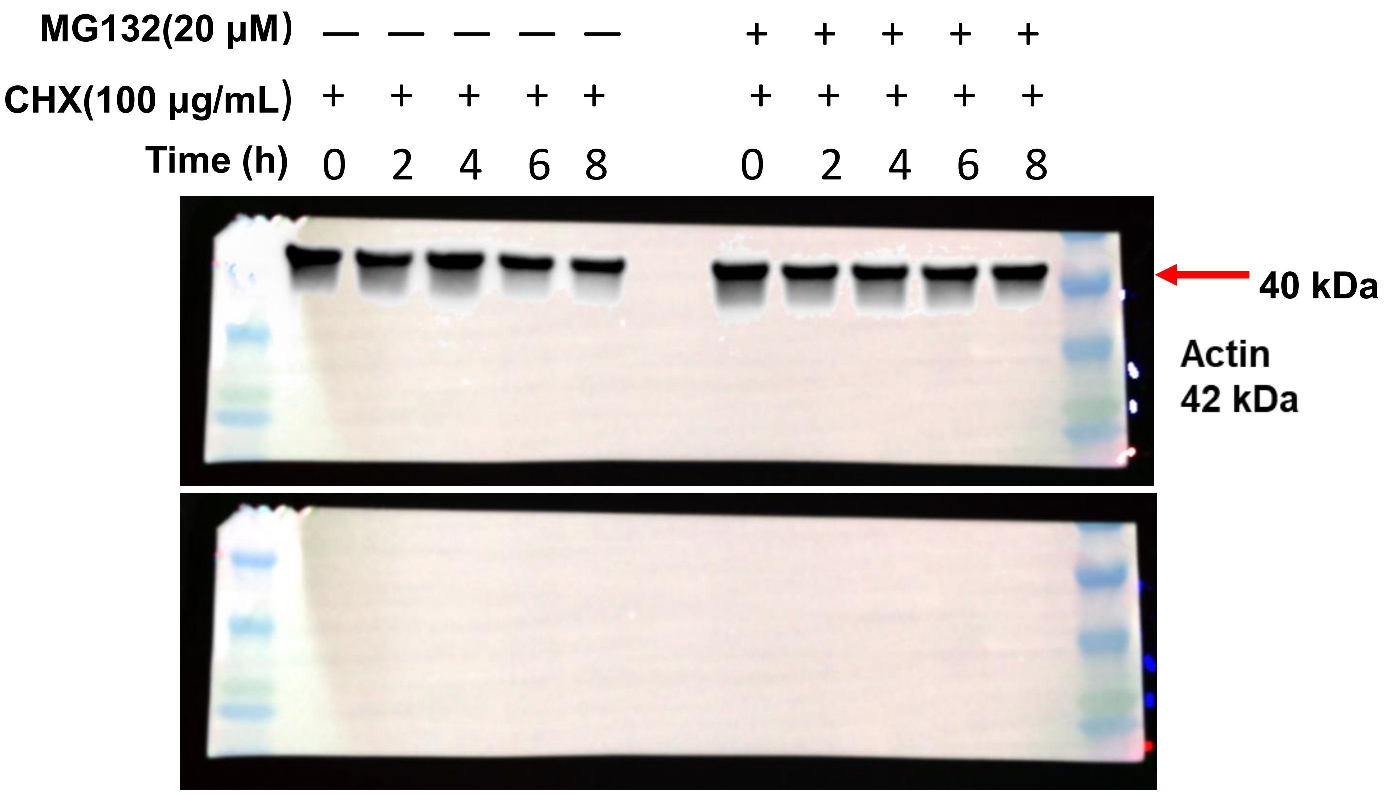

Repeat 1, the merged image

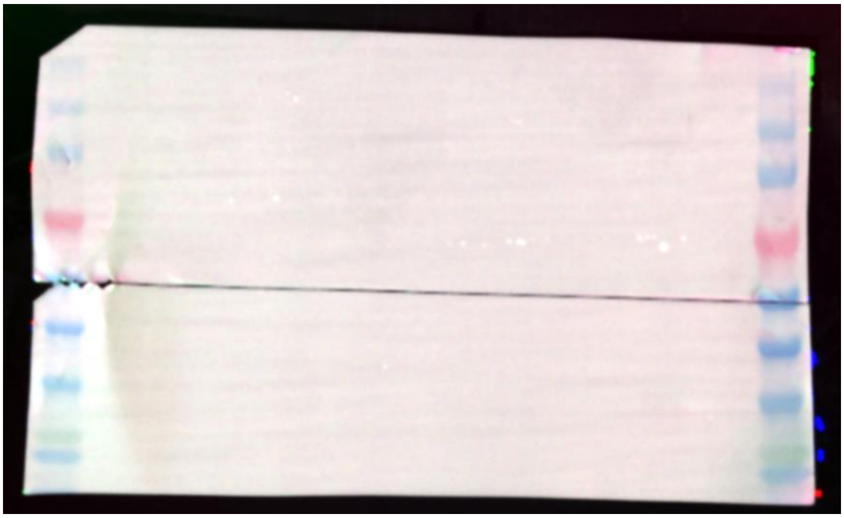

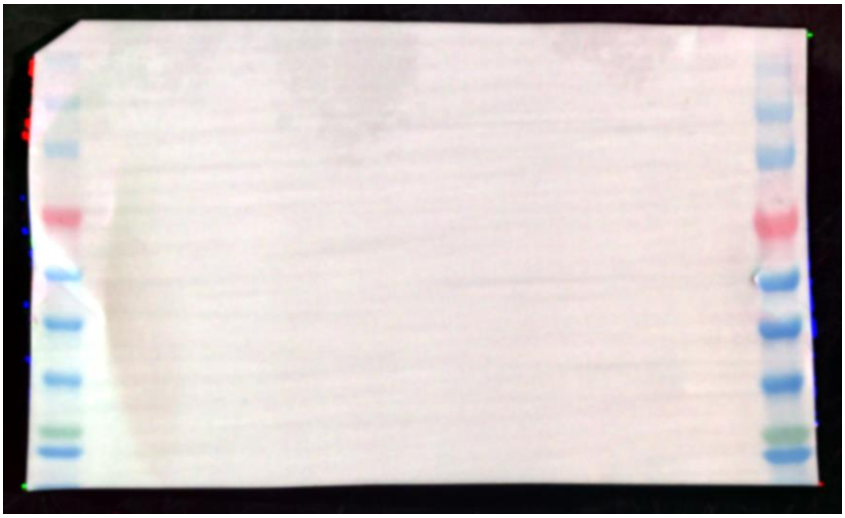

Repeat 2

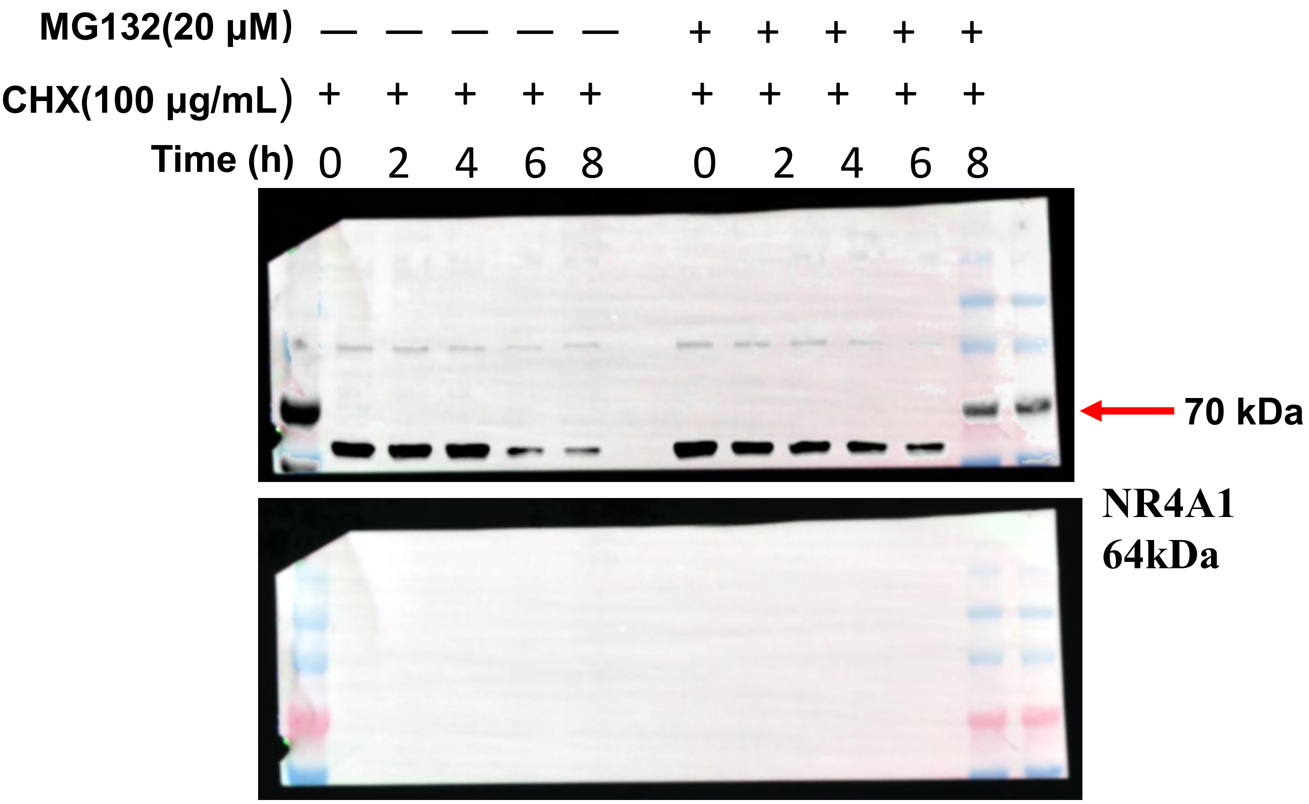

Repeat 2

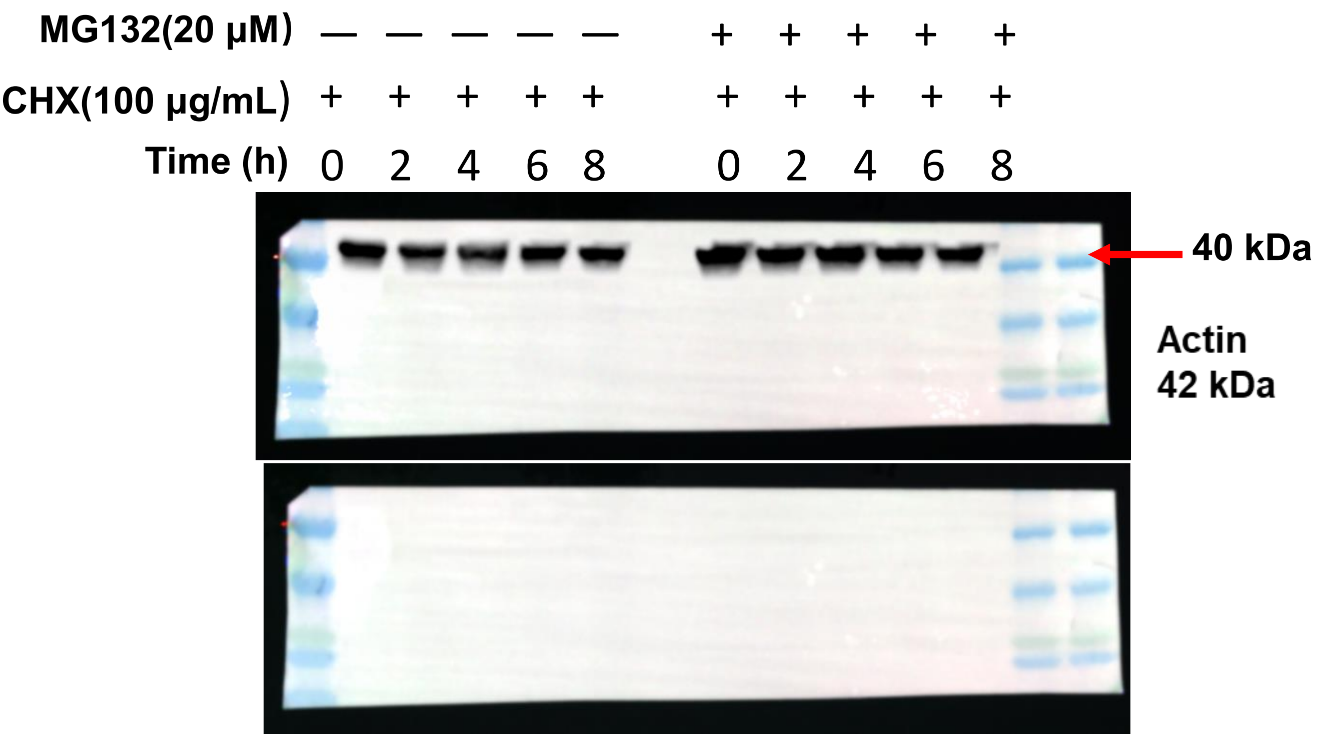

Repeat 2, the merged image

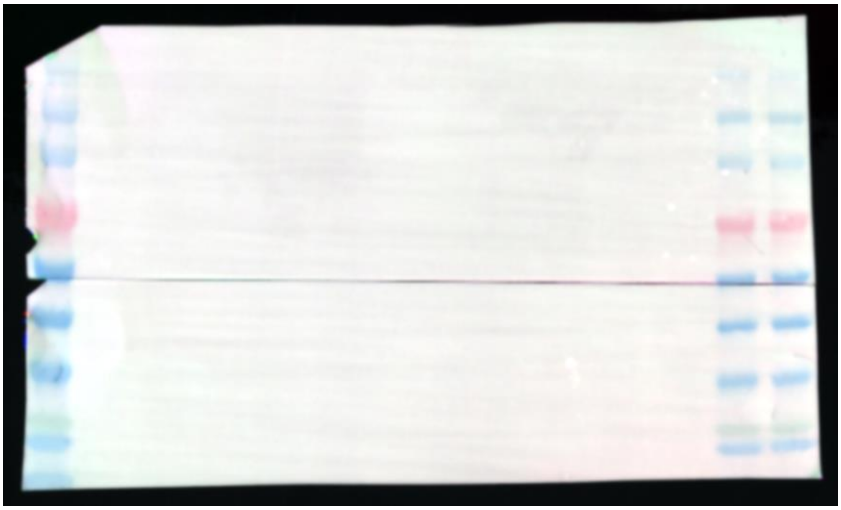

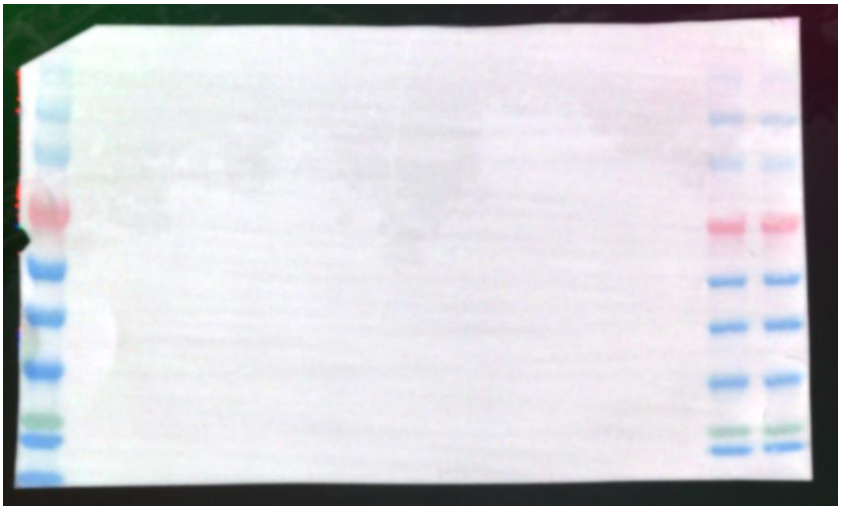

Repeat 3

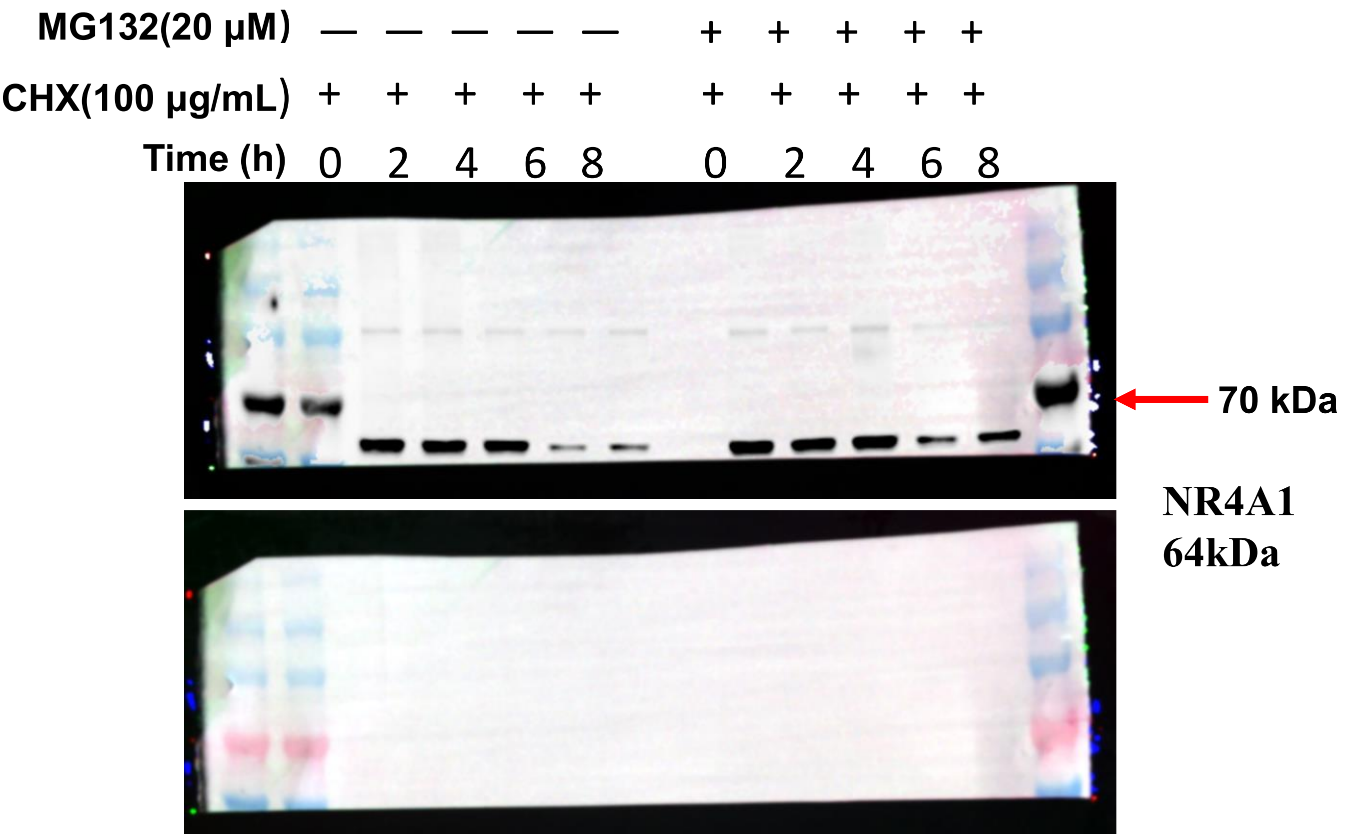

Repeat 3

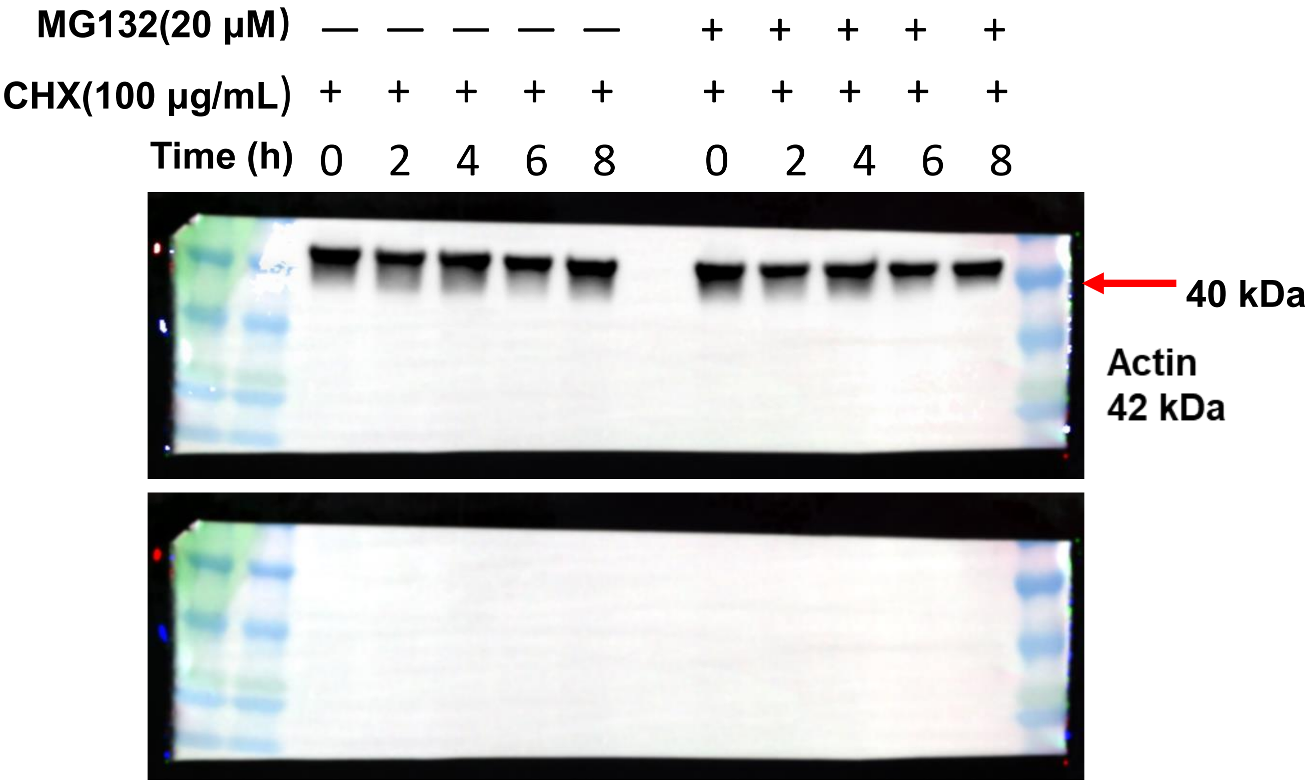

Repeat 3, the merged image

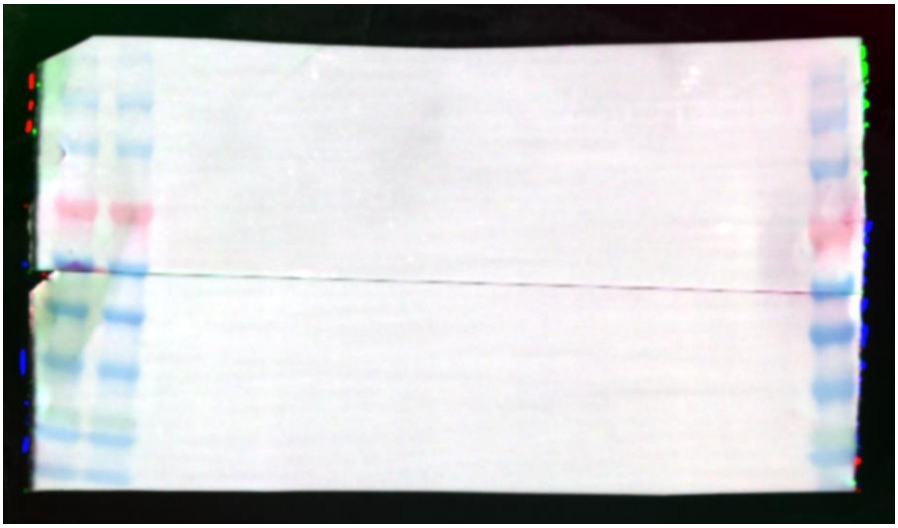

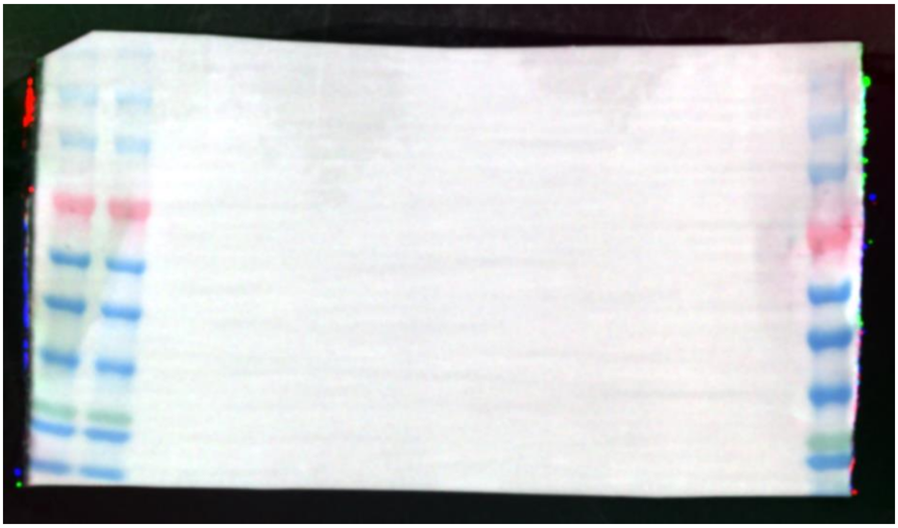

Using Chloroquine to determine the degradation pathway of the NR4A1 protein

Repeat 1

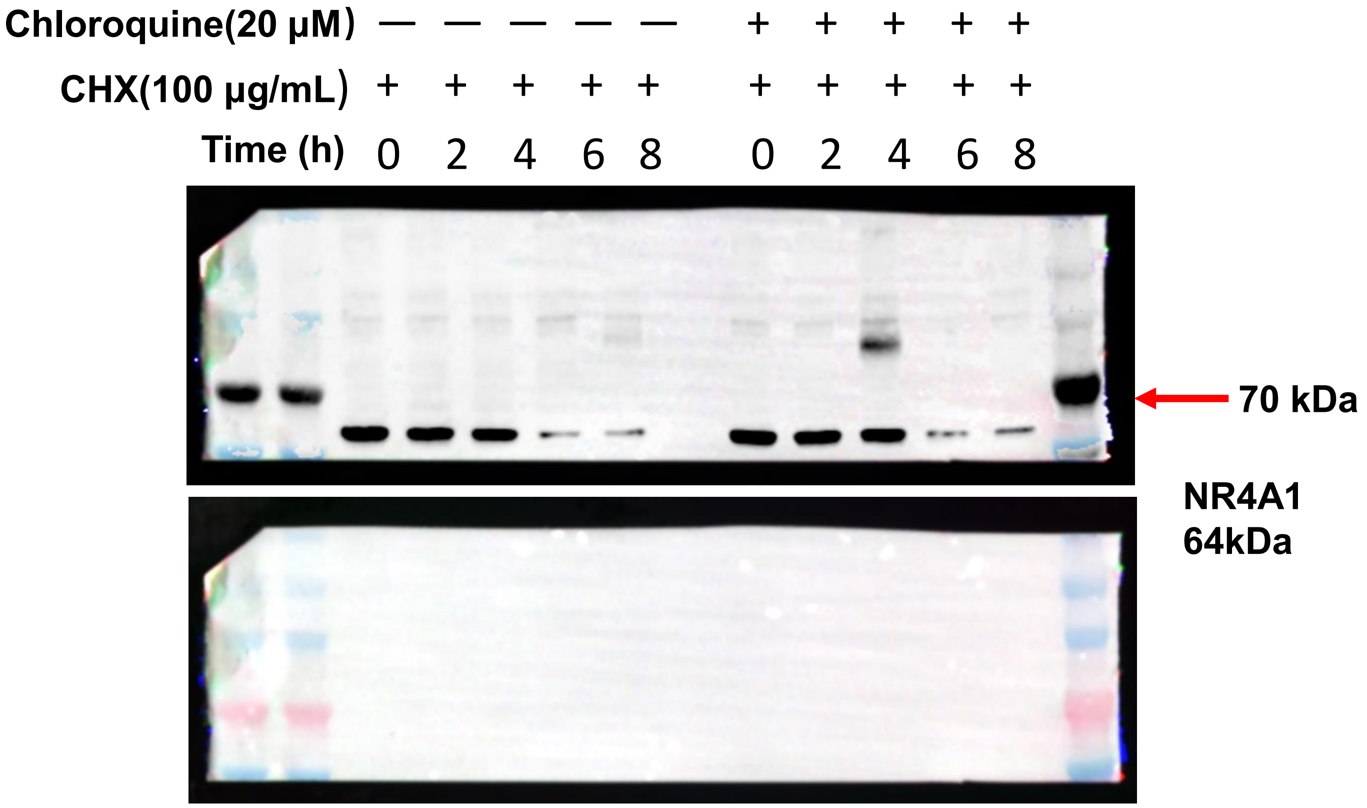

Repeat 1

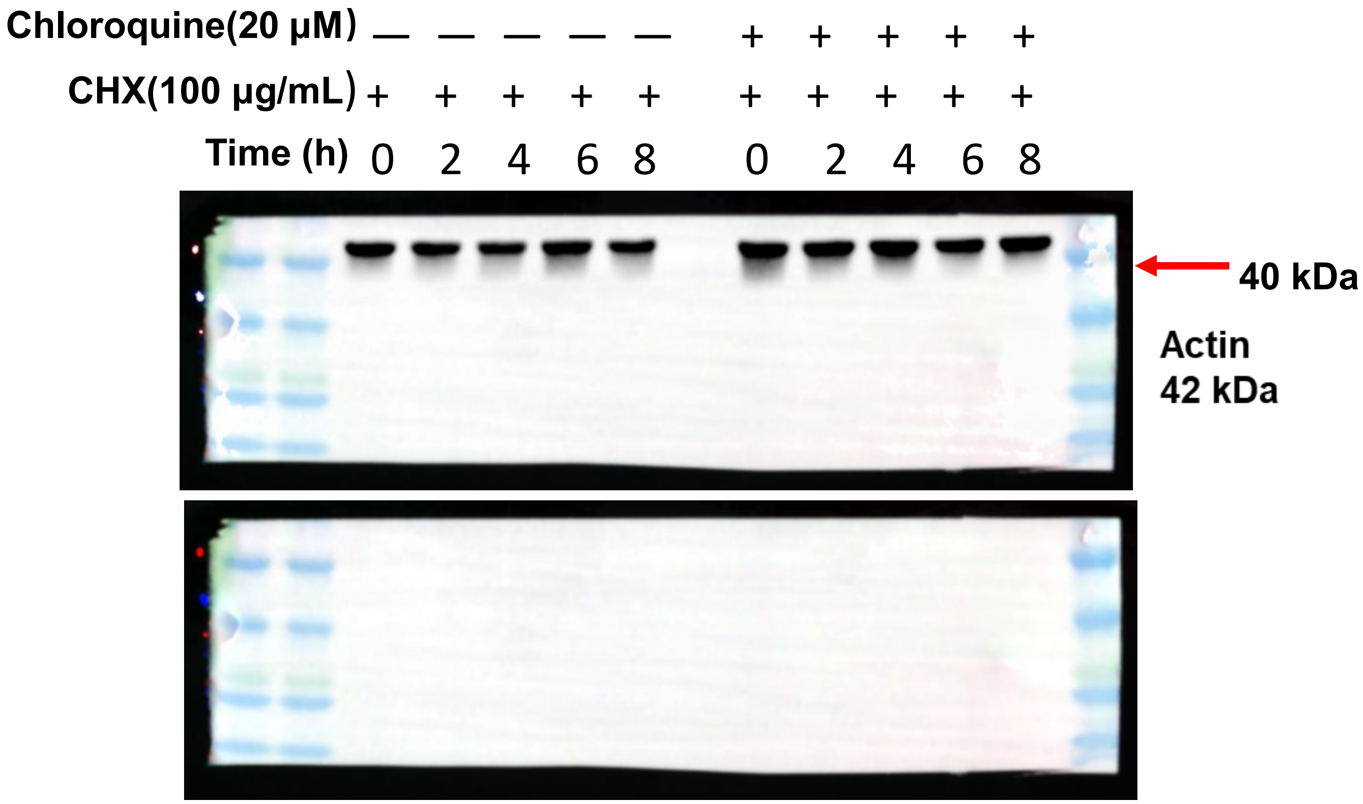

Repeat 1, the merged image

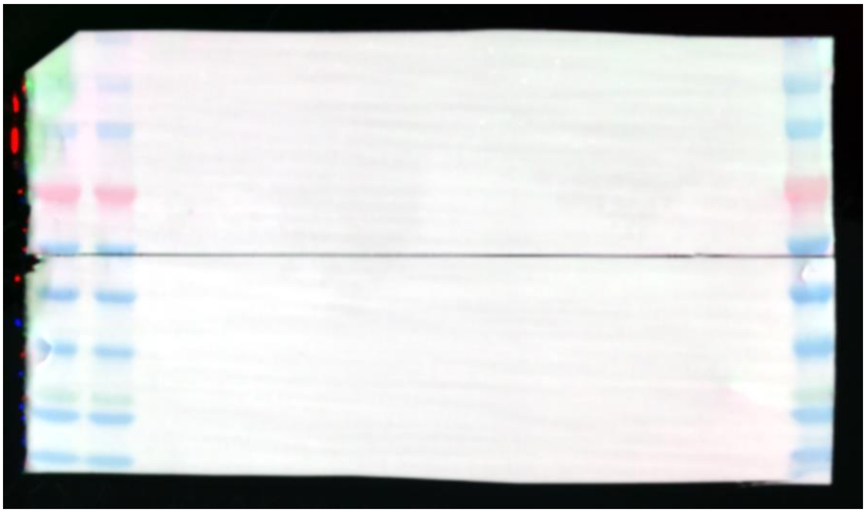

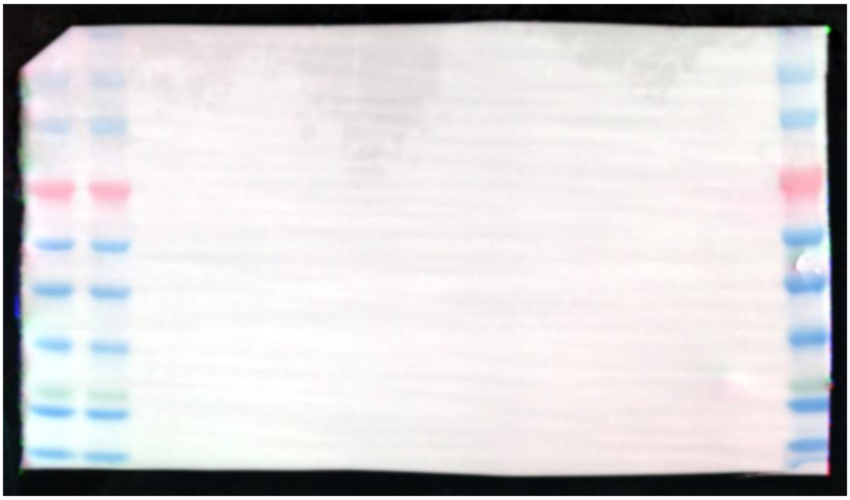

Repeat 2

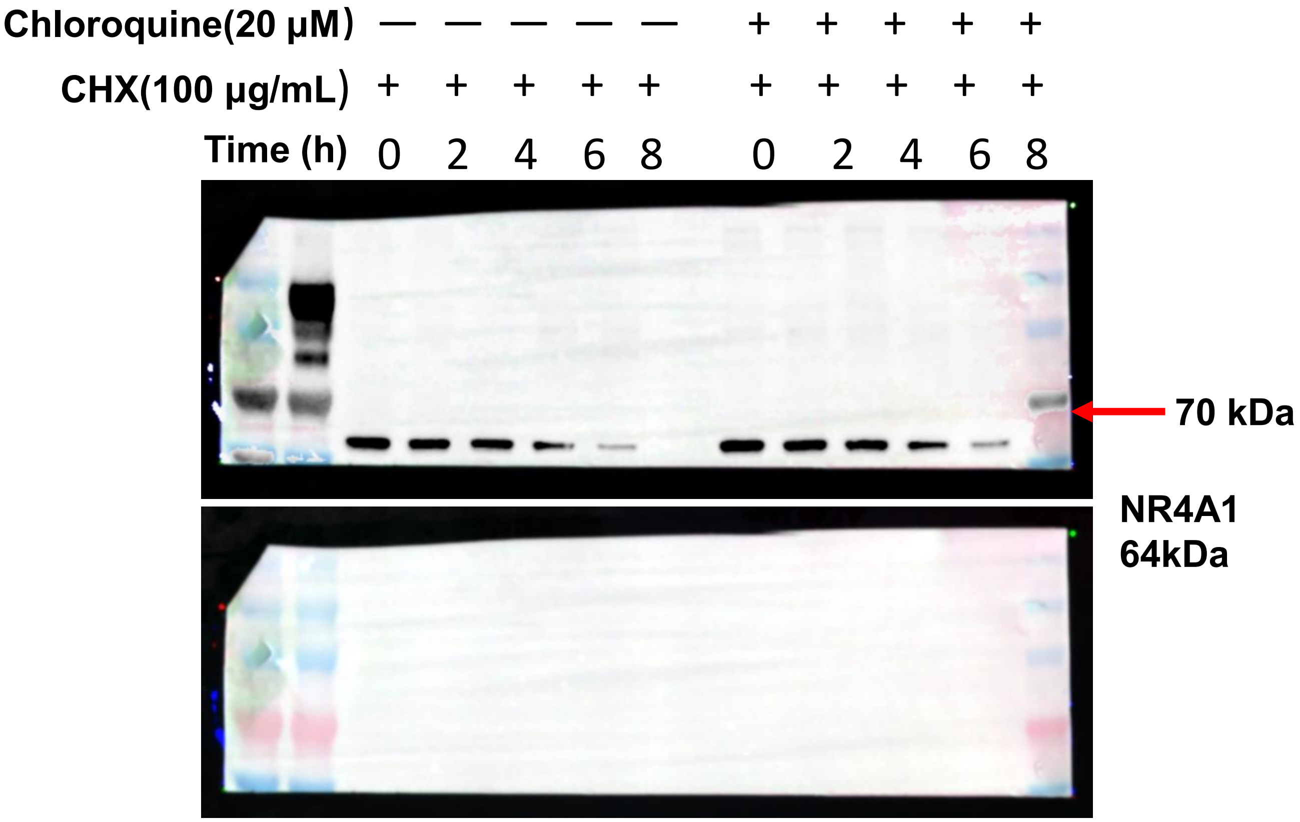

Repeat 2

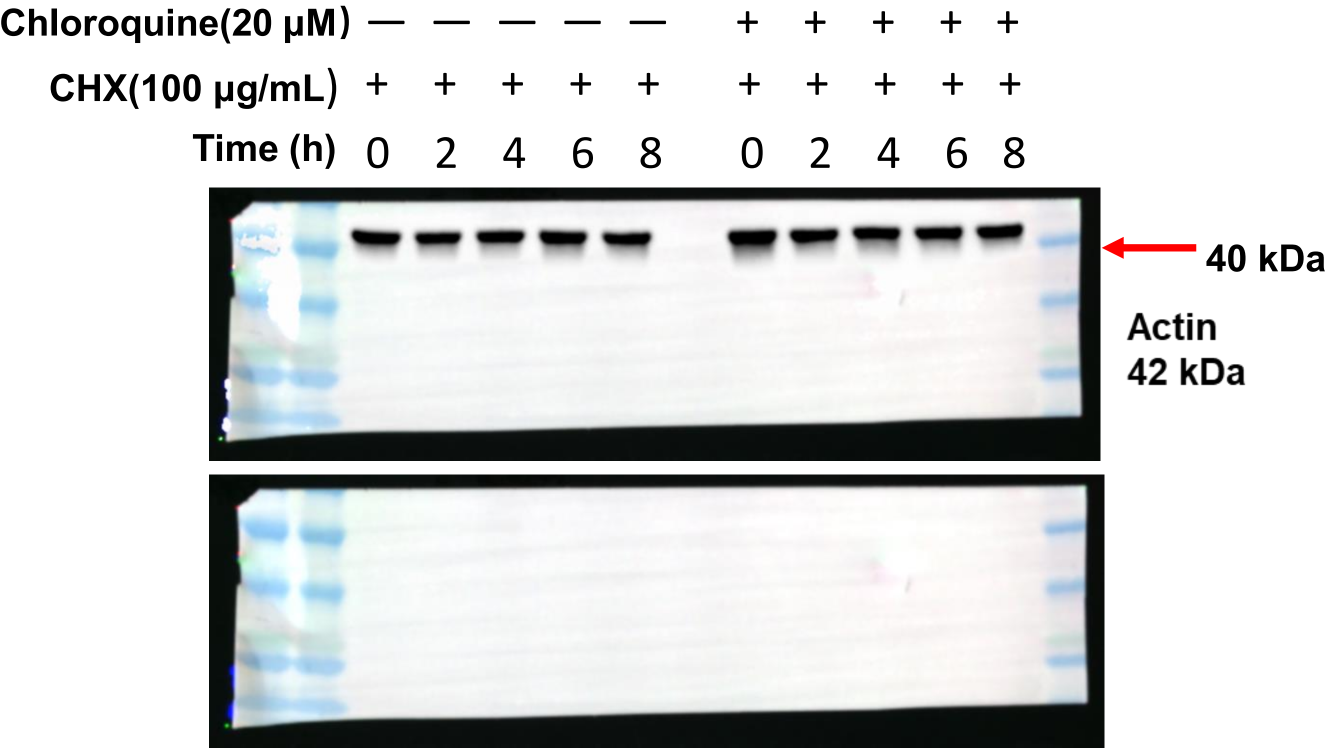

Repeat 2, the merged image

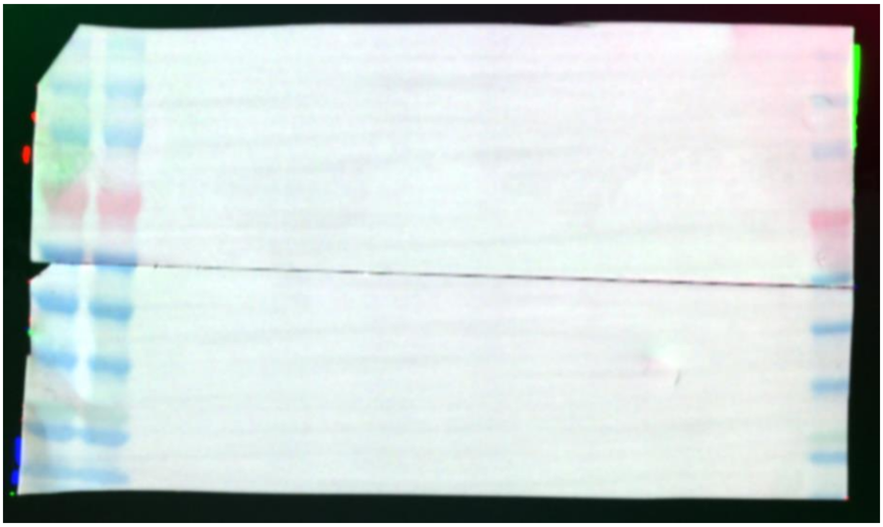

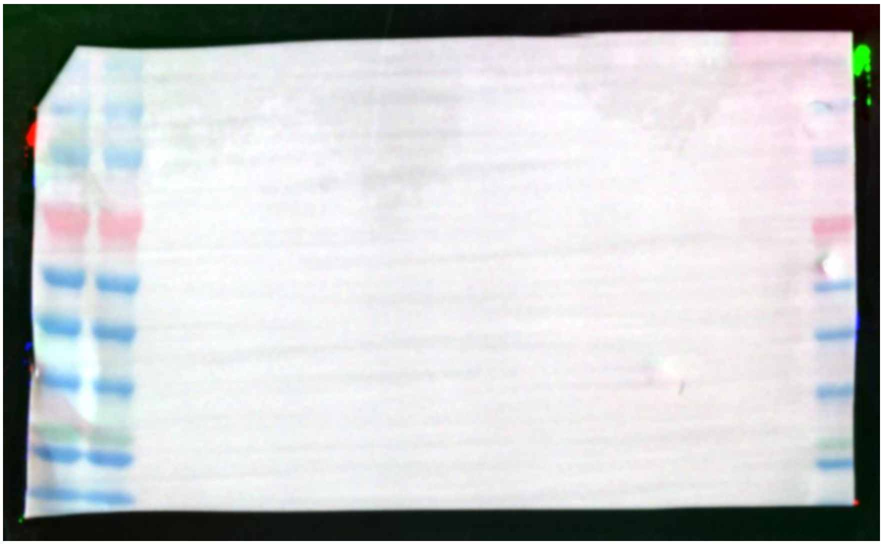

Repeat 3

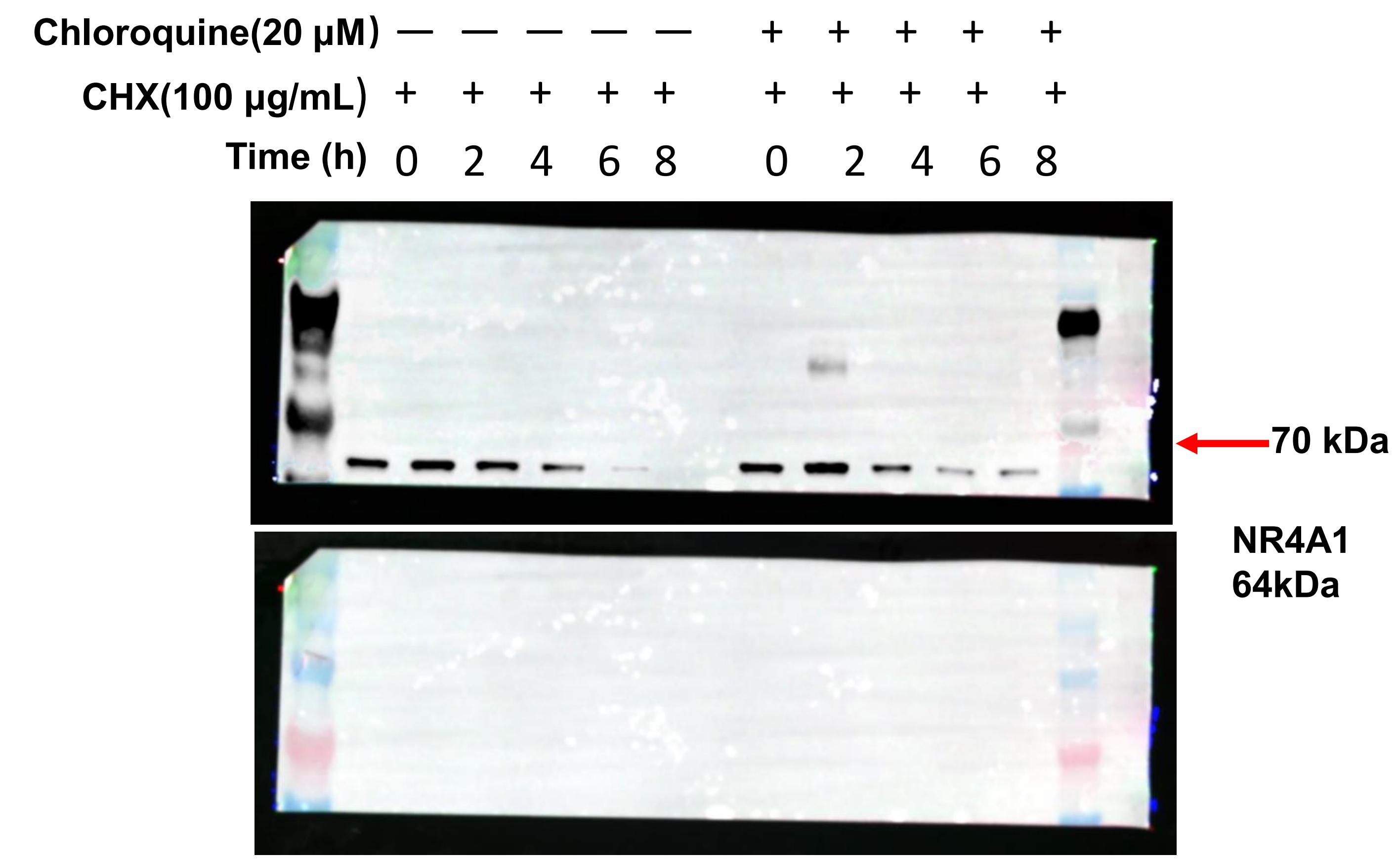

Repeat 3

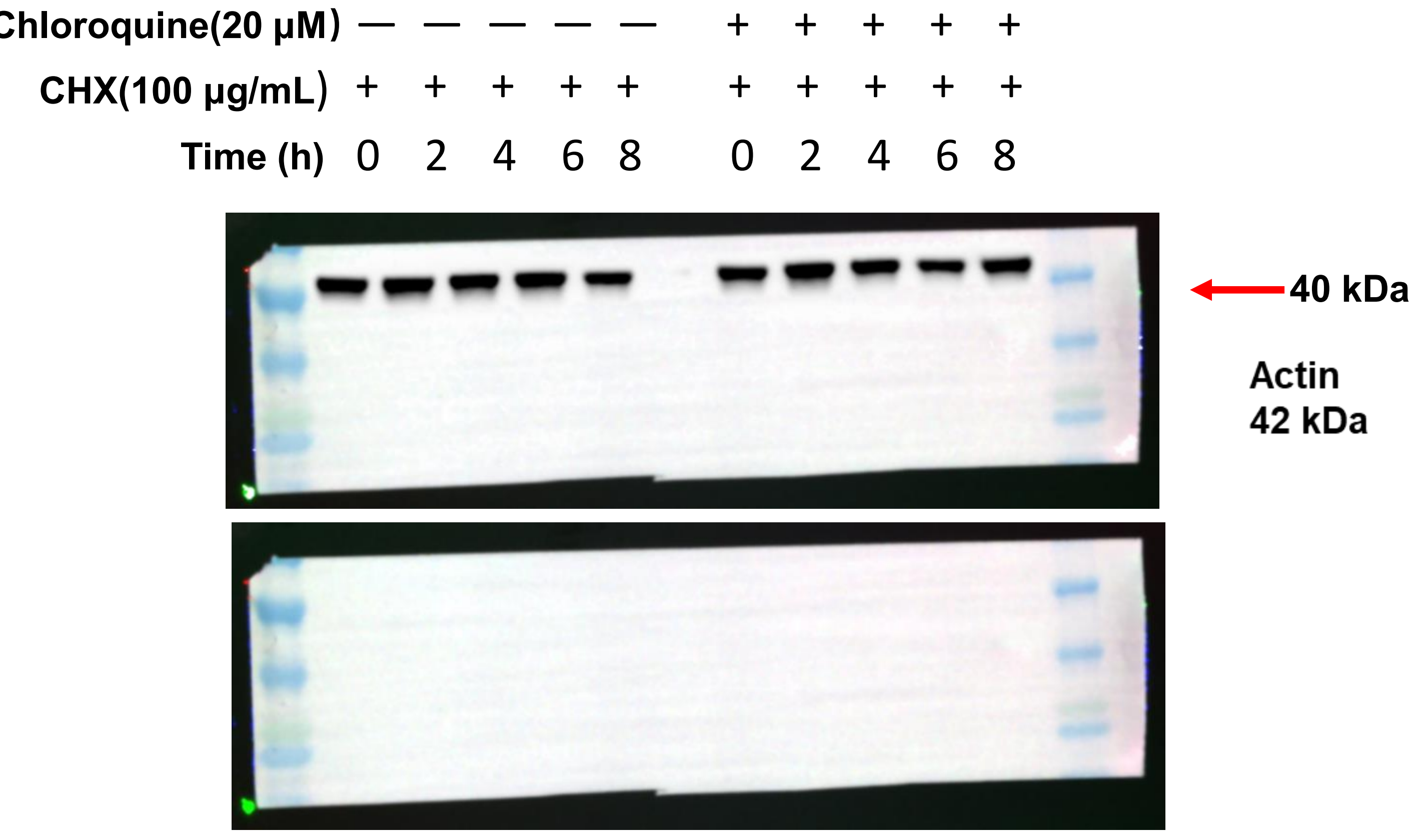

Repeat 3, the merged image

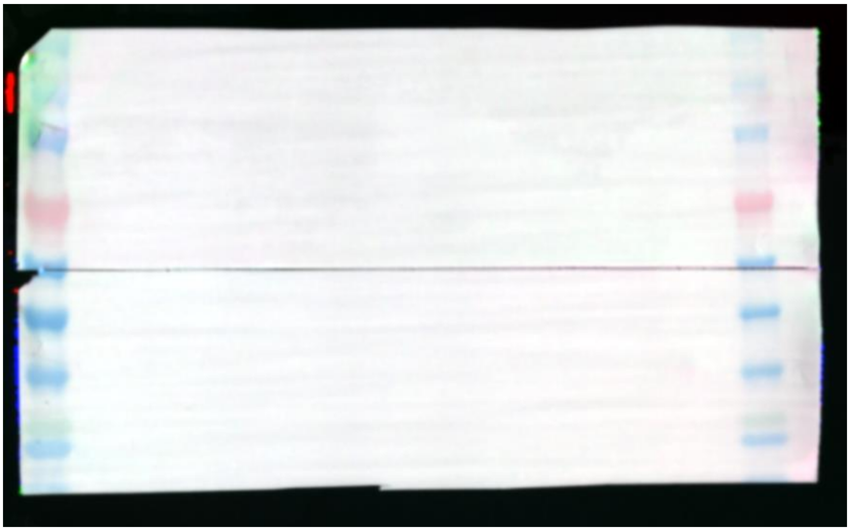

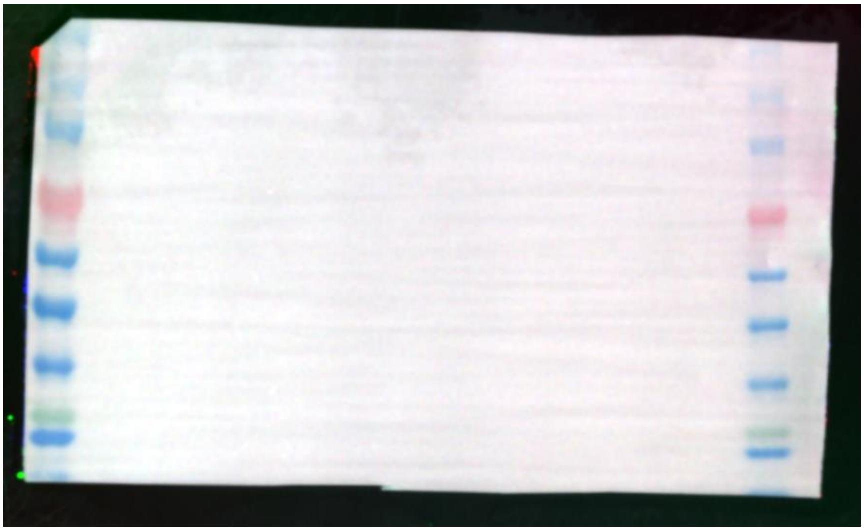

Analysis of ubiquitination of NR4A1

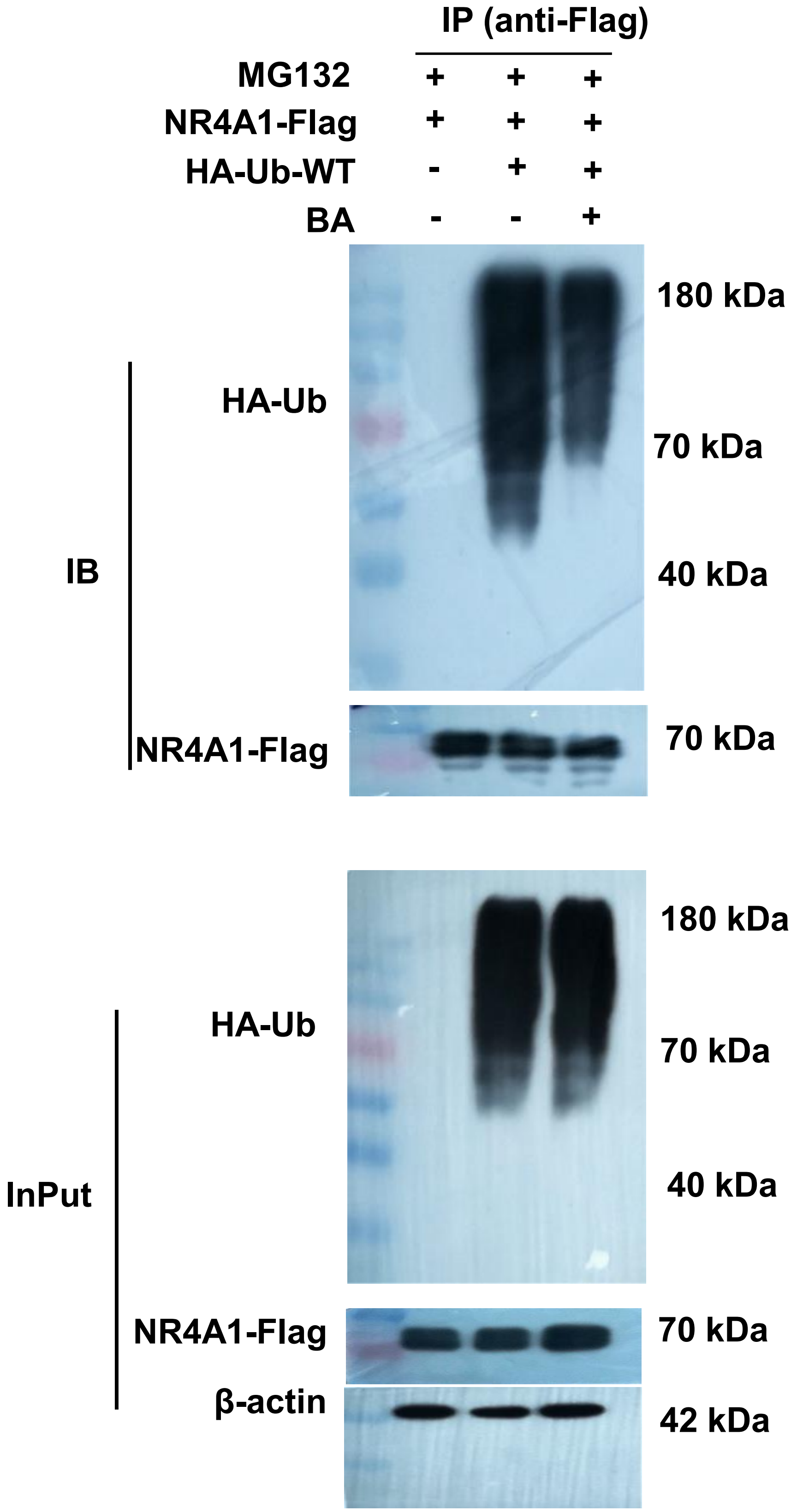

BA attenuates ubiquitination of NR4A1

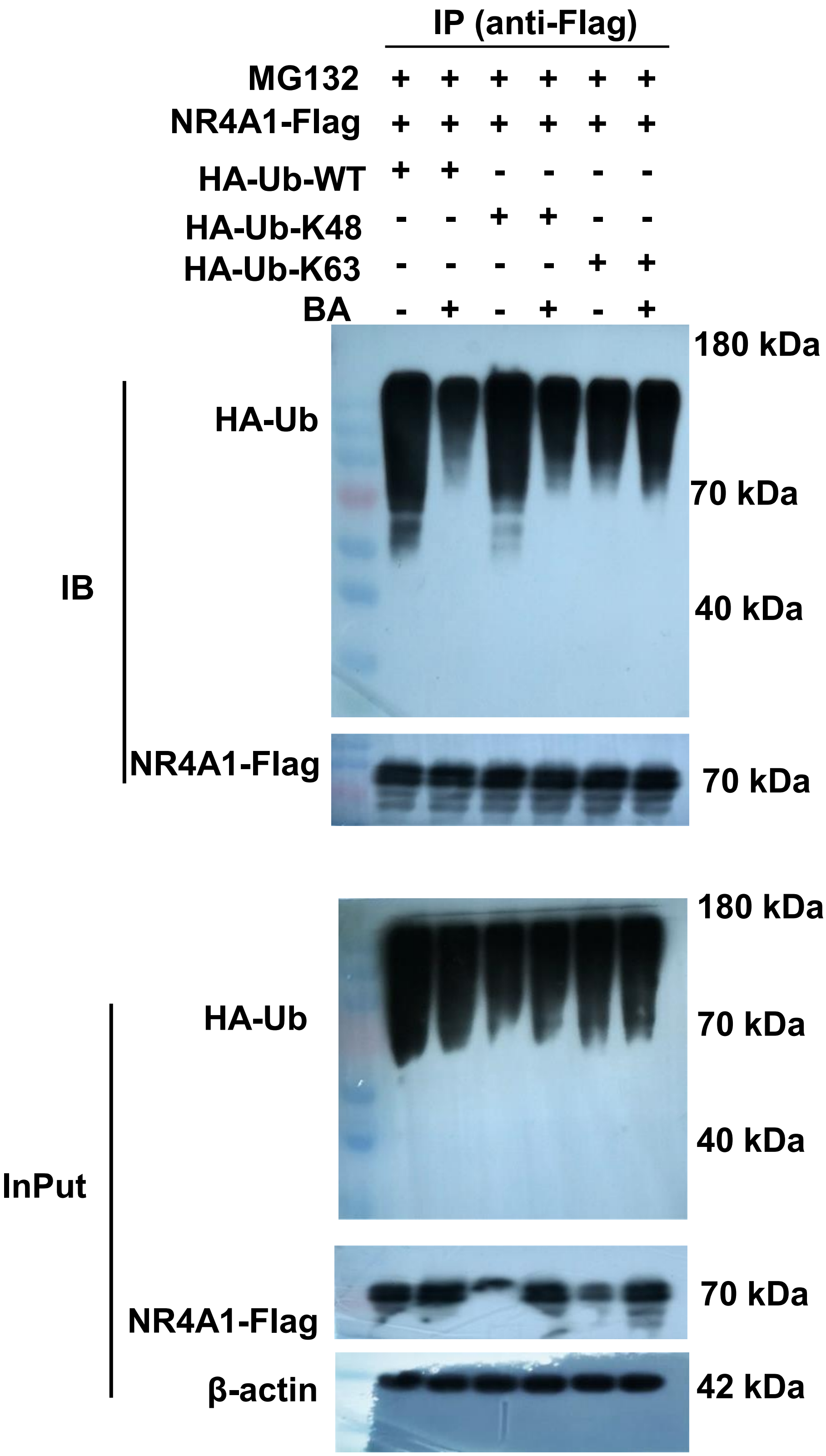

The BA affects the K48 ubiquitin chain of NR4A1

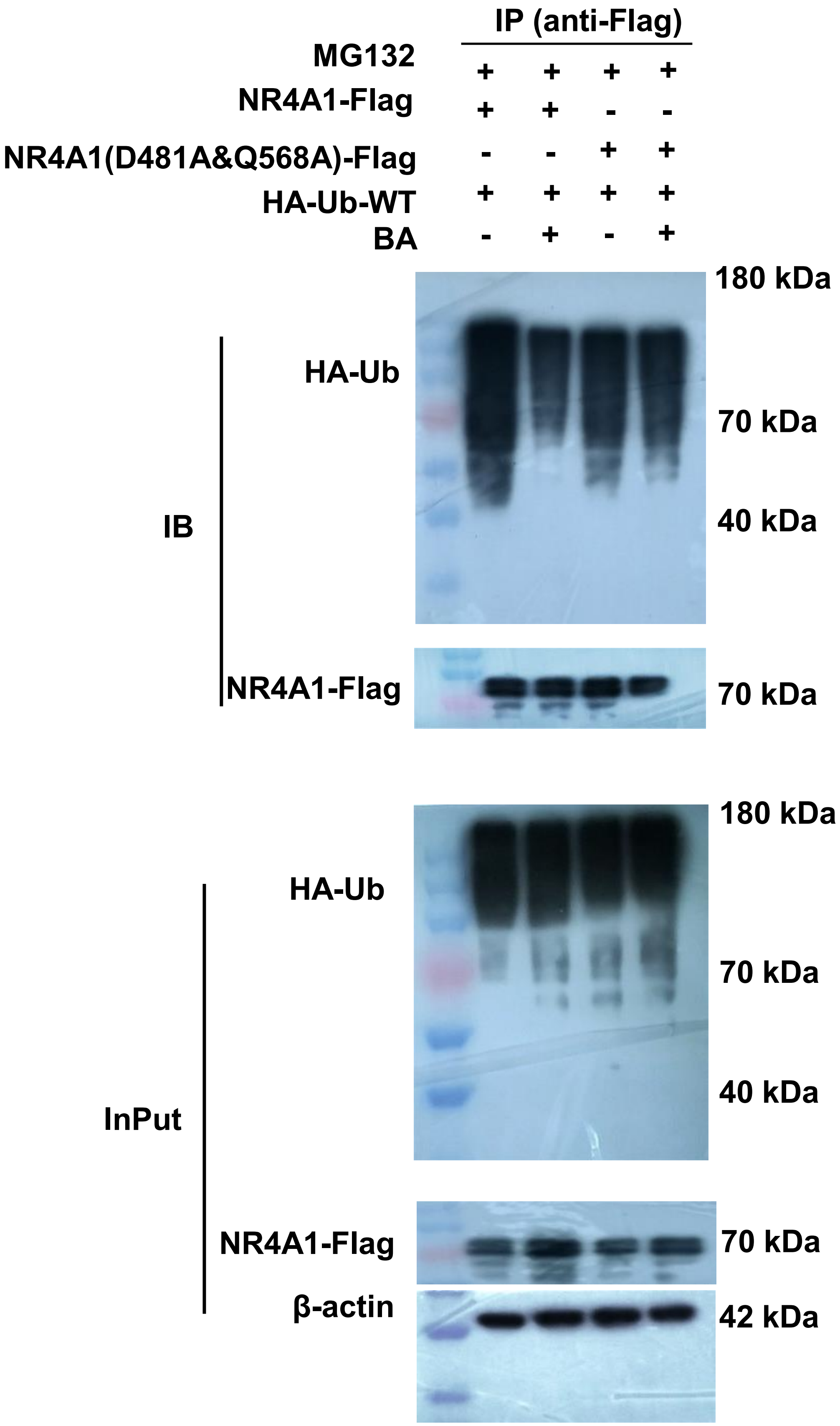

BA binds to D481 and Q568. Mutating these residues to alanine (D481A/Q568A) attenuates BA-mediated suppression of NR4A1 ubiquitination

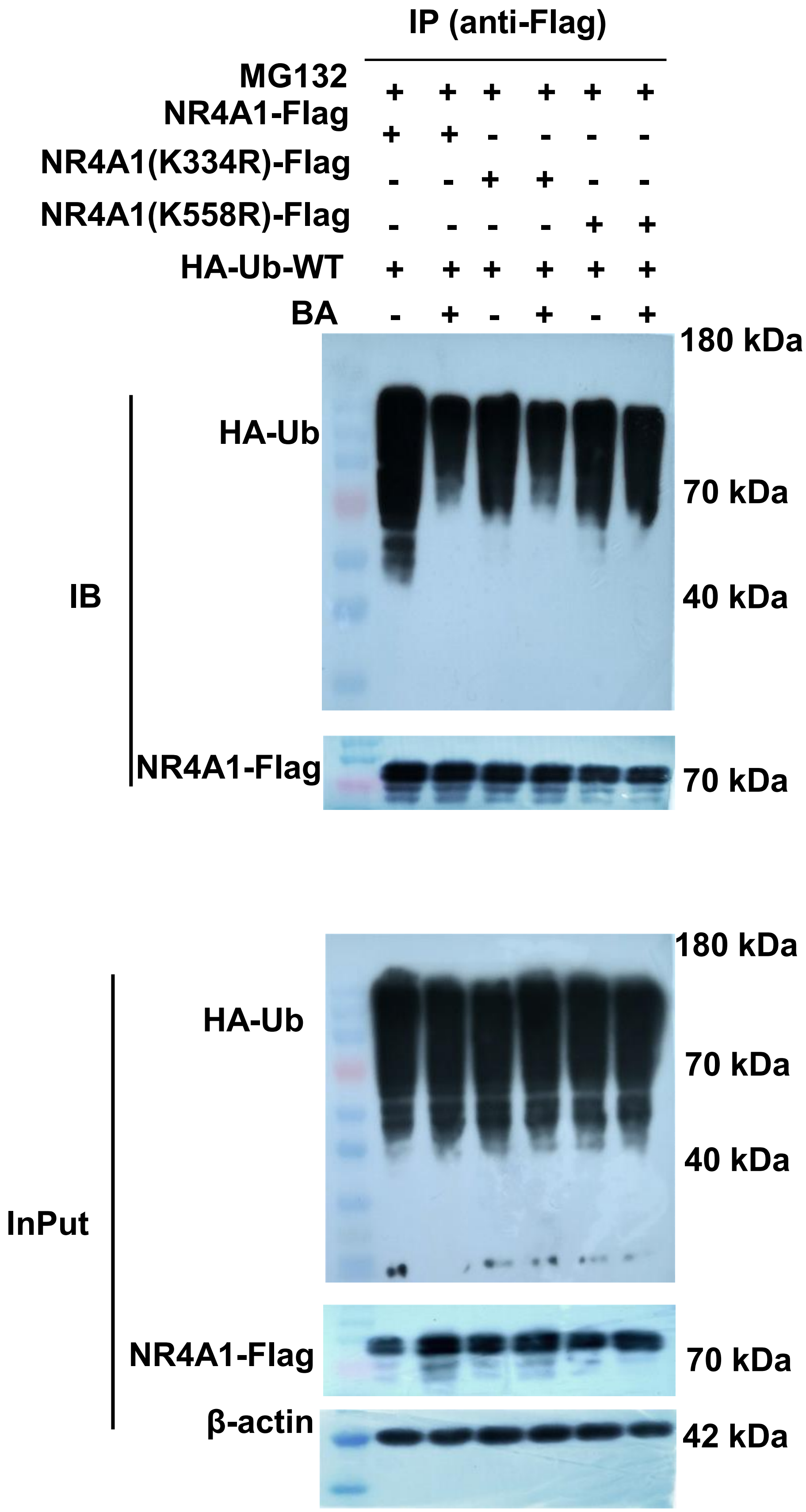

K558 serves as a key residue governing BA's suppression of NR4A1 ubiquitination
