# Supplementary material for: Bruceine A protects nuclear receptor 4A1 from ubiquitin-degradation to alleviate mesangial proliferative glomerulonephritis
Source: Signal Transduct Target Ther. 2025 Dec 5;10:397. doi: 10.1038/s41392-025-02495-2 (PMC12678413; doi:10.1038/s41392-025-02495-2)
Supplement: Supplementary file 3 — Table 1 and Table 2 [file 41392_2025_2495_MOESM3_ESM.docx]

**Table 1.** **gRNA target sequence of NR4A1**

| **Gene** | **gRNA target sequence** |
| --- | --- |
| **NR4A1** | gRNA-A1: AAGACCGGCCACACCCCAGT-GGG |
|  | gRNA-A2: ATCTTACAAAAACACGCGGA-AGG |
|  | gRNA-B1: TGAAAGTCAGCCGATCTGCT-TGG |
|  | gRNA-B2: GCTTGCCCAGCTGGCGGGGT-TGG |

**Table 2.** **Primer sequences of RT-PCR**

| **Gene** | **Forward primer** **(5 ′ –3 ′)** | **Reverse primer (3 ′ –5 ′)** |
| --- | --- | --- |
| **IL-6** | AGAGACTTCCAGCCAGTTGC | AGTCTCCTCTCCGGACTTGT |
| **CCL2** | TAGCATCCACGTGCTGTCTC | CAGCCGACTCATTGGGATCA |
| **NF-κB** | ATGGCAGACGACGATCCTTT | AGGTATGGGCCATCTGTTGAC |
| **CyclinE** | AGGCGAGGATGAGAGCAGTTC | AAGAAGTCCTGTGCCAAGTAGAATG |
| **α-SMA** | ACCATCGGGAATGAACGCTT | CTGTCAGCAATGCCTGGGTA |
| **TGF-β** | AGGGCTACCATGCCAACTTC | CCACGTAGTAGACGATGGGC |
| **FN**  **CyclinB** | GGATCCCCTCCCAGAGAAGT  GATACTGCCTCTCCAAGCCC | GGGTGTGGAAGGGTAACCAG  TGTTCTTGACAGTCCATTCACCA |
| **TNF-α** | ACGTCGTAGCAAACCACCAA | AAATGGCAAATCGGCTGACG |
| **GAPDH** | CAGTGGCAAAGTGGAGATTGTTG | TCGCTCCTGGAAGATGGTGAT |
|  |  |  |
