# Supplementary material for: Bruceine A protects nuclear receptor 4A1 from ubiquitin-degradation to alleviate mesangial proliferative glomerulonephritis
Source: Signal Transduct Target Ther. 2025 Dec 5;10:397. doi: 10.1038/s41392-025-02495-2 (PMC12678413; doi:10.1038/s41392-025-02495-2)
Supplement: Supplementary file 4 — Table 3 [file 41392_2025_2495_MOESM4_ESM.docx]

**Table 3. Characteristics of Participants**

| Study ID | Group | Gender | Age (Years) | eGFR (mL/min/1.73 m2) | CKD stage | Specimens | Pathological Diagnosis (Lee grading, Oxford classification) |
| --- | --- | --- | --- | --- | --- | --- | --- |
| IgAN_01 | IgAN | female | 33 | 93.52 | 1 | kidney tissue | IgAN (Ⅱ, M1E0S1T0C0) |
| IgAN_02 | IgAN | female | 34 | 115.85 | 1 | kidney tissue | IgAN (Ⅲ, M1E0S1T0C0) |
| IgAN_03 | IgAN | female | 50 | 64.44 | 2 | kidney tissue | IgAN (Ⅲ, M1E0S1T0C1) |
| IgAN_04 | IgAN | female | 49 | 70.54 | 2 | kidney tissue | IgAN (Ⅲ, M1E0S1T0C0) |
| IgAN_05 | IgAN | female | 35 | 61.54 | 2 | kidney tissue | IgAN (Ⅱ, M1E0S0T0C0) |
| IgAN_06 | IgAN | male | 44 | 49.28 | 3a | kidney tissue | IgAN (Ⅳ, M1E0S1T1C1) |
| IgAN_07 | IgAN | male | 54 | 43.08 | 3b | kidney tissue | IgAN (Ⅳ, M1E1S1T1C1) |
| IgAN_08 | IgAN | female | 36 | 46.01 | 3a | kidney tissue | IgAN (Ⅴ, M1E1S1T2C0) |
| NM_01 | normal control | male | 67 | 73.41 | 2 | kidney tissue | Urothelial carcinoma |
| NM_02 | normal control | female | 56 | 93.51 | 1 | kidney tissue | Hydronephrosis with kidney stones |
| NM_03 | normal control | male | 49 | 106.84 | 1 | kidney tissue | Chromophobe renal cell carcinoma |
| NM_04 | normal control | female | 73 | 46.68 | 3a | kidney tissue | Clear cell renal cell carcinoma |
| NM_05 | normal control | female | 39 | 101.28 | 1 | kidney tissue | Renal cell carcinoma |
| NM_06 | normal control | female | 33 | 75.64 | 2 | kidney tissue | Urothelial carcinoma |
| NM_07 | normal control | female | 45 | 105.54 | 1 | kidney tissue | Renal angiomyolipoma |
| NM_08 | normal control | female | 65 | 63.04 | 2 | kidney tissue | Urothelial carcinoma |

eGFR, estimated glomerular filtration rate; CKD, chronic kidney disease.
