# Supplementary material for: Bruceine A protects nuclear receptor 4A1 from ubiquitin-degradation to alleviate mesangial proliferative glomerulonephritis
Source: Signal Transduct Target Ther. 2025 Dec 5;10:397. doi: 10.1038/s41392-025-02495-2 (PMC12678413; doi:10.1038/s41392-025-02495-2)
Supplement: Supplementary file 5 — Table 4 [file 41392_2025_2495_MOESM5_ESM.docx]

| Variables | IgAN (n=8) | NM (n=8) | *P* value |
| --- | --- | --- | --- |
| Gender (n,%) |  |  |  |
| male | 2 (25.0) | 2 (25.0) |  |
| female | 6 (75.0) | 6 (75.0) |  |
| Age [years, mean ± sd] | 41.9 ± 8.4 | 53.4 ± 14.3 | 0.094 |
| Body temperature (℃) | 36.4 ± 0.2 | 36.4 ± 0.2 | 0.801 |
| MAP (mmHg) | 89.4 ± 8.7 | 100.4 ± 9.8 | 0.794 |
| Laboratory values(mean ± sd) |  |  |  |
| Serum creatinine (μmol/L) | 104.8 ± 35.4 | 79.7 ± 20.9 | 0.096 |
| Urea (mmol/L) | 5.5 ± 1.7 | 5.3 ± 1.6 | 0.464 |
| Uric Acid (μmol/L) | 377.3 ± 85.2 | 372.0 ± 93.8 | 0.951 |
| eGFR [ml/(min•1.73m2)] | 68.0 ± 25.2 | 83.2 ± 22.0 | 0.95 |
| Serum albumin (g/L) | 41.8 ± 2.8 | 42.2 ± 5.0 | 0.246 |
| ALT (U/L) | 17.6 ± 9.0 | 17.6 ± 12.1 | 0.687 |
| AST (U/L) | 19.1 ±3.9 | 20.3 ± 14.6 | 0.18 |
| Hemoglobin (g/L) | 119.1 ± 21.5 | 119.1 ± 23.3 | 0.898 |
| WBC (10^9/L) | 7.4 ± 3.4 | 8.1 ± 1.9 | 0.512 |
| RBC (10^12/L) | 7.8 ± 10.3 | 8.7 ± 11.4 | 0.863 |
| PLT (10^9/L) | 260.0 ± 68.0 | 218.4 ± 70.1 | 0.644 |
| Serum Calcium (mmol/L) | 2.3 ± 0.2 | 1.1 ± 0.9 | 0.14 |
| Phosphorus (mmol/L) | 1.2 ± 0.2 | 1.1 ± 0.1 | 0.241 |
| FPG (mmol/L) | 5.8 ± 0.8 | 7.8 ± 3.7 | 0.117 |
| HbA1c (%) | 5.6 ± 0.4 | 5.9 ± 0.8 | 0.088 |
| Triglyceride (mmol/L) | 1.5 ± 0.6 | 2.3 ± 1.6 | 0.146 |
| Total Cholesterol (mmol/L) | 4.9 ± 1.0 | 4.4 ± 1.1 | 0.887 |
| HDL-C (mmol/L) | 1.2 ± 0.2 | 1.0 ± 0.4 | 0.058 |
| LDL-C (μmol/L) | 3.1 ± 0.9 | 2.4 ± 1.0 | 0.646 |
| IgA (g/L) | 2.9 ± 1.1 | — | — |
| IgG (g/L) | 9.0 ± 3.8 | — | — |
| IgM (g/L) | 1.1 ± 0.5 | — | — |
| C3 (g/L) | 1.0 ± 0.2 | — | — |
| C4 (g/L) | 0.3 ± 0.1 | — | — |
| UPCR (mg/g) | 964.1 ± 678.1 | — | — |
| 24h Proteinuria (mg) | 1070.8 ± 773.6 | — | — |

**Table 4. Demographic and Clinical Characteristics of the Participants at Baseline**

IgA nephropathy, IgAN; NM, normal; MAP, mean arterial pressure; eGFR, estimated glomerular filtration rate; ALT, alanine aminotransferase; AST, aspartate aminotransferase; WBC, white blood cell; RBC, red blood cell; PLT, blood platelet; FPG, fasting plasma glucose; HDL-C, high density lipoprotein cholesterol; LDL-C, low-density lipoprotein cholesterol; UPCR, urinary protein-to-creatinine ratio.
