# Supplementary material for: Bruceine A protects nuclear receptor 4A1 from ubiquitin-degradation to alleviate mesangial proliferative glomerulonephritis
Source: Signal Transduct Target Ther. 2025 Dec 5;10:397. doi: 10.1038/s41392-025-02495-2 (PMC12678413; doi:10.1038/s41392-025-02495-2)
Supplement: Supplementary file 6 — Table 5 [file 41392_2025_2495_MOESM6_ESM.docx]

**Table 5. Basic Information and Pathological Diagnosis of Single-Cell Transcriptome Clinical Samples**

| Study ID | Group | Gender | Age (Years) | eGFR (mL/min/1.73 m^2^) | CKD stage | Specimens | Pathological Diagnosis (Oxford classification) |
| --- | --- | --- | --- | --- | --- | --- | --- |
| IgAN_01 | IgAN | female | 34 | 115.27 | 1 | kidney tissue | IgAN (M1E0S0T0C0) |
| IgAN_02 | IgAN | female | 38 | 70.27 | 3a | kidney tissue | IgAN (M1E0S0T0C0) |
| IgAN_03 | IgAN | female | 34 | 105.15 | 1 | kidney tissue | IgAN (M1E0S0T0C0) |
| IgAN_04 | IgAN | female | 60 | 23.21 | 4 | kidney tissue | IgAN (M1E1S0T0C2) |
| IgAN_05 | IgAN | male | 34 | 92.56 | 1 | kidney tissue | IgAN (M1E0S1T1C1) |
| IgAN_06 | IgAN | female | 61 | 43.53 | 3b | kidney tissue | IgAN (M1E0S1T1C1) |
| IgAN_07 | IgAN | female | 28 | 113.83 | 1 | kidney tissue | IgAN (M1E0S0T0C0) |
| IgAN_08 | IgAN | female | 39 | 95.98 | 1 | kidney tissue | IgAN (M1E0S1T0C0) |
| IgAN_09 | IgAN | female | 54 | 44.25 | 3b | kidney tissue | IgAN (M1E0S1T2C0) |
| NC_KID_01 | normal control | female | 39 | 101.28 | 3a | kidney tissue | Renal cell carcinoma |
| NC_KID_02 | normal control | female | 36 | 109.43 | 3a | kidney tissue | Renal angiomyolipoma |
| NC_KID_03 | normal control | female | 58 | 97.87 | 3a | kidney tissue | kidney stones |
| NC_KID_04 | normal control | female | 33 | 75.64 | None | kidney tissue | High-grade invasive papillary urothelial carcinoma of the renal pelvis |
| NC_KID_05 | normal control | male | 59 | 75.18 | None | kidney tissue | Clear cell renal cell carcinoma |
| NC_KID_06 | normal control | female | 35 | 112.35 | None | kidney tissue | Clear cell renal cell carcinoma |
| NC_KID_07 | normal control | female | 37 | 108.66 | None | kidney tissue | Clear cell renal cell carcinoma |
| NC_KID_08 | normal control | female | 64 | 79.14 | None | kidney tissue | Chromophobe Renal Cell Carcinoma |
| NC_KID_09 | normal control | male | 60 | 94.26 | None | kidney tissue | Papillary Renal Cell Carcinoma |

Abbreviations: IgAN, IgA Nephropathy; NC, Normal Control; KID, Kidney Tissue; eGFR, estimated Glomerular Filtration Rate; CKD, Chronic Kidney Disease.
