# Supplementary material for: Bruceine A protects nuclear receptor 4A1 from ubiquitin-degradation to alleviate mesangial proliferative glomerulonephritis
Source: Signal Transduct Target Ther. 2025 Dec 5;10:397. doi: 10.1038/s41392-025-02495-2 (PMC12678413; doi:10.1038/s41392-025-02495-2)
Supplement: Supplementary file 7 — Table 6 [file 41392_2025_2495_MOESM7_ESM.docx]

**Table 6. Demographic and Clinical Characteristics of Single-Cell Transcriptome Renal Tissue Samples**

| Variables | IgAN (n=9） | NC (n=9） | *P* value |
| --- | --- | --- | --- |
| Gender (n,%) |  |  | 0.527 |
| male | 1 (11.1) | 2 (22.2) |  |
| female | 8 (88.9) | 7 (77.8) |  |
| Age [years,*M*（*P*25-*P*75）] | 38.0 (34.0-57.0) | 39.0 (35.5-59.5) | 0.452 |
| Laboratory values |  |  |  |
| Serum creatinine (μmol/L) | 90.0 (65.0-118.5) | 66.0 (62.5-73.5) | 0.133 |
| Urea (mmol/L) | 5.4 ± 2.1 | 4.9 ± 1.7 | 0.643 |
| Uric Acid (μmol/L) | 362.0 (301.0-419.0) | 302.5 (295.0-337.0) | 0.194 |
| eGFR [ml/(min•1.73m^2^)] | 78.2 ± 34.2 | 94.9 ± 14.8 | 0.207 |
| UPCR (mg/g) | 965.2 ± 898.2 | — | — |
| ALT (U/L) | 23.8 ± 19.7 | 19.7 ± 12.6 | 0.605 |
| AST (U/L) | 18.0(16.5-23.5) | 18.0 (15.5-22.5) | 0.929 |
| Hemoglobin (g/L) | 130.2 ± 17.8 | 125.6 ± 19.7 | 0.605 |
| WBC (10^9/L) | 7.5 ± 1.7 | 7.2 ± 0.9 | 0.610 |
| RBC (10^12/L) | 273.0 (252.0-351.5) | 229.0 (204.5-299.5) | 0.171 |
| PLT (10^9/L) | 291.6 ± 57.0 | 252.2 ± 55.4 | 0.157 |
| Serum Calcium (mmol/L) | 2.3 ± 0.1 | 2.4 ± 0.1 | 0.378 |
| Phosphorus (mmol/L) | 1.1 ± 0.2 | 1.1 ± 0.1 | 0.608 |
| Potassium (mmol/L) | 3.9 ± 0.4 | 3.9 ± 0.5 | 0.960 |
| FPG (mmol/L) | 4.9 (4.8-6.4) | 5.8(5.4-6.6) | 0.102 |
| HbA1c (%) | 5.5 ± 0.6 | 5.8 ± 0.4 | 0.421 |
| Triglyceride (mmol/L) | 1.8 ± 0.9 | 1.5 ± 0.5 | 0.534 |
| Total Cholesterol (mmol/L) | 4.79 ± 1.08 | 4.67 ± 0.77 | 0.809 |
| HDL-C (mmol/L) | 1.3 ± 0.4 | 1.3 ± 0.3 | 0.862 |
| LDL-C (mmol/L) | 3.0 ± 1.0 | 2.8 ± 0.5 | 0.553 |

Abbreviations: IgAN, IgA Nephropathy; NC, normal Control; eGFR, estimated Glomerular Filtration Rate; UPCR, urinary protein-to-creatinine ratio; ALT, alanine aminotransferase; AST, aspartate aminotransferase; WBC, white blood cell; RBC, red blood cell; PLT, blood platelet; FPG, fasting plasma glucose; HbA1c, glycated Hemoglobin; HDL-C, high density lipoprotein cholesterol; LDL-C, low-density lipoprotein cholesterol.
